# Reductive Deoxygenation of Aromatic Aldehydes and Ketones with Boron Triiodide

**DOI:** 10.1021/acs.joc.6c01013

**Published:** 2026-08-13

**Authors:** Thomas M. Chiarella, Anthony P. Furrule, Florence J. Williams

**Affiliations:** 4083 University of Iowa, Iowa City, Iowa 52246, United States

## Abstract

Deoxygenation of carbonyls is an area of value in synthetic transformations because it allows for the traceless utilization of carbonyl chemistry, a mainstay in synthetic manipulations. Since oxygenation patterns are common in biological settings, deoxygenation can also be used to valorize biologically derived feedstocks. Herein, we describe the use of potassium borohydride and iodine, two common and benchtop-stable reagents, to generate boron triiodide in situ, which performs full deoxygenation of aryl aldehydes and ketones. For aldehydes, benzyl iodide products are formed, whereas for ketones, the fully reduced methylene is produced. Byproducts upon workup are low-toxicity boric acid and iodide salts, making the process practical and appealing for a wide range of applications.

## Graphical abstract

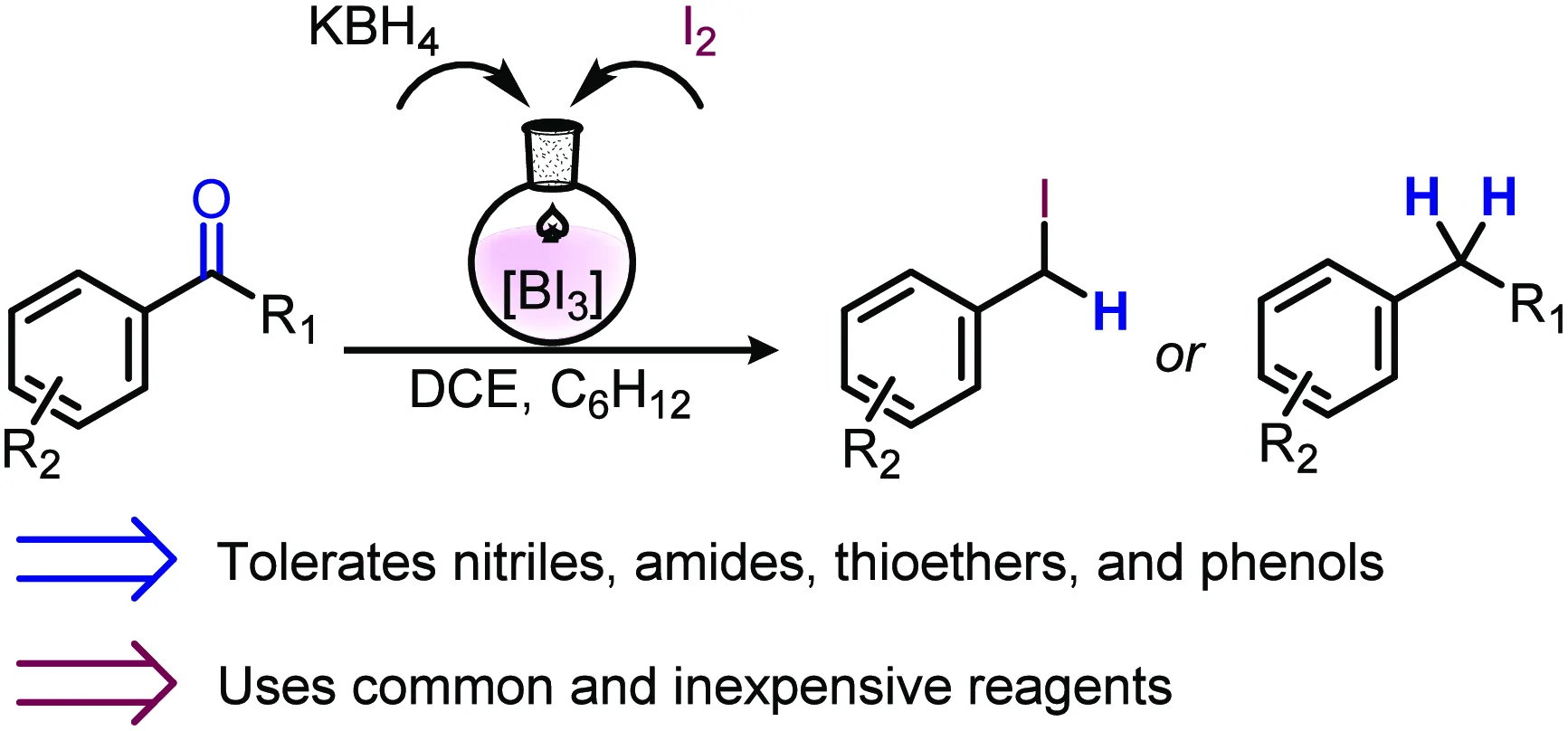

## Introduction

The synthetic strategy in organic chemistry has long centered around oxygenated functionality. Whether through the utilization of acidic hydrogens adjacent to carbonyls, the ease of nucleophilic attack at carbonyl carbons, electrophilic radicals selectively targeting electron rich α-hydrogens on alcohols and ethers, or the ability to use oxygen as a leaving group upon activation (e.g., as a tosylate or mesylate), oxygen-containing groups remain focal points in the construction of carbon-based compounds. This central role is facilitated by the ease of oxidation state manipulation on oxygen-bearing carbons (Figure 1a).

**Figure 1:**
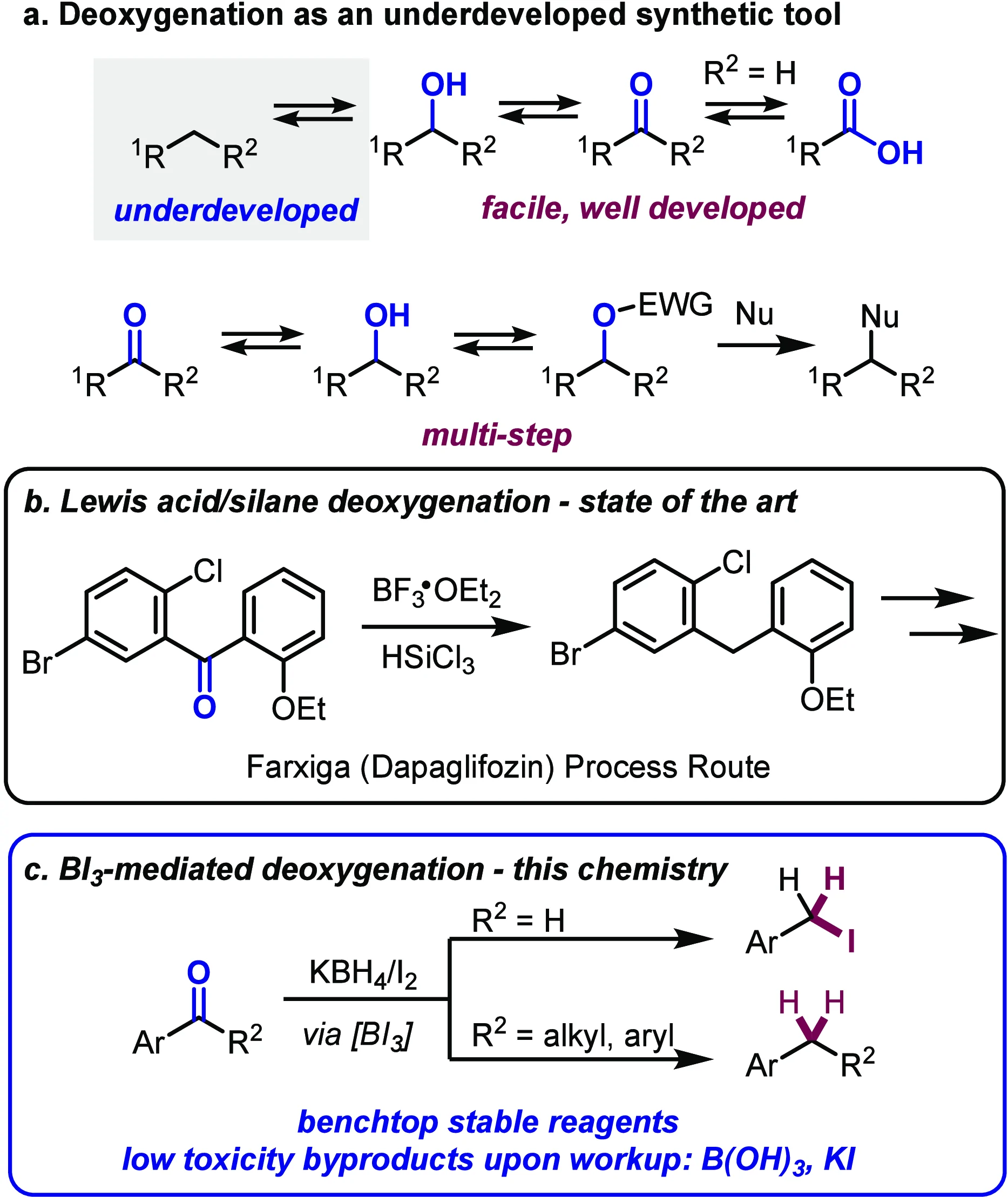
Deoxygenation strategies in organic synthesis.

Full reduction/deoxygenation traditionally involves harsh reaction conditions (e.g., Wolff–Kishner or Clemmensen reductions) or multiple steps (for instance, conversion of an alcohol to a leaving group followed by nucleophilic displacement).^1–3^ This history may be one reason why deoxygenation reactions are found regularly in total synthesis publications but less frequently in medicinal chemistry routes.^4^ Nevertheless, deoxygenation is employed on the process scale to access diarylmethanes, such as for drugs empaglifozin and dapaglifozin, the top 3 and 5 small molecule drugs, respectively, by sales in 2024 (Figure 1b).^5–7^

The state of the art for deoxygenation, particularly from the aldehyde, ketone, or carboxylic acid starting materials, involves the use of Lewis acids in combination with hydridic silanes or hydrogen gas.^1,2,8^ Most methods use boron Lewis acids for these transformations, with BCF (tris-pentafluorophenylborane) being the most frequently used. Recent shifts away from BCF, which requires careful handling under inert conditions, have led to the adoption of BF_3_ adducts and boronic acids.^8^ Transition-metal catalysts can also be used with hydridic silanes or borohydride reagents.^9–13^ As this field evolves, a focus on practical reagents that have low toxicity and low safety risks has emerged.^2,11,14^

In addition to full reduction, another valuable transformation is the partial reduction of carbonyls, followed by nucleophilic displacement. Other than amines, which can perform reductive aminations,^15^ most nucleophiles are incorporated using a three-step reaction sequence: (1) reduction to the alcohol, (2) conversion of the alcohol to a leaving group, and (3) displacement of the alcohol by a nucleophile (Figure 1A). For halogen nucleophiles, a handful of reports have found that these three steps can be performed in one reaction.^16–18^

Herein, we report a new method to perform full reduction/deoxygenation of aryl ketones as well as partial reduction and iododeoxygenation of aryl aldehydes. These transformations utilize benchtop-stable and common KBH_4_/I_2_ to generate BI_3_ in situ and occur in 1–2 h at room temperature. The byproducts have low toxicity (boric acid and KI), making this strategy an appealing option for many applications.^19,20^ It is noteworthy that the reaction requires an increased pressure to generate the BI_3_ intermediate, and hydrogen iodide (HI) is an intermediate of this reaction.

## Results and Discussion

We began optimization studies with the conversion of 4-bromobenzaldehyde to 4-bromobenzyl iodide (Table 1) using our laboratory’s optimized method for the generation of BI_3_ from KBH_4_ and iodine.^21^ Both iodine and KBH_4_ are required for the reaction (entries 1 and 2, respectively), as well as a BI_3_ pregeneration step (entries 3–12 vs entry 15). When KBH_4_ was used alone, only 6% yield of the alcohol product was observed based on NMR spectroscopy, likely due to the low solubility of KBH_4_ in cyclohexane. As in earlier studies, BI_3_ generation can be performed in any alkane solvent that has a sufficiently high boiling point (≥70 °C, entry 8).^21^ DCE was used as a cosolvent to improve the solubility for later scope investigations, but it is not a necessary component of the reaction (entries 9 and 10). Arene solvents can also be used for the generation of BI_3_ and the final reaction solvent, although when toluene was used, a small amount of the Friedel–Crafts product with toluene was observed (entry 11). Increasing the number of equivalents of BI_3_ relative to starting material resulted in over-reduction to the methyl group (entry 10).

**Table 1:**
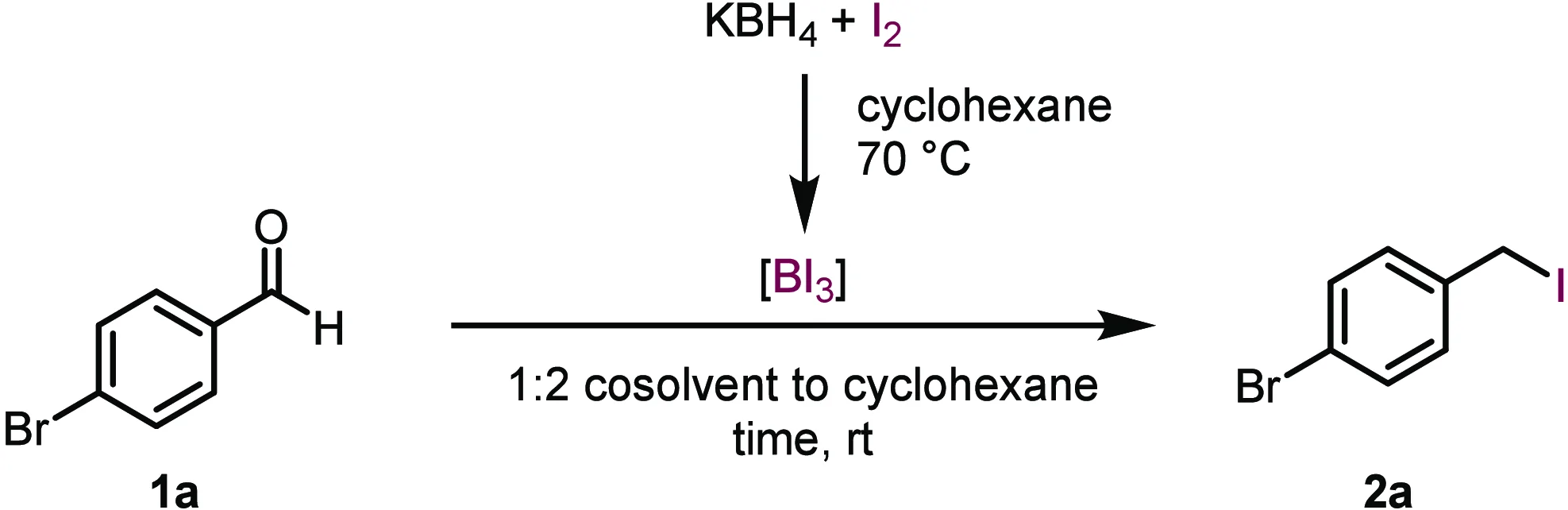
Optimization of Aldehyde Reduction and Iodination

entry	cosolvent	KBH_4_ (equiv)	I_2_ (equiv)	time (h)	concentration (M)	NMR yield (%)
1	DCE	2	0	1	0.03	0^a^
2	DCE	0	3	1	0.03	0
3	DCE	1	2	1	0.03	88
4	DCE	1.5	3	1	0.03	94
5	DCE	1.5	3	1	0.07	68
6	DCE	2	4	1	0.03	97
7	DCE	2	4	0.25	0.03	74
8^b^	DCE	2	4	1	0.03	96
9	none	1.5	3	1	0.03	94
10	none	5	10	1	0.03	72^c^
11	toluene	1.5	3	1	0.03	78
12^d^	benzene	2	4	1	0.03	69
13^e^	DCE	0	0	1	0.03	15
14^f^	DCE	0	0	1	0.03	77
15^g^	none	2	4	1	0.03	trace

a A 6% conversion to alcohol was observed.

b Heptane was used instead of cyclohexane to generate BI_3_.

c An 18% NMR yield of the methyl product was observed.

d BI_3_ generation was also carried out in benzene.

e The reaction was performed with 2 equiv of BI_3_.

f The reaction was performed with 2 equiv of BI_3_ and 1.5 equiv of water.

g No pregeneration of BI_3_; all components were mixed and then heated at 70 °C.

Pure BI_3_ can be used to perform the same reaction, verifying that BI_3_ is likely the active agent in this transformation, although stoichiometric water is important for efficient conversion (entries 13 and 14). With in situ-generated BI_3_, sufficient water to promote the reaction likely originates from the KBH_4_ or the solvents used, which are not dried prior to use.

Though 1.5 equiv of KBH_4_ and 3 equiv of iodine were sufficient for excellent conversion to 4-bromobenzyl iodide (entry 3), 2 equiv of KBH_4_ and 4 equiv of iodine were used for scope investigations to account for any batch variability in BI_3_ generation (entry 4). The fact that a rigorously dry solvent or reagents are not required for BI_3_ generation increases the simplicity of the procedure, but varying amounts of moisture, particularly on humid days, can have a modest effect on the rate and yield of the BI_3_ generation step.

With the optimal conditions determined, a substrate scope was evaluated (Scheme 1). *Ortho*, *meta*, and *para* substitutions were tolerated without an issue, including 1,3,5-trimethylbenzaldehyde (**2b**). However, in the case of orthohalogenated substrates **2f** and **2g**, extended reaction times were needed. A variety of halogenations were tolerated (**2a**, **2f**, and **2g**), including the trifluoromethyl group (**2g** and **2j**), which has been shown to be sensitive to boron trihalide-mediated halogen exchange in other reports.^22^ Naphthyl substrate **2d** was run for only 30 min; longer reaction times produced the fully reduced methyl product. It is likely that the extended arene facilitates over-reduction due to its ability to stabilize cationic or radical intermediates (vide infra). Although nitrile **2e** exhibited slightly decreased yields, like amide **2h**, there was no evidence of nitrile reduction. For these two substrates, as well as thioether **2i**, we hypothesize that the Lewis basic sites coordinate and sequester BI_3_, slowing the reaction. For nitriles **2e** and thioether **2i**, additional equivalents of BI_3_ were sufficient to allow for efficient conversion. Notably, no evidence of thioether degradation (e.g., cleavage or iodine-mediated oxidation) was observed with substrate **2i**. In the case of amide **2h**, the use of pure BI_3_ improved yields as compared with using in situ-generated BI_3_. The reaction also performed well on the gram scale (**2a**).

**Scheme 1:**
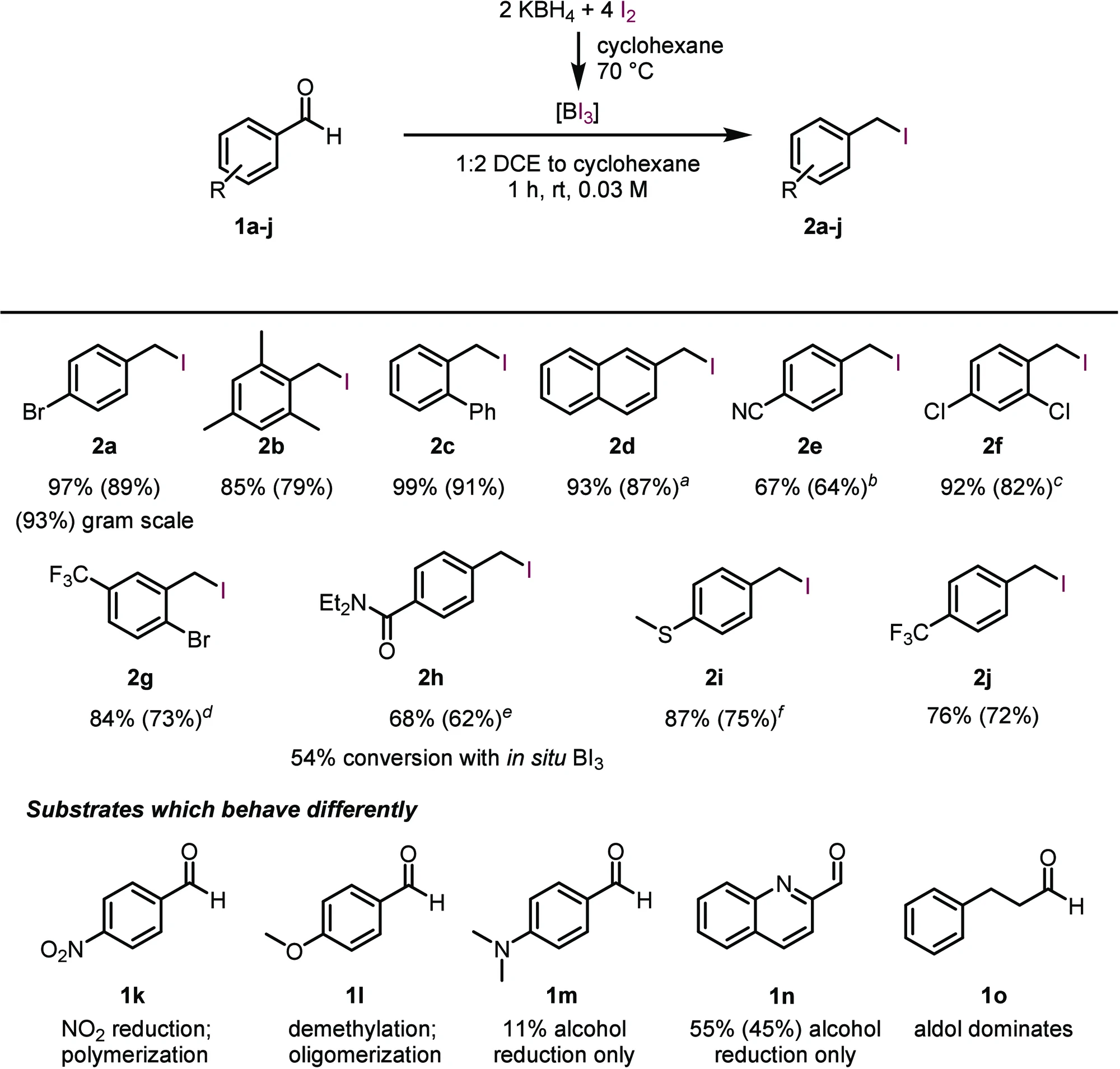
Aldehyde Substrate Scope^a^

a Reaction was performed for 30 min instead of 1 h.
b Reaction was run with 4 and 8 equiv of KBH_4_ and I_2_, respectively.
c Reaction was run for 3 h.
d Reaction was performed for 16 h.
e Reaction was run with 4 equiv of pure BI_3_ and 3 equiv of H_2_O.
f Reaction was performed with 3 and 6 equiv of KBH_4_ and I_2_, respectively.
g All reactions were performed on a 0.4 mmol scale at 0.03 M. BI_3_ was generated in a 25 mL round-bottom flask. Once BI_3_ generation was complete, the reaction flask was purged with N_2_. Conversions to the product are based on ^1^H NMR spectroscopy using 1,3,5-trimethoxybenzene as an internal standard. Isolated yields are given in parentheses.

Several unsuccessful examples were noted. An apparent polymerization occurred with 4-nitrobenzaldehyde (**1k**), which is assumed to arise following the reduction of the nitro group.^21^ Such a reduction would generate a nucleophilic aniline in the presence of an electrophilic benzyl iodide. Similarly, although anisaldehyde (**1l**) converts into the benzyl iodide, demethylation was concomitantly observed (33% 4-hydroxyphenyl iodomethane) along with decomposition products that appear to arise from oligomerization. In the case of **1m** and **1n**, only alcohol was observed without iododeoxygenation. Finally, when an aliphatic aldehyde (**1o**) was used, aldol condensation products (dimerization) were prevalent.

For aryl ketones, BI_3_ treatment fully reduced the carbonyl to an aryl methylene. Optimization studies determined that slightly altered conditions were needed for this transformation (more equivalents of KBH_4_ and iodine, lower concentration, and a reaction time of 2 h (Table 2)). When only 1 equiv of KBH_4_ and 2 equiv of I_2_ were used, benzhydryl iodide was observed by NMR, which will degrade in the presence of light or oxygen (entry 3).^23^ Similar to the aldehyde reduction studies, BI_3_ generation could be performed in cyclohexane or heptane (entry 7), and a cosolvent was not necessary for the reactivity (entry 10). DCE and toluene were both tolerated as cosolvents. Notably, no Friedel–Crafts adducts between the substrate and toluene were observed in entry 11. Finally, pure BI_3_ with a stoichiometric amount of water was confirmed to perform the reaction similar to that of in situ-generated BI_3_ (entry 12), and pregeneration of BI_3_ was necessary for a successful reaction (entry 13).

**Table 2:**
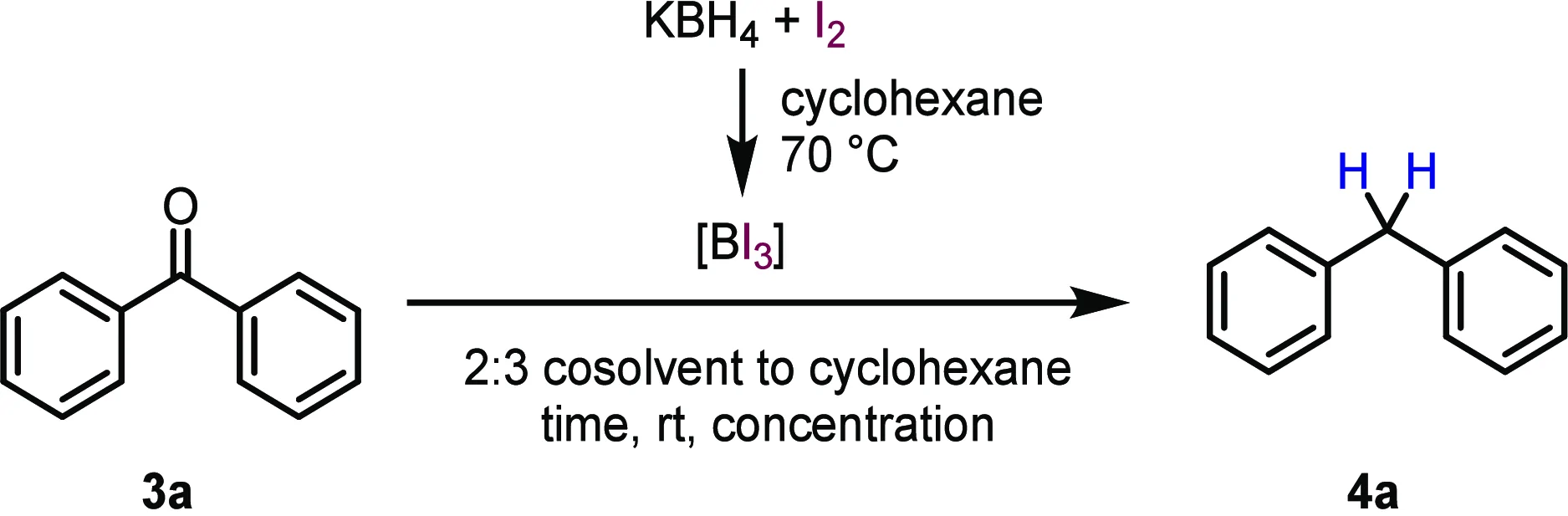
Optimization of Ketone Reduction with Benzophenone

entry	cosolvent	KBH_4_ (equiv)	I_2_ (equiv)	time (h)	concentration (M)	NMR yield (%)
1	DCE	4	0	2	0.02	0^a^
2	DCE	0	8	2	0.02	0^a^
3	DCE	1	2	2	0.02	decomposed
4	DCE	2	4	2	0.02	90
5	DCE	3	6	2	0.02	91
6	DCE	4	8	2	0.02	94
7^b^	DCE	4	8	2	0.02	92
8	DCE	4	8	1	0.02	90
9	DCE	4	8	2	0.04	87
10	none	4	8	2	0.02	85
11	toluene	4	8	2	0.02	88
12^c^	DCE	0	0	2	0.02	93
13^d^	none	4	8	2	0.02	trace

a The alcohol product was not observed either.

b BI_3_ was generated in heptane instead of cyclohexane.

c The reaction was performed with 4 equiv of pure BI_3_ and 3 equiv of water.

d All components were mixed and then heated 70 °C.

The conditions in entry 6 were used to examine the substrate scope (Scheme 2). Both benzophenones and aryl ketones performed well in this reduction, although aryl aliphatic ketones required greater KBH_4_ and iodine loadings, as well as extended reaction times. Sterically demanding *ortho* substituents, such as in *o*-bromoarene **3b**, did not negatively impact the reaction. Extended aromaticity (**3h**) and different halogenation patterns (**3b**, **3c**, and **3i**) were also well tolerated. Though alcohol **3g** resulted in a decreased yield, this entry is notable because hydroxyl groups can readily form complexes with BX_3_ reagents. When fluorenone **3e** was used, a significant amount of the fluorene dimer was observed (14% by NMR analysis and 12% isolated yield (see Scheme 4)). Low isolated yields when using aryl alkyl ketones **3h**–**3j** are due to the moderate volatility of the final products. Branched ketone **3k** performed only modestly (45%), largely stopping at the benzylic iodide (31%), suggesting that sterics play an important role in the final reduction step for ketones.

**Scheme 2:**
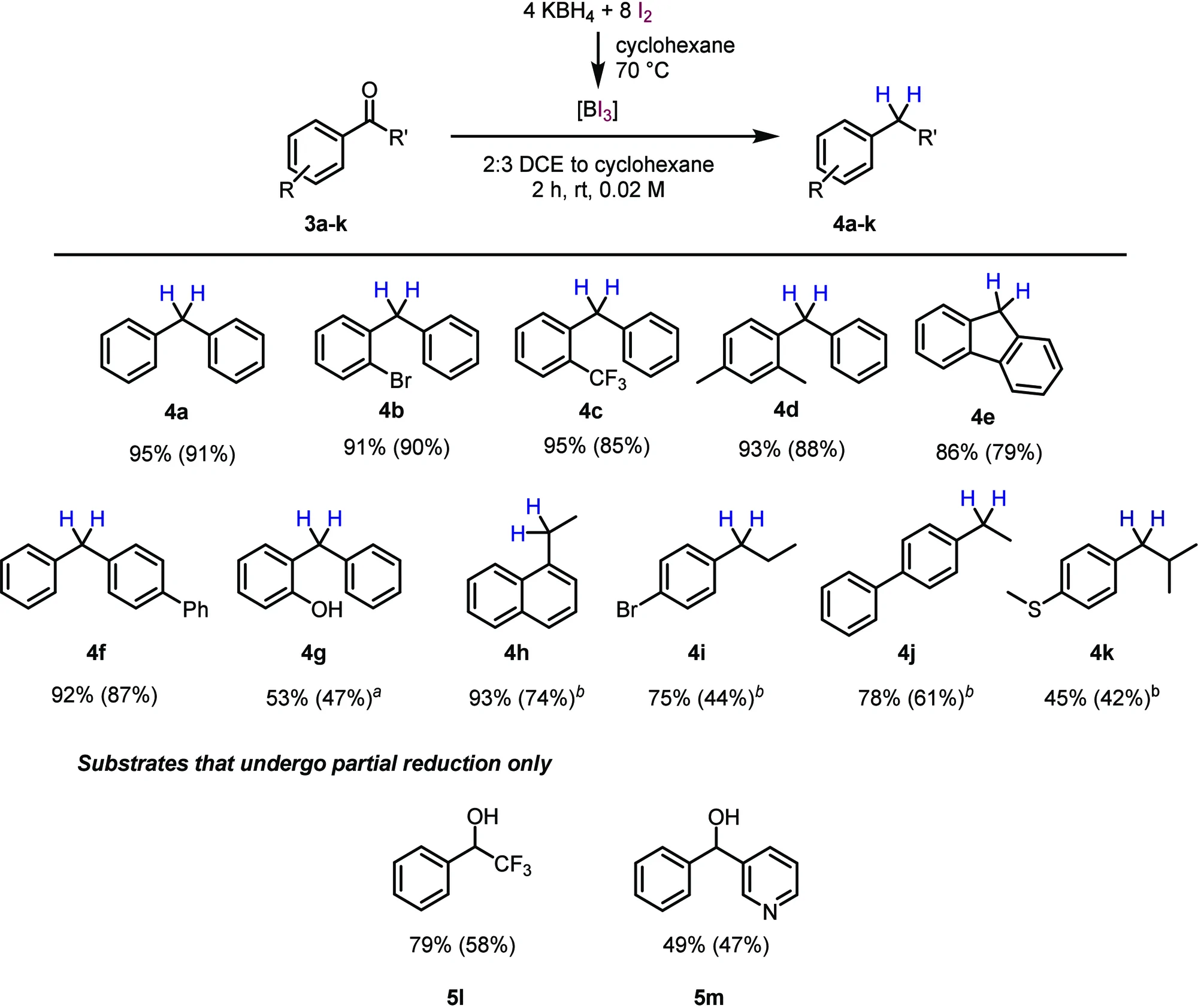
Ketone Substrate Scope^a^

a Reaction run at 70 °C.
b Reaction performed with 5 and 10 equiv of KBH_4_ and I_2_, respectively, for 6 h.
c All reactions were performed on a 0.4 mmol scale at 0.03 M. BI_3_ was generated in a 50 mL round-bottom flask. Once BI_3_ generation was complete, the reaction flask was purged with N_2_. Conversions to the product were determined by ^1^H NMR spectroscopy using 1,3,5-trimethoxybenzene as an internal standard. Isolated yields are given in parentheses.

Trifluoroacetophenone **3l** and pyridyl compound **3m** underwent partial reduction to alcohol only. Importantly, this reduction may also be BI_3_-mediated, as KBH_4_ treatment alone resulted in 14% conversion to **5l** and no conversion to **5m** under these conditions.

Additional mechanistic studies were performed to glean insight into plausible intermediates of the reaction pathway. First, we hypothesized that the reaction begins with a reduction of the substrate to the alcohol since substrates **3l** and **3m** generate alcohol products. Subjecting alcohol substrates **5a** and **5b** to the reaction conditions generated the benzyl iodide and methine products, respectively (Scheme 3). While these reactions can be facilitated by HI alone, literature reports require heating or an extended reaction time, suggesting an active role for BI_3_ in these conversions.^24,25^

**Scheme 3:**
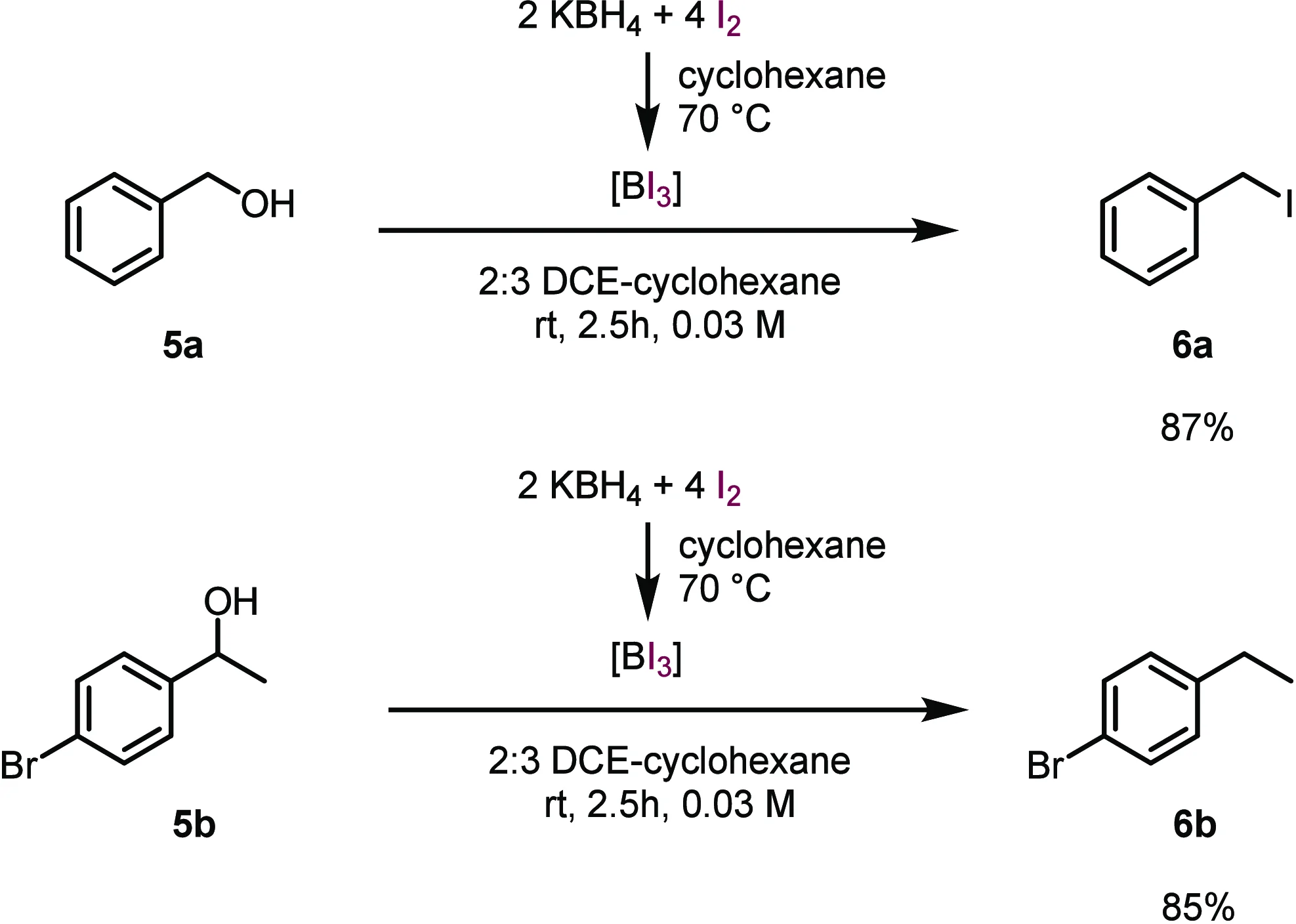
Reactions with Primary and Secondary Benzyl Alcohols

Fluorene substrate **3e** generated a significant amount of the fluorene dimer, which is suggestive of a fluorene radical intermediate (Scheme 4). However, common radical traps are incompatible with BI_3_,^21^ which precludes their use as mechanistic probes. Therefore, to test for radical intermediacy, cyclopropyl ketone **3o** was subjected to the reaction conditions. 1,4-Diphenylbutane was observed by GC-HRMS, indicating full reduction and ring opening of this species. However, we cannot exclude the mechanistic possibility of BI_3_ addition across the cyclopropane ring, followed by hydrodeboronation and reduction of the benzyl iodide, rather than radical-mediated ring opening (Scheme 4). Finally, when triphenylacetophenone **3n** was used, tetraphenylethylene was formed as the major product (Scheme 4), which supports the intermediacy of a cation.

**Scheme 4:**
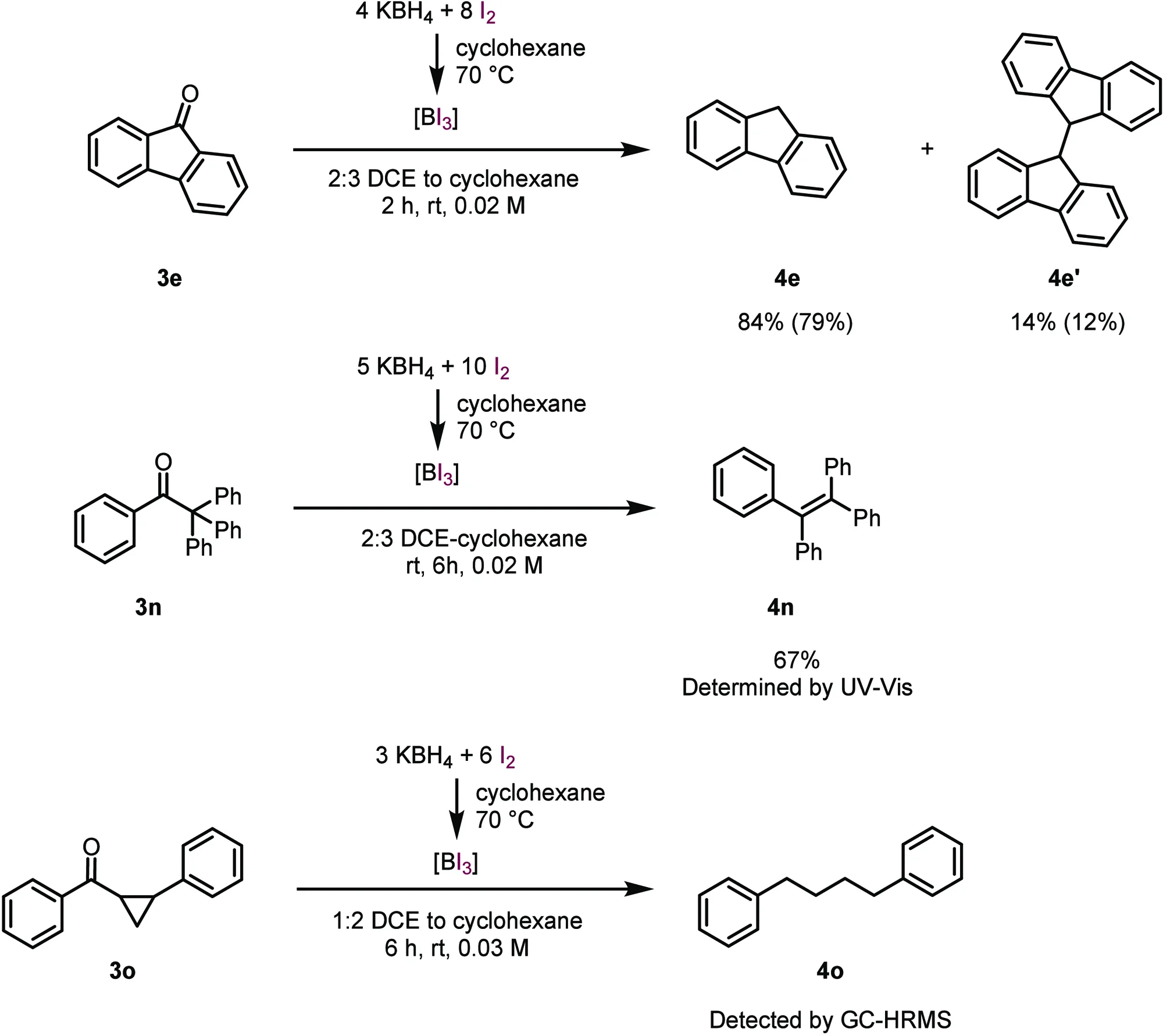
Mechanistic Tests

Given the reaction behavior observed in substrate scope studies, the observed byproducts, and mechanistic understanding obtained from other BI_3_-mediated reductions,^26^ a plausible mechanism can be presented (Scheme 5). Like BI_3_-mediated acene reductions, this reaction benefits from the presence of water and is generating some amount of HI as a byproduct.^26^ Therefore, the carbonyl of the substrate is likely activated through protonation (from HI or the BI_3_•OH_2_ adduct) or coordination to boron, facilitating attack by iodide to form the iodohydrin or boron-coordinated analogue. Then, similar to BI_3_-mediated acene reduction,^26^ a small amount of the benzyl iodide is homolytically cleaved to generate the iodine radical. This iodine radical catalyzes the formation of additional benzyl radicals through a radical propagation sequence, forming I_2_ as a byproduct. The benzyl radical then reacts with HI, which serves as a hydrogen atom donor. From the alcohol, an S_N_1 reaction occurs facilitated by BI_3_ to generate the benzyl iodide. This type of substitution is typical for boron trihalides.^27,28^ For aryl ketone substrates, another iodine radical-catalyzed propagation sequence is proposed to furnish the final product. The lower stability of a primary benzylic radical generated from aldehyde substrates, as compared to secondary benzylic radicals from ketone substrates, may explain why the benzyl iodide is isolated as a product from aldehydes rather than the fully reduced methyl/tolyl products. This explanation would also be consistent with the tendency of naphthyl aldehyde substrate **1d** to over-reduce, as the extended aromaticity would provide additional stability to the intermediate radical.

**Scheme 5:**
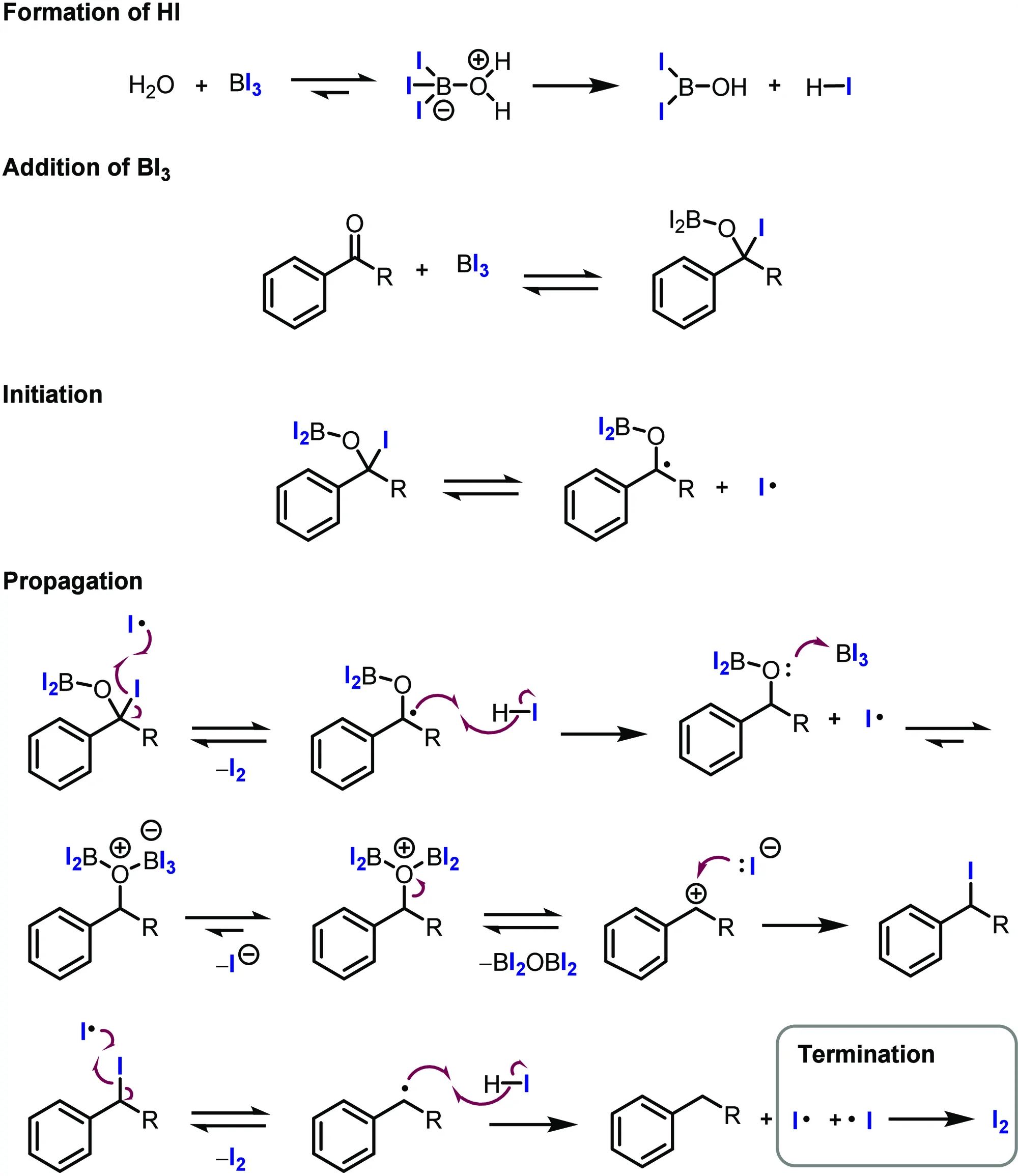
A Proposed Mechanism

To probe the effect of substrate electronic character on reaction rate, an LFER (linear free energy relationship) analysis was performed (Scheme 6). Relative rates (*k*_X_/*k*_H_) were determined by GC analysis of benzyl iodide products in competition experiments. Interestingly, no linear fit was obtained using traditional Hammett values (*R* ^2^ = 0.0043 (see the )).^29^ A partial fit was realized when using radical stabilization values (Scheme 6 (*R* ^2^ = 0.99; ρ = 1.193).^30^ However, substrate *p*-trifluoromethylbenzaldehyde (**1j**) was an outlier from this trend. Outliers in LFER plots typically occur either when the mechanism of the reaction is different for the outlier substrate(s), such as a different rate-determining step, or when there are competing decomposition pathways complicating analysis. In this assay, GC results for substrate **1j** indicated that only 53–56% of the material was accounted for when measuring remaining starting material, intermediate alcohol, or product, in stark contrast to the other substrates (90–100%). The GC trace did not show significant unidentified peaks (see the ). These data suggest that a decomposition pathway is complicating the LFER analysis for trifluoromethyl substrate **1j**, presumably oligomerization to generate low-volatility byproduct(s).

**Scheme 6:**
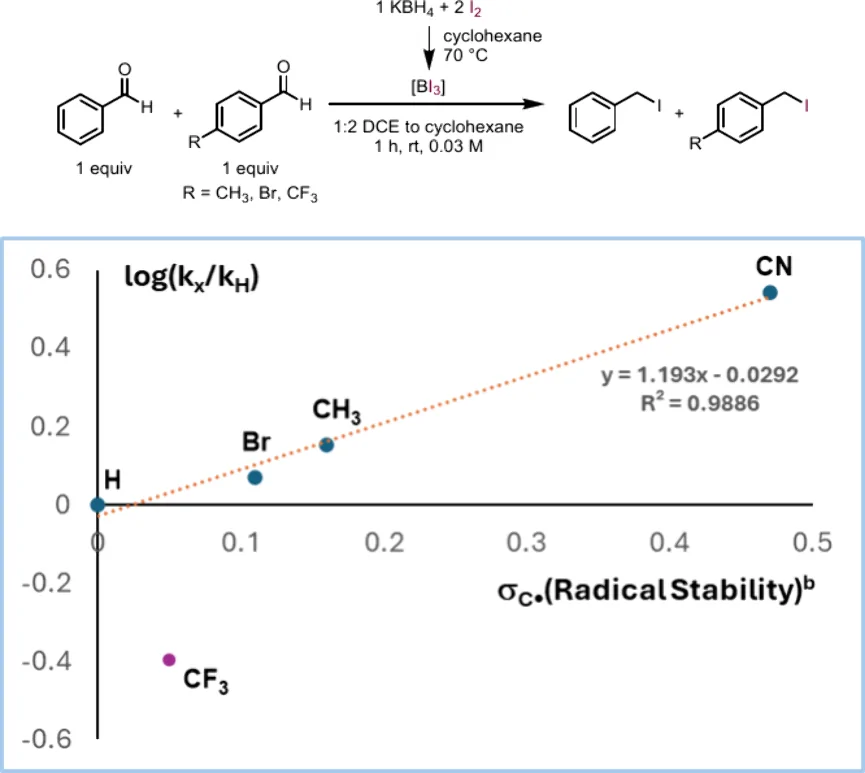
LFER Plot^a^

a The ratio of rate constants was determined based on the ratio of benzyl iodide products in a competition experiment, as determined by GC quantification.
b Values for radical stability are taken from ref 30.

Given the high degree of fit for the other four data points, we propose that a radical is involved in the rate-determining step, which would mean that either the generation of the benzylic radical or hydrogen atom transfer is rate-limiting. Substrates that halt at the alcohol (**1m**, **1n**, **3l**, and **3m**) may be sufficiently electron-deficient (before or after BI_3_ coordination) that the rate of the S_N_1 reaction to install the benzylic iodide becomes rate-determining.

## Conclusions

A boron triiodide-based metal-free method for reducing aromatic aldehydes to benzylic halides and deoxygenating aryl ketones to the corresponding alkane has been developed. The reaction does not require heating, uses common, nonflammable, and benchtop-stable reagents to generate BI_3_, and tolerates several functional groups, including those sensitive to reduction (e.g., CN) and those that present steric encumbrance. Lewis basic functionality (e.g., thioether and amide) requires additional equivalents of BI_3_ but does not prevent reactivity with the Lewis acidic BI_3_ reagent. The reaction conditions do not tolerate alkyl ethers, due to ether cleavage,^31,32^ nitroarenes, due to reduction to the aniline,^21^ or dialkyl ketones, due to aldol reactivity.^33,34^ Additionally, strongly electron-deficient substrates undergo only partial reduction to the alcohol.

A mechanism that starts with the iodohydrin or the corresponding boron-coordinated analogue has been proposed. The iodine radical acts as a catalyst for the generation of benzylic radicals, and hydrogen iodide serves as the ultimate reductant. Using HI alone requires extended reaction times and increased temperatures, supporting an active role of the boron reagent in this process.^24,25^

Overall, this method constitutes an appealing and practical option for deoxygenation of aryl aldehydes and ketones due to the simple reagents and reaction setup as well as the low-toxicity byproducts (boric acid, iodide salts, and I_2_).

## Supplementary Material











## Data Availability

The data underlying this study are available in the published article and its .

## Notes

FAIR data, including the primary NMR FID files, for compounds **2a–2n** (ZIP)
FAIR data, including the primary NMR FID files, for compounds **4a–4n** (ZIP)
FAIR data, including the primary NMR FID files, for compounds **5l** (ZIP)
FAIR data, including the primary NMR FID files, for compounds **5m** (ZIP)
Experimental procedures, characterization data, and NMR spectra of all compounds (PDF)
