# Supplementary material for: Reductive Deoxygenation of Aromatic Aldehydes and Ketones with Boron Triiodide
Source: J Org Chem. 2026 Aug 13;91(34):11704–11. doi: 10.1021/acs.joc.6c01013 (PMC13532174; doi:10.1021/acs.joc.6c01013)
Supplement: Supplementary file 1 [file jo6c01013_si_005.pdf]

Supporting Information

Reductive Deoxygenation of Aromatic Aldehydes and Ketones with Boron Triiodide

Thomas M. Chiarella,[a] Anthony P. Furrule,[a] and Florence J. Williams*[a]

[a]University of Iowa, Iowa City, IA, 52242

Contents

1.1. General Information S-2

1.2 Experimental Methods S-2

1.2.1 Preparation of Starting Materials S-2

1.2.2 Synthesis of benzyl iodides from benzaldehydes S-3

1.2.3 Alternate procedures S-4

1.2.4 Synthesis of alkanes from aromatic ketones S-5

1.2.5 Gram scale experiment S-6

1.3. Mechanistic Studies S-7

1.4. LFER Experiment S-10

1.5. Characterization data S-12

1.6. Spectral data S-21

2. References: S-69

1. Supplemental Data

1.1. General Information

All reactions were performed under an N_2 atmosphere unless otherwise stated. Solvents were used as received, without drying by distillation, mol sieves treatment or solvent system. Deuterated chloroform ($CDCl_3$) was stored in a container with oven-dried (>200 °C) molecular sieves (4 Å). BI_3 = boron triiodide, DCE = 1,2-dichloroethane, DCM = dichloromethane, EtOAc = ethyl acetate, Na_2SO_4 = sodium sulfate, HCl = hydrochloric acid. All reagents were purchased from Sigma Aldrich, ThermoFisher Scientific, TCI, VWR, AmBeed, Oakwood, and Cambridge Isotope Laboratories, Inc. Hydrogen nuclear magnetic resonance (^{1}H-NMR) spectra were recorded at 300, 400, or 600 MHz Bruker instruments, and coupling constants (*J*) are reported in Hertz (Hz). Carbon nuclear magnetic resonance (^{13}C-NMR) spectra were recorded at 75, 100, or 150 MHz. Fluorine nuclear magnetic resonance (^{19}F-NMR) spectra were recorded at 282 or 377 MHz. Chemical shifts are reported in δ (ppm) and referenced to the residual protonated solvent (^{1}H) peaks, to the deuterated solvent (^{13}C) peaks: $CDCl_3$ (7.26 ppm, ^{1}H; 77.16 ppm, ^{13}C). s = singlet, d = doublet, t = triplet, q = quartet, m = multiplet, bs = broad singlet, etc. Structural assignments were made with additional information from gHSQC experiments UV/Vis data were recorded on a Beckman Coulter DU 720 spectrometer. GC data were collected on a Shimadzu GC-2010 spectrometer.

1.2 Experimental Methods

1.2.1 Preparation of Starting Materials

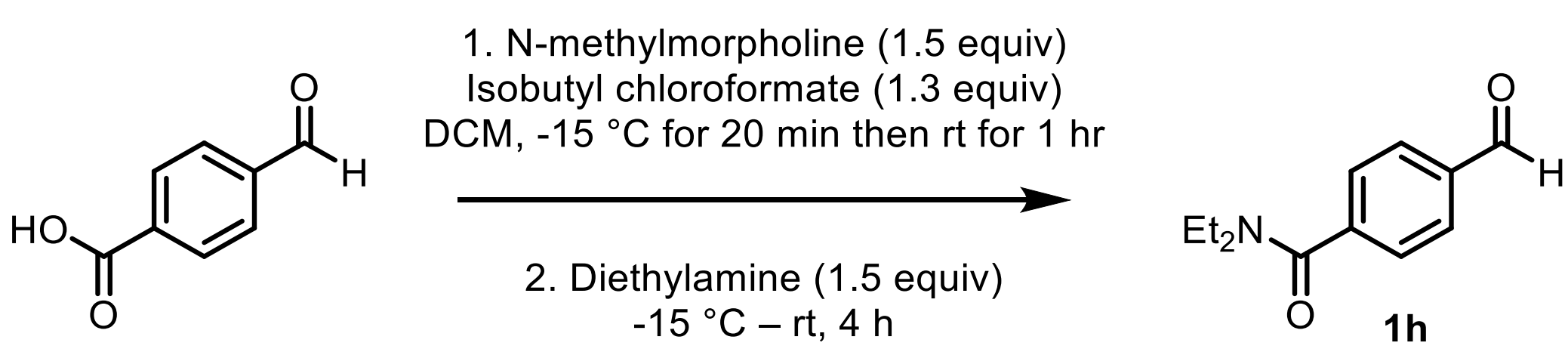

Scheme S1: Preparation of 4-formyl-*N,N*-diethylbenzamide

The dimethyl amide was prepared following literature procedure.[1] 4-formylbenzoic acid (1.50 g, 10.000 mmol, 1.00 equiv.), and DCM (15 mL) were added to a single neck 50 mL roundbottom flask with a stir bar. The flask was cooled to –15 °C and N-methylmorpholine (1.80 mL, 15.00 mmol. 1.50 equiv.) was added followed by the dropwise addition of isobutyl chloroformate (1.80 mL, 13.00 mmol, 1.30 equiv.). The reaction was allowed to stir for 20 min. at –15 °C, then warmed up to room temperature and stirred for 1 hr. After 1 hr., the reaction was cooled back down to –15 °C, and dimethylamine (1.70 mL, 15.00 mmol, 1.50 equiv.) was added dropwise. The reaction was run for 4 hr. and then added to a separatory funnel. The reaction mixture was extracted with 1 M HCl (3 x 20 mL) followed by a saturated aqueous sodium bicarbonate solution (10 mL). The organic fraction was dried over Na_2SO_4, and concentrated in vacuo. The crude oil was purified by column chromatography (1:1 hexane:EtOAc) to obtain the final product (1.088 g, 53% yield) as a light-yellow liquid. ^{1}H-NMR ($CDCl_3$, 400 MHz): δ 10.04 (s, 1H), 7.92 (d, J = 8.3 Hz, 2H), 7.52 (d, J = 8.0 Hz, 2H), 3.56 (m, 2H), 3.21 (m, 2H), 1.28-1.25 (m, 3H), 1.12-1.09 (m, 3H) ppm. ^{13}C{^{1}H}-NMR ($CDCl_3$, 100 MHz): δ 191.7, 170.0, 143.1, 136.7, 130.0, 127.1, 43.3, 39.5, 14.3, 13.0 ppm. Spectral data were consistent with literature values.[2]

1.2.2 Synthesis of benzyl iodides from benzaldehydes

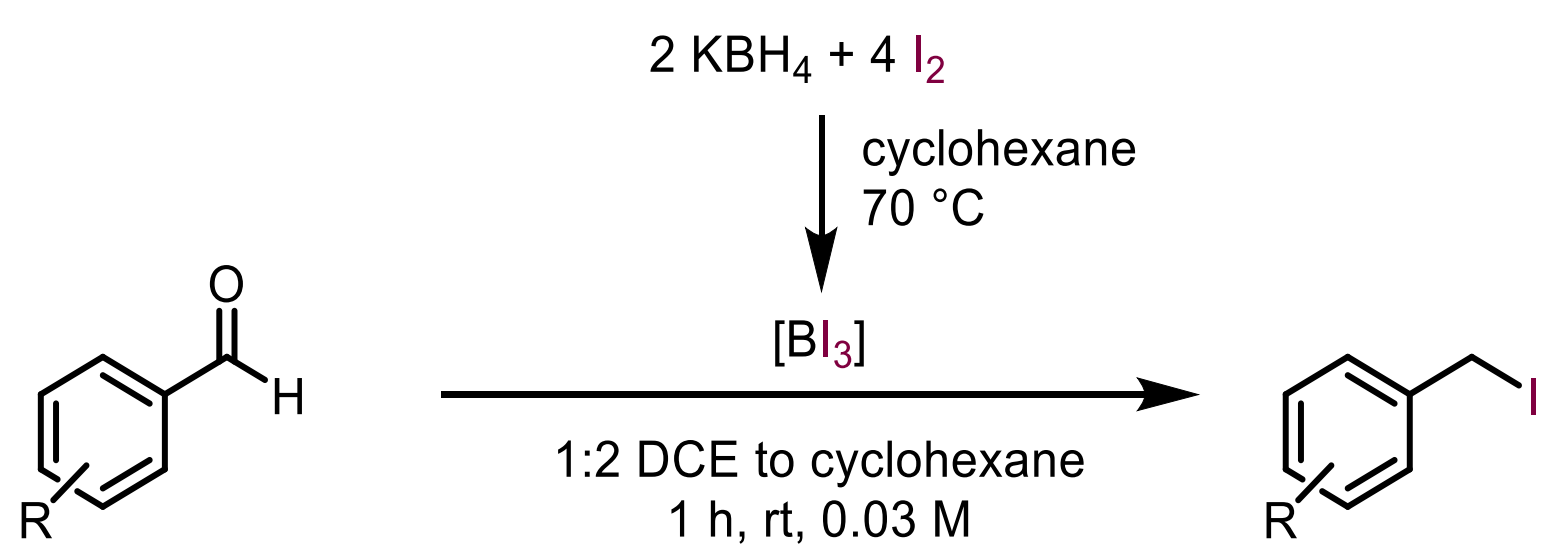

1a-i **2a-i**

General procedure for the conversion of benzaldehydes to benzyl iodides:

KBH_4 (43.2 mg, 0.800 mmol, 2.00 equiv.), I_2 (406.1 mg, 1.600 mmol, 4.000 equiv.) and cyclohexane (4 mL) were added to a 25 mL single neck round bottom flask equipped with a stir bar. The round bottom flask was capped with a rubber septum cap, and the septum was tightened with a copper wire. The flask was placed in a 70 °C oil bath and allowed to stir until BI_3 generation was complete. Completion of BI_3 generation can occur anywhere from 10 to 90

minutes and can be indicated by a change in color. Colors can range from completely clear to magenta.[3] The reaction was allowed to cool to room temperature and subsequently punctured with a needle, a nitrogen line was attached (note: a nitrogen line was not added during the BI_3 generation), and diluted with an additional 4 mL cyclohexane. Aldehyde (0.4 mmol) was weighed out in a 20 mL scintillation vial and dissolved in DCE (2 mL). The resulting aldehyde solution was injected into the round bottom flask containing *in situ* generated BI_3, after which a color change to dark purple was almost immediately observed. The vial was rinsed with another 2 mL of DCE and injected into the reaction mixture.

After 1 hour, the reaction was cooled to 0 °C and quenched with a 5% $Na_2S_2O_3$ solution, 10% ascorbic acid solution, or 10% sodium ascorbate solution (10 mL) while stirring. The contents were added to a separatory funnel, and the flask was washed with 10 mL EtOAc. The separatory funnel was shaken until all the iodine was consumed, as determined by loss of color, and the layers were separated. Aqueous phase was washed with EtOAc (2 x 10 mL). The organic fraction was then washed with brine (1 x 10mL), dried over Na_2SO_4 and concentrated in vacuo. The crude product was analyzed and quantified by ^{1}H-NMR spectroscopy using 1,3,5-trimethoxybenzene as an internal standard. Column chromatography with silica gel was used to isolate the final products.

1.2.3 Alternate procedures

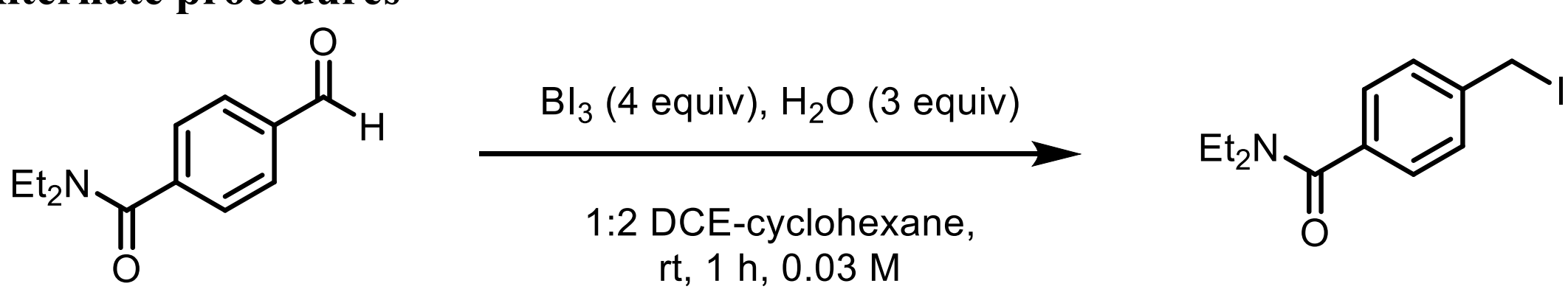

1h **2h**

Scheme S2: Preparation of 4-(iodomethyl)-*N,N*-diethylbenzamide using pure BI_3.

N,N-diethyl-4-formylbenzamide (41.1 mg, 0.200 mmol, 1.00 equiv.), DCE (2 mL), and cyclohexane (3 mL) were added to a 25 mL single neck round bottom flask equipped with a stir bar. The flask was capped with a rubber septum and an N_2 line was added. In the glovebox, BI_3 (313.2 mg, 0.800 mmol, 4.00 equiv.) was weighed in a vial. The vial was capped with a septum cap and taken out of the glovebox. Water (10.8 µL, 3 equiv.) was injected into the reaction mixture. To the vial containing BI_3, 0.5 mL cyclohexane was added to dissolve the BI_3, and the

solution was injected into the reaction flask. The vial was rinsed with an additional 0.5 mL cyclohexane and injected into the reaction flask in which the reaction became a dark purple.

After 1 hour, the reaction was cooled to 0 °C, and quenched with a 10% ascorbic acid solution (10 mL, aq). The contents were added to a separatory funnel, and the flask was washed with 10 mL EtOAc. The organic layer was separated, and the aqueous phase was washed with EtOAc (2 x 10mL). All organic layers were combined then washed with brine (1 x 10mL), dried over Na_2SO_4 and concentrated in vacuo. The crude product was analyzed and quantified by ^{1}H-NMR spectroscopy using 1,3,5-trimethoxybenzene as an internal standard. Column chromatography with silica gel was used to isolate the final product.

1.2.4 Synthesis of alkanes from aromatic ketones

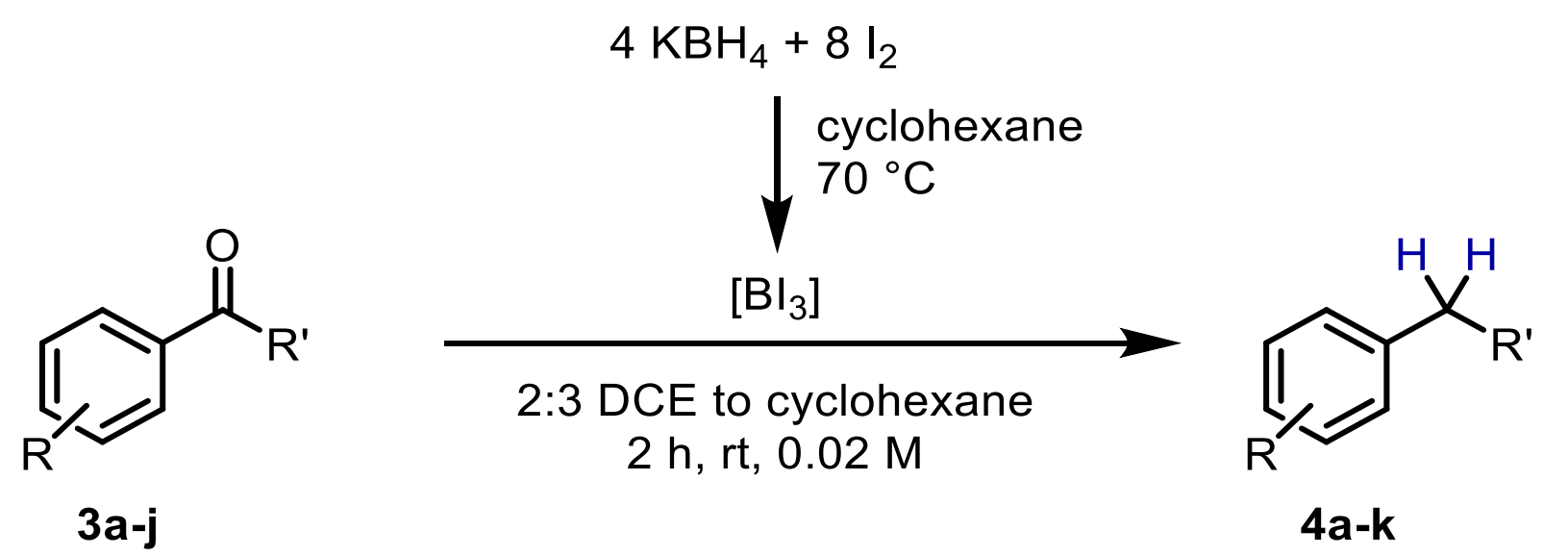

KBH_4 (43.2 mg, 0.800 mmol, 4.00 equiv.) and I_2 (406.1 mg, 1.600 mmol, 8.00 equiv.) and cyclohexane (4 mL) were added to a single neck 50 mL round bottom flask equipped with a stir bar. The flask was then capped with a rubber septum and tightened with a copper wire. The flask was placed in a 70 °C oil bath and allowed to stir until BI_3 generation was complete. Completion of BI_3 generation can occur anywhere from 10 to 90 minutes and can be indicated by a change in color. Colors can range from completely clear to magenta.[3] The reaction was allowed to cool to room temperature and subsequently punctured with a needle, a nitrogen line was attached (note: a nitrogen line was not added during the BI_3 generation), and diluted with an additional 2 mL cyclohexane. Ketone (0.2 mmol) was weighed out in a 20 mL scintillation vial and dissolved in DCE (2 mL). The resulting ketone solution was injected into the round bottom flask containing *in situ* generated BI_3, after which a color change to dark purple was almost immediately observed. The vial was rinsed with another 2 mL of DCE and injected into the reaction mixture.

After 2 hours, the reaction was cooled to 0 °C and quenched with a 5% $Na_2S_2O_3$ solution, 10% ascorbic acid solution, or 10% sodium ascorbate solution (10 mL) while stirring. The contents

were added to a separatory funnel, and the flask was washed with 10 mL EtOAc. The separatory funnel was shaken until all the iodine was consumed, as determined by loss of color, and the layers were separated. Aqueous phase was washed with EtOAc (2 x 10 mL). The organic fraction was then washed with brine (1 x 10mL), dried over Na_2SO_4 and concentrated in vacuo. The crude product was analyzed and quantified by ^{1}H-NMR spectroscopy using 1,3,5-trimethoxybenzene as an internal standard. Column chromatography was used to isolate the final products.

Note: For isolation, the reaction was run on a 0.4 mmol scale.

1.2.5 Gram scale experiment

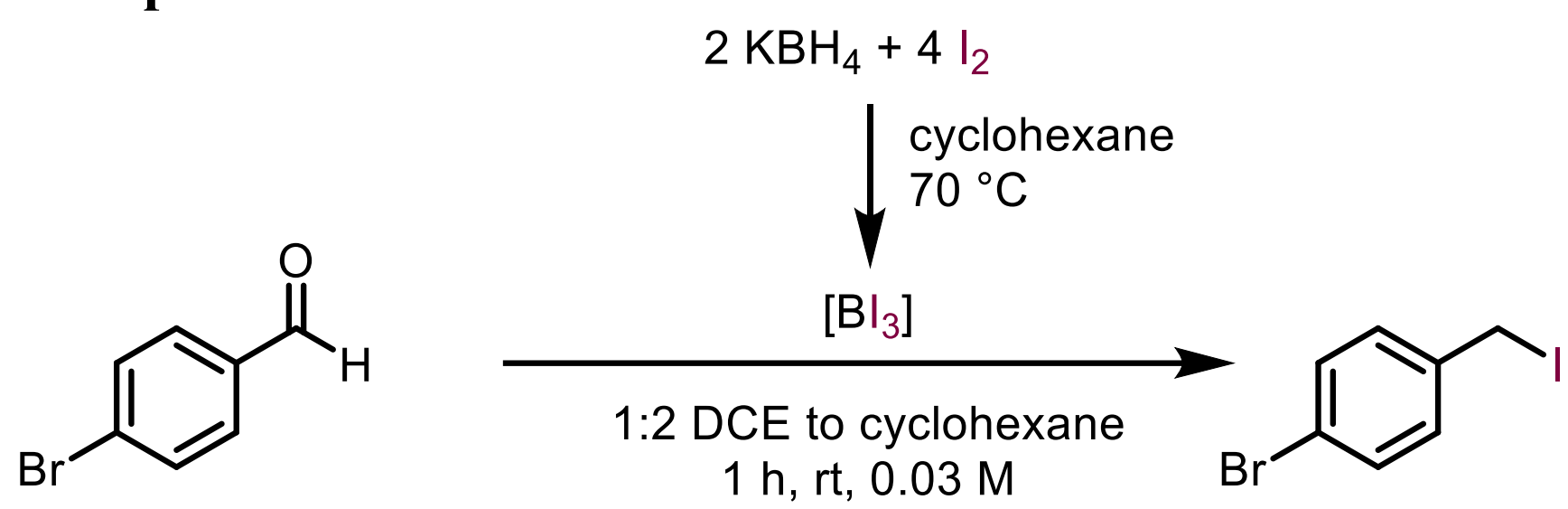

KBH_4 (0.647 g, 12.000 mmol, 2.00 equiv.), I_2 (6.09 g, 24.000 mmol, 4.000 equiv.) and cyclohexane (40 mL) were added to a 500 mL three-necked round bottom flask equipped with a stir bar. Each neck was capped with a rubber septum cap, and the septa were tightened with a copper wire. The flask was placed in a 70 °C oil bath and allowed to stir until the purple color of iodine disappeared (about 30 minutes). The reaction was allowed to cool to room temperature and subsequently punctured with a needle, and a nitrogen line was attached (note: a nitrogen line was not added during the BI_3 generation). The septum in the middle neck was removed, and quickly replaced with a 150 mL addition funnel. To the addition funnel was added 93 mL of cyclohexane, and was added to the solution. 4-bromobenzaldehyde (1.11 g, 6.0 mmol) was weighed out in a 125 mL Erlenmeyer flask and dissolved in DCE (33 mL). The resulting aldehyde solution was added to the addition funnel, and the solution was added into the round bottom flask containing *in situ* generated BI_3, after which a color change to dark purple was almost immediately observed. (Note: the solution does not need to be added dropwise). The vial was rinsed with another 34 mL of DCE and added into the addition funnel, and drained into the reaction mixture.

After 1 hour, the reaction was cooled to 0 °C and quenched with a 10% ascorbic acid solution in water (50 mL). The contents were added to a separatory funnel, and separated. The aqueous layer was washed with EtOAc (2 x 30 mL) The organic fractions were then washed with brine (1 x 50 mL) and dried over Na_2SO_4 and concentrated in vacuo. Purified by column chromatography (1% EtOAc in hexanes) to obtain desired product (1.66 g, 93% yield).

1.3. Mechanistic Studies

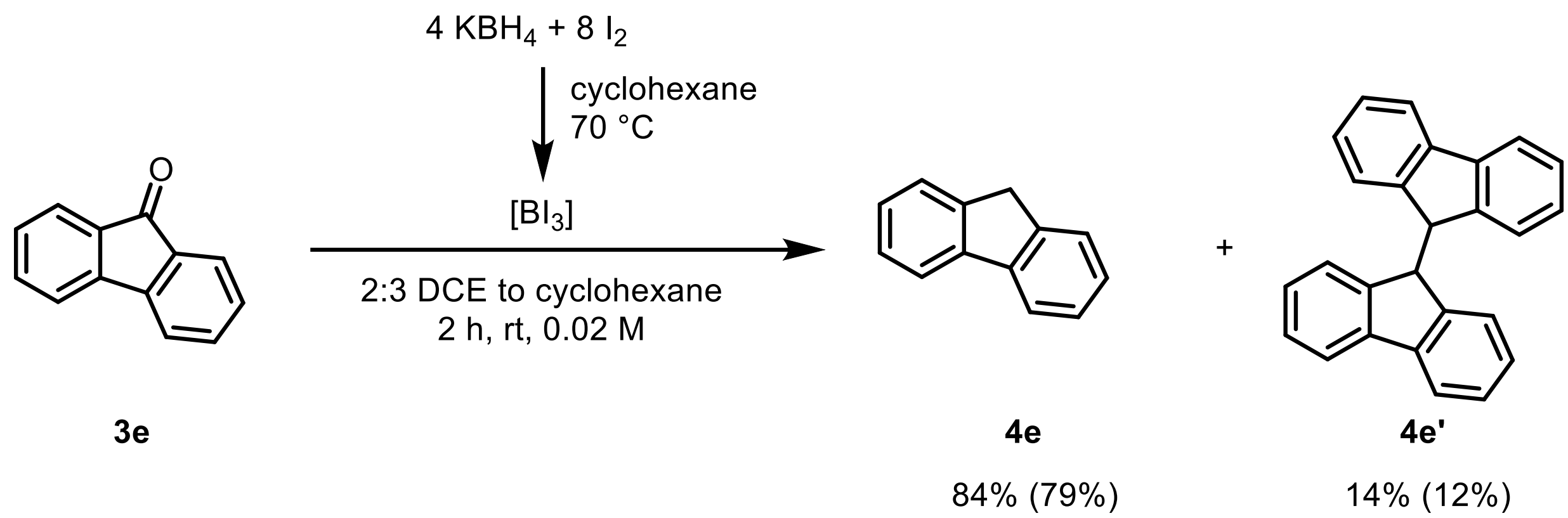

Scheme S3: Preparation of **4e'**.

The general procedure for ketone reduction was followed. Isolation was performed with chromatography (100% hexanes to 5% EtOAc in hexanes) to obtain dimerized product (8.1 mg 12% yield) as a yellow solid. ^{1}H-NMR ($CDCl_3$, 300 MHz): δ 7.65 (d, J = 7.6 Hz, 4H), 7.30-7.26 (m, (overlapping with $CHCl_3$), 4H), 7.09 (t, J = 7.5 Hz, 4H), 6.96 (d, J = 7.5 Hz, 4H), 4.85 (s, 2H) ppm. ^{13}C{^{1}H}-NMR ($CDCl_3$, 75 MHz): δ 144.8, 141.7, 127.4, 126.8, 124.2, 119.8, 49.9 ppm. Spectral data were consistent with literature values.[4]

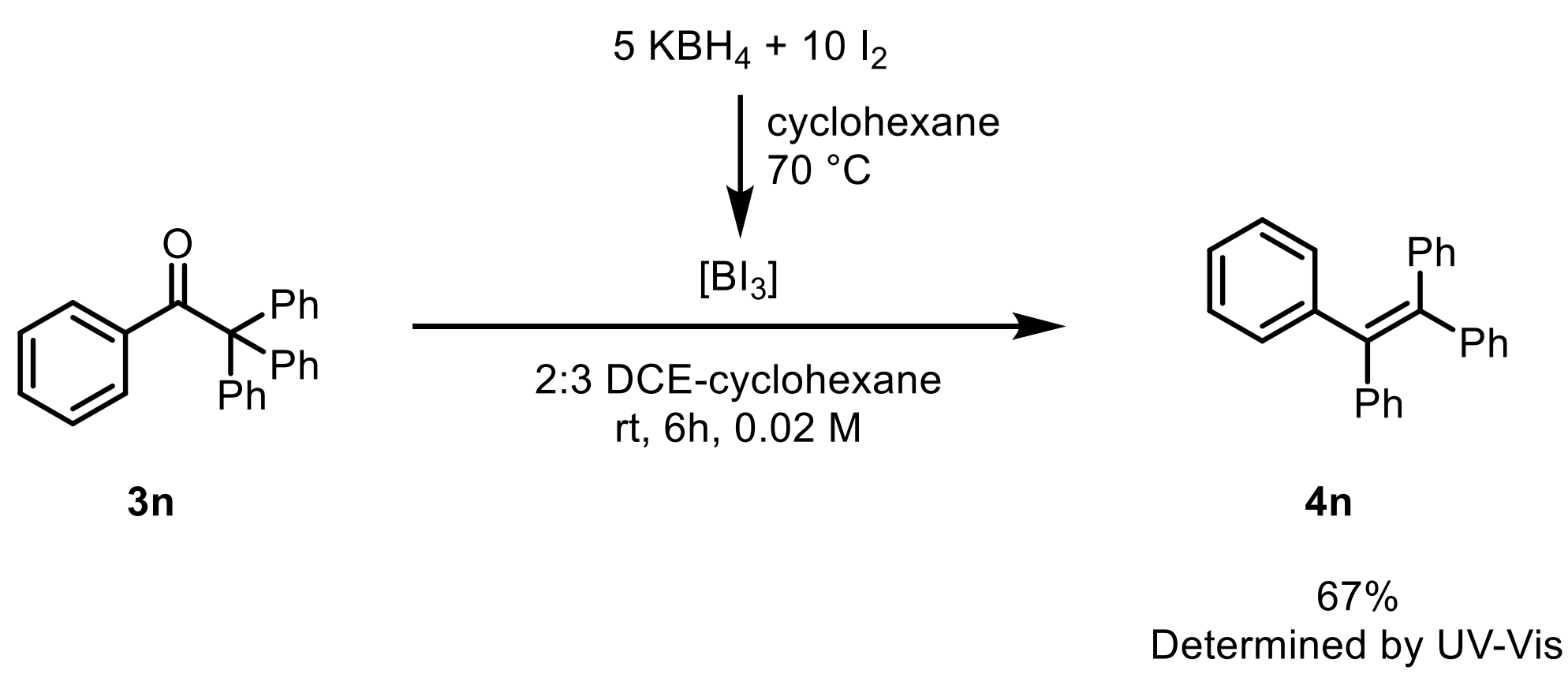

Scheme S4: Preparation of **4n.**

The general procedure was followed using 5.00 equiv. KBH_4 and 10.00 equiv. I_2 and reaction was run for 6 hours. The crude reaction mixture after workup was assessed by ^{1}H NMR, ^{13}C NMR, GC-HRMS, UV-Vis, and TLC in comparison to a commercial standard of **4n**, providing confidence of its formation in the sample as the main product. Separation of tetraphenylethylene from starting material proved challenging. A small amount (<10%) of tetraphenylethylene **4n** was partially purified (~90%) by column chromatography (100% hexane) to further confirm generation of the product (^{1}H NMR and ^{13}C NMR of this partially purified **4n** stacked over commercial tetraphenylethylene can be found in section 1.6). HRMS (EI): m/z calculated for $C_{26}H_{20}$: 332.1565, found: 332.1552.

For quantification of the tetraphenyl ethylene product in the crude reaction mixture, the reaction material was added to a 100 mL volumetric flask followed by EtOAc to generate a 100 mL solution. 0.5 mL was removed from the flask and diluted to 10 mL. This solution was then analyzed solution by UV-Vis spectroscopy and compared to UV-Vis intensity values for a known concentration of tetraphenylethylene **4m** (4.24×10^{-5} M).

Figure S1: Absorbance vs. Wavelength of **3n**.

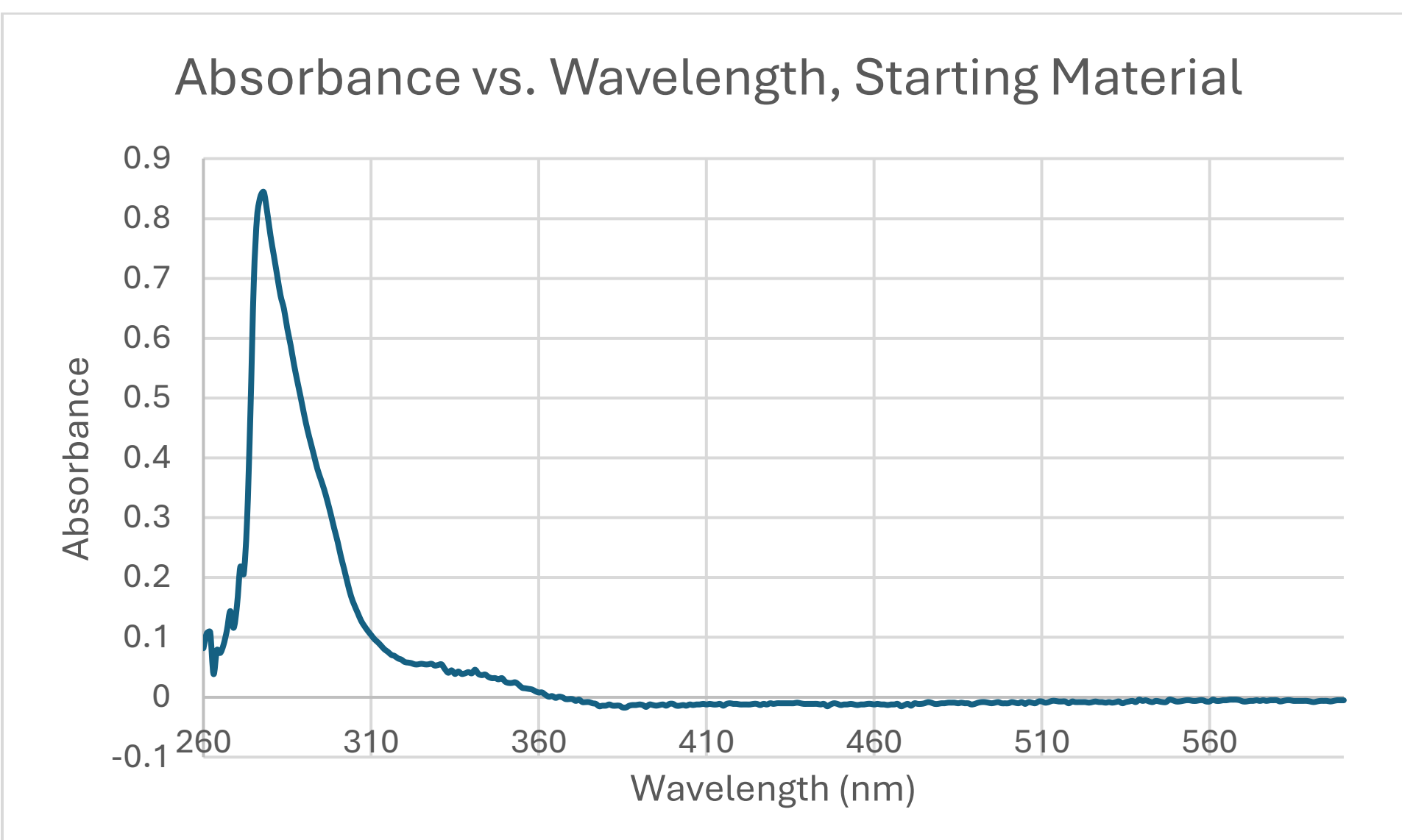

Figure S2: Absorbance vs. Wavelength of commercial tetraphenylethylene.

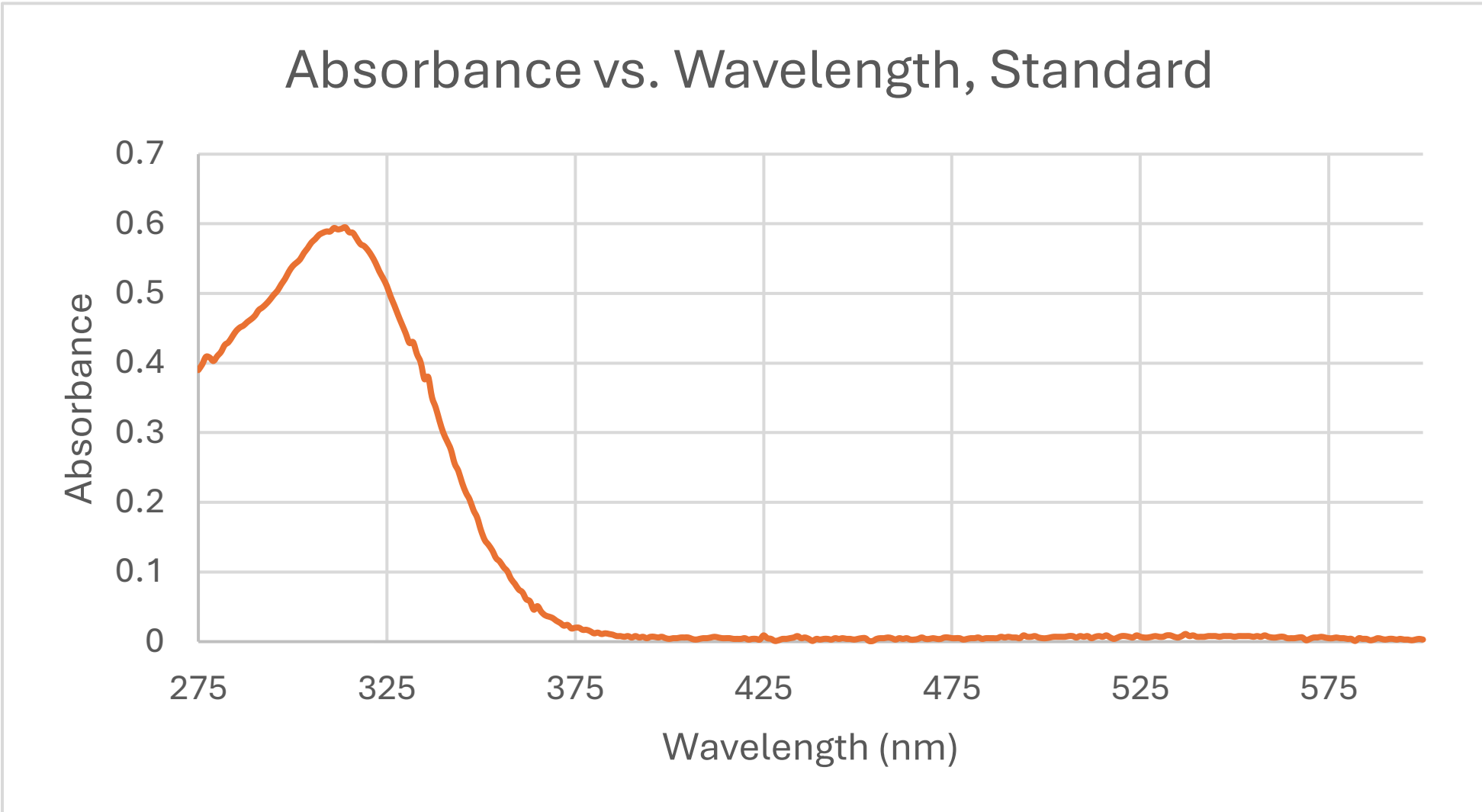

Figure S3: Absorbance vs. Wavelength of reaction aliquot.

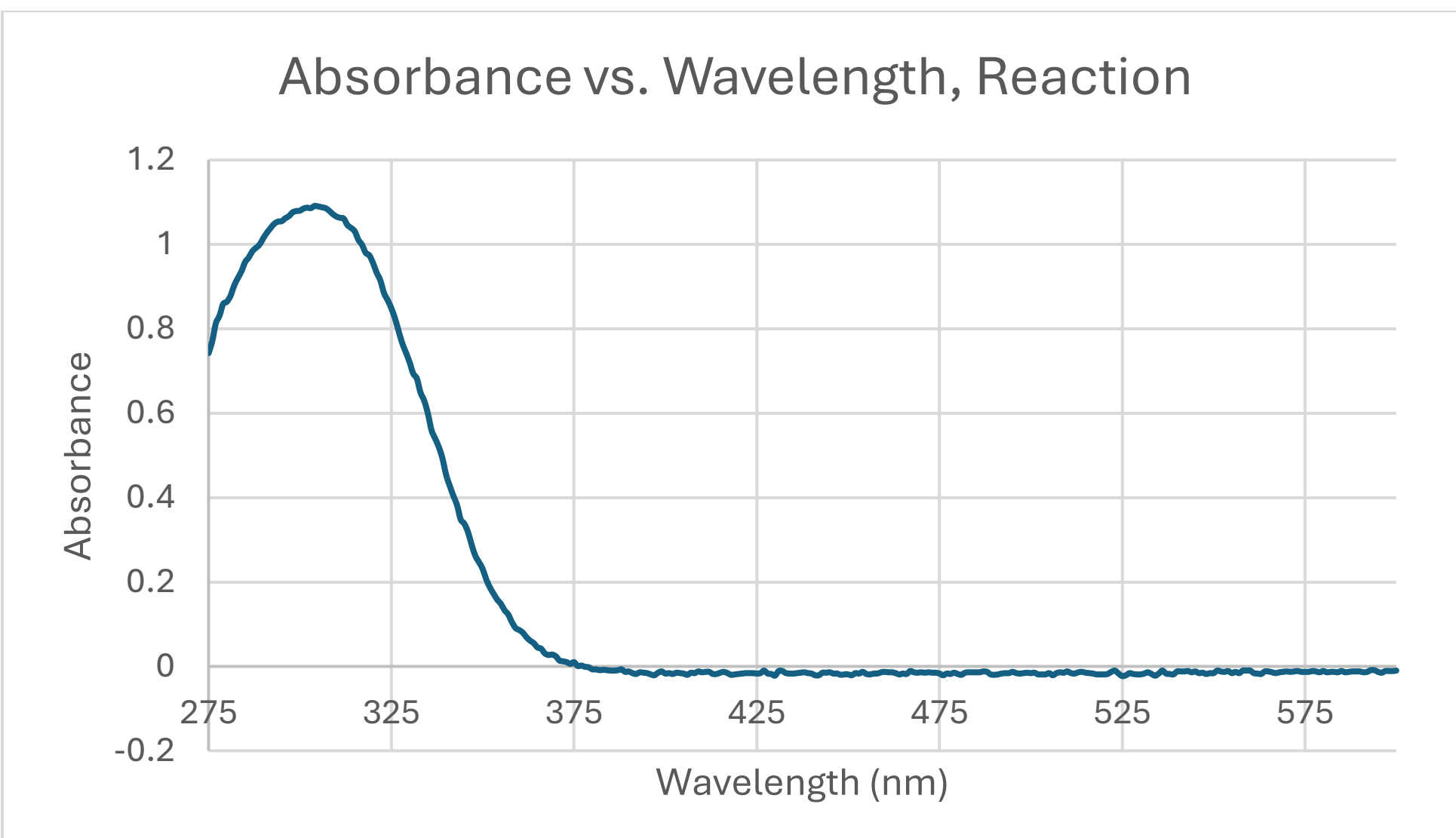

Figure S4: Sample calculations of percent yield using absorption values at $\lambda = 316$ and 331 nm.

A_1 = Absorbance of tetraphenylethylene at a selected wavelength

A_2 = Absorbance of reaction mixture at a selected wavelength

C_1 = Known concentration of tetraphenylethylene

C_2 = Concentration of tetraphenylethylene in reaction mixture

$$\frac{A_1}{A_2} = \frac{C_1}{C_2}$$

Average % yield using intensities at 316 nm (**68%**) and 331 nm (**66%**): **67%**

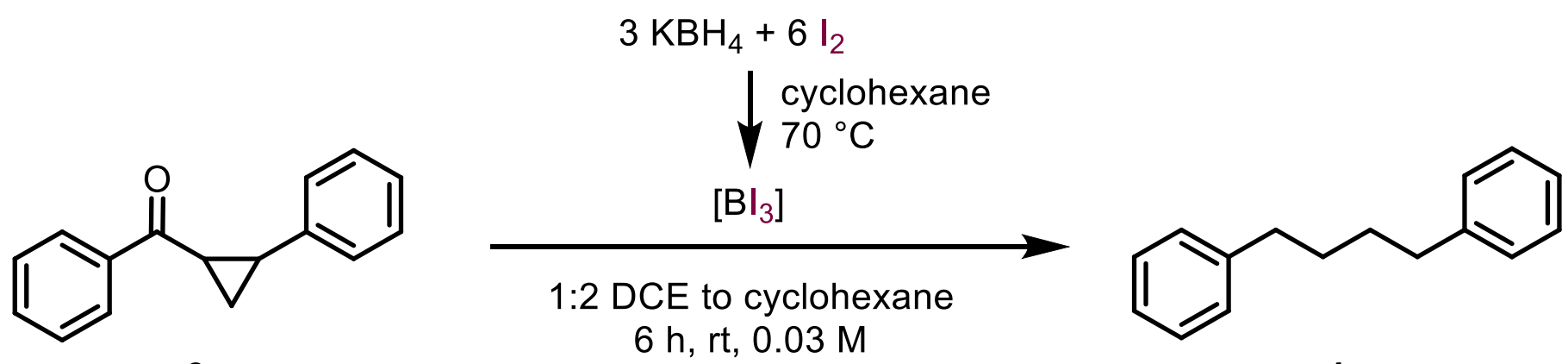

Scheme S5: Ring-opening experiment

KBH_4 (43.2 mg, 0.800 mmol, 3.00 equiv.), I_2 (406.1 mg, 1.600 mmol, 6.000 equiv.) and cyclohexane (4 mL) were added to a 25 mL single neck round bottom flask equipped with a stir bar. The round bottom flask was capped with a rubber septum cap, and the septum was tightened with a copper wire. The flask was placed in a 70 °C oil bath and allowed to stir until BI_3 generation was complete (about 30 minutes). Once completed, the reaction was allowed to cool to room temperature and subsequently punctured with a needle, a nitrogen line was added, and the reaction was diluted with an additional 2 mL cyclohexane. Ketone (0.27 mmol) was weighed out in a 20 mL scintillation vial and dissolved in DCE (1.5 mL). The resulting ketone solution was injected into the round bottom flask containing *in situ* generated BI_3, after which a color change to dark purple was almost immediately observed. The vial was rinsed with another 1.5 mL of DCE and injected into the reaction mixture.

After 2 hours, the reaction was cooled to 0 °C and quenched with a 5% $Na_2S_2O_3$ solution (10 mL). The contents were added to a separatory funnel, and the flask was washed with 10 mL EtOAc. The separatory funnel was shaken until all the iodine was consumed, as determined by loss of color, and the layers were separated. Aqueous phase was washed with EtOAc (2 x 10 mL). The organic fraction was then washed with brine (1 x 10mL), dried over Na_2SO_4 and concentrated in vacuo. Crude mixture was analyzed by GC-HRMS. HRMS (EI): m/z calculated for $C_{16}H_{18}$: 210.1409, found: 210.1402.

1.4. LFER Experiment

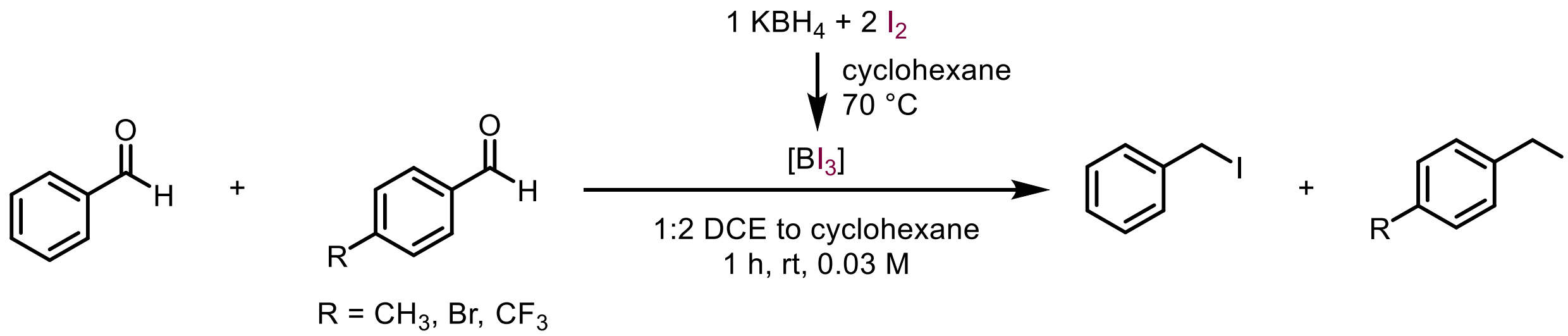

Scheme S6: LFER experiment

KBH_4 (21.6 mg, 0.400 mmol, 1.00 equiv.), I_2 (203.0 mg, 0.800 mmol, 2.000 equiv.) and cyclohexane (2 mL) were added to a 25 mL single neck round bottom flask equipped with a stir bar. The round bottom flask was capped with a rubber septum cap, and the septum was tightened with a copper wire. The flask was placed in a 70 °C oil bath and allowed to stir until BI_3 generation was complete. Once complete, the reaction was allowed to cool to room temperature and subsequently punctured with a needle, a nitrogen line was, and diluted with an additional 6 mL cyclohexane. Benzaldehyde (0.4 mmol) and the competing aldehyde (0.4 mmol) were weighed in separate scintillation vials. Added 1 mL DCE to each vial, then combined the solutions in a syringe, and injected the two aldehydes together into the reaction. Each vial was rinsed with another 1 mL of DCE and injected into the reaction mixture together. After 1 h, reaction was cooled to 0 °C and quenched with 10 mL isopropanol. The resulting mixture was filtered into a 100 mL volumetric flask, followed by the addition of decane (0.400 mmol) as an internal standard. The solution was diluted to 100 mL with EtOAc and submitted for GC analysis.

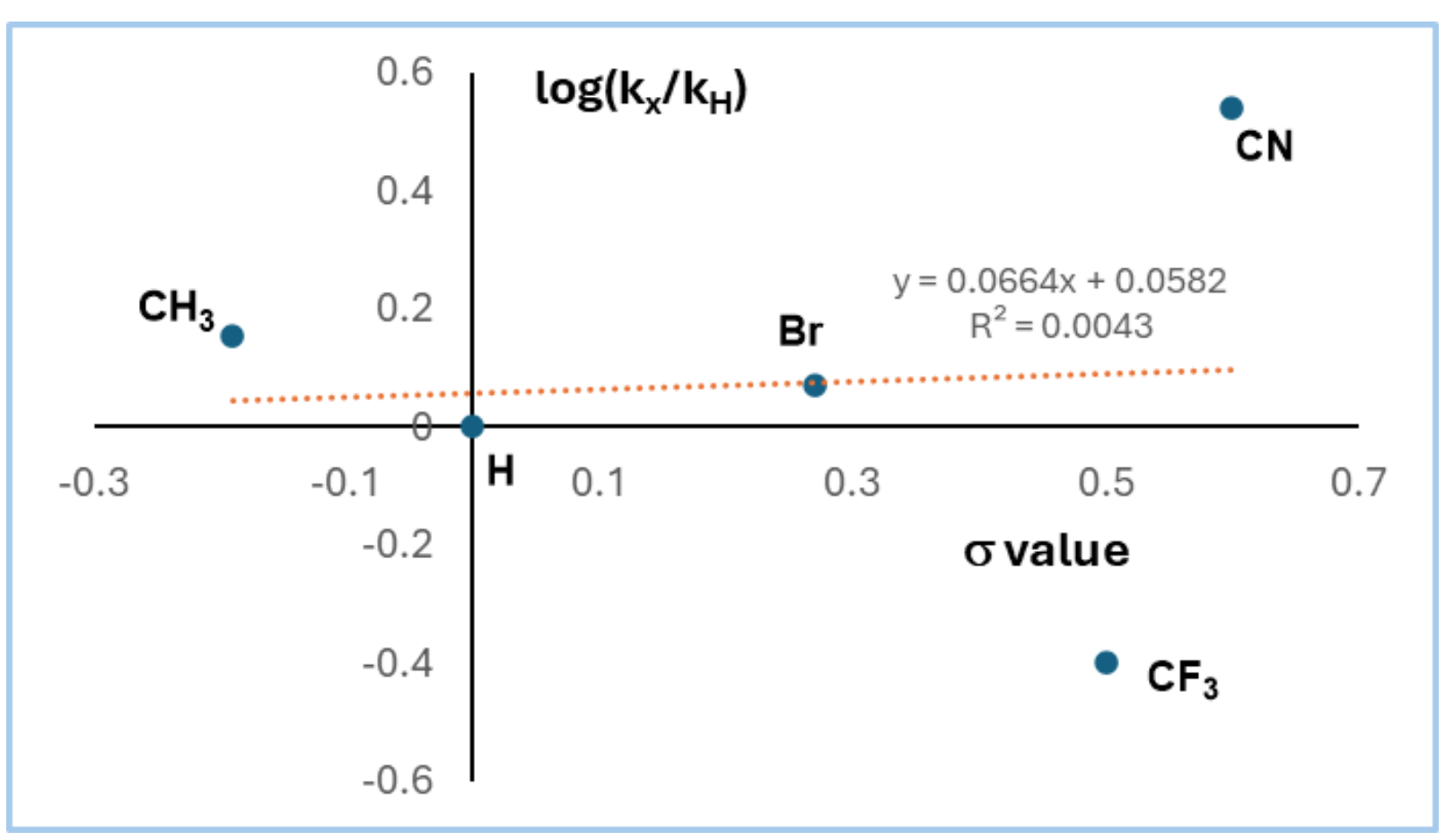

Figure S5. Linear Free Energy Relationship Plot using Hammett values showing poor linear fit.

1.5. Characterization data
Aldehydes:

4-bromobenzyl iodide (2a)

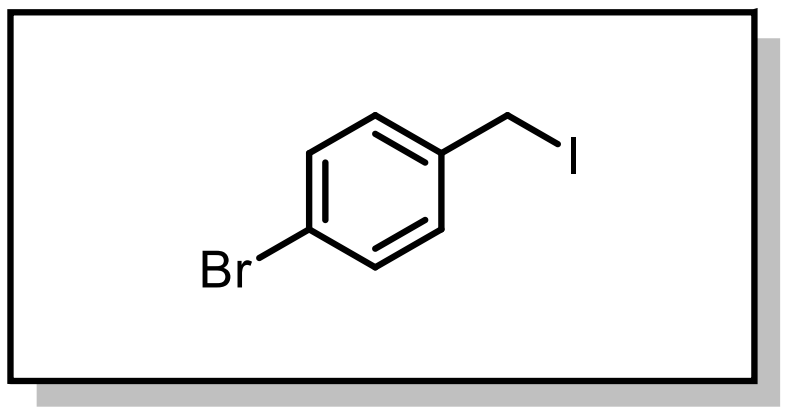

The general procedure was followed. Purified by column chromatography (1% ether in pentane). R_f = 0.59 in 1% ether in pentane. ^{1}H-NMR ($CDCl_3$, 300 MHz): δ 7.44 (d, J = 8.5 Hz, 2H), 7.27 (d, J = 8.3 Hz, 2H), 4.42 (s, 2H) ppm. ^{13}C{^{1}H}-NMR ($CDCl_3$, 100 MHz): δ 138.5, 132.1, 130.5, 121.8, 4.3 ppm. Spectral data were consistent with literature values.[5]

Appearance: White crystals

NMR yield: 97%

Isolated yield: 105.5 mg (89%)

2,4,6-trimethylbenzyl iodide (2b)

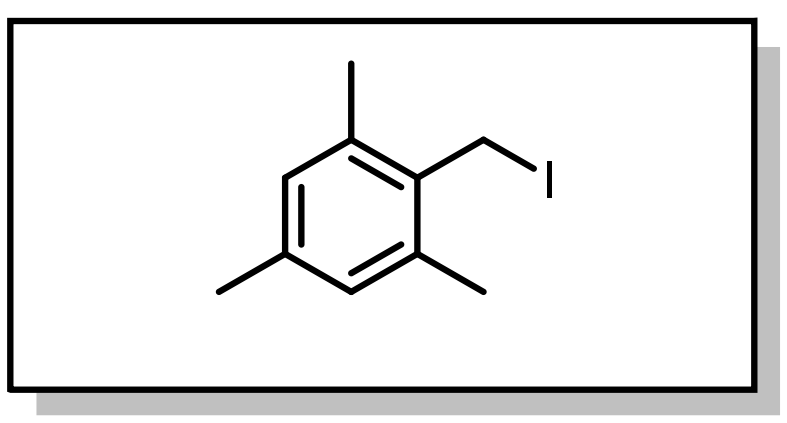

The general procedure was followed. Purified by column chromatography (1% ether in pentane). R_f = 0.55 in 100% hexane. ^{1}H-NMR ($CDCl_3$, 400 MHz): δ 6.83 (s, 2H), 4.46 (s, 2H), 2.32 (s, 6H), 2.25 (s, 3H) ppm. ^{13}C{^{1}H}-NMR ($CDCl_3$, 100 MHz): δ 138.1, 137.0, 132.0, 129.6, 21.2, 19.4, 4.1 ppm. Spectral data were consistent with literature values.[6]

Appearance: White crystals

NMR yield: 85%

Isolated yield: 82.0 mg (79%)

2-(iodomethyl)biphenyl (2c)

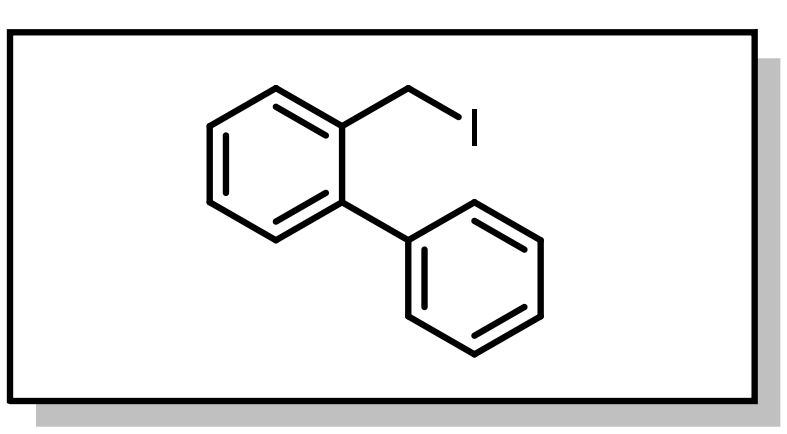

The general procedure was followed. Purified by column chromatography (1-5% EtOAc in hexanes). R_f = 0.60 in 1% EtOAc in hexanes. ^{1}H-NMR ($CDCl_3$, 400 MHz): δ 7.54-7.52 (m, 1H), 7.48-7.44 (m, 4H), 7.43-7.40 (m, 1H), 7.35-7.29 (m, 2H), 7.21-7.18 (m, 1H), 4.44 (s, 2H) ppm. ^{13}C{^{1}H}-NMR ($CDCl_3$, 100 MHz): δ 141.7, 140.6, 136.6, 130.8, 130.7, 128.8, 128.5, 128.2, 128.1, 127.5, 5.3 ppm. Spectral data were consistent with literature values.[7]

Appearance: Viscous, pale-yellow liquid. Solidifies in freezer.

NMR yield: 99%

Isolated yield: 107.4 mg (91%)

2-(iodomethyl)naphthalene (2d)

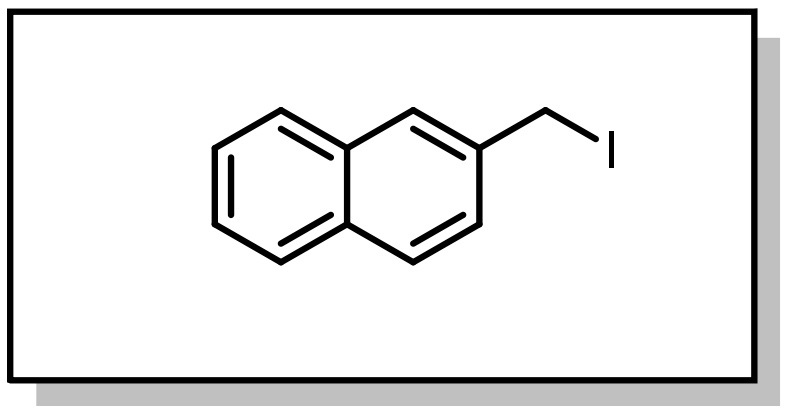

The reaction was quenched after 30 minutes instead of 1 hour to prevent methyl product from forming. Purified by column chromatography (1-5% EtOAc in hexanes). R_f = 0.32 in 1% EtOAc in hexanes. ^{1}H-NMR ($CDCl_3$, 400 MHz): δ 7.83-7.79 (m, 4H), 7.49-7.47 (m, 3H), 4.64 (s, 2H) ppm. ^{13}C{^{1}H}-NMR ($CDCl_3$, 100 MHz): δ 136.7, 133.4, 132.9, 128.9, 127.94, 127.87, 127.2, 127.1, 126.6. 126.5, 6.6 ppm. Spectral data were consistent with literature values.[8]

Appearance: Light yellow solid

NMR yield: 93%

Isolated yield: 93.6 mg (87%)

4-(iodomethyl)benzonitrile (2e)

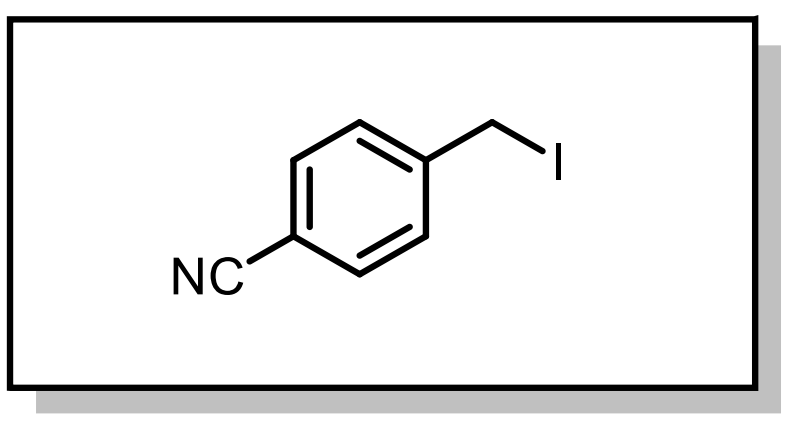

The reaction was run with 4 and 8 equivalences of KBH_4 and I_2 respectively. Purified by column chromatography (2% EtOAc in hexanes). R_f = 0.31 in 5% EtOAc in hexanes. ^{1}H-NMR ($CDCl_3$, 400 MHz): δ 7.59 (d, J = 8.3 Hz, 2H), 7.46 (d, J = 8.2 Hz, 2H), 4.44 (s, 2H) ppm. ^{13}C{^{1}H}-NMR ($CDCl_3$, 100 MHz): δ 144.8, 132.7, 129.6, 118.6, 111.7, 2.9 ppm. Spectral data were consistent with literature values.[9]

Appearance: White solid

NMR yield: 67%

Isolated yield: 62.8 mg (64%)

2,4-dichloro-1-(iodomethyl)benzene (2f)

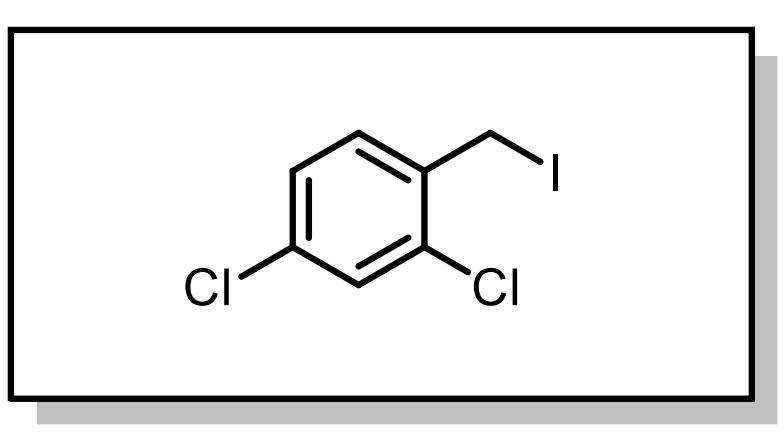

The reaction was run for 3 hours. Purified by column chromatography (100% heptane). ^{1}H-NMR ($CDCl_3$, 400 MHz): δ 7.36 (d, J = 2.1 Hz, 1H), 7.34 (d, J = 8.3 Hz, 1H), 7.19 (dd, J = 8.3, 2.1 Hz, 1H), 4.49 (s, 2H) ppm. ^{13}C{^{1}H}-NMR ($CDCl_3$, 100 MHz): δ 135.5, 134.573, 134.567, 131.4, 130.1, 127.8, 1.2 ppm. Spectral data were consistent with literature values.[10]

Appearance: White crystals

NMR yield: 92%

Isolated yield: 94.4 mg (82%)

2-bromo-5-(trifluoromethyl)benzyl iodide (2g)

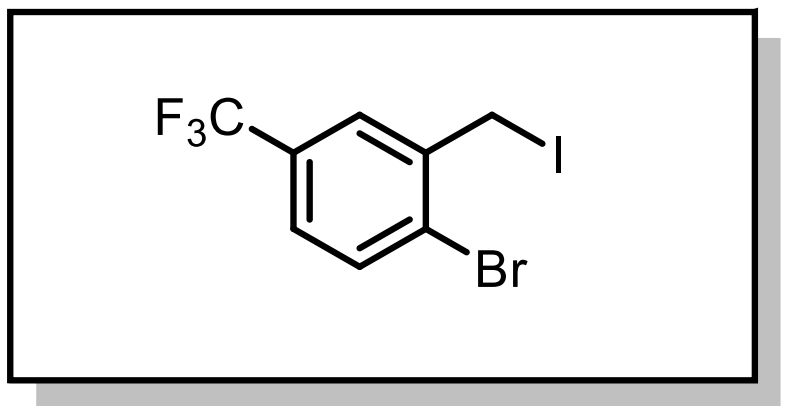

The reaction was run for 16 hours. Purified by column chromatography (1% ether in pentane). R_f = 0.74 in 1% ether in pentane. ^{1}H-NMR ($CDCl_3$, 400 MHz): δ 7.68-7.66 (m (overlapping s and d), 2H), 7.37 (dd, *J* = 8.3, 2.0 Hz, 1H), 4.55 (s, 2H) ppm. $^{13}C\{^1H\}$-NMR ($CDCl_3$, 100 MHz): δ 139.7, 134.3, 130.7 (q, *J* = 32.9 Hz), 128.0 (q, *J* = 1.4 Hz), 127.3 (q, *J* = 3.7 Hz), 126.2 (q, *J* = 3.6 Hz), 123.6 (q, *J* = 270.7 Hz), 3.9 ppm. ^{19}F-NMR ($CDCl_3$, 377 MHz): δ -62.8 (s, 3F) ppm. IR (neat, cm^{-1}): ν = 1600, 1326, 1197, 1145, 1108, 1071, 896, 823. HRMS (EI): m/z calculated for $C_8H_5F_3Br^+$ [M-I]: 236.9521, found: 236.9522.

Appearance: White crystals

Melting point: 50-51 °C

NMR yield: 84%

Isolated yield: 107.0 mg (73%)

4-(iodomethyl)-N,N-diethylbenzamide (2h)

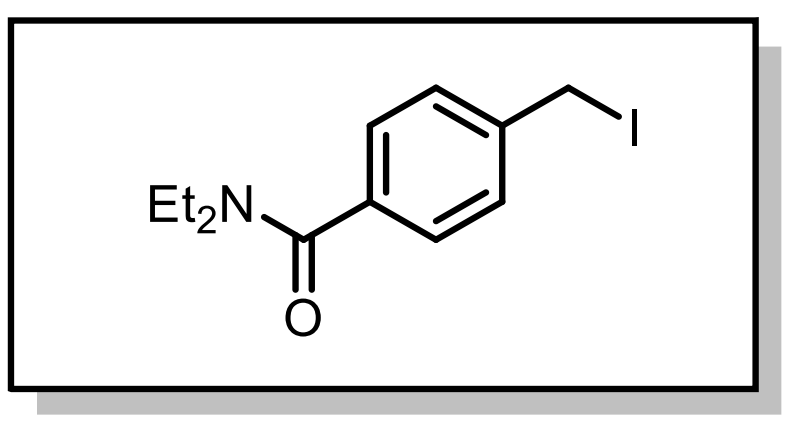

Using 4 equivalences of pure BI_3 with 3 equivalences of water produced better results than *in situ* BI_3 generation. Purified by column chromatography (30% EtOAc in hexanes). R_f = 0.15 in 30% EtOAc in hexanes. ^{1}H-NMR ($CDCl_3$, 600 MHz): δ 7.39 (d, *J* = 8.1 Hz, 2H), 7.29 (d, *J* = 8.1 Hz, 2H), 4.44 (s, 2H), 3.53 (bs, 2H), 3.25 (bs, 2H), 1.23 (bs, 3H), 1.11 (bs, 3H) ppm. $^{13}C\{^1H\}$-NMR ($CDCl_3$, 150 MHz): δ 170.8, 140.4, 136.8, 128.9, 127.0, 43.4, 39.3, 14.3, 13.0, 4.7 ppm. IR (neat, cm^{-1}): ν = 2961, 2927, 1616, 1565, 1466, 1421, 1086, 803, 579. HRMS (EI): m/z calculated for $C_{12}H_{16}NO^+$ [M-I]: 190.1226, found: 190.1229.

Appearance: White solid

Melting point: 82-84 °C

NMR yield: 68%

Isolated yield: 39.6 mg (62%)

(4-(iodomethyl)phenyl)(methyl)sulfane (2i)

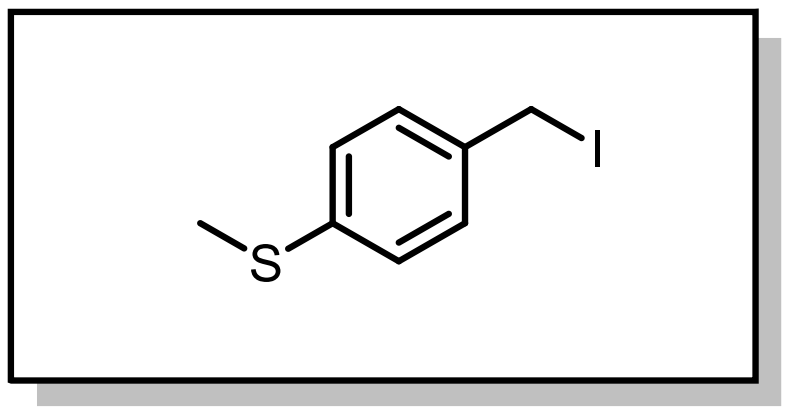

The reaction was run with 3 and 6 equivalences of KBH_4 and I_2 respectively. Purified by column chromatography (1% EtOAc in hexanes). R_f = 0.28 in 1% EtOAc in hexanes. ^{1}H-NMR ($CDCl_3$, 400 MHz): δ 7.30 (d, J = 8.4 Hz, 2H), 7.16 (d, J = 8.4 Hz, 2H), 4.45 (s, 2H), 2.47 (s, 3H) ppm. ^{13}C{^{1}H}-NMR ($CDCl_3$, 100 MHz): δ 138.6, 136.0, 129.3, 126.8, 15.7, 5.9 ppm. Spectral data were consistent with literature values.[11]

Appearance: White solid

NMR yield: 87%

Isolated yield: 79.2 mg (75%)

4-(trifluoromethyl)benzyl iodide (2j)

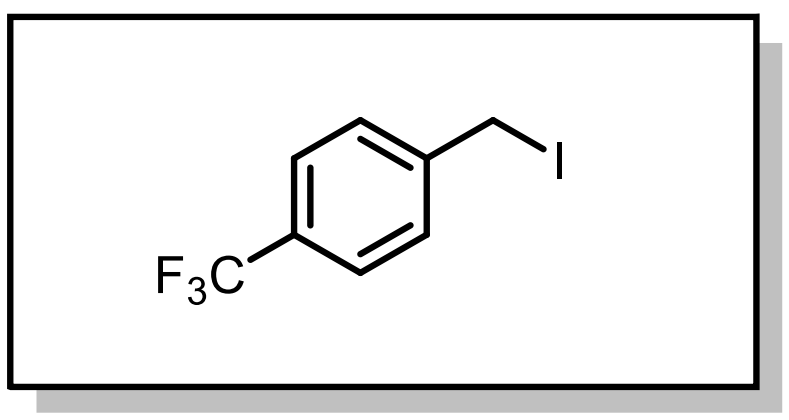

The general procedure was followed. Purified by column chromatography (1% ether in pentane). R_f = 0.69 in 1% ether in pentane. ^{1}H-NMR ($CDCl_3$, 400 MHz): δ 7.56 (d, J = 8.2 Hz, 2H), 7.48 (d, J = 8.2 Hz, 2H), 4.45 (s, 2H) ppm. ^{13}C{^{1}H}-NMR ($CDCl_3$, 100 MHz): δ 143.5 (q, J = 1.3 Hz), 130.1 (q, J = 32.4 Hz), 129.2, 126.0 (q, J = 3.8 Hz), 124.1 (q, J = 270.3 Hz), 3.4 ppm. ^{19}F-NMR ($CDCl_3$, 377 MHz): δ -62.7 (s, 3F) ppm. Spectral data were consistent with literature values.[12]

Appearance: White crystals

NMR yield: 76%

Isolated yield: 82.1 mg (72%)

Quinolin-2-ylmethanol (2n)

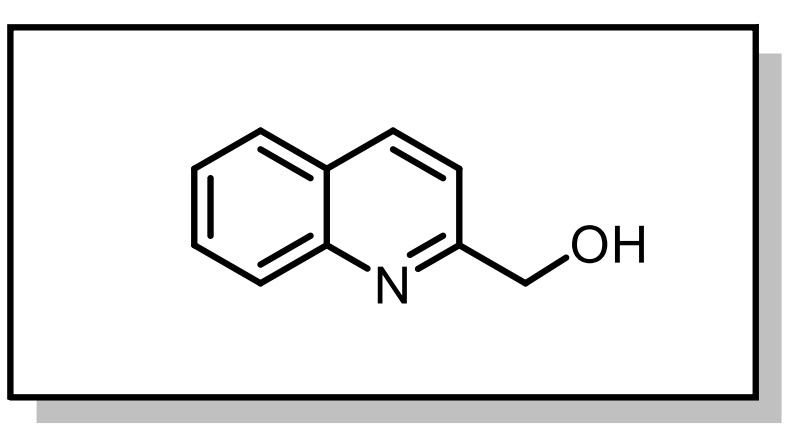

The reaction was run with 4 and 8 equivalences of KBH_4 and I_2 respectively. Purified by column chromatography (25% EtOAc in hexanes, followed by an acetone flush). ^{1}H-NMR ($CDCl_3$, 300 MHz): δ 8.12 (d, J = 8.5 Hz, 1H), 8.07 (d, J = 8.4 Hz, 1H), 7.81 (dd, J = 8.1, 1.1 Hz, 1H), 7.72 (ddd, J = 8.4, 7.0, 1.5 Hz, 1H), 7.56-7.51 (m, 1H), 7.29 (d, J = 8.5 Hz, 1H), 4.92 (s, 2H), 4.15 (bs, 1H) ppm. ^{13}C{^{1}H}-NMR ($CDCl_3$, 75 MHz): δ 159.2, 146.8, 137.0, 129.9, 128.7, 127.8, 127.7, 126.5, 118.5, 64.3 ppm. Spectral data were consistent with literature values.[13]

Appearance: Yellow solid

NMR yield: 55%

Isolated yield: 28.7 mg (45%)

Ketones:

Diphenylmethane (4a)

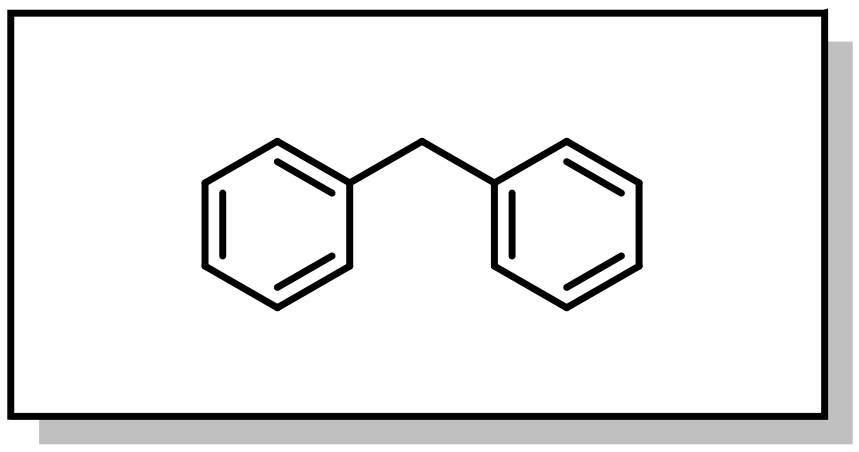

The general procedure was followed. Purified by column chromatography (1% ether in pentane). R_f= ^{1}H-NMR ($CDCl_3$, 400 MHz): δ 7.34-7.30 (m, 4H), 7.24-7.21 (m, 6H), 4.02 (s, 2H) ppm. ^{13}C{^{1}H}-NMR ($CDCl_3$, 100 MHz): δ 141.2, 129.1, 128.6, 126.2, 42.1 ppm. Spectral data were consistent with literature values.[14]

Appearance: Clear liquid or white crystals (melting point is very close to room temperature).

NMR yield: 95%

Isolated yield: 61.6 mg (91%)

2-benzyl-1-bromobenzene (4b)

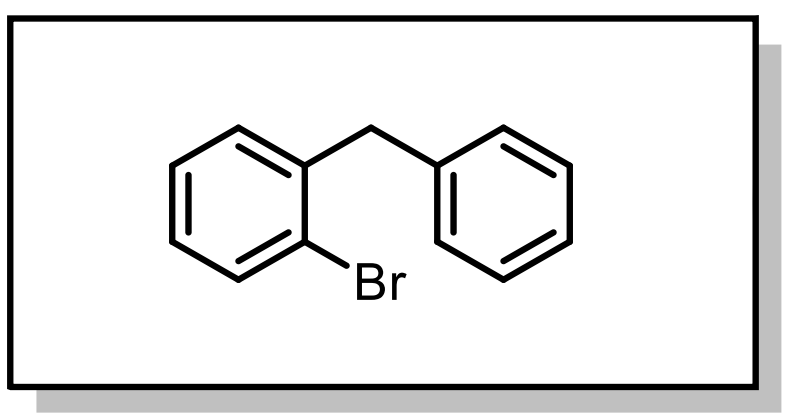

The general procedure was followed. Purified by column chromatography (1% ether in pentane). R_f = 0.43 in 1% ether in pentane. ^{1}H-NMR ($CDCl_3$, 400 MHz): δ 7.57 (dd, J = 7.9, 1.2 Hz, 1H), 7.32-7.28 (m, 2H), 7.25-7.19 (m, 4H), 7.14 (dd, J = 7.7, 1.6 Hz, 1H), 7.08 (td, J = 7.6, 1.8 Hz, 1H), 4.13 (s, 2H) ppm. ^{13}C{^{1}H}-NMR ($CDCl_3$, 100 MHz): δ 140.5, 139.6, 133.0, 131.2, 129.1, 128.6, 128.0, 127.6, 126.4, 125.0, 41.9 ppm. Spectral data were consistent with literature values.[15]

Appearance: Clear liquid

NMR yield: 91%

Isolated yield: 88.8 mg (90%)

1-benzyl-2-(trifluoromethyl)benzene (4c)

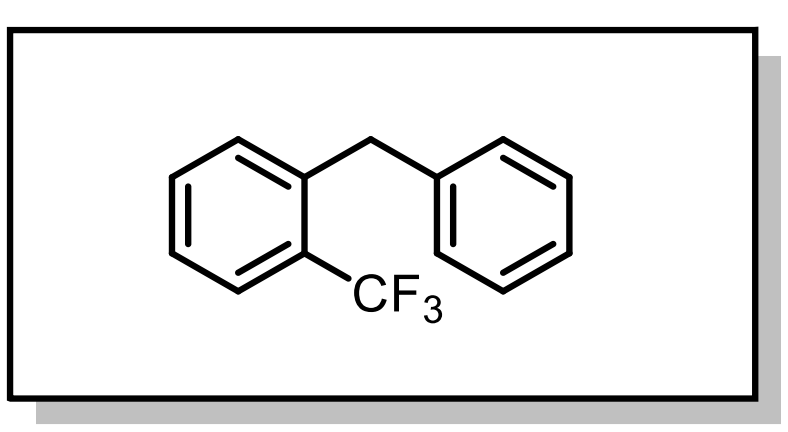

The general procedure was followed.. Purified by column chromatography (1% EtOAc in hexanes). R_f = 0.44 in 1% EtOAc in hexanes. ^{1}H-NMR ($CDCl_3$, 400 MHz): δ 7.69 (d, J = 7.8 Hz, 1H), 7.44 (t, J = 7.5 Hz, 1H), 7.34-7.30 (m, 3H), 7.26-7.23 (m, 1H), 7.20-7.17 (m, 3H), 4.21 ppm. ^{13}C{^{1}H}-NMR ($CDCl_3$, 100 MHz): δ 140.0, 139.7, 131.9, 129.3, 128.9 (q, J = 29.6 Hz), 128.7, 126.5, 126.4, 126.0 (q, J = 5.7 Hz), 124.7 (q, J = 272.3 Hz), 37.9 ppm. ^{19}F-NMR ($CDCl_3$, 377 MHz): δ -59.6 (s, 3F) ppm. Spectral data were consistent with literature values.[16]

Appearance: Clear liquid

NMR yield: 95%

Isolated yield: 80.2 mg (85%)

1-Benzyl-2,4-dimethylbenzene (4d)

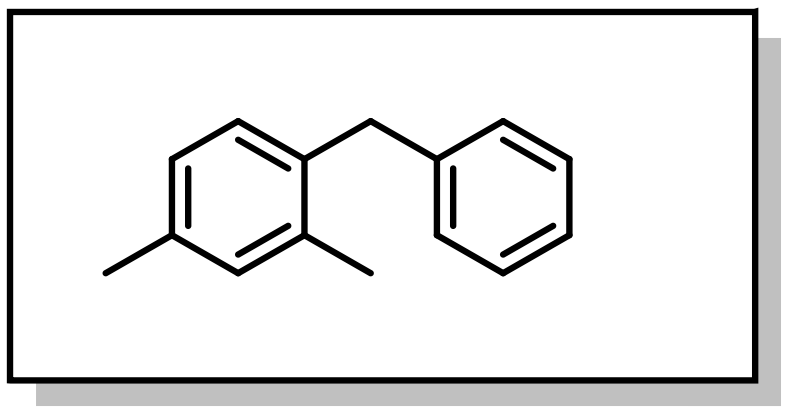

The general procedure was followed. Purified by column chromatography (1% ether in pentane). R_f = 0.33 in 1% ether in pentane. ^{1}H-NMR ($CDCl_3$, 300 MHz): δ 7.37-7.32 (m, 2H), 7.29-7.20 (m, 3H), 7.10-7.04 (m, 3H), 4.04 (s, 2H), 2.39 (s, 3H), 2.29 (s, 3H) ppm. $^{13}C\{^1H\}$-NMR ($CDCl_3$, 75 MHz): δ 140.8, 136.5, 136.0, 131.2, 130.0, 128.80, 128.79 128.5, 126.7, 125.9, 39.2, 21.0, 19.7 ppm. Spectral data were consistent with literature values.[17]

Appearance: Clear liquid

NMR yield: 93%

Isolated yield: 68.8 mg (88%)

Fluorene (4e)

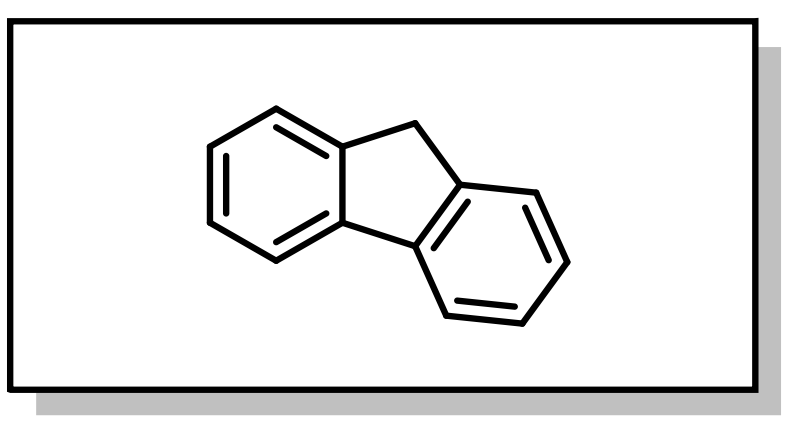

The general procedure was followed. Purified by column chromatography (100% hexanes). R_f = 0.35 in 100% hexanes. ^{1}H-NMR ($CDCl_3$, 400 MHz): δ 7.80 (d, J = 7.5 Hz, 2H), 7.56 (d, J = 7.4 Hz, 2H), 7.39 (t, J = 7.4 Hz, 2H), 7.31 (td, J = 7.4, 1.2 Hz, 2H), 3.91 (s, 2H) ppm. $^{13}C\{^1H\}$-NMR ($CDCl_3$, 100 MHz): δ 143.3, 141.8, 126.9, 126.8, 125.2, 120.0, 37.1 ppm. Spectral data were consistent with literature values.[18]

Appearance: White solid

NMR yield: 86%

Isolated yield: 53.3 mg (79%)

4-benzylbiphenyl (4f)

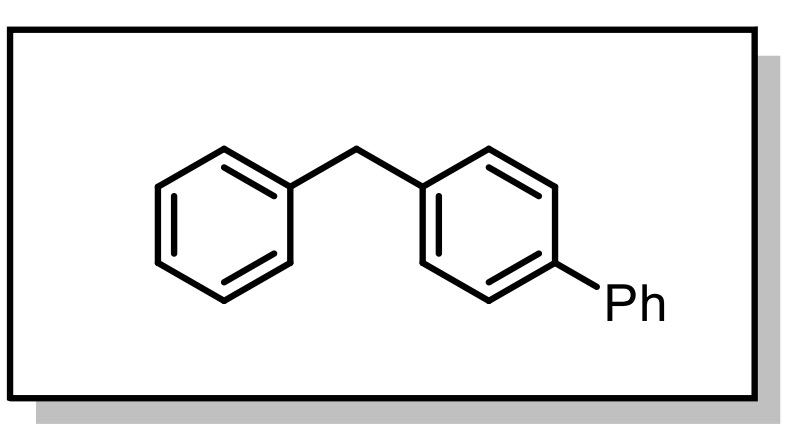

The general procedure was followed. Purified by column chromatography (1-5% EtOAc in hexanes). R_f = 0.12 in 1% EtOAc in hexanes. ^{1}H-NMR ($CDCl_3$, 300 MHz): δ 7.57-7.53 (m, 2H), 7.50 (d, J = 8.3 Hz, 2H), 7.39 (t, J = 7.7 Hz, 2H), 7.32-7.16 (m, 8H), 4.00 (s, 2H) ppm. $^{13}C\{^1H\}$-NMR ($CDCl_3$, 75 MHz): δ 141.11, 141.12, 140.4, 139.2, 129.4, 129.1, 128.8, 128.6, 127.3, 127.2, 127.1, 126.3, 41.7 ppm. Spectral data were consistent with literature values.[19]

Appearance: White solid

NMR yield: 92%

Isolated yield: 85.0 mg (87%)

o-Benzylphenol (4g)

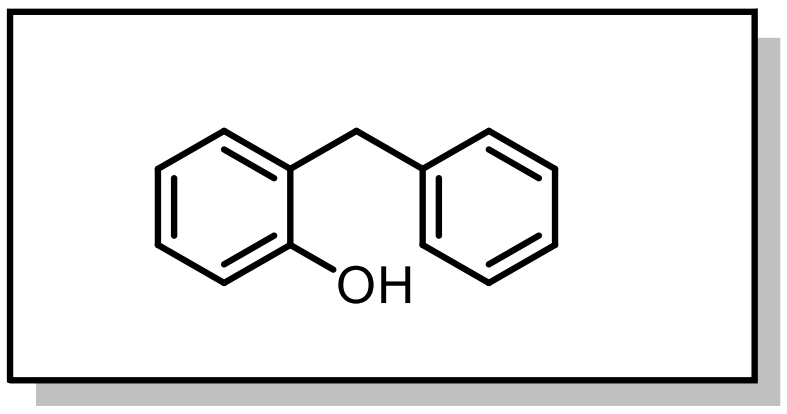

The reaction was run at 70 °C. Purified by column chromatography (5-10% EtOAc in hexanes). R_f = 0.27 in 10% EtOAc in hexanes. ^{1}H-NMR ($CDCl_3$, 400 MHz): δ 7.33-7.22 (m, 5H), 7.16-7.12 (m, 2H), 6.91 (t, J = 7.4 Hz, 1H), 6.78 (d, J = 7.3 Hz, 1H), 4.71 (s, 1H), 4.01 (s, 2H) ppm. ^{13}C{^{1}H}-NMR ($CDCl_3$, 100 MHz): δ 153.8, 140.0, 131.1, 128.81, 128.76, 128.0, 127.1, 126.5, 121.1, 115.9, 36.5 ppm. Spectral data were consistent with literature values.[20]

Appearance: Clear liquid

NMR yield: 53%

Isolated yield: 34.9 mg (47%)

1-ethylnaphthalene (4h)

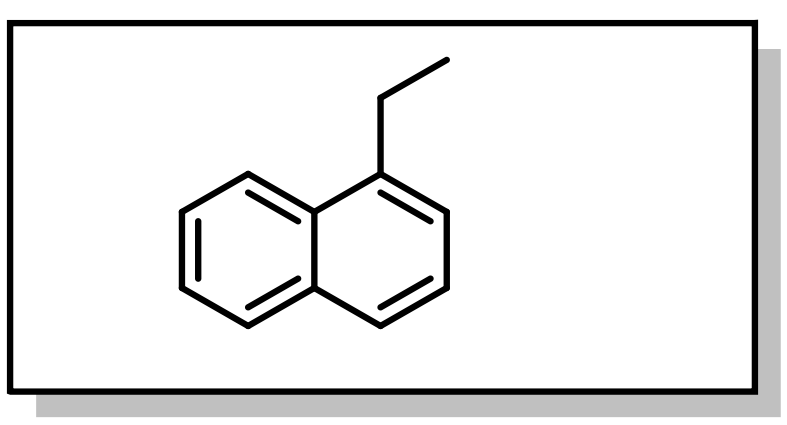

The reaction was run for 6 h with 5 and 10 equivalences of KBH_4 and I_2 respectively. Purified by column chromatography (100% pentane). R_f = 0.64 in 100% pentane. ^{1}H-NMR ($CDCl_3$, 300 MHz): δ 8.12-8.09 (d, J = 7.8 Hz, 1H), 7.91-7.88 (m, 1H), 7.75 (d, J = 8.1 Hz, 1H), 7.56-7.37 (m, 4H), 3.16 (q, J = 7.5 Hz, 2H), 1.44 (t, J = 7.5 Hz, 3H) ppm. ^{13}C{^{1}H}-NMR ($CDCl_3$, 75 MHz): δ 140.4, 134.0, 131.9, 128.9, 126.5, 125.8, 125.5, 125.0, 123.9, 26.0, 15.2 ppm. Spectral data were consistent with literature values.[21]

Appearance: Clear liquid

NMR yield: 93%

Isolated yield: 23.0 mg (74%)

4-bromopropylbenzene (4i)

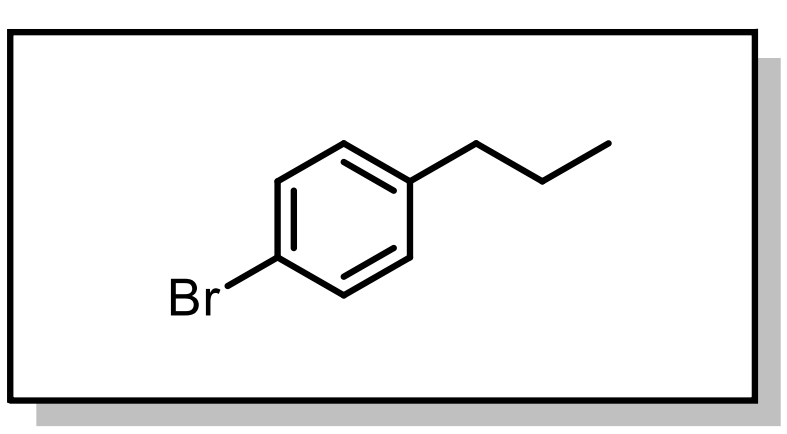

The reaction was run for 6 h with 5 and 10 equivalences of KBH_4 and I_2 respectively. Purified by column chromatography (100% pentane). R_f = 0.76 in 100% pentane. ^{1}H-NMR ($CDCl_3$, 400 MHz): δ 7.39 (d, J = 8.4 Hz, 2H), 7.05 (d, J = 8.4 Hz, 2H), 2.54, (t, J = 7.8 Hz, 2H), 1.62 (sextet, J = 7.6 Hz, 2H), 0.93 (t, J = 7.4 Hz, 3H) ppm. ^{13}C{^{1}H}-NMR ($CDCl_3$, 100 MHz): δ

141.7, 131.4, 130.4, 119.4, 37.5, 24.5, 13.8 ppm. Spectral data were consistent with literature values.[22]

Appearance: Clear liquid

NMR yield: 75%

Isolated yield: 17.5 mg (44%)

4-ethylbiphenyl (4j)

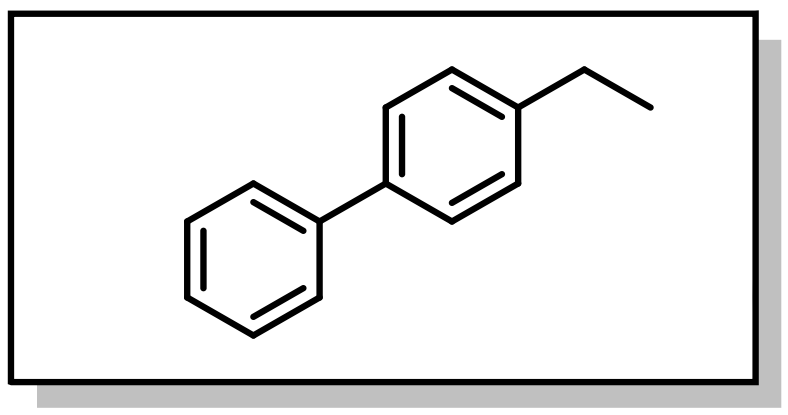

The reaction was run for 6 h with 5 and 10 equivalences of KBH_4 and I_2 respectively. Purified by column chromatography (100% hexanes). R_f = 0.46 in 100% hexanes. ^{1}H-NMR ($CDCl_3$, 300 MHz): δ 7.62-7.59 (m, 2H), 7.55 (d, *J* = 8.2 Hz, 2H), 7.47-7.42 (m, 2H), 7.37-7.33 (m, 1H), 7.30 (d, *J* = 8.3 Hz, 2H), 2.72 (q, *J* = 7.6 Hz, 2H), 1.31 (t, *J* = 7.6 Hz, 3H) ppm. ^{13}C{^{1}H}-NMR ($CDCl_3$, 75 MHz): δ 143.5, 141.3, 138.8, 128.8, 128.4, 127.2, 127.15, 127.10, 28.7, 15.7 ppm. Spectral data were consistent with literature values.[23]

Appearance: White solid

NMR yield: 78%

Isolated yield: 17.3 mg (61%)

4-isobutylphenyl methyl sulfide (4k)

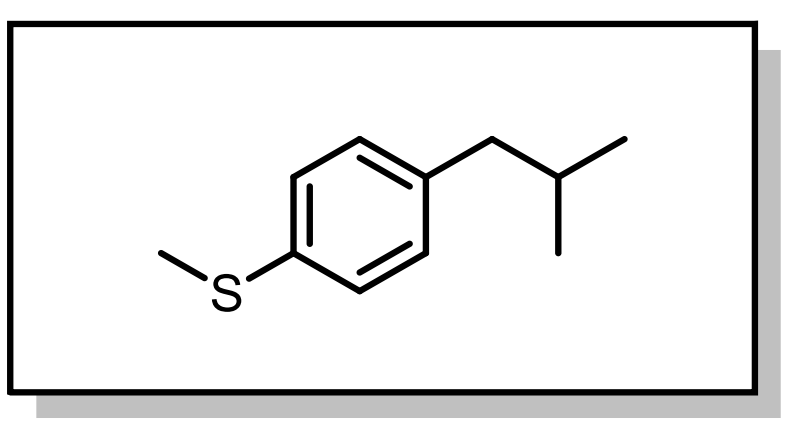

The reaction was run for 6 h with 5 and 10 equivalences of KBH_4 and I_2 respectively. Purified by column chromatography (100% pentane). R_f = 0.47 in 100% pentane. ^{1}H-NMR ($CDCl_3$, 400 MHz): δ 7.20 (d, *J* = 8.3 Hz, 2H), 7.07 (d, *J* = 8.3 Hz, 2H), 2.47 (s, 3H), 2.44 (d, *J* = 7.2 Hz, 2H), 1.84 (septet, *J* = 6.7 Hz, 1H), 0.90 (d, *J* = 6.6 Hz) ppm. ^{13}C{^{1}H}-NMR ($CDCl_3$, 100 MHz): δ 138.9, 135.1, 129.7, 127.0, 45.0, 30.3, 22.4, 16.4 ppm. IR (neat, cm^{-1}): ν = 2954, 2919, 2868, 1493, 1462, 1090, 835, 787. HRMS (EI): m/z calculated for $C_{11}H_{16}S$: 180.0973, found: 180.0973.

Appearance: Clear liquid

NMR yield: 45%

Isolated yield: 30.5 mg (42%)

1-phenyl-2,2,2-trifluoromethylethanol (5l)

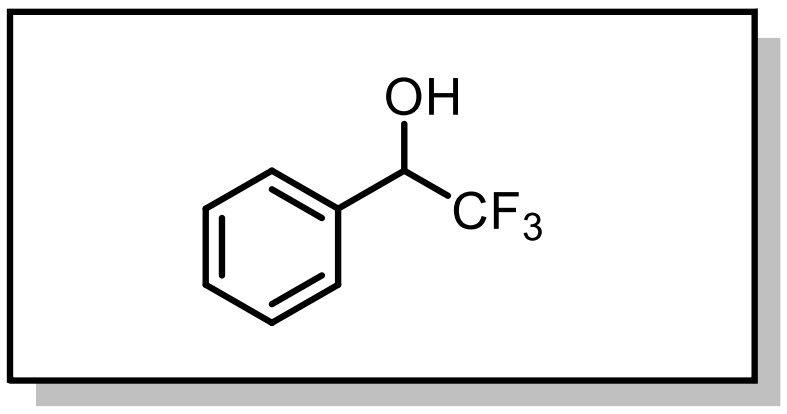

The reaction was run for 6 h. Purified by column chromatography (10% ether in pentane, R_f = 0.13). ^{1}H-NMR ($CDCl_3$, 300 MHz): δ 7.50-7.41 (m, 5H), 5.03 (q, *J* = 6.7 Hz, 1H), 2.57 (bs, 1H) ppm. ^{13}C{^{1}H}-NMR ($CDCl_3$, 75 MHz): δ 134.0 (q, *J* = 1.2 Hz), 129.6, 128.7, 127.5 (q, *J* = 1.0 Hz), 124.3 (q, *J* = 280.3 Hz), 72.9 (q, *J* = 31.7 Hz) ppm. ^{19}F-NMR ($CDCl_3$, 282 MHz): δ -78.3 (d, *J* = 6.7 Hz, 3F) ppm. Spectral data were consistent with literature values.[24]

Appearance: Clear liquid

NMR yield: 79%

Isolated yield: 41.0 mg (58%)

1-phenyl-1-(pyrid-3-yl)methanol (5m)

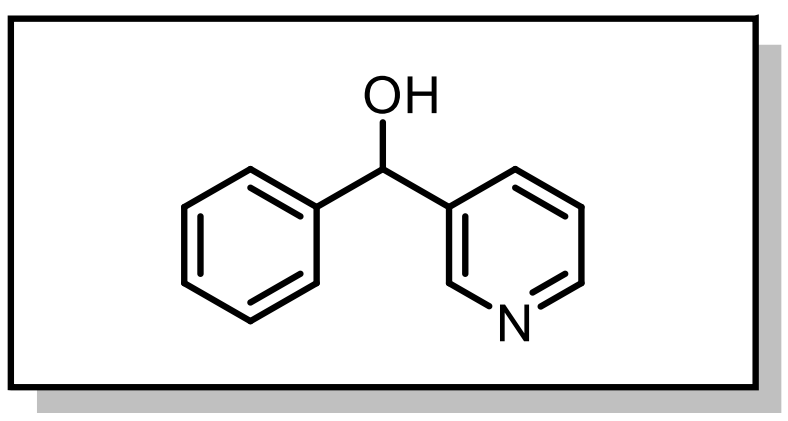

The general procedure was followed, but quenched with a sodium ascorbate solution (10%, 10 mL). Purified by column chromatography (20% hexanes in EtOAc). R_f = 0.27 in 20% hexanes in EtOAc. ^{1}H-NMR ($CDCl_3$, 400 MHz): δ 8.53 (s, 1H), 8.40 (s, 1H), 7.69 (d, *J* = 7.9 Hz, 1H), 7.37-7.27 (m, 5H), 7.23 (dd, *J* = 7.8, 4.8 Hz, 1H), 5.84 (s, 1H), 3.25 (bs, 1H) ppm. ^{13}C{^{1}H}-NMR ($CDCl_3$, 100 MHz): δ 148.5, 148.1, 143.3, 139.7, 134.5, 128.8, 128.0, 126.7, 123.6, 74.0 ppm. Spectral data were consistent with literature values.[25]

Appearance: White solid

NMR yield: 49%

Isolated yield: 17.3 mg (47%)

1.6. Spectral data

Figure S6: ^{1}H-NMR (300 MHz) of 2a in $CDCl_3$

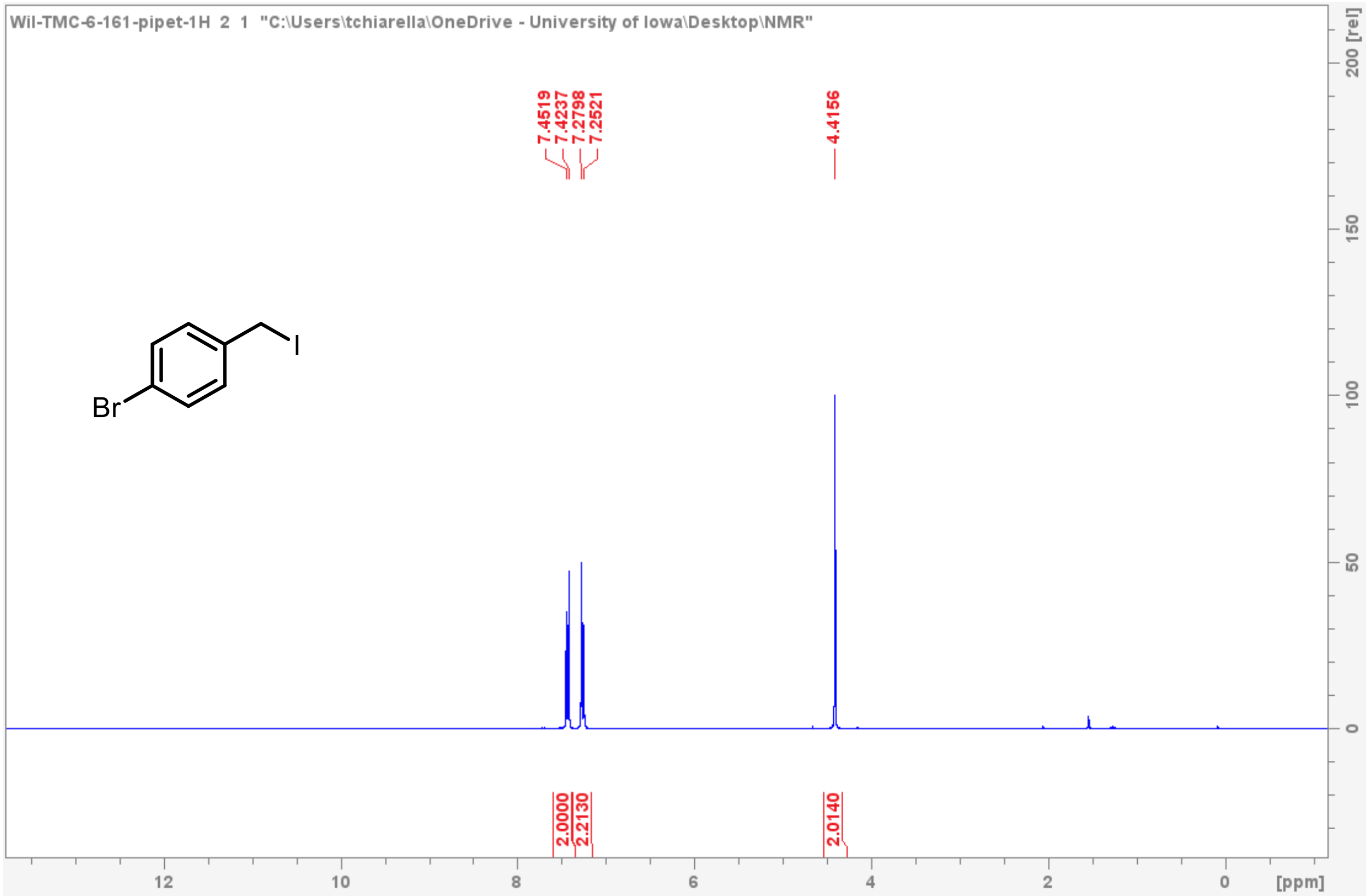

Figure S7: $^{13}C\{^1H\}$-NMR (100 MHz) of 2a in $CDCl_3$ (100 MHz)

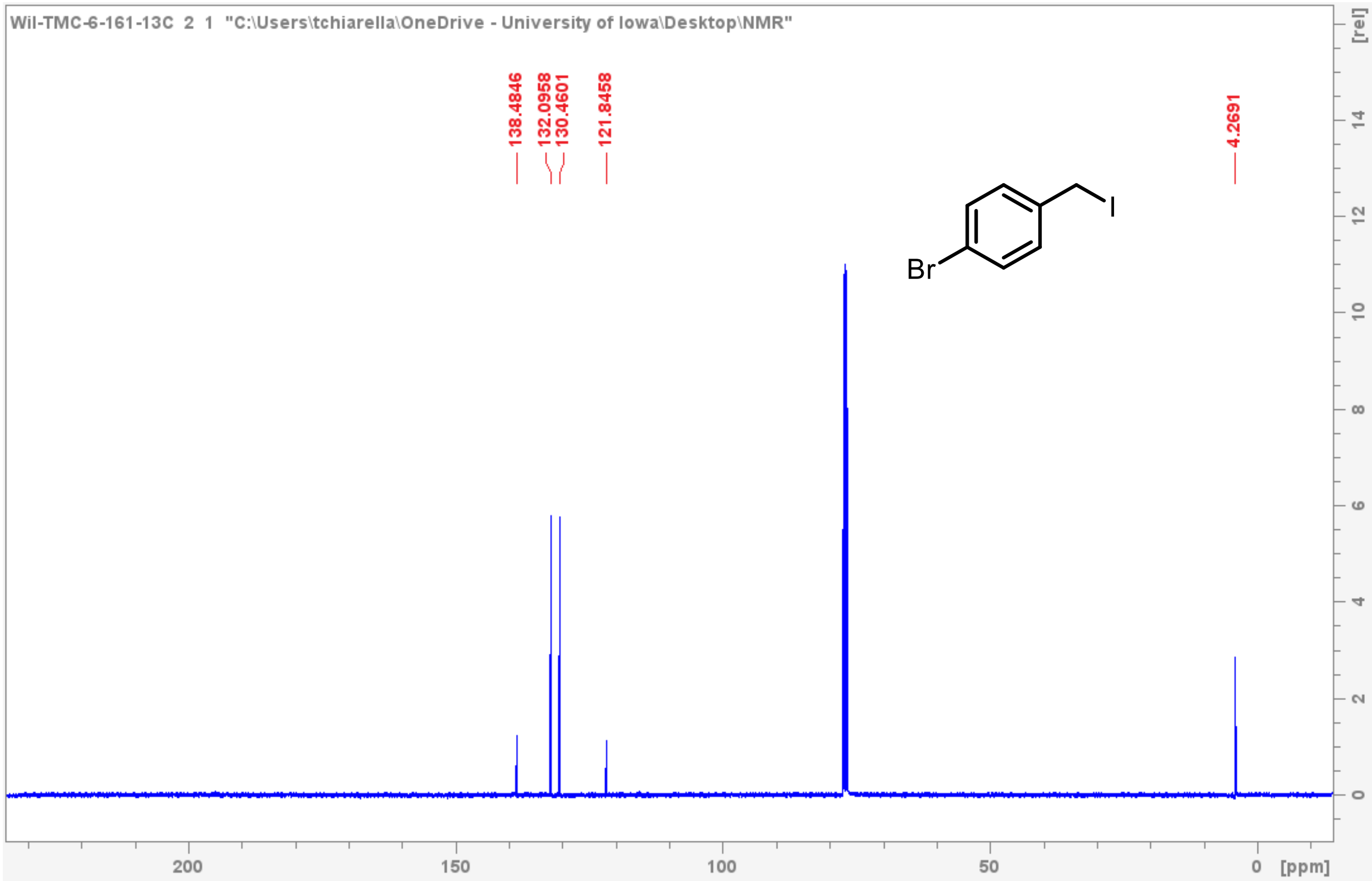

Figure S8: ^1H-NMR (400 MHz) of 2b in $CDCl_3$

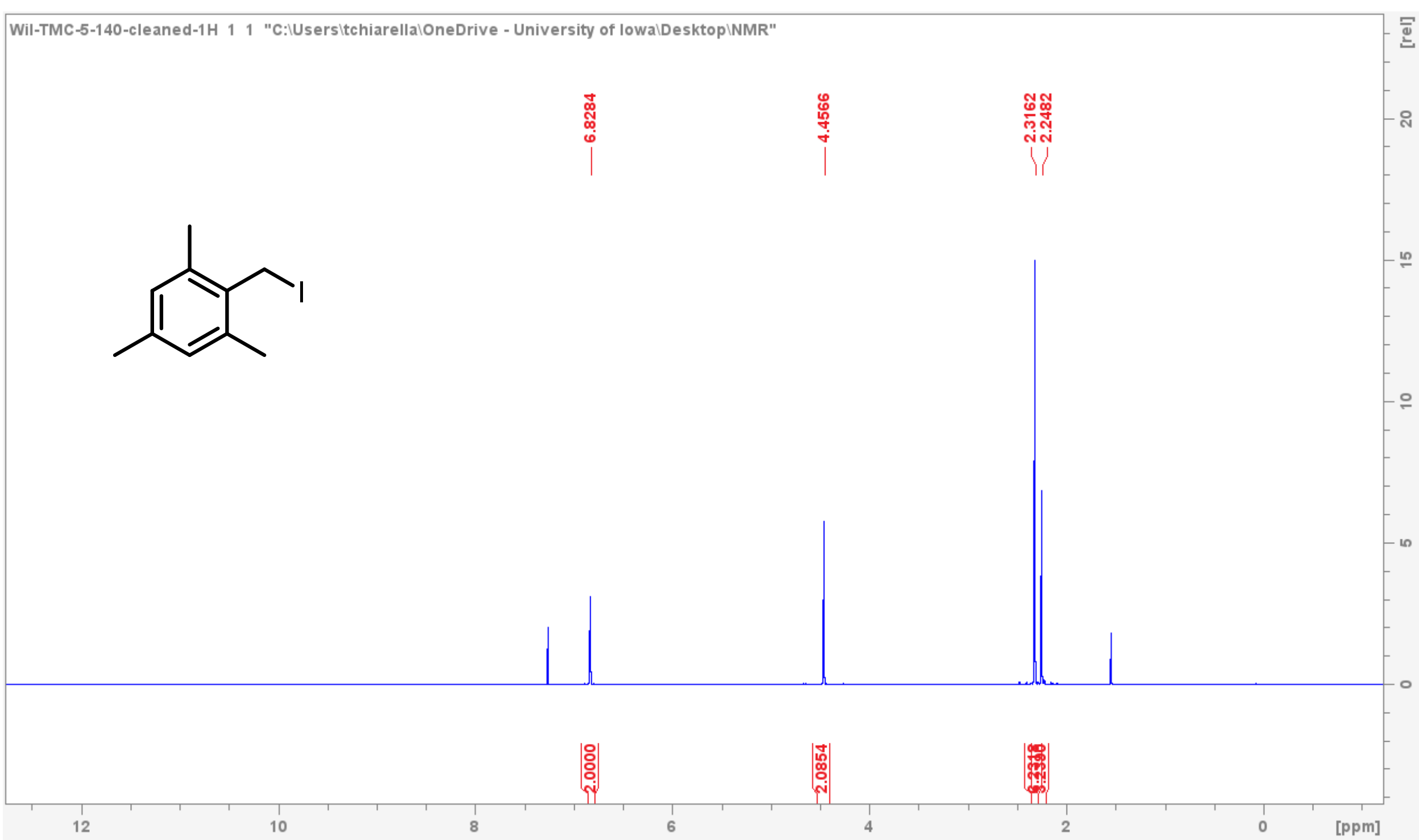

Figure S9: Zoomed in ^{1}H-NMR (400 MHz) of 2b in $CDCl_3$

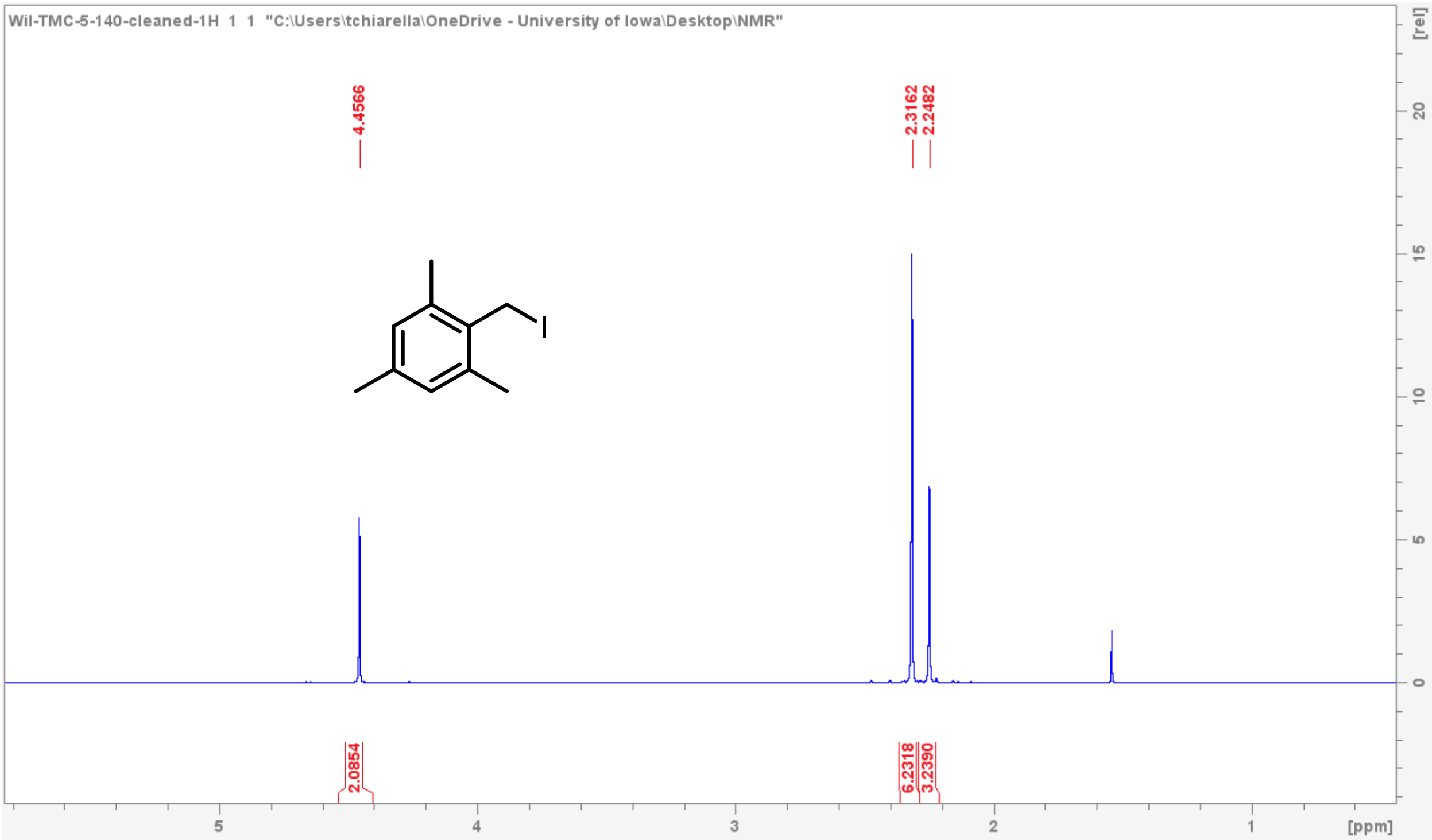

Figure S10: $^{13}C\{^{1}H\}$-NMR (100 MHz) of 2b in $CDCl_3$

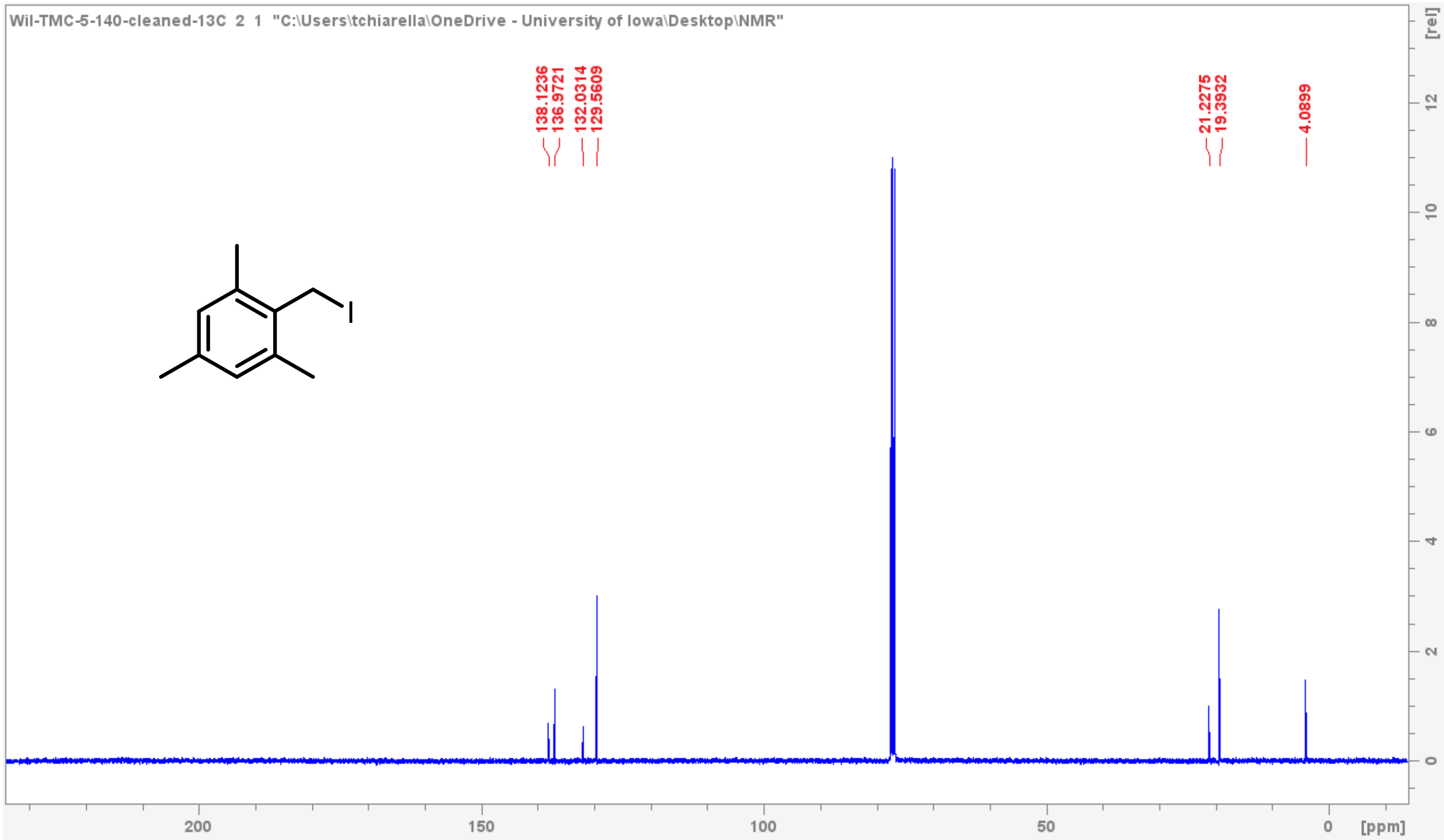

Figure S11: ^{1}H-NMR (400 MHz) of 2c in $CDCl_3$

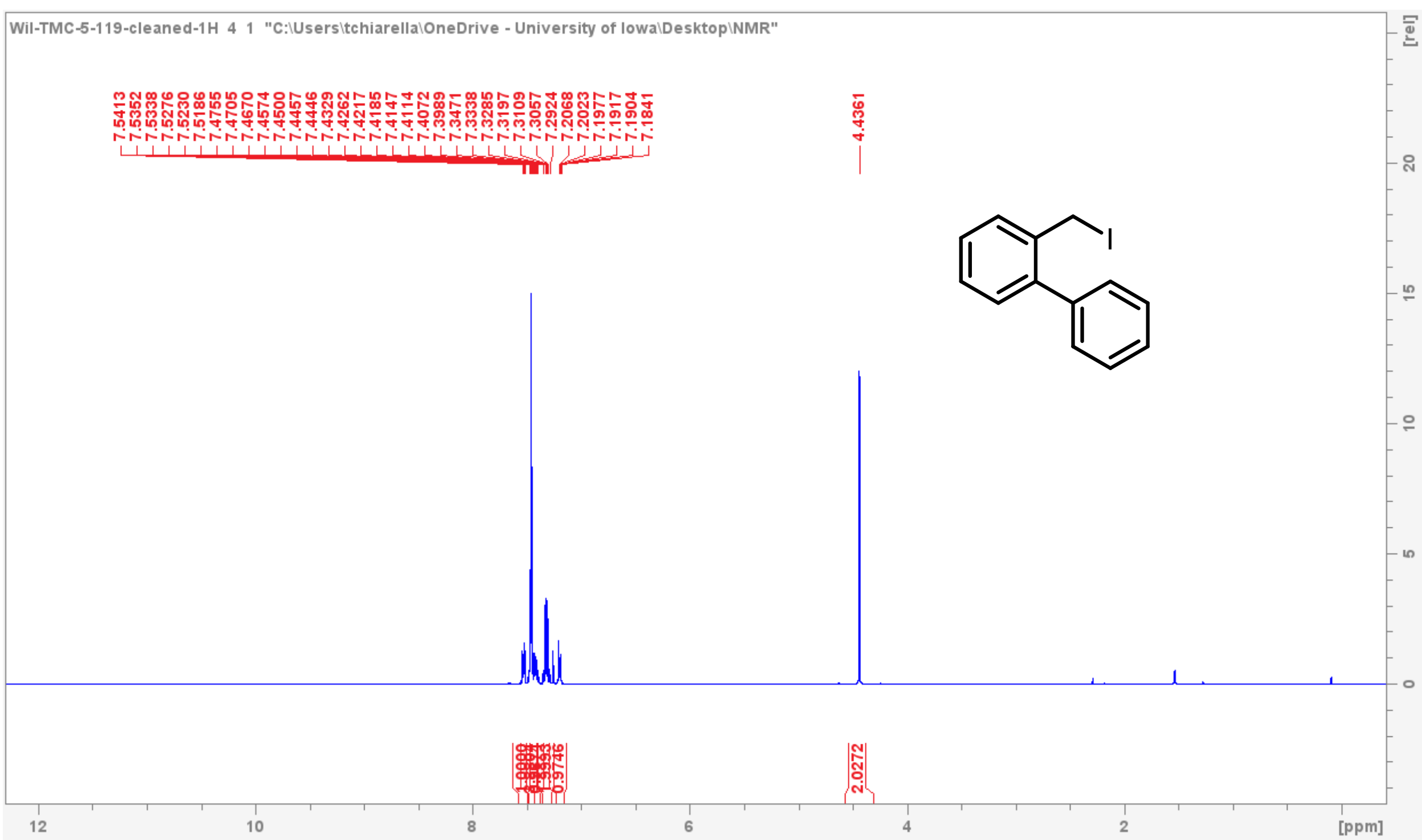

Figure S12: Zoomed in ^{1}H-NMR (400 MHz) of 2c in $CDCl_3$

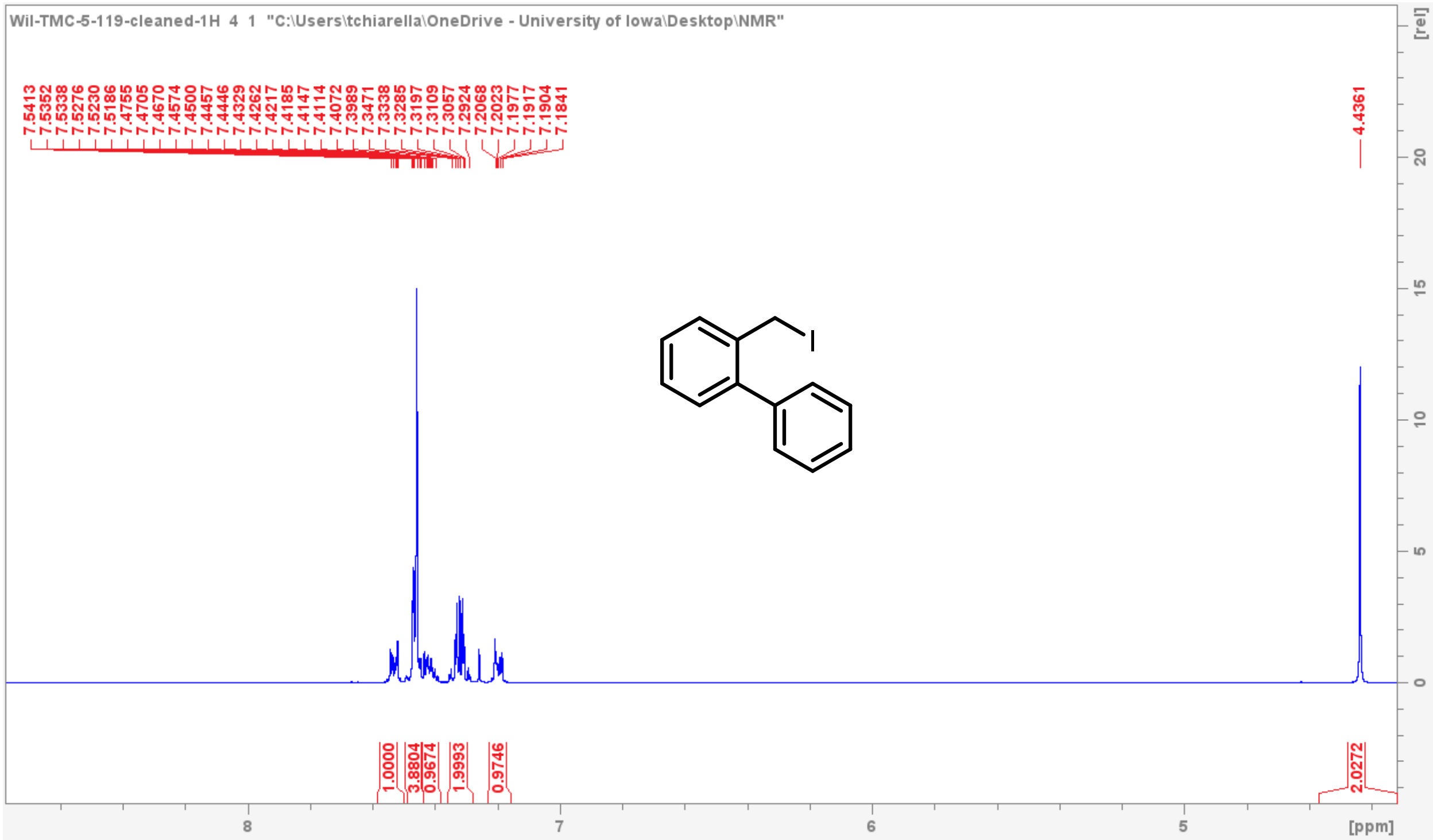

Figure S13: ^{13}C{^{1}H}-NMR (100 MHz) of 2c in $CDCl_3$

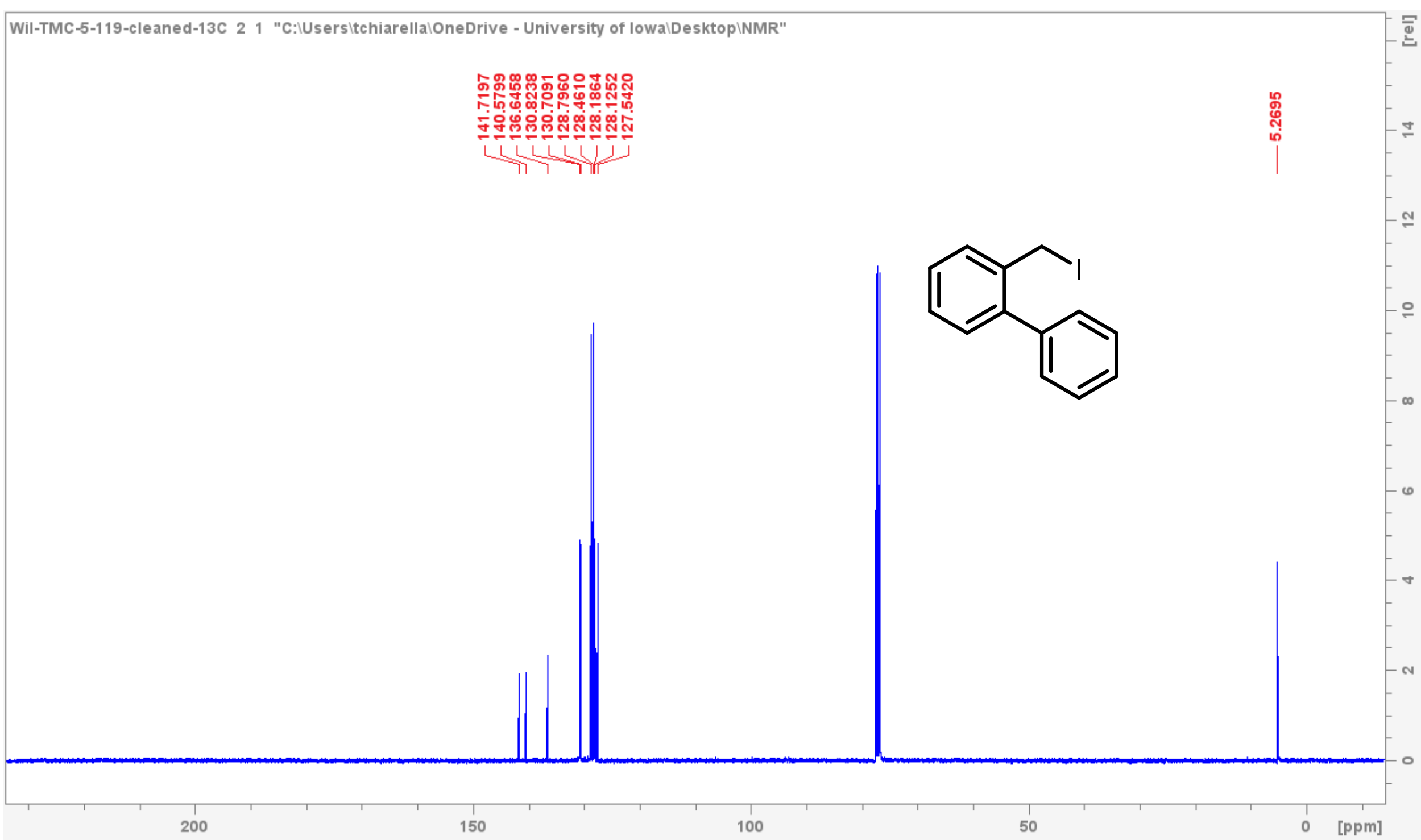

Figure S14: ^{1}H-NMR (400 MHz) of 2d in $CDCl_3$

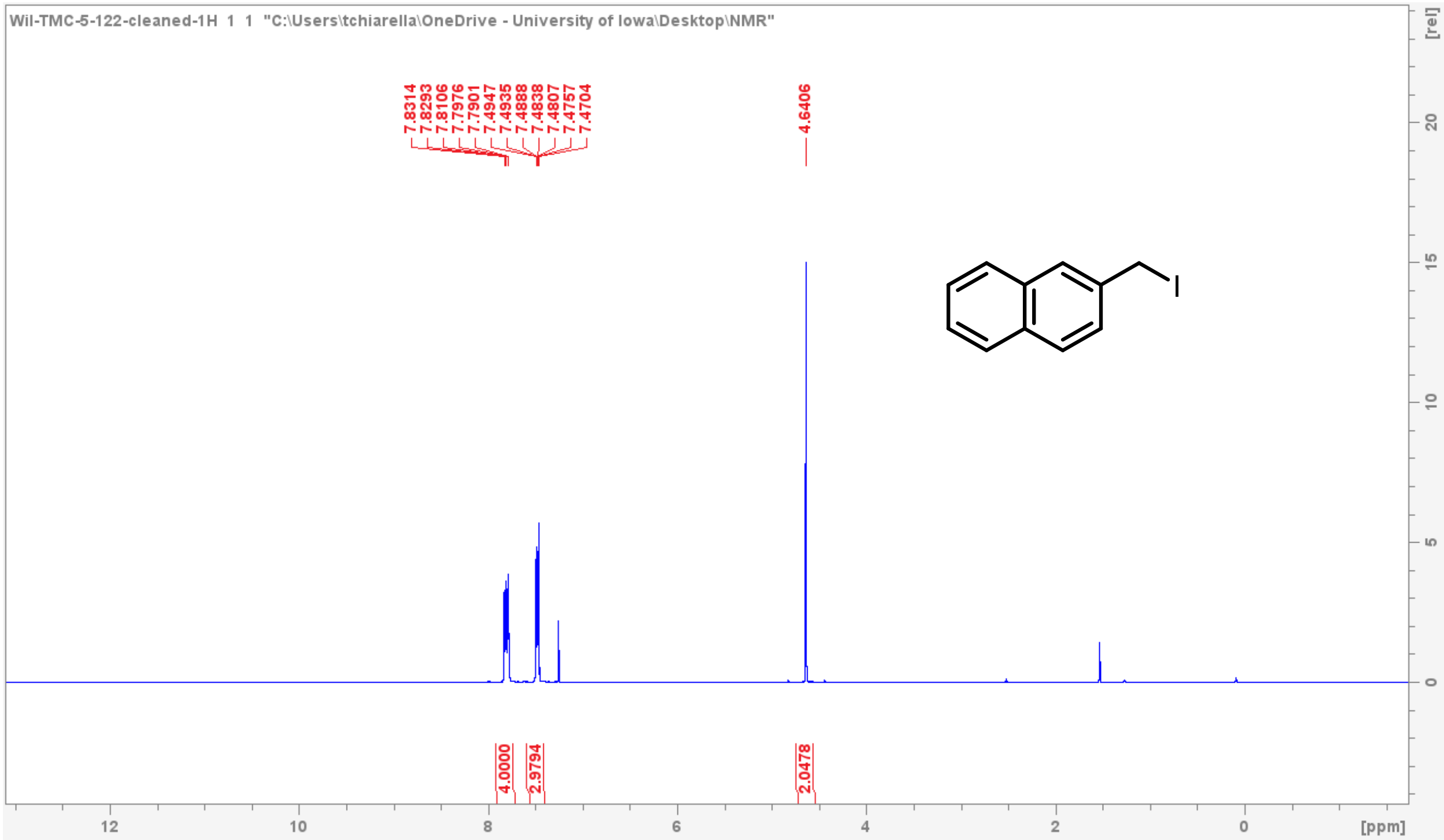

Figure S15: $^{13}C\{^{1}H\}$-NMR (100 MHz) of 2d in $CDCl_3$

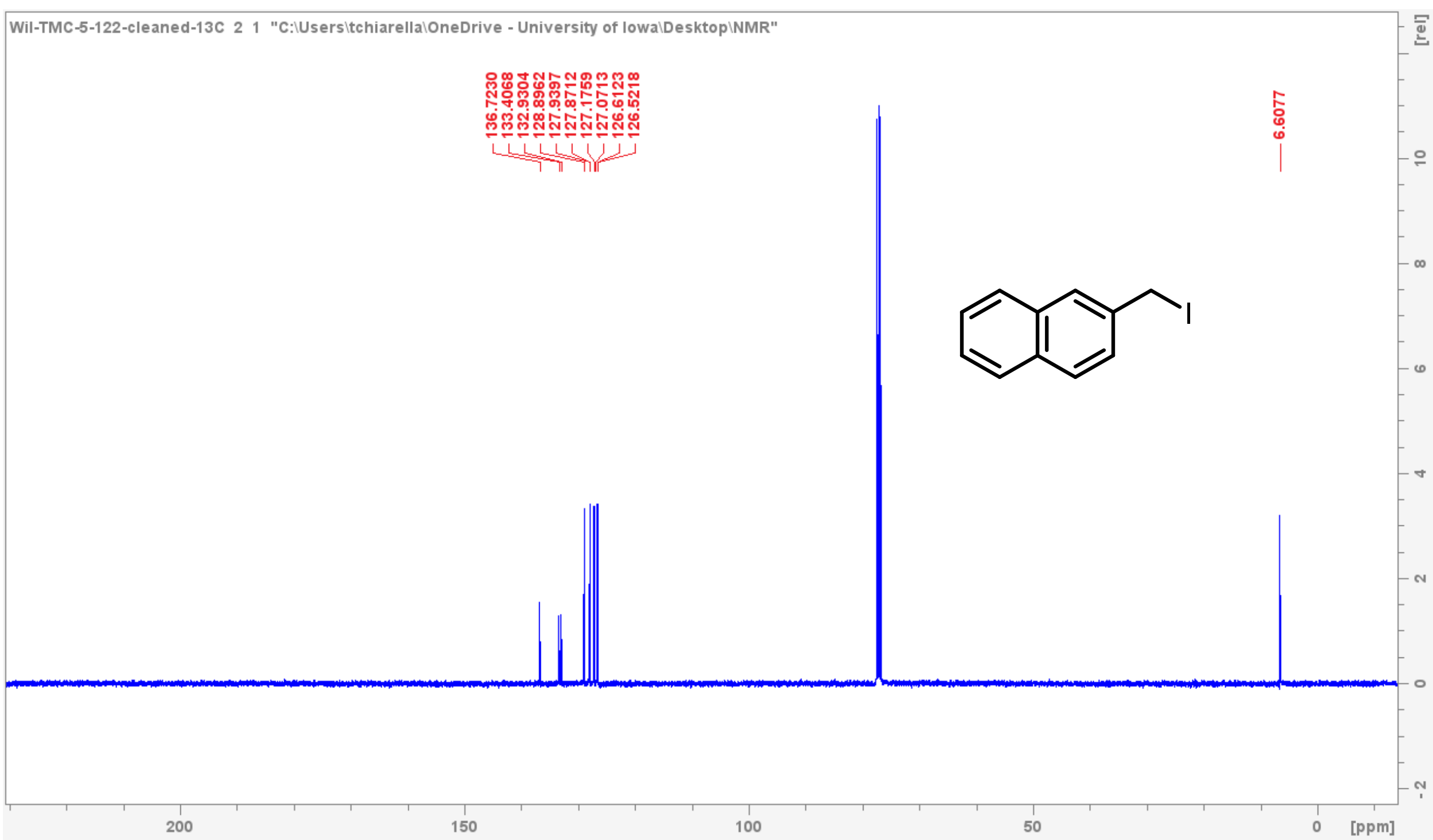

Figure S16: ^{1}H-NMR (400 MHz) of 2e in $CDCl_3$

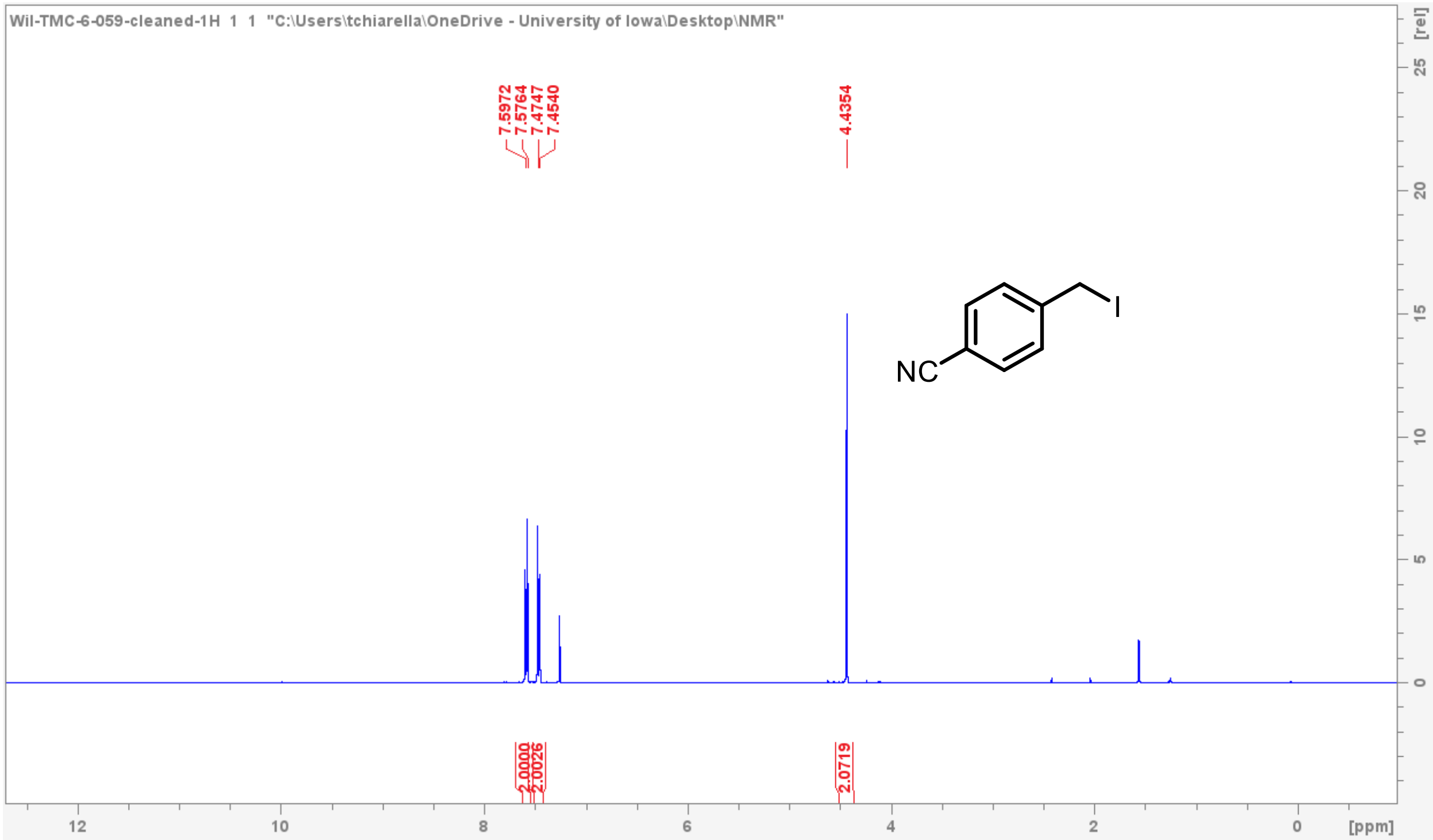

Figure S17: $^{13}C\{^{1}H\}$-NMR (100 MHz) of 2e in $CDCl_3$

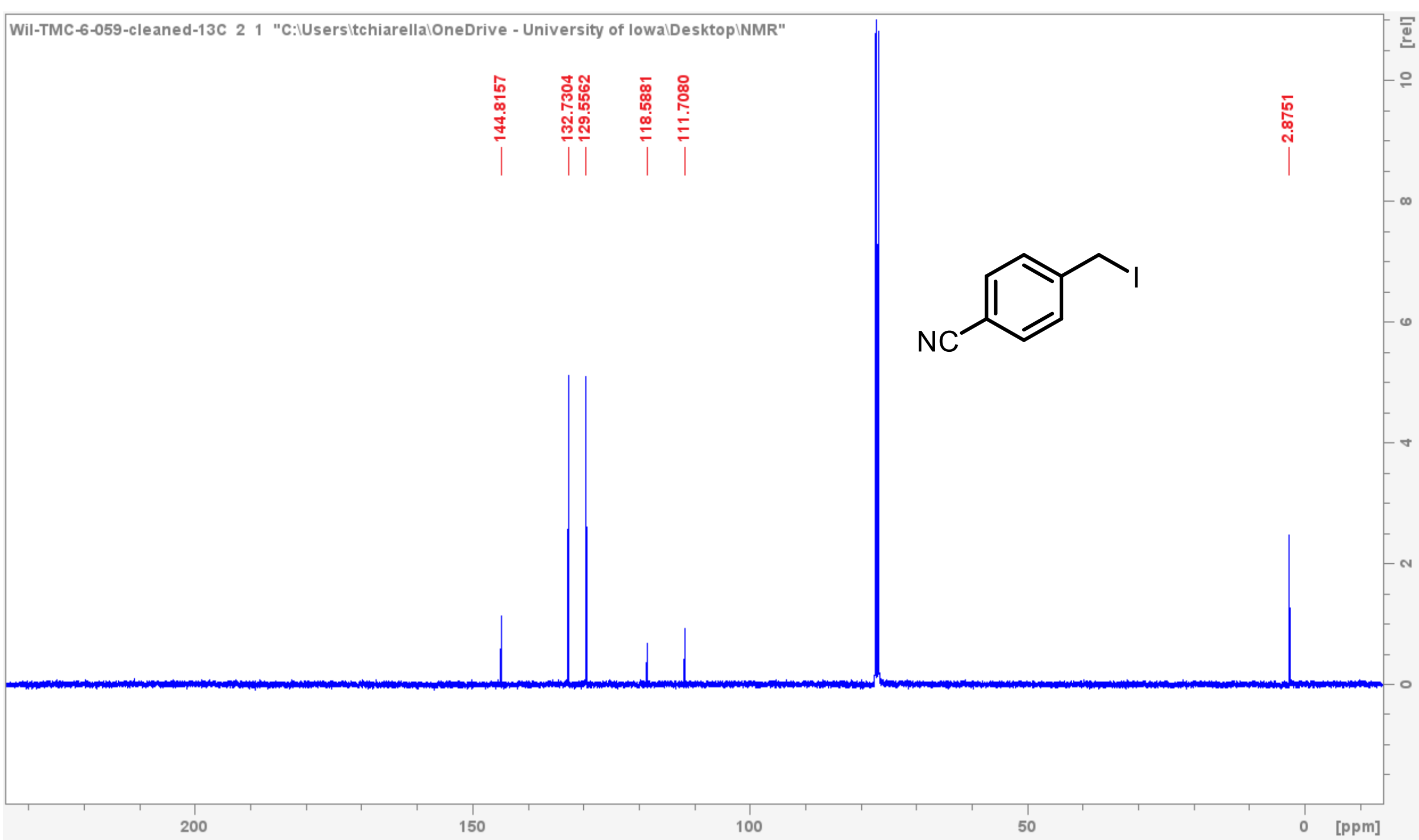

Figure S18: ^{1}H-NMR (400 MHz) of 2f in $CDCl_3$

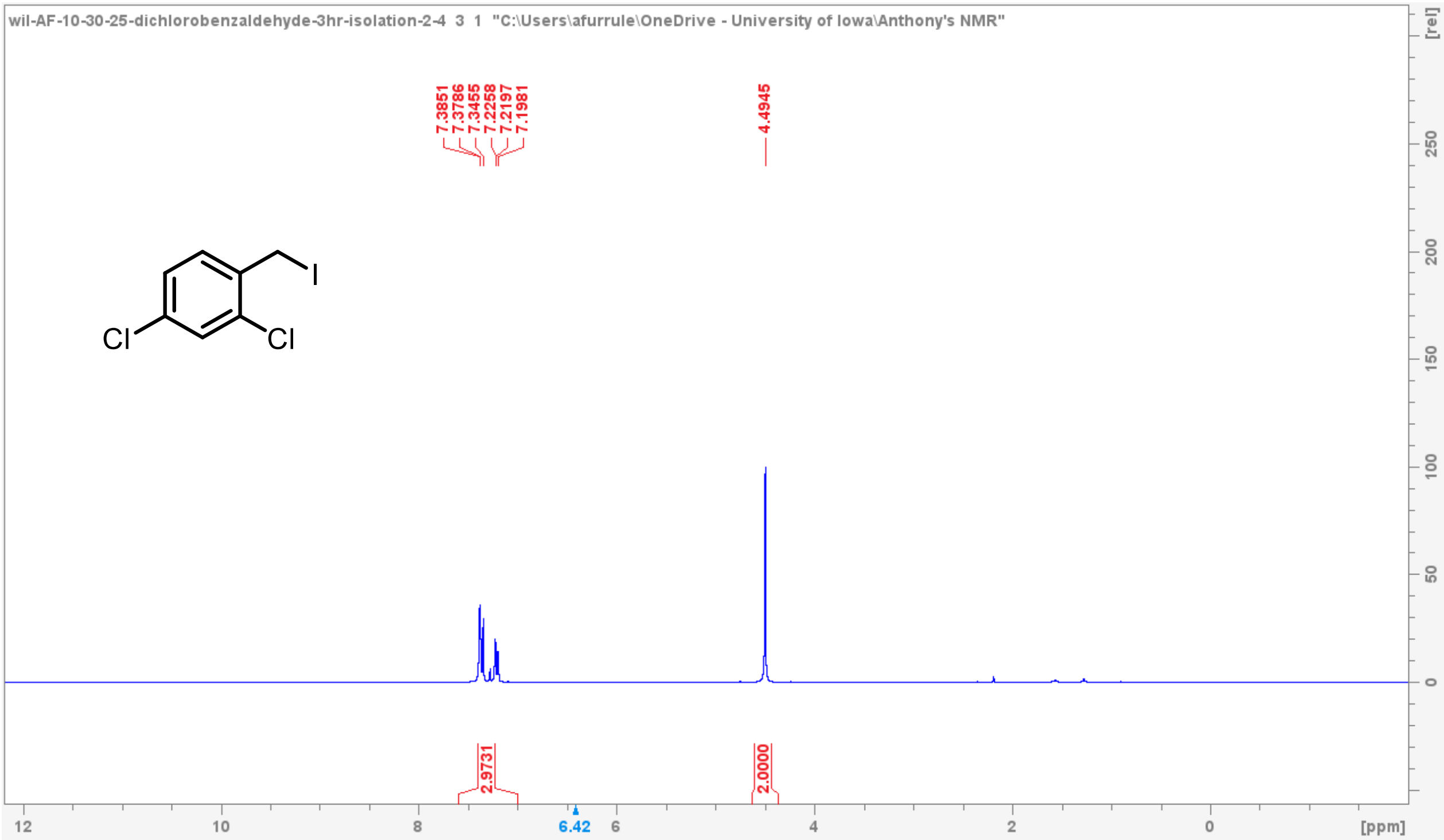

Figure S19: $^{13}C\{^{1}H\}$-NMR (100 MHz) of 2f in $CDCl_3$

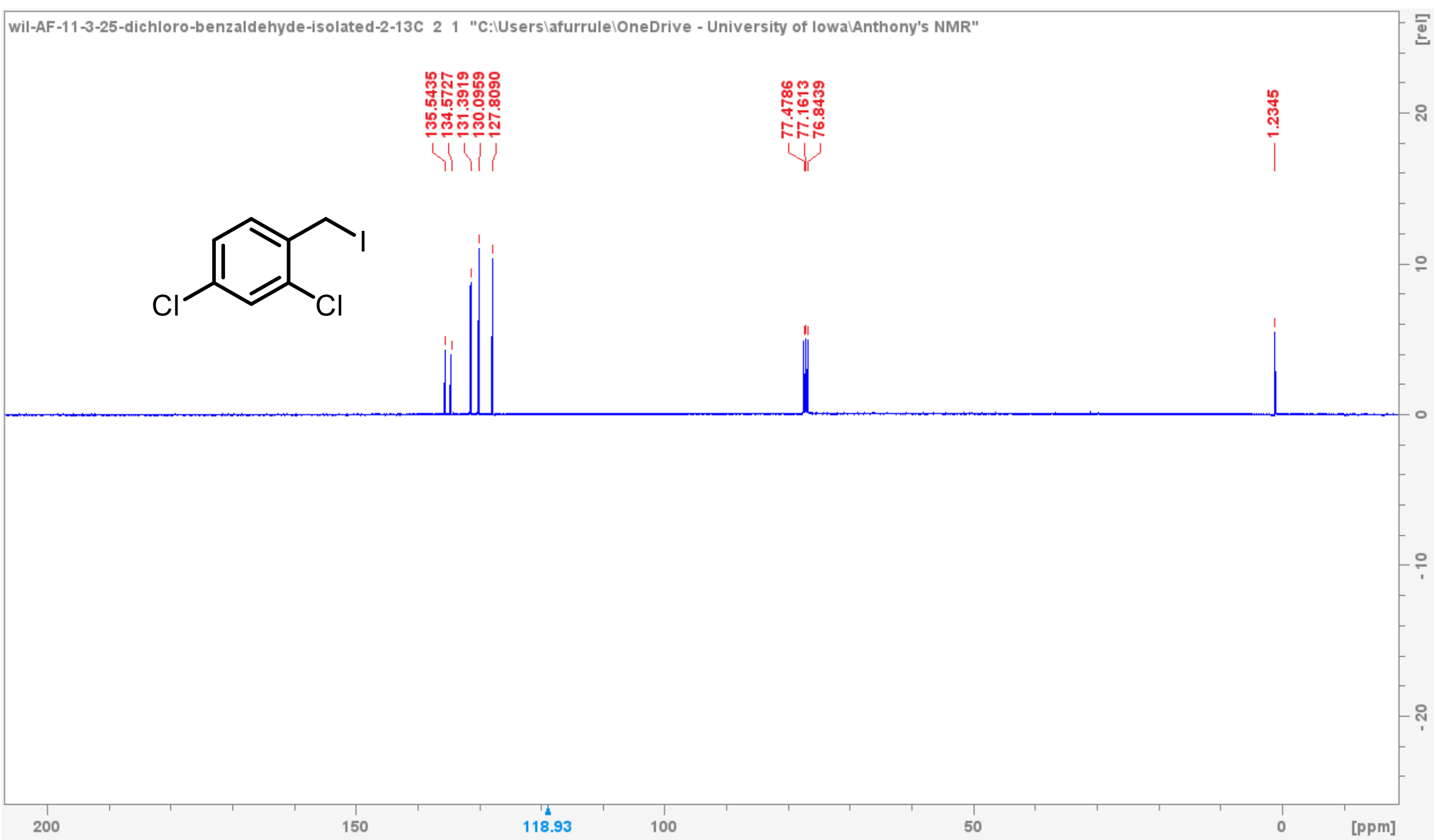

Figure S20: ^{1}H-NMR (400 MHz) of 2g in $CDCl_3$

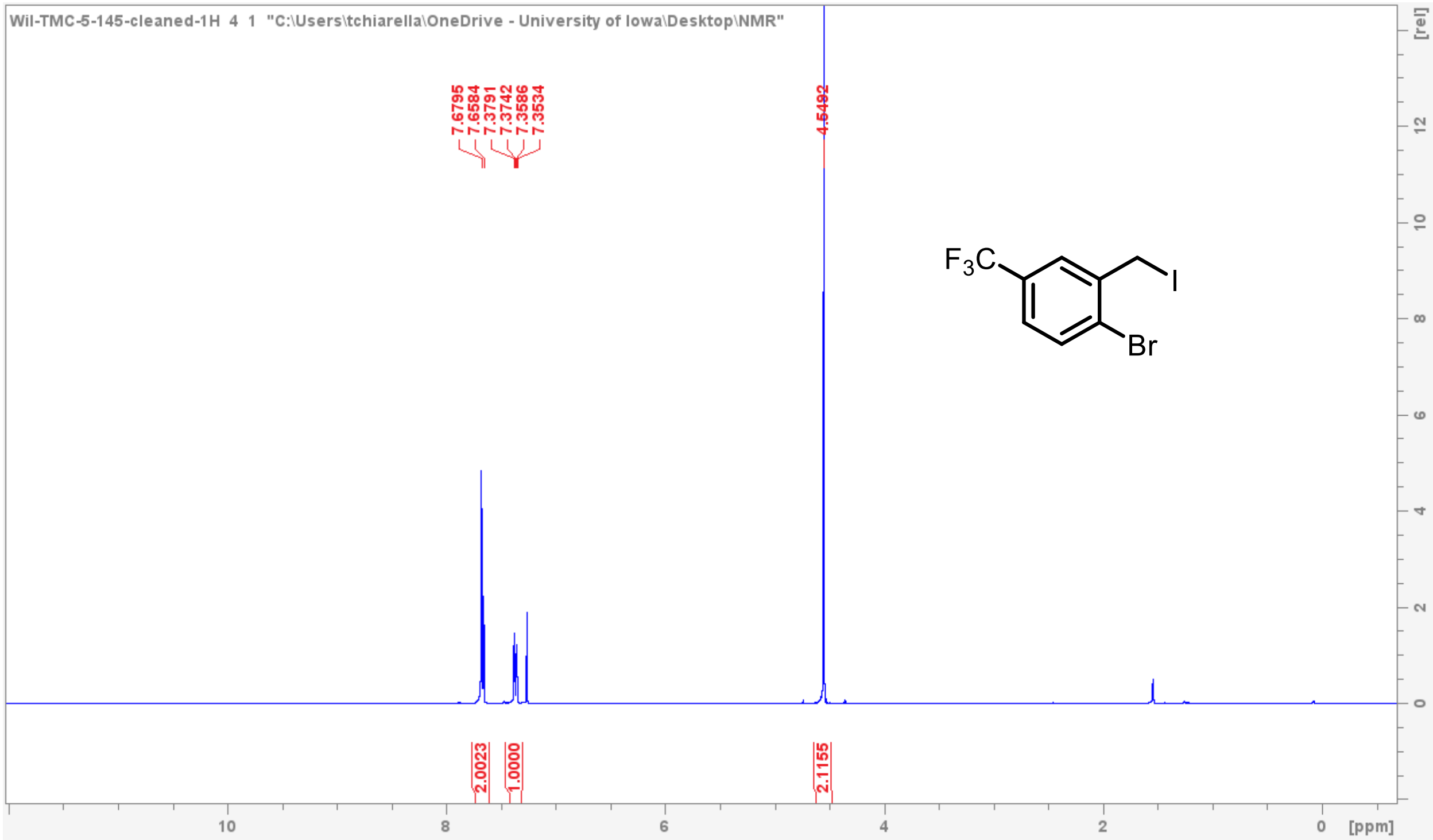

Figure S21: $^{13}C\{^{1}H\}$-NMR (100 MHz) of 2g in $CDCl_3$

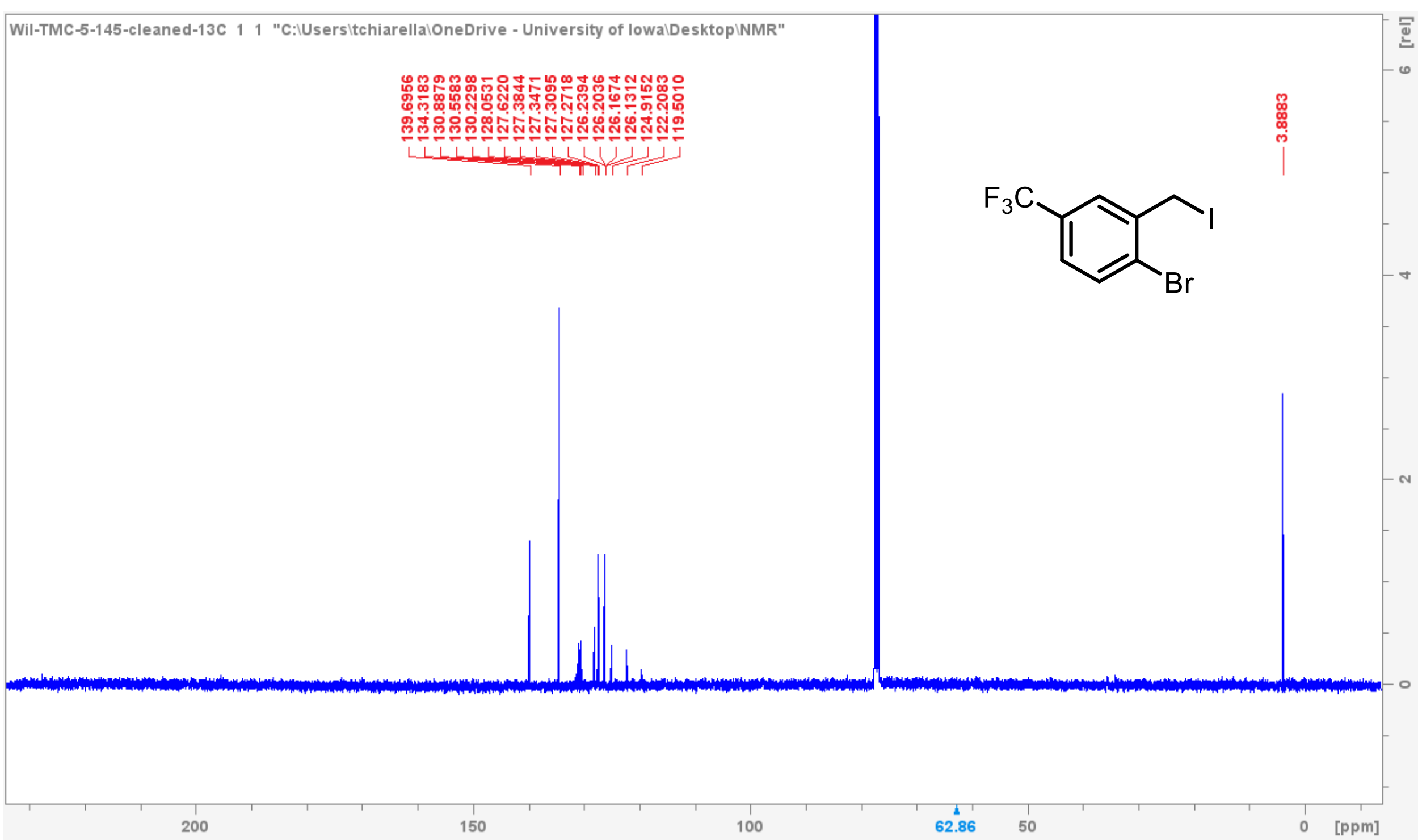

Figure S22: ^{19}F-NMR (377 MHz) of 2g in $CDCl_3$

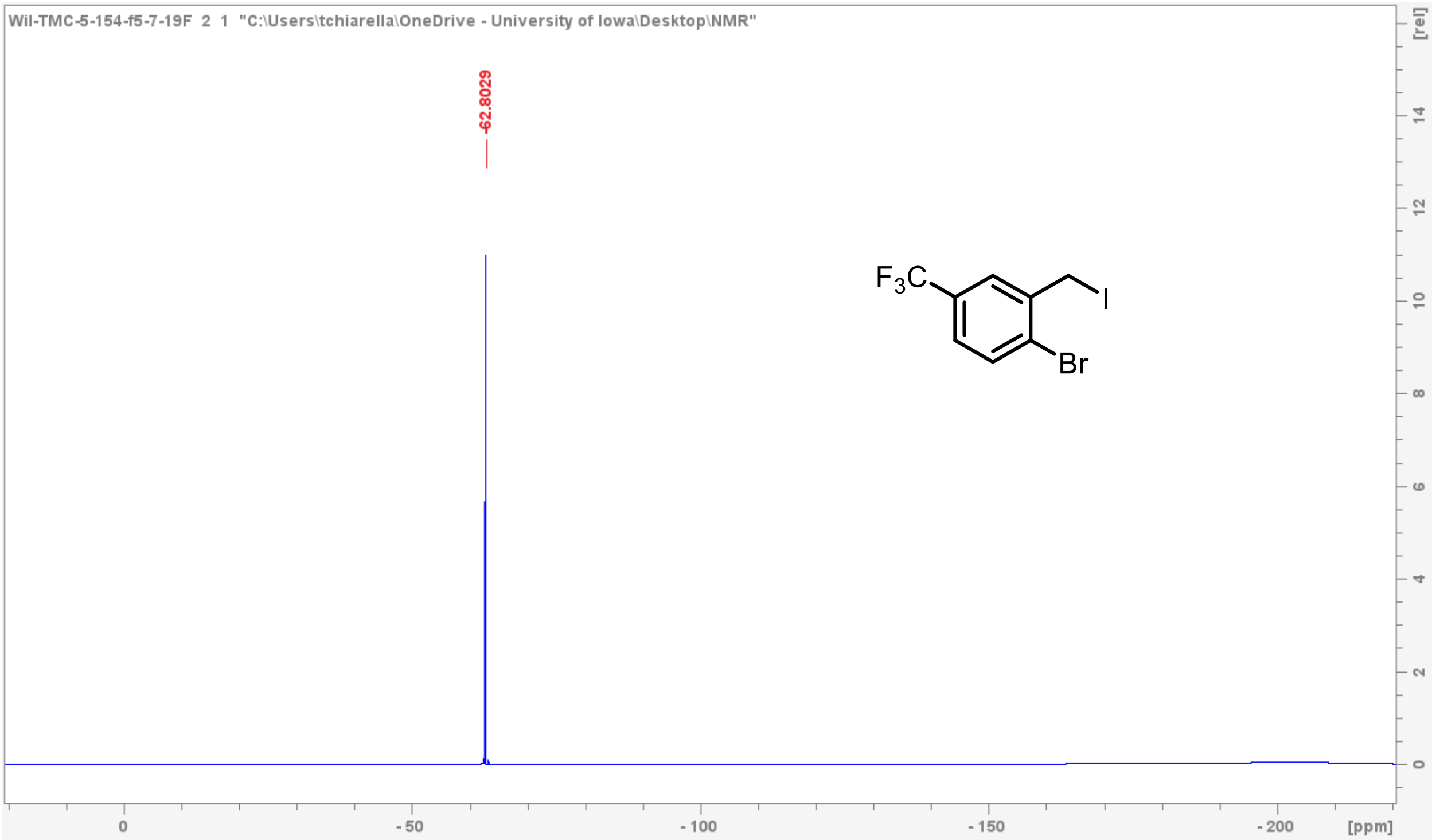

Figure S23: HSQC of 2g in $CDCl_3$

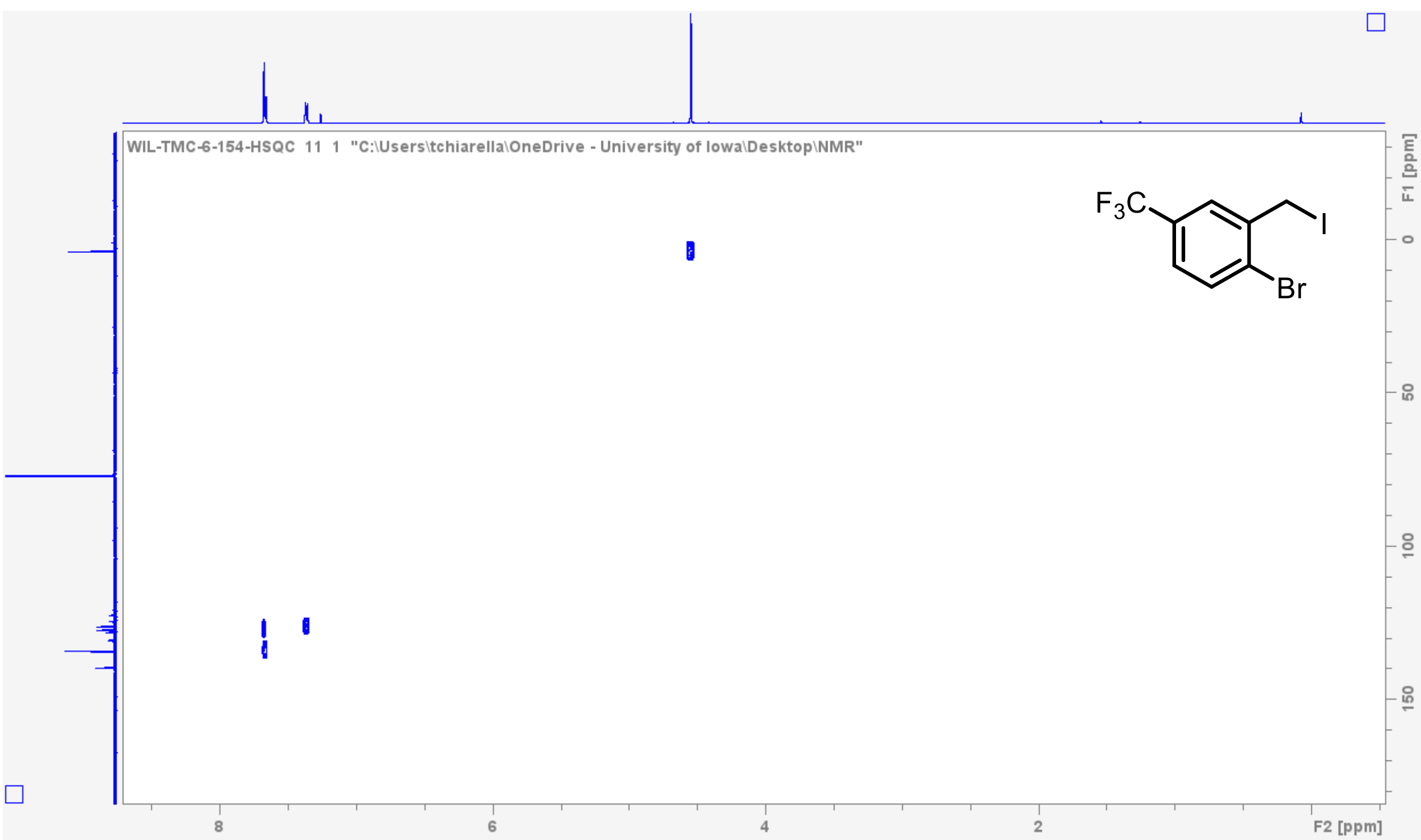

Figure S24: ^{1}H-NMR (600 MHz) of 2h in $CDCl_3$

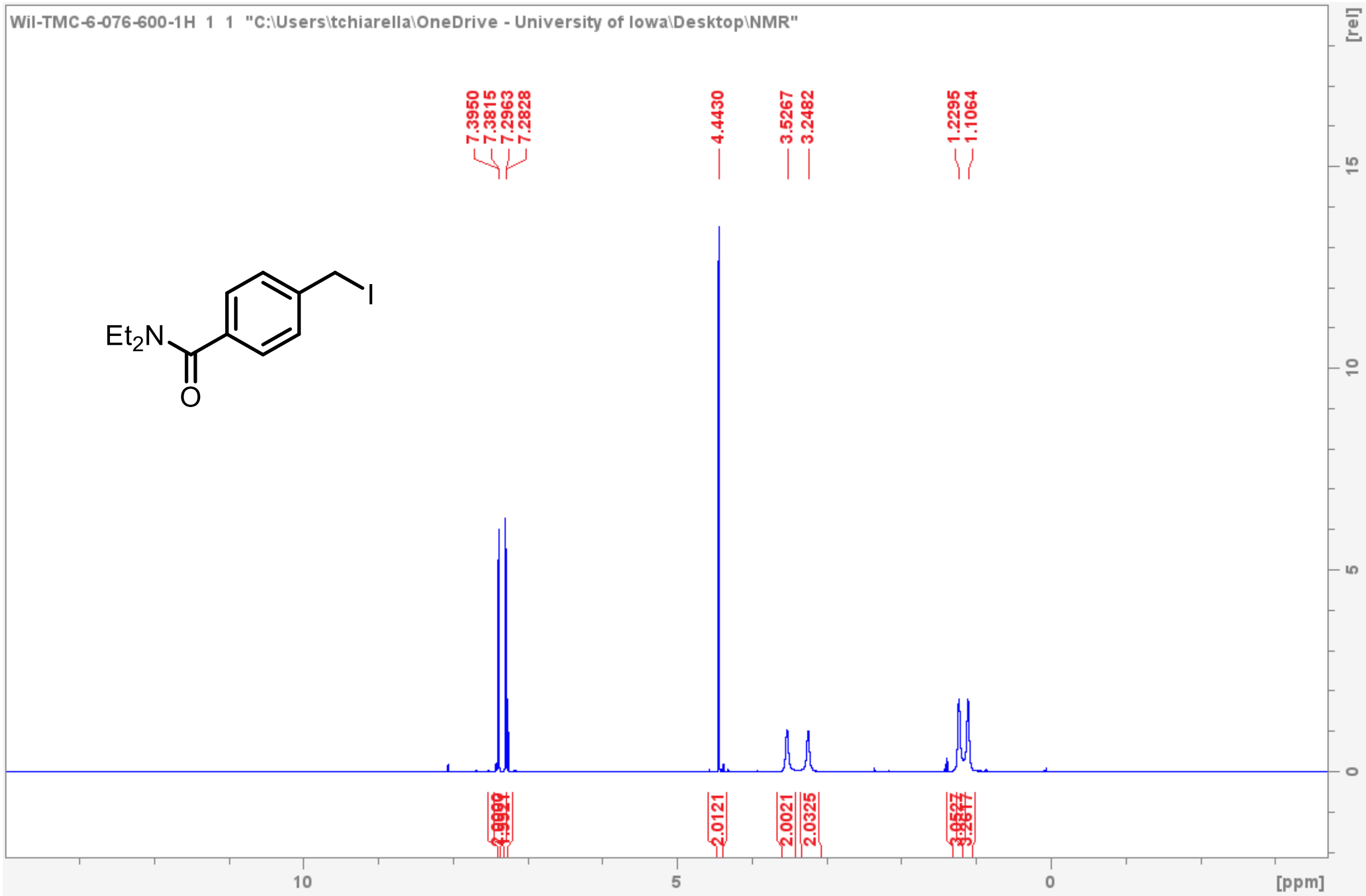

Figure S25: Zoomed in ^{1}H-NMR (600 MHz) of 2h in $CDCl_3$

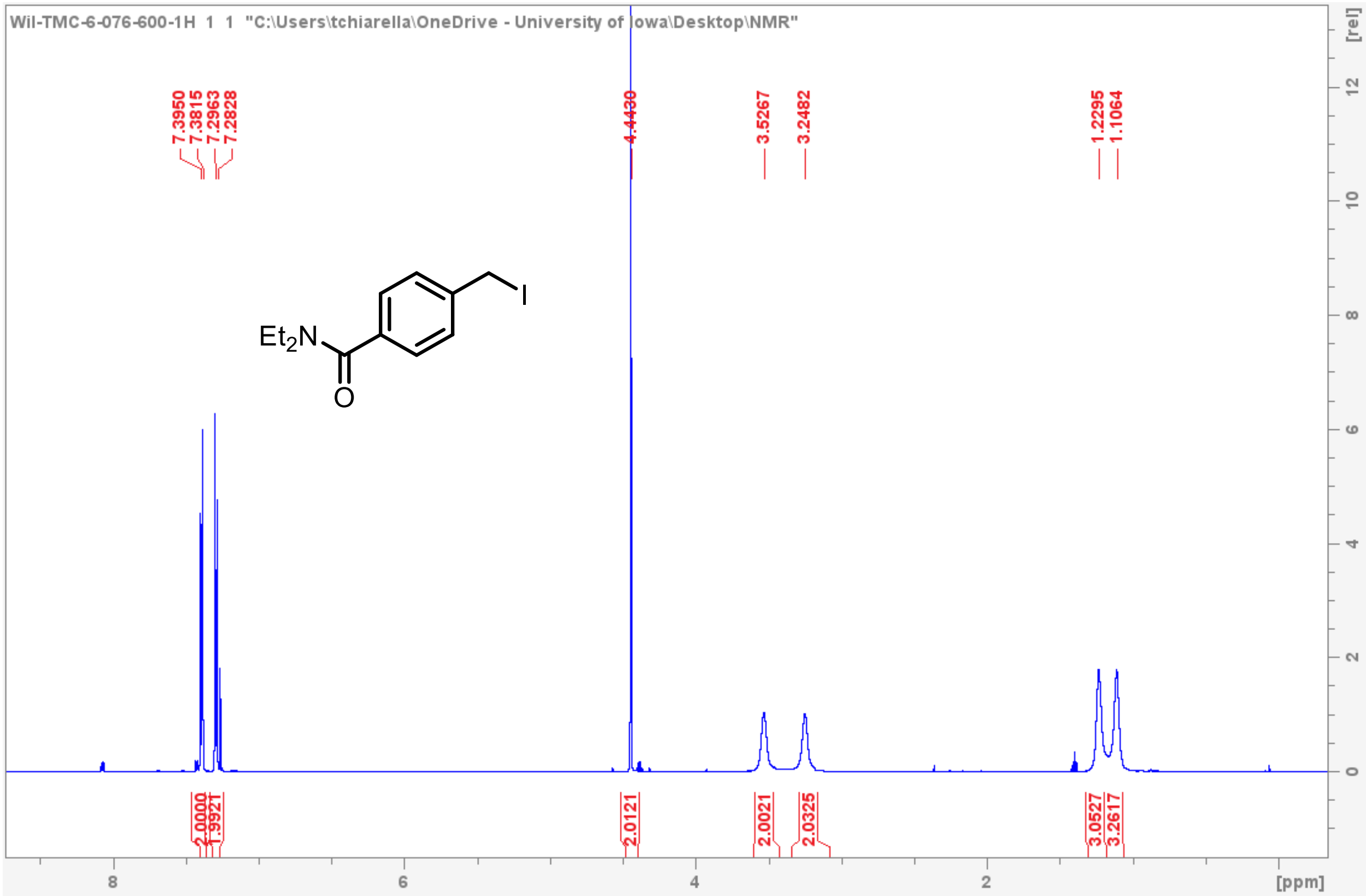

Figure S26: $^{13}C\{^1H\}$-NMR (150 MHz) of 2h in $CDCl_3$

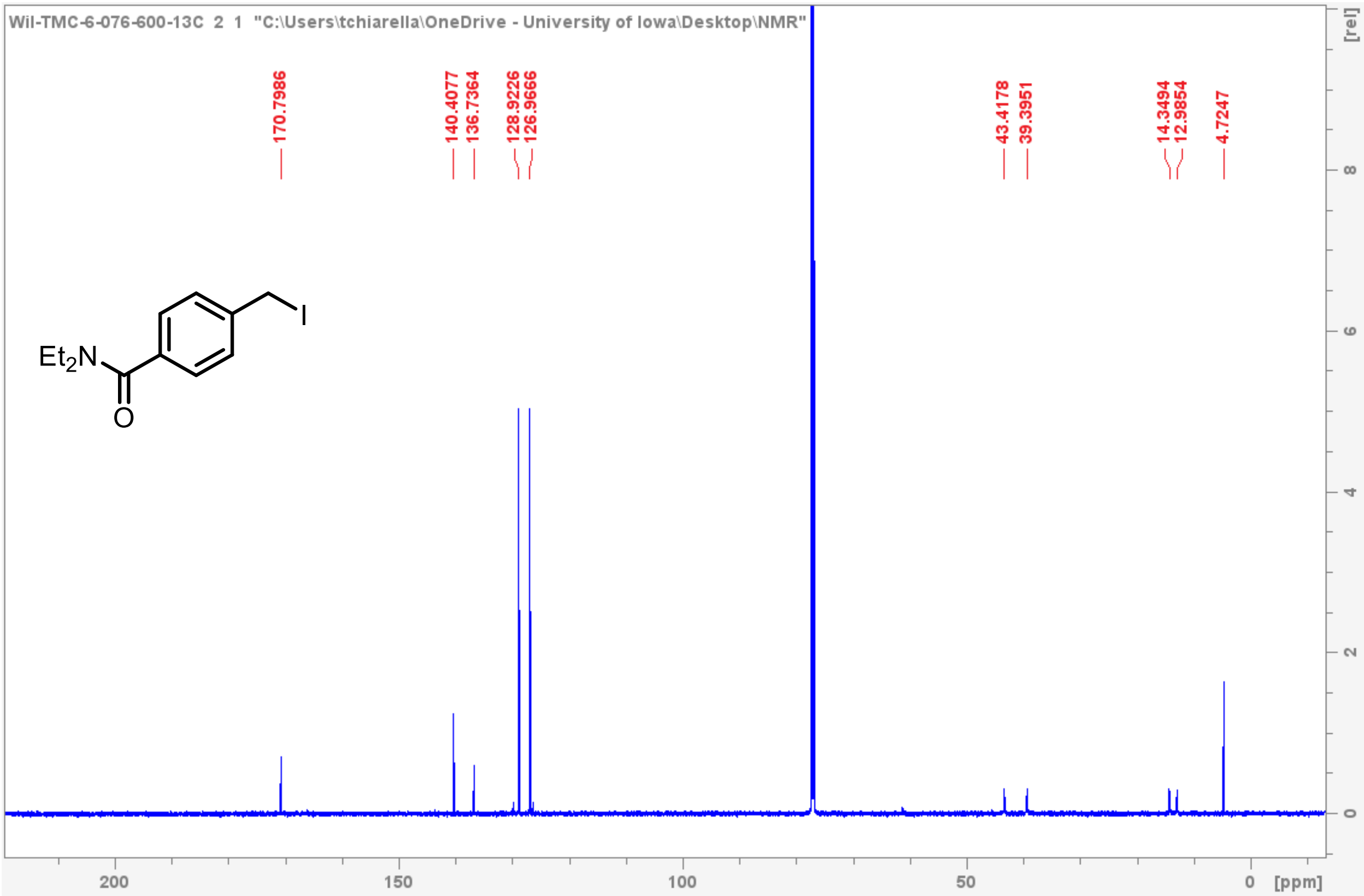

Figure S27: HSQC of 2h in $CDCl_3$

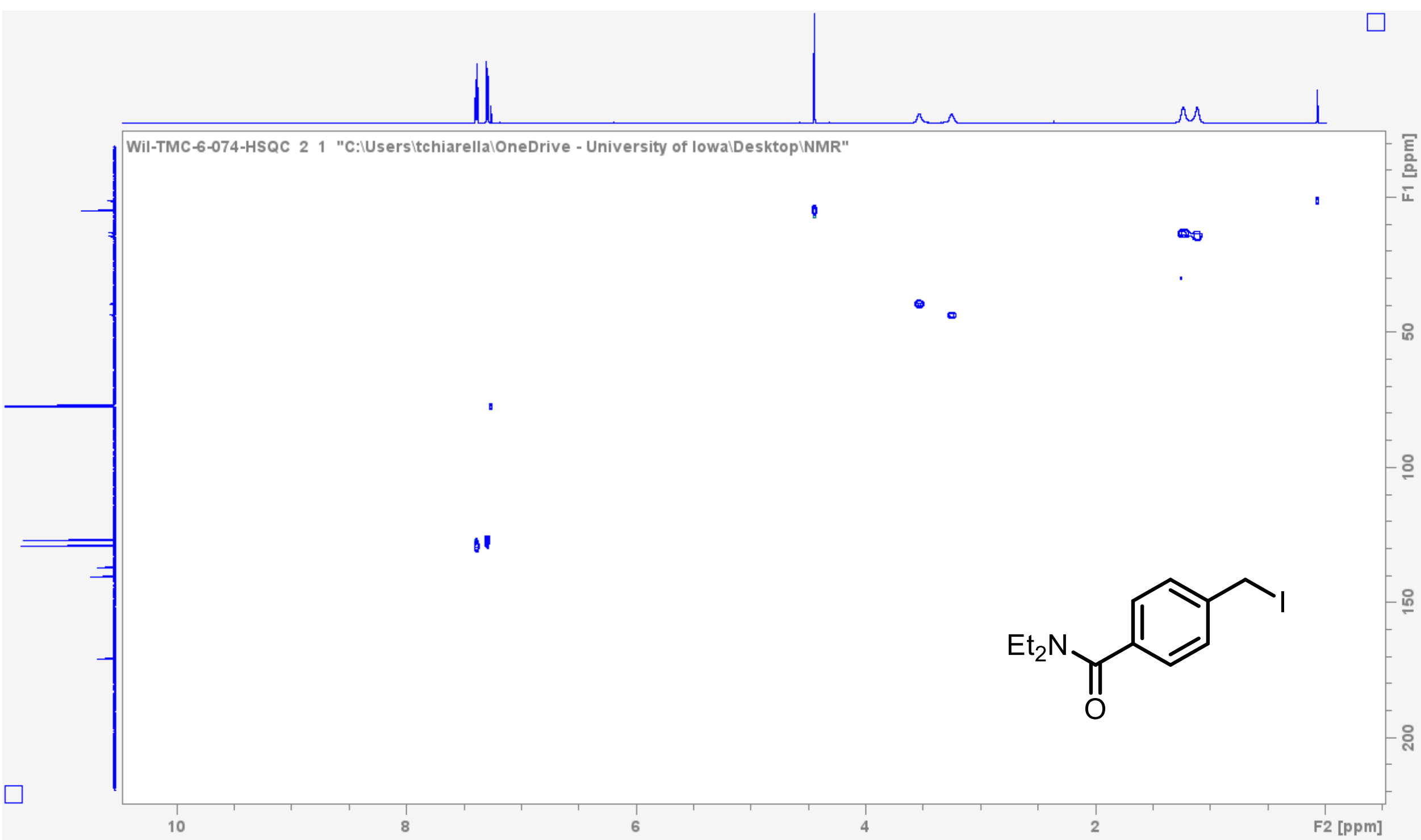

Figure S28: ^{1}H-NMR (400 MHz) of 2i in $CDCl_3$

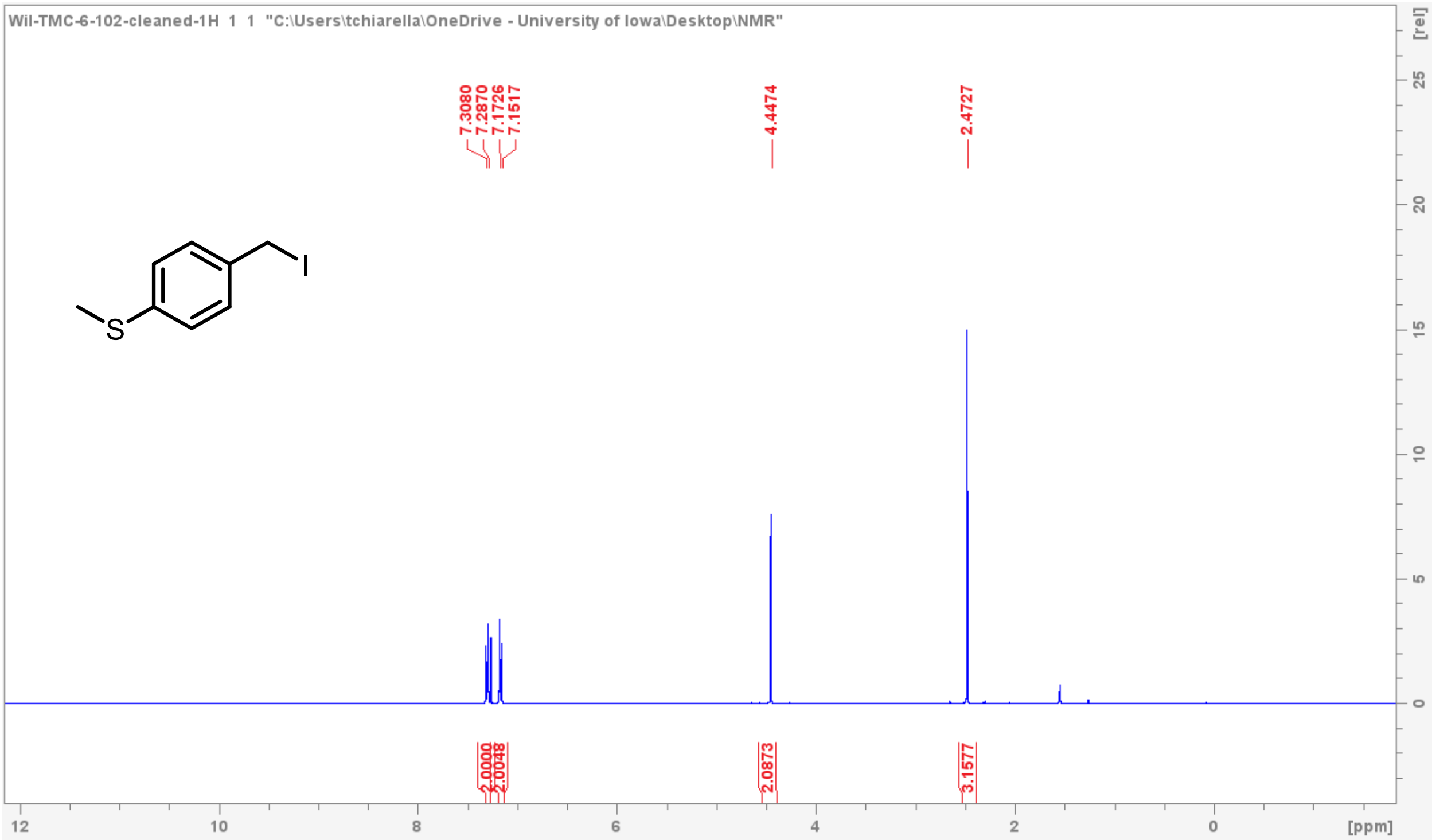

Figure S29: $^{13}C\{^1H\}$-NMR (100 MHz) of 2i in $CDCl_3$

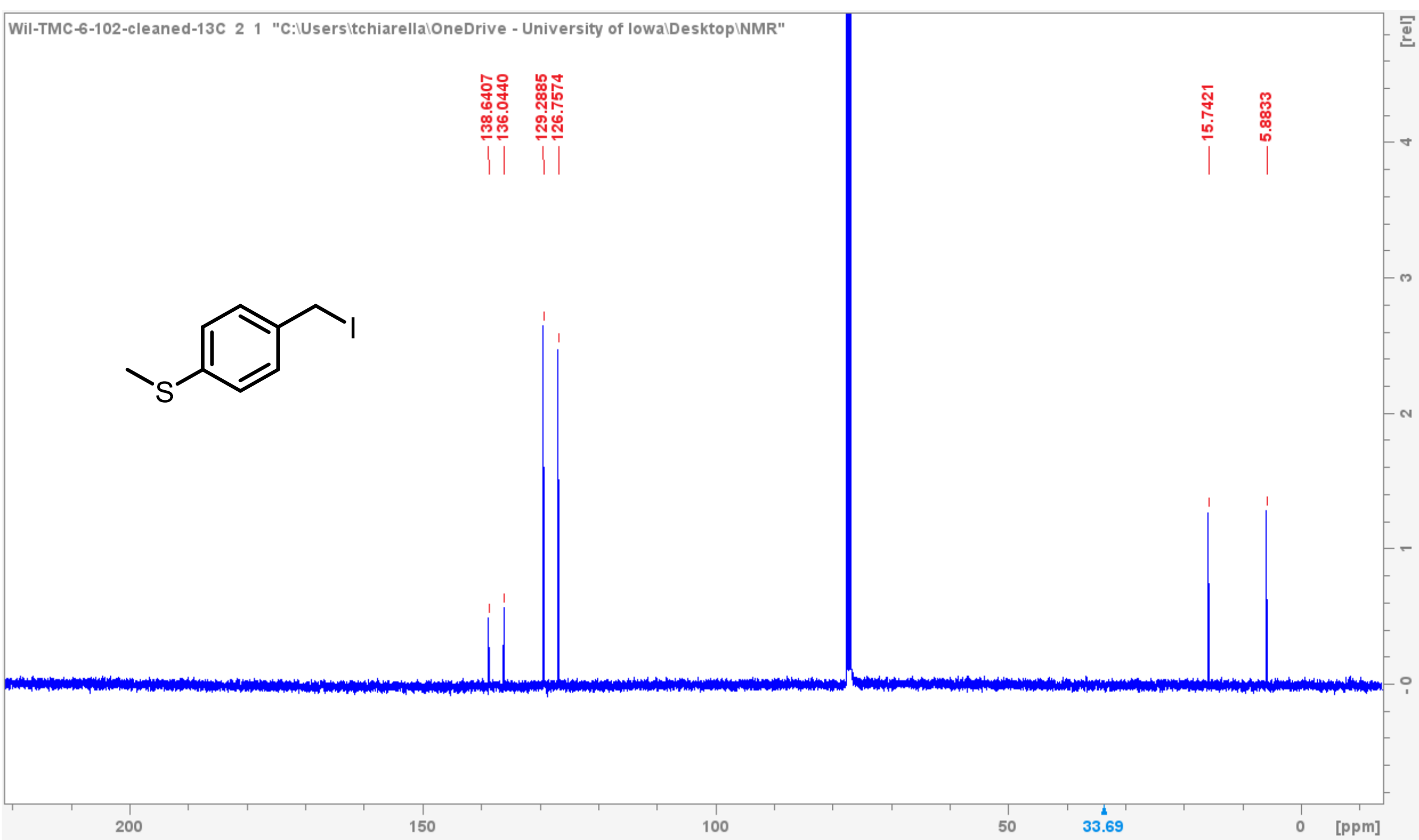

Figure S30: ^{1}H-NMR (400 MHz) of 2j in $CDCl_3$

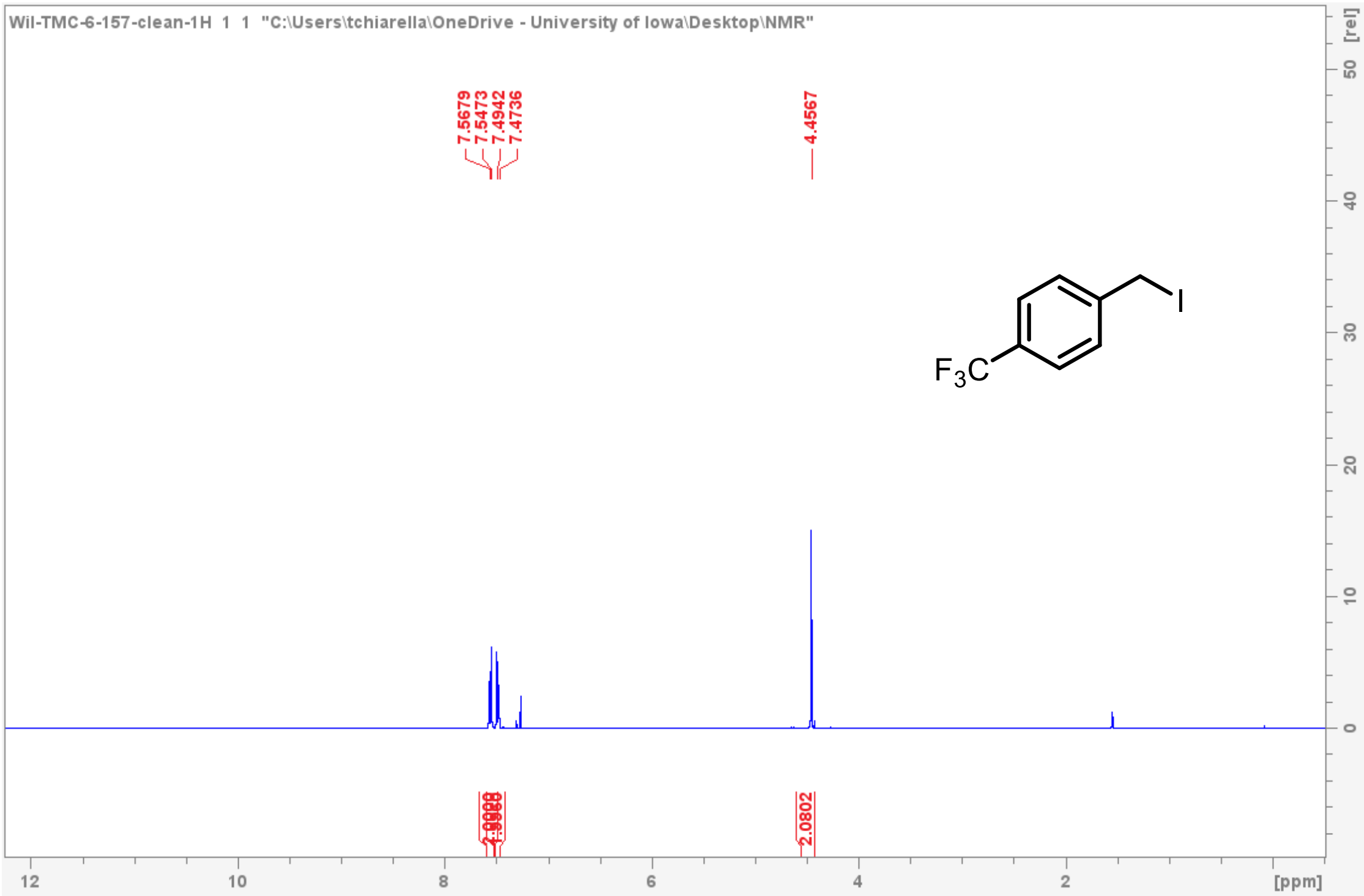

Figure S31: Zoomed in ^{1}H-NMR (400 MHz) of 2j in $CDCl_3$

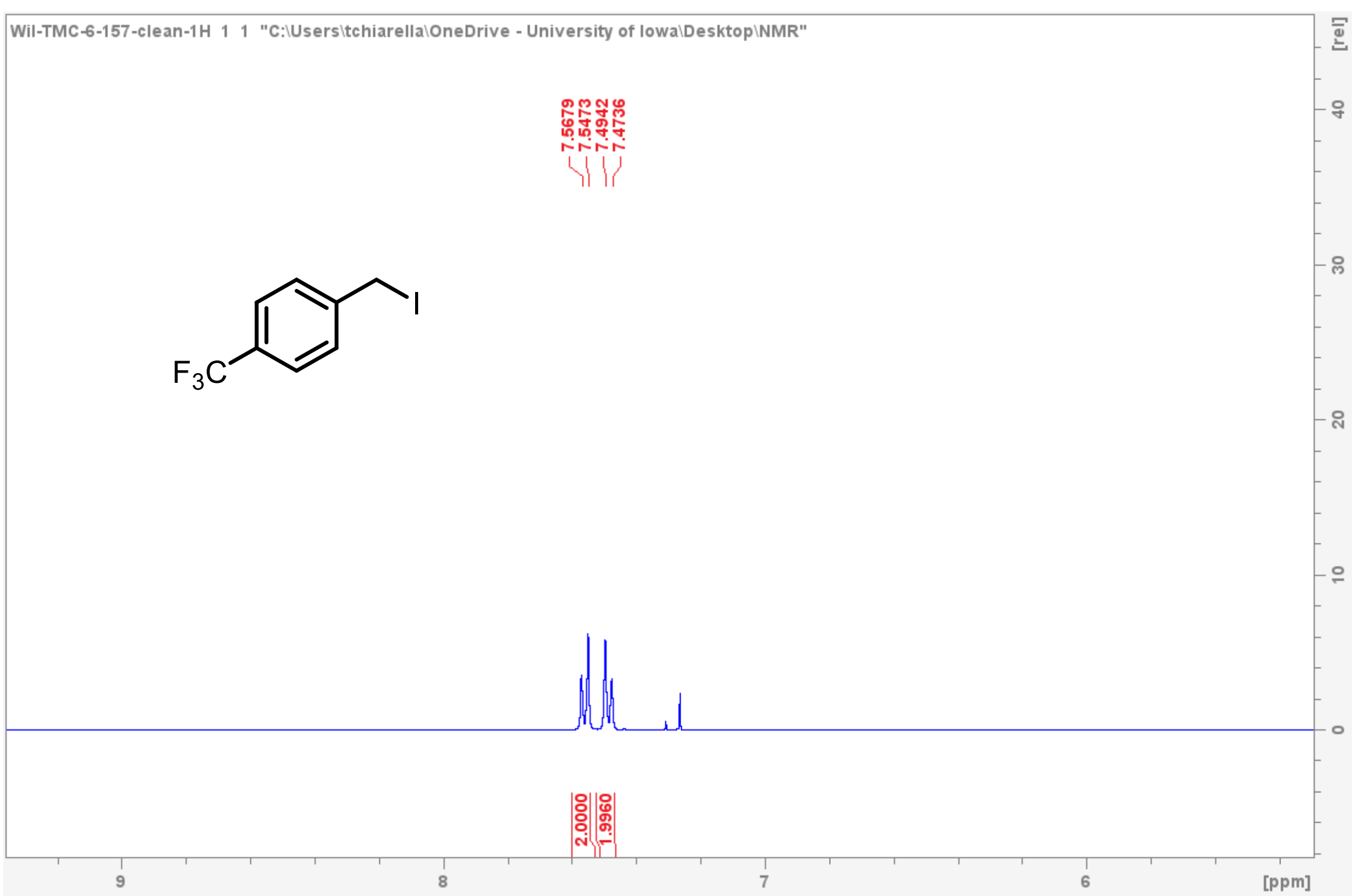

Figure S32: $^{13}C\{^{1}H\}$-NMR (100 MHz) of 2j in $CDCl_3$

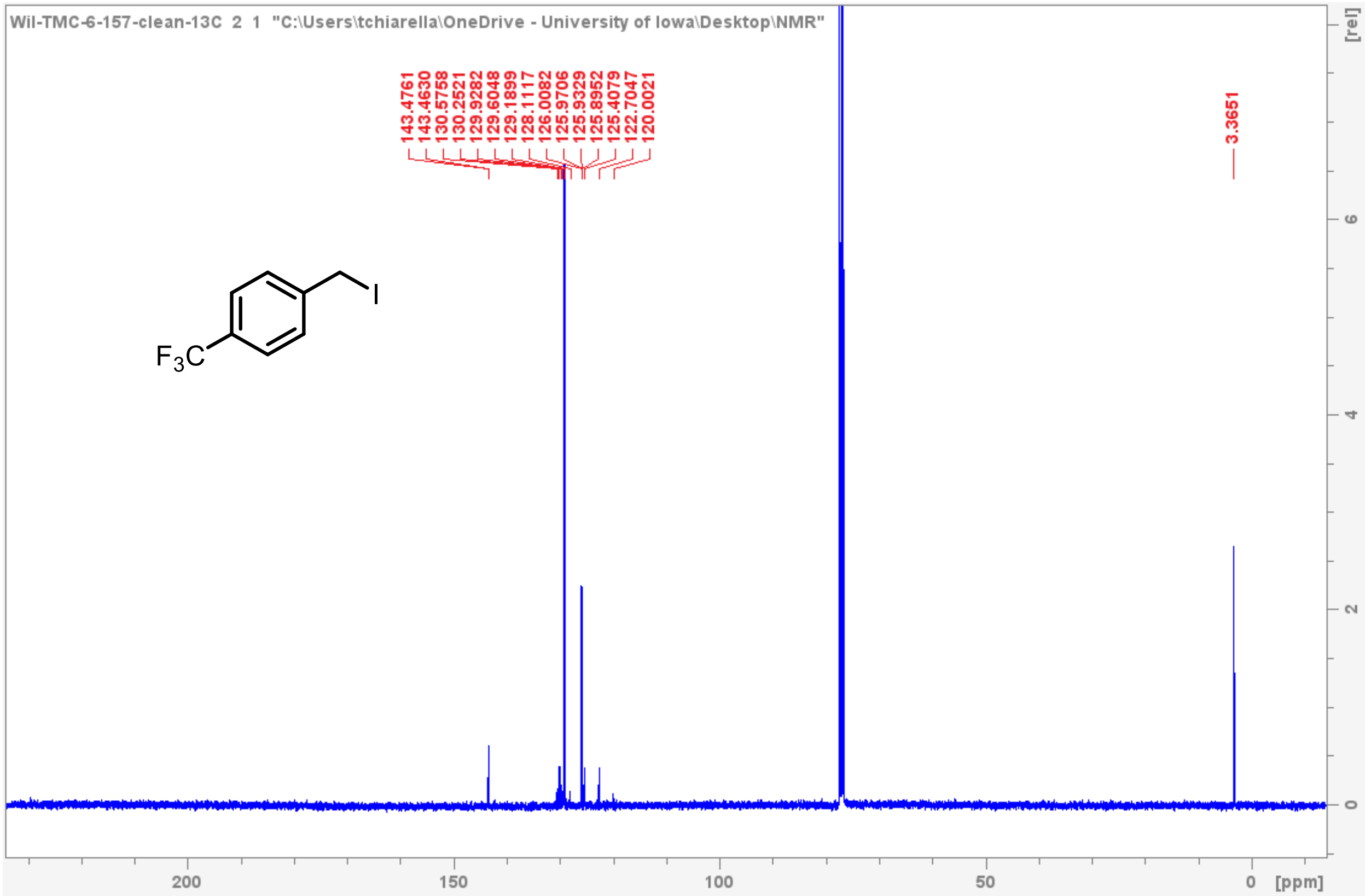

Figure S33: ^{19}F-NMR (377 MHz) of 2j in $CDCl_3$

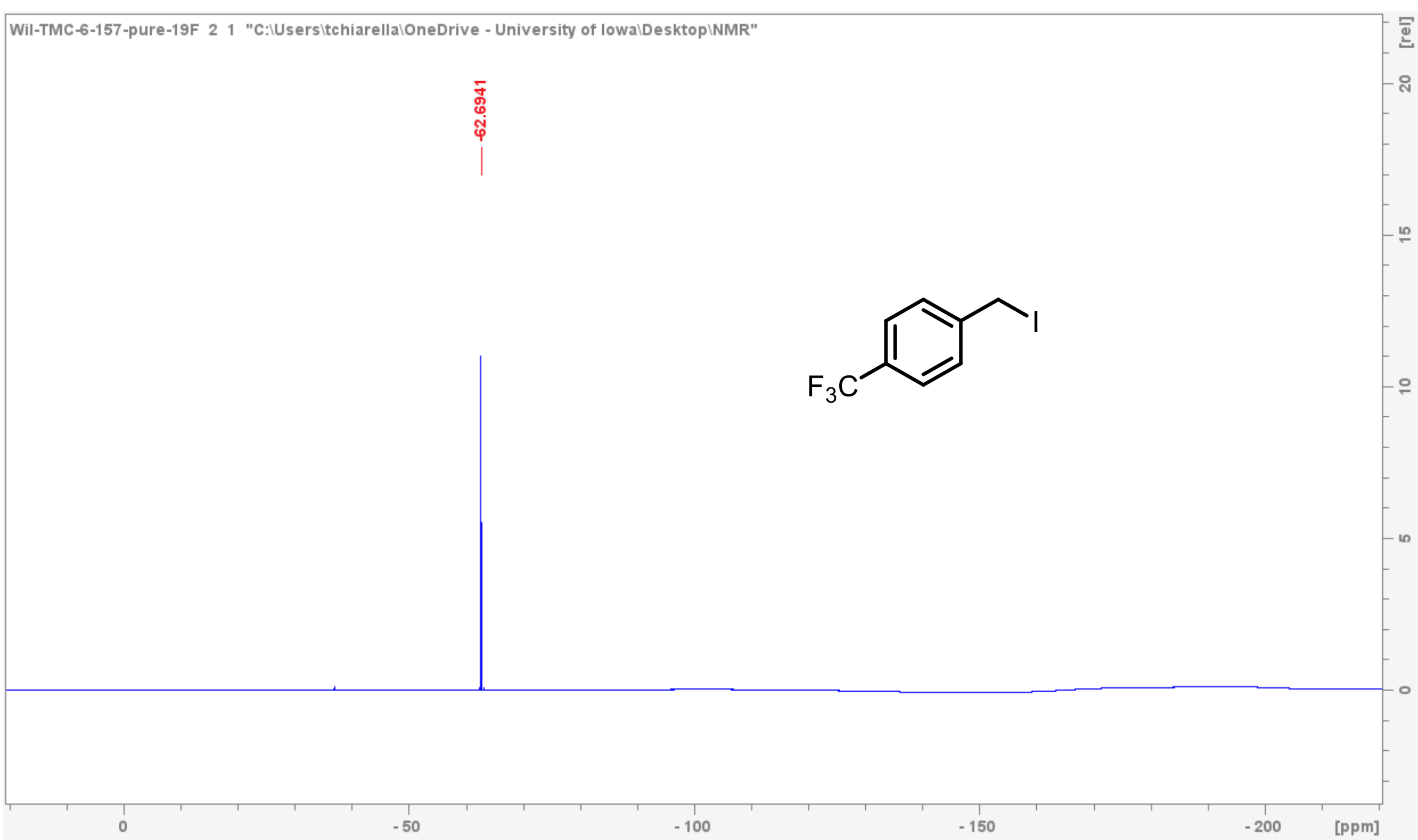

Figure S34: ^{1}H-NMR (300 MHz) of 2n in $CDCl_3$

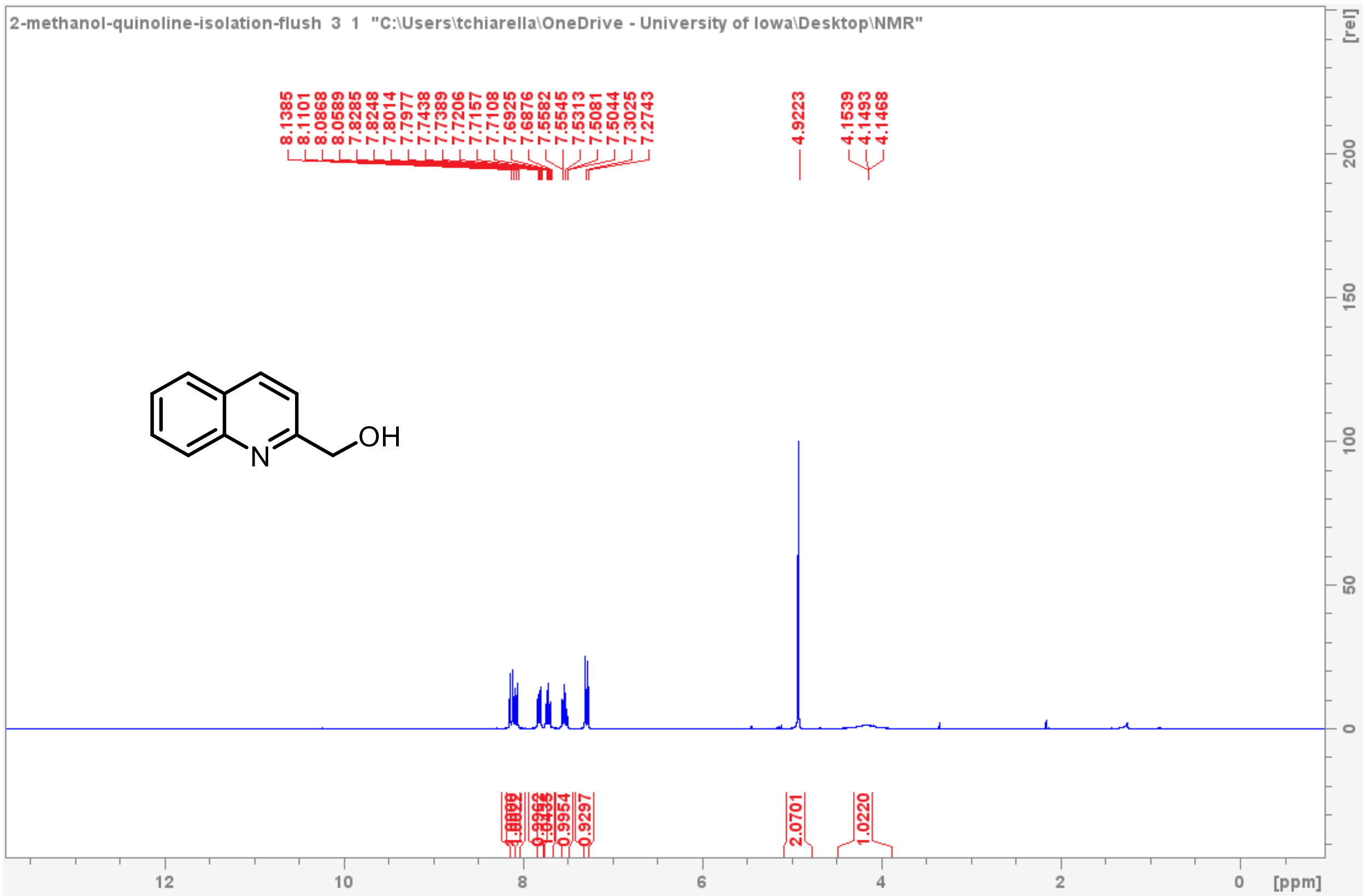

Figure S35: Zoomed in ^{1}H-NMR (300 MHz) of 2n in $CDCl_3$

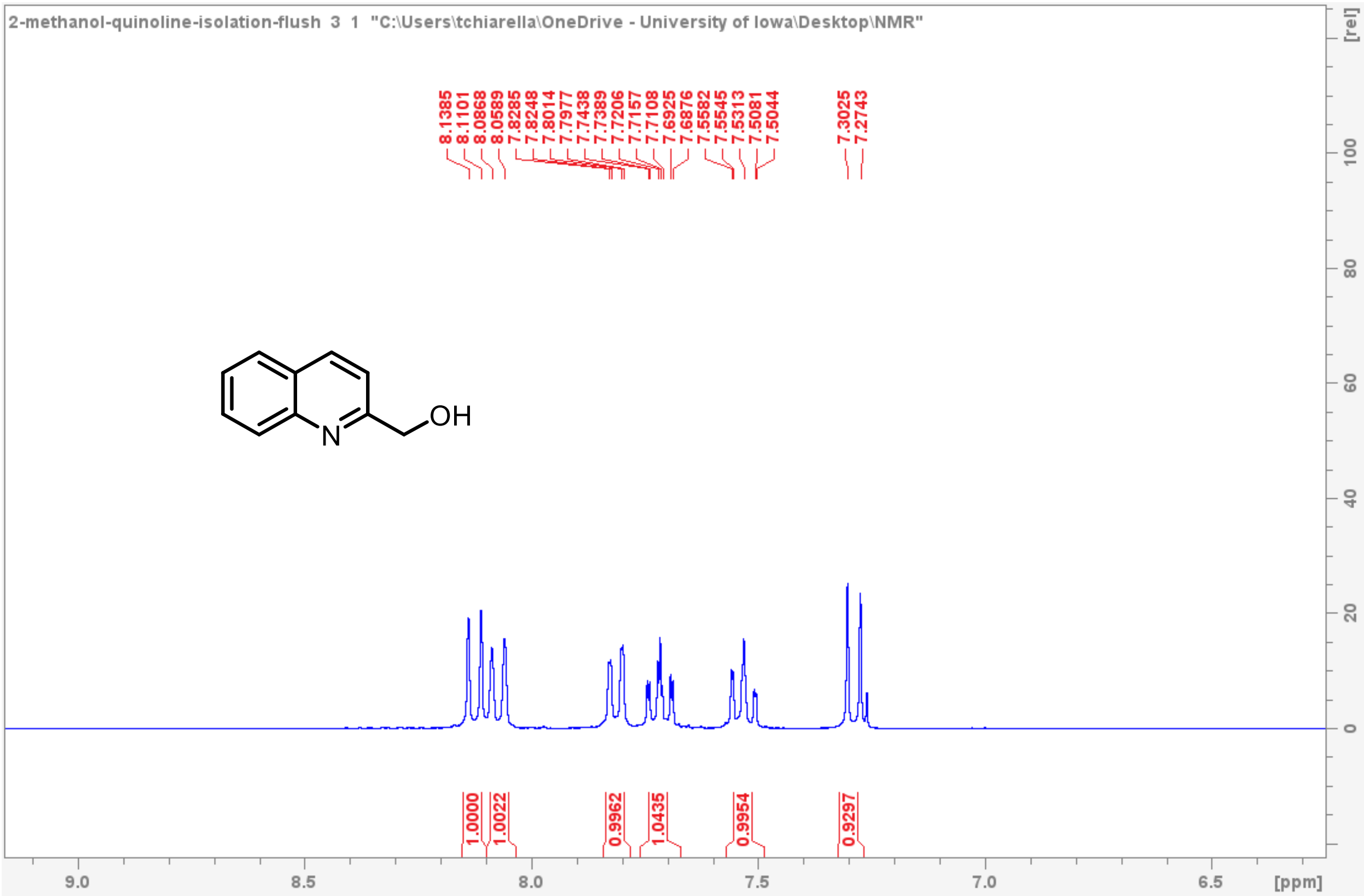

Figure S36: $^{13}C\{^{1}H\}$-NMR (75 MHz) of 2n in $CDCl_3$

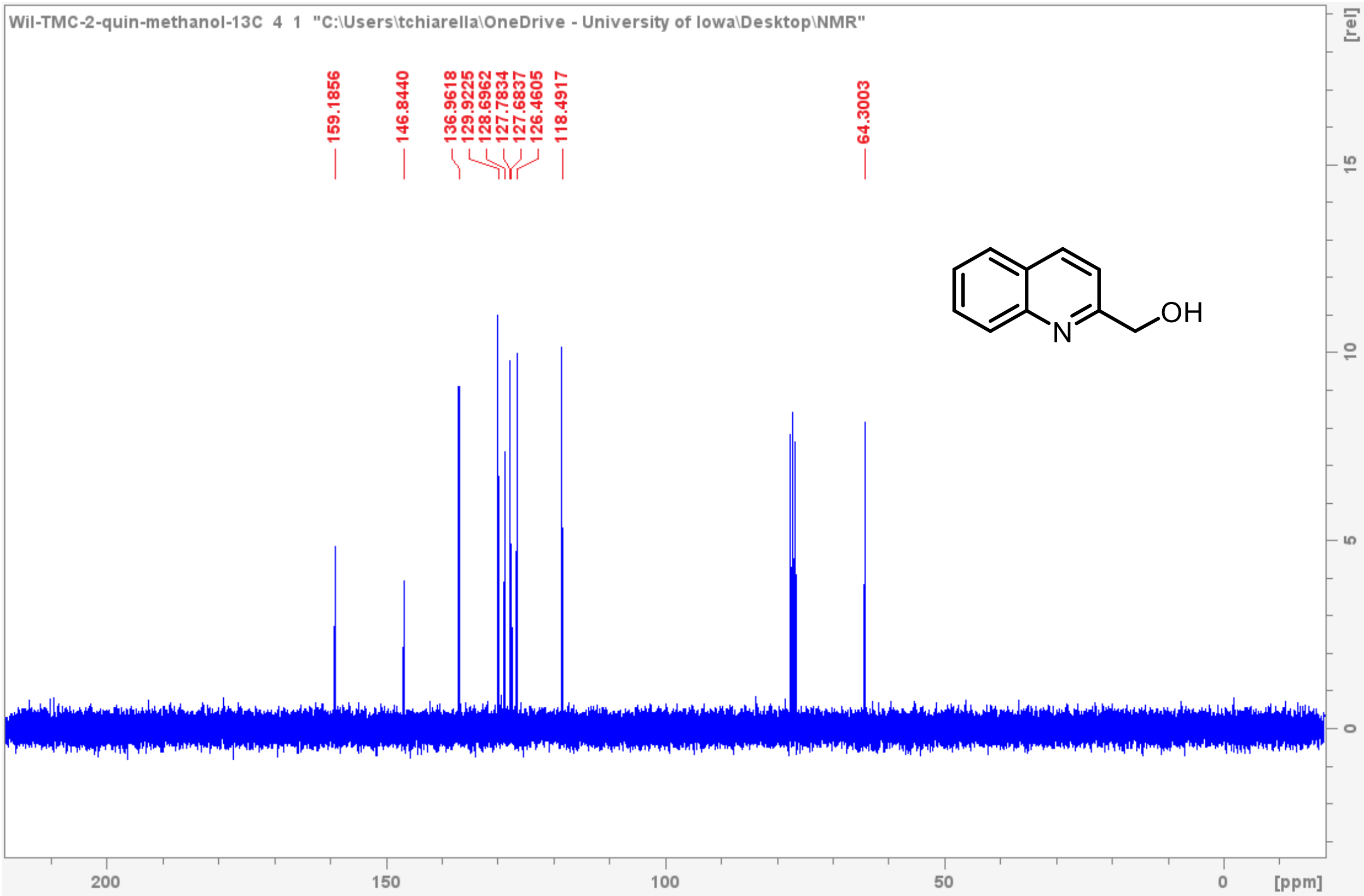

Figure S37: ^{1}H-NMR (400 MHz) of 4a in $CDCl_3$

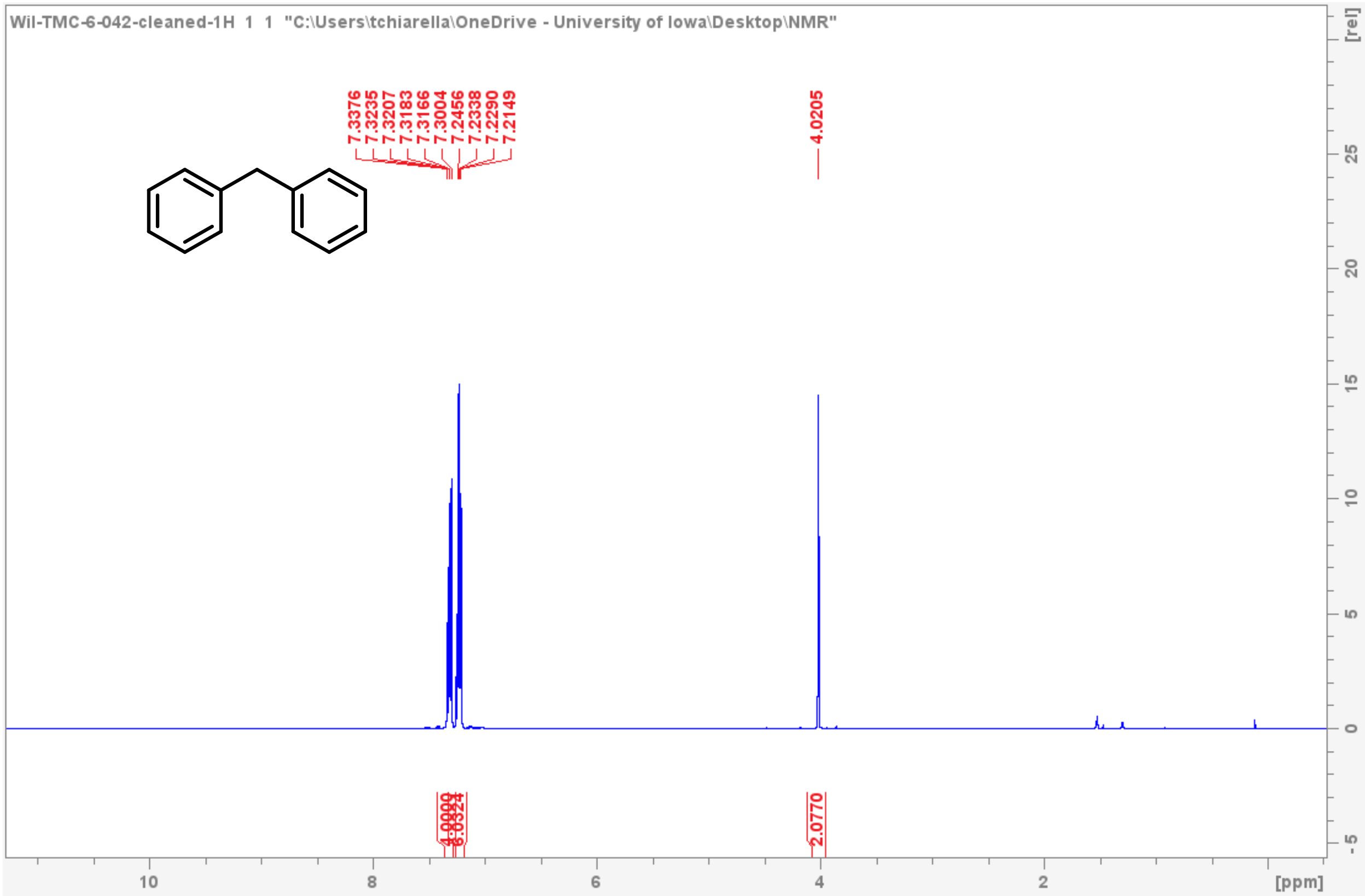

Figure S38: $^{13}C\{^{1}H\}$-NMR (100 MHz) of 4a in $CDCl_3$

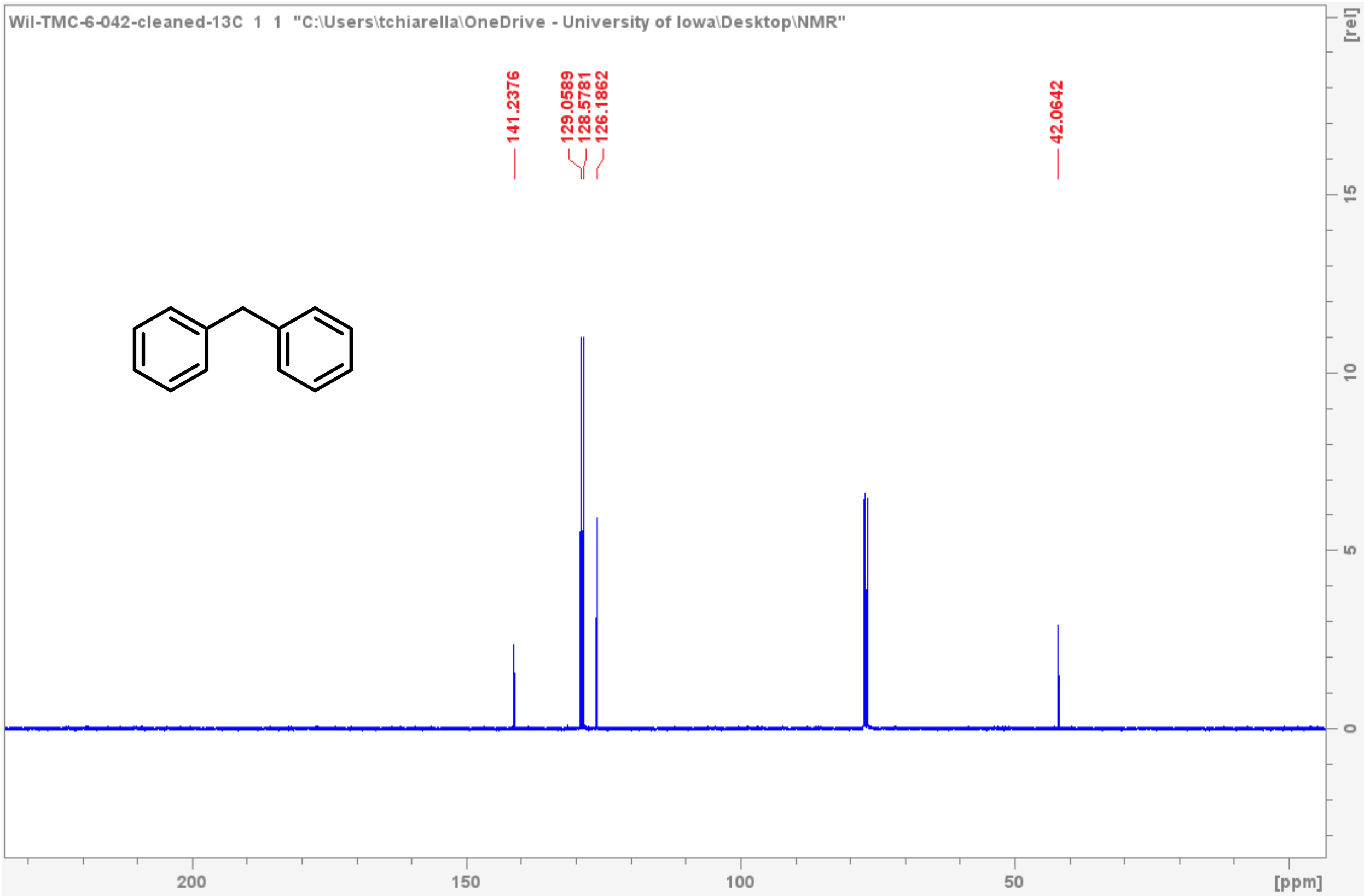

Figure S39: ^{1}H-NMR (400 MHz) of 4b in $CDCl_3$

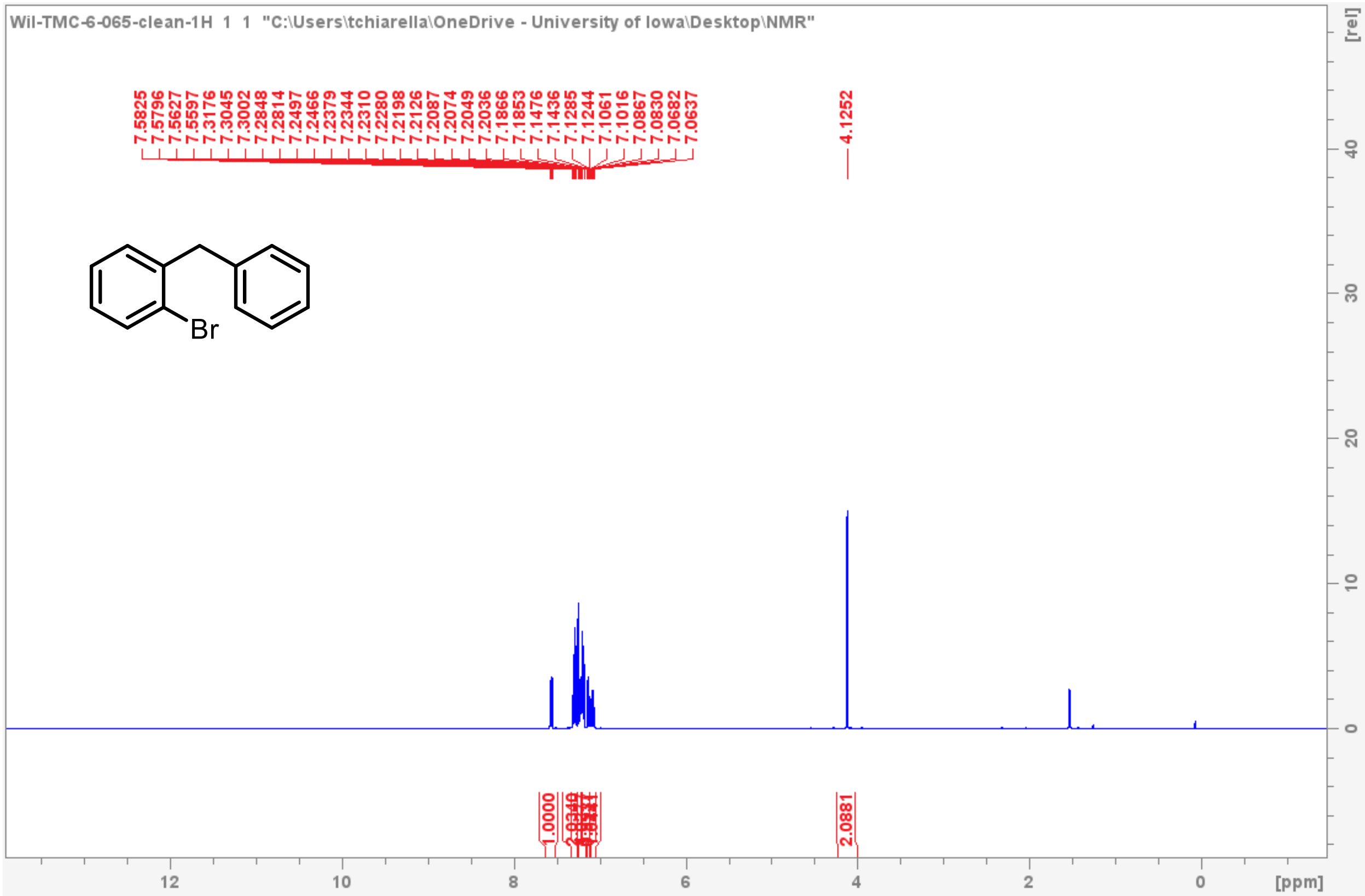

Figure S40: Zoomed in ^{1}H-NMR (400 MHz) of 4b in $CDCl_3$

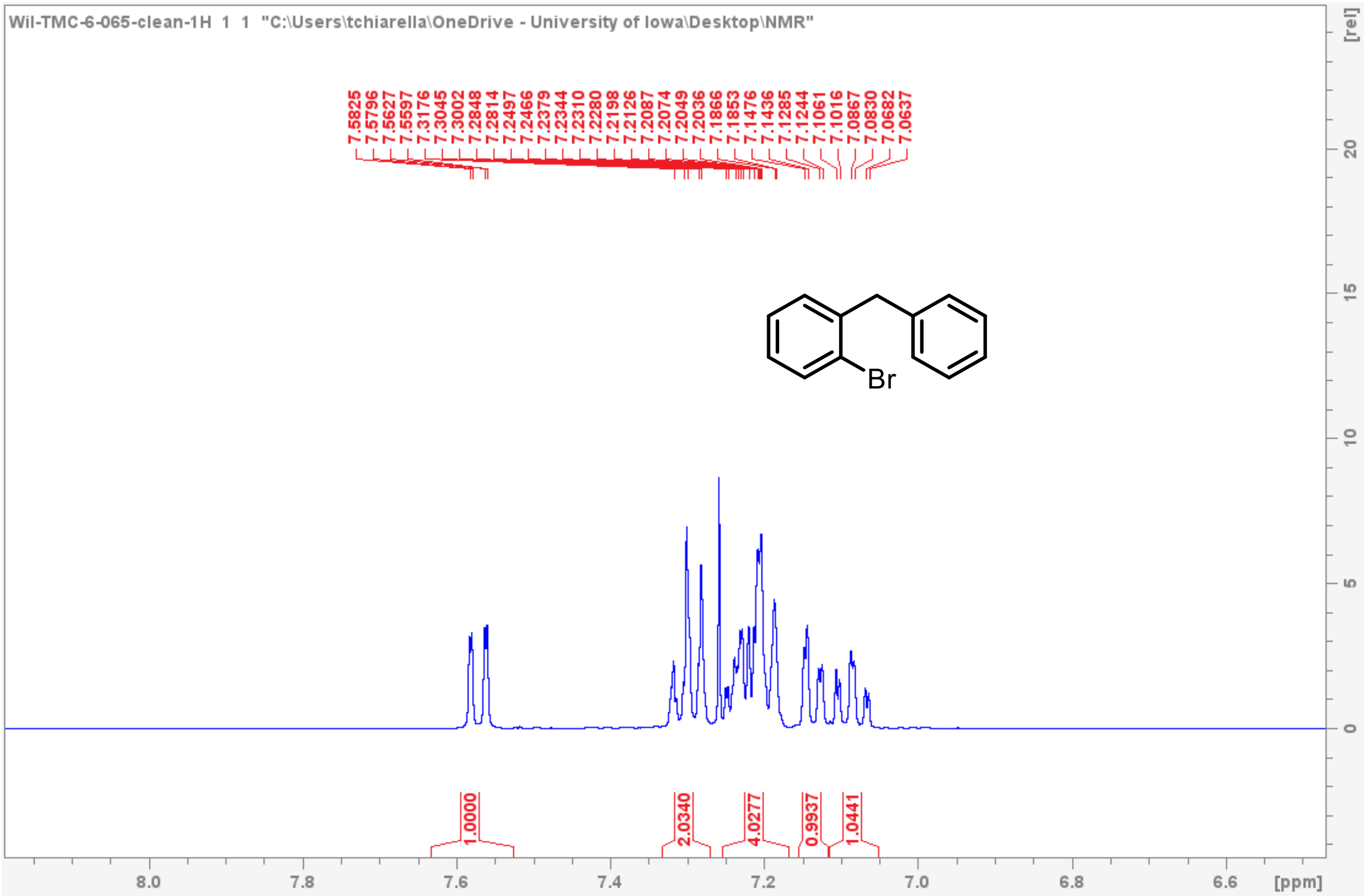

Figure S41: $^{13}C\{^{1}H\}$-NMR (100 MHz) of 4b in $CDCl_3$

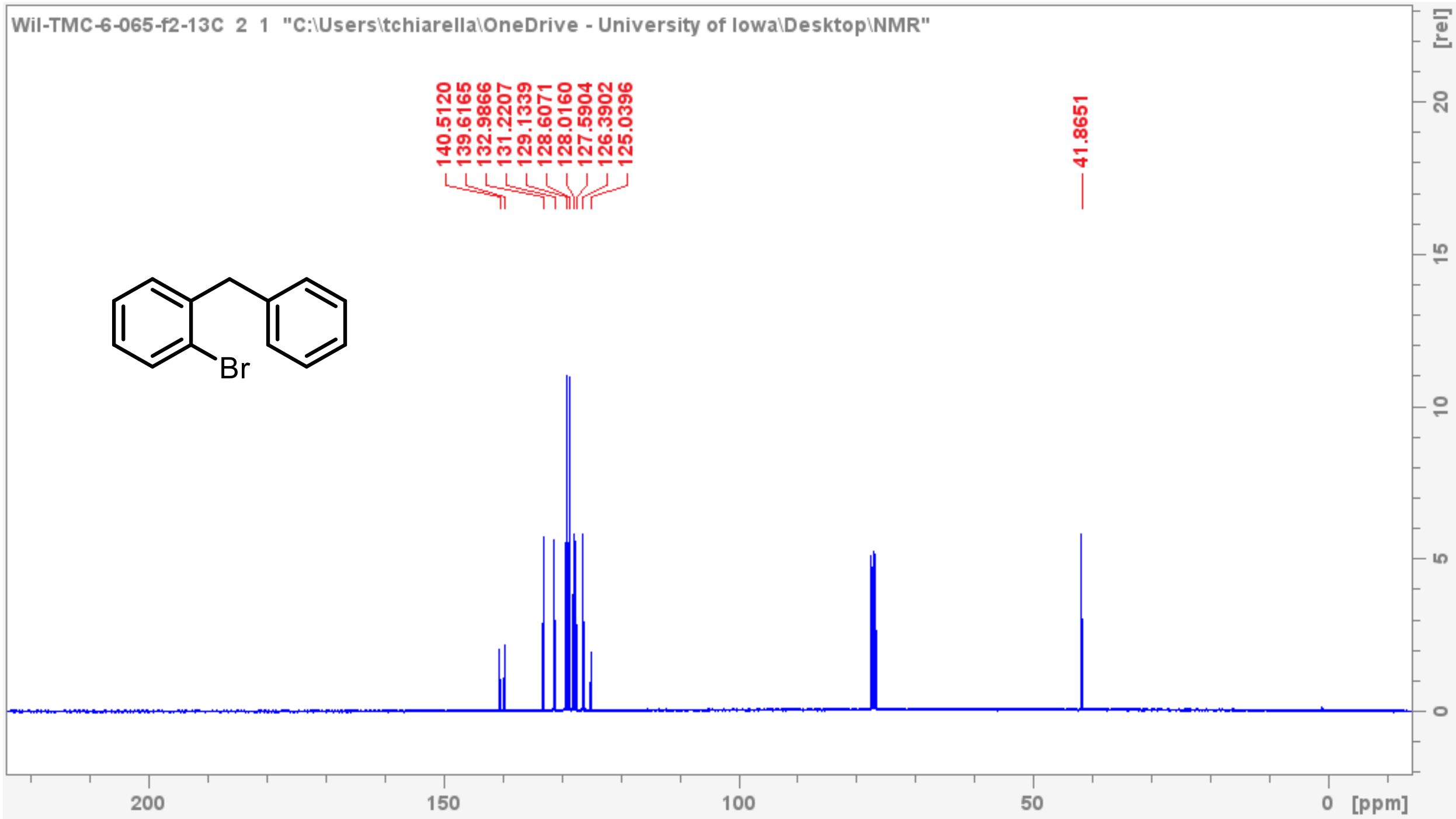

Figure S42: ^{1}H-NMR (400 MHz) of 4c in $CDCl_3$

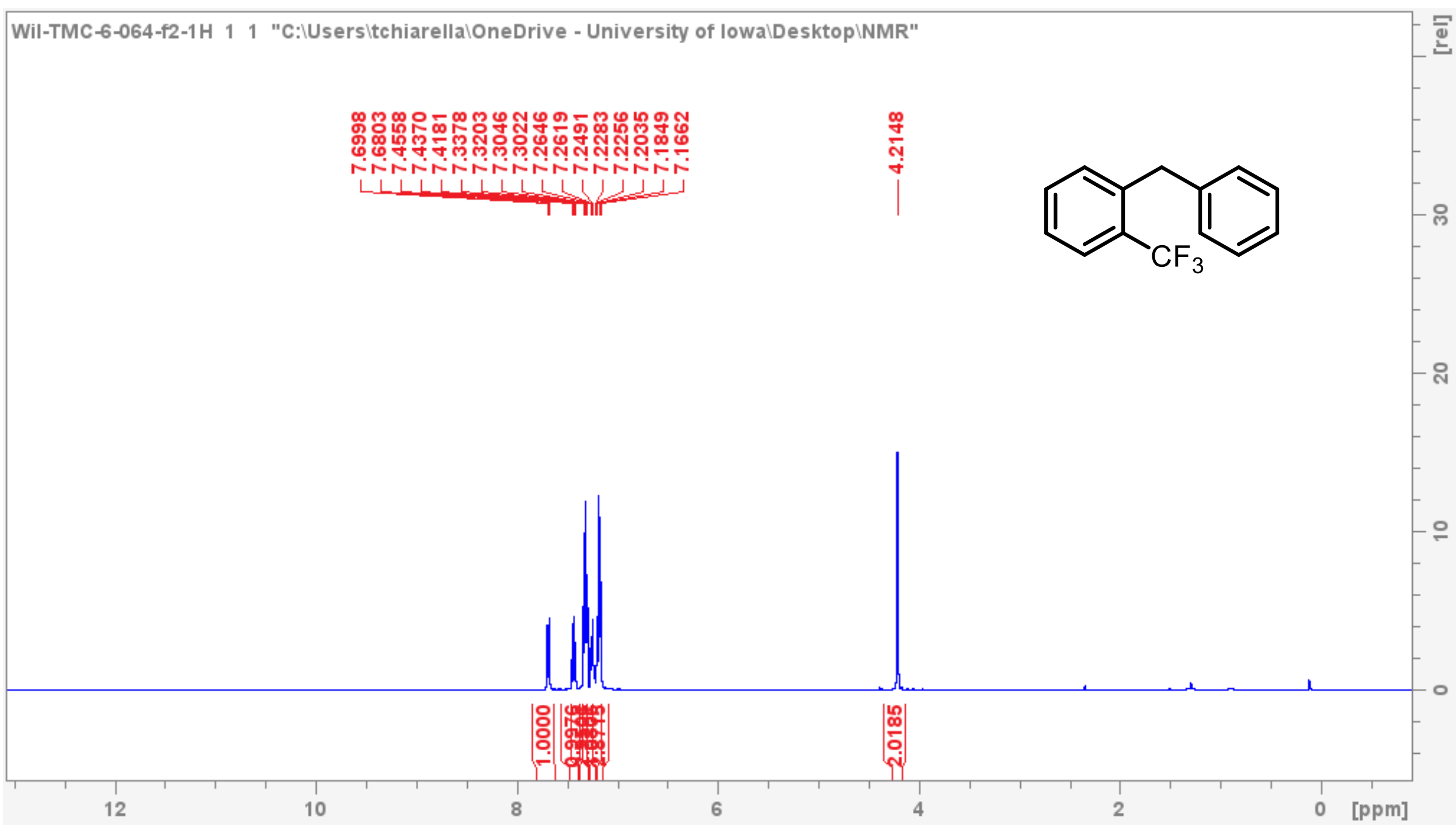

Figure S43: Zoomed in ^{1}H-NMR (400 MHz) of 4c in $CDCl_3$

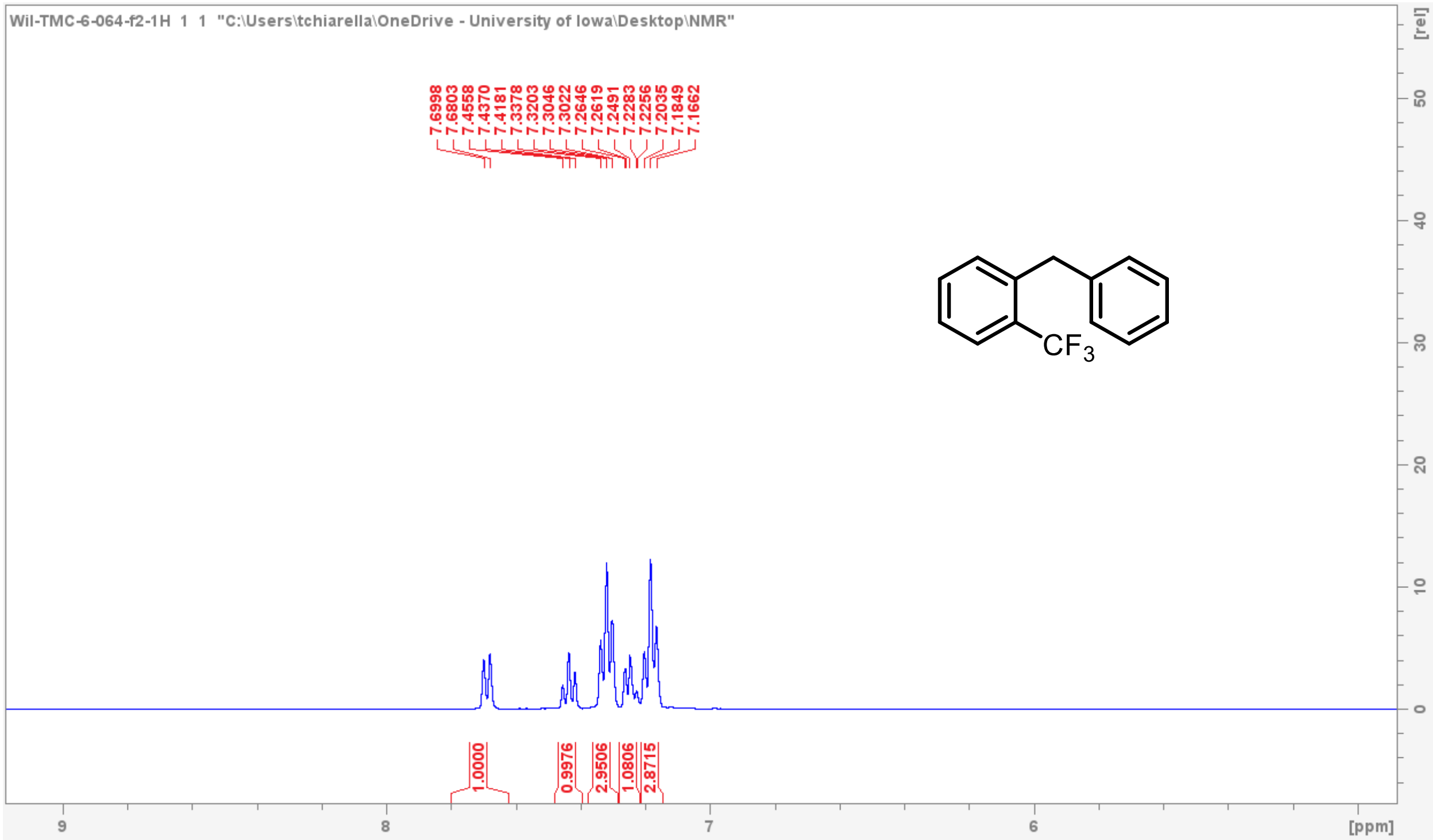

Figure S44: $^{13}C\{^{1}H\}$-NMR (100MHz) of 4c in $CDCl_3$

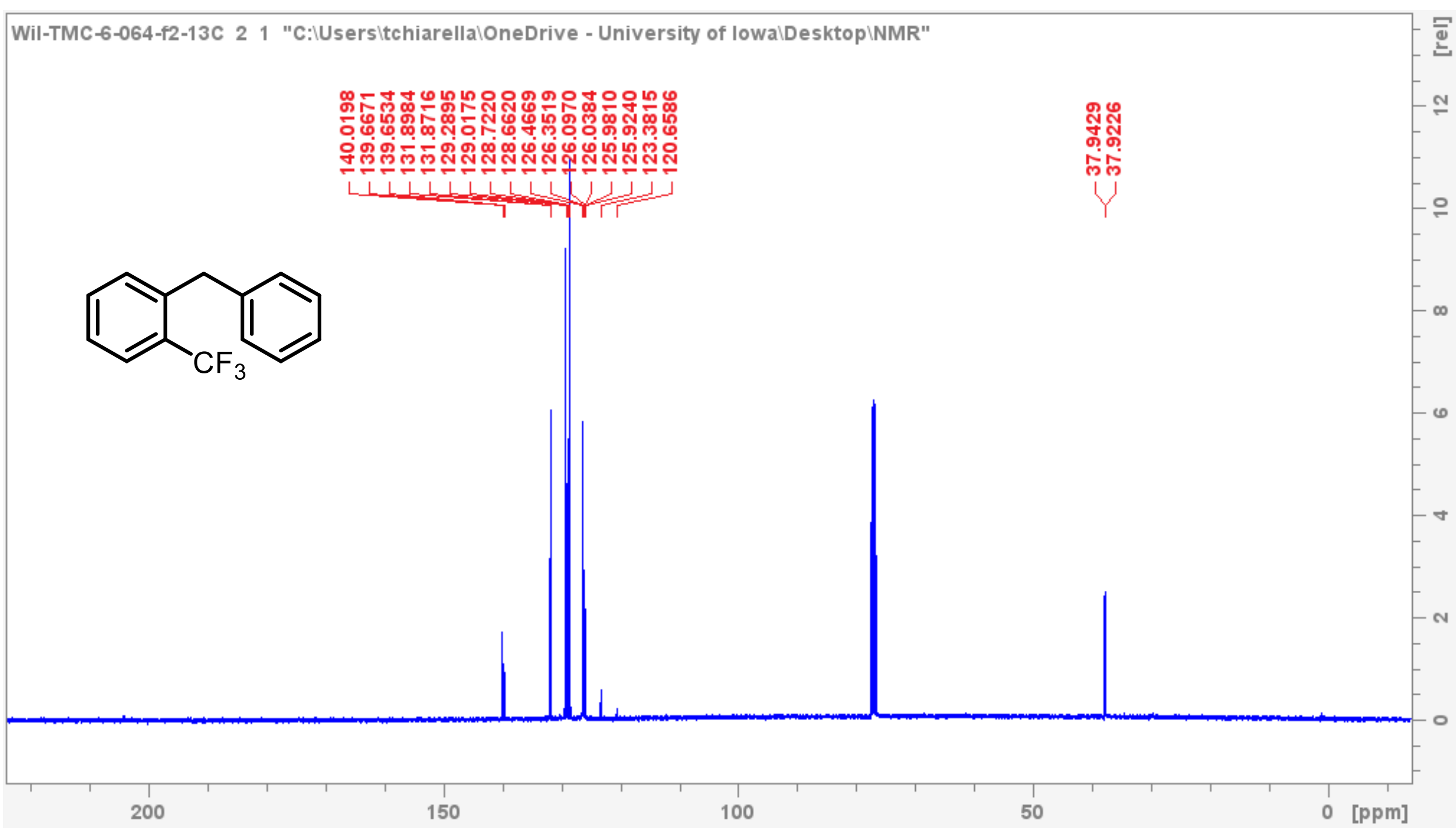

Figure S45: ^{19}F-NMR (377 MHz) of 4c in $CDCl_3$

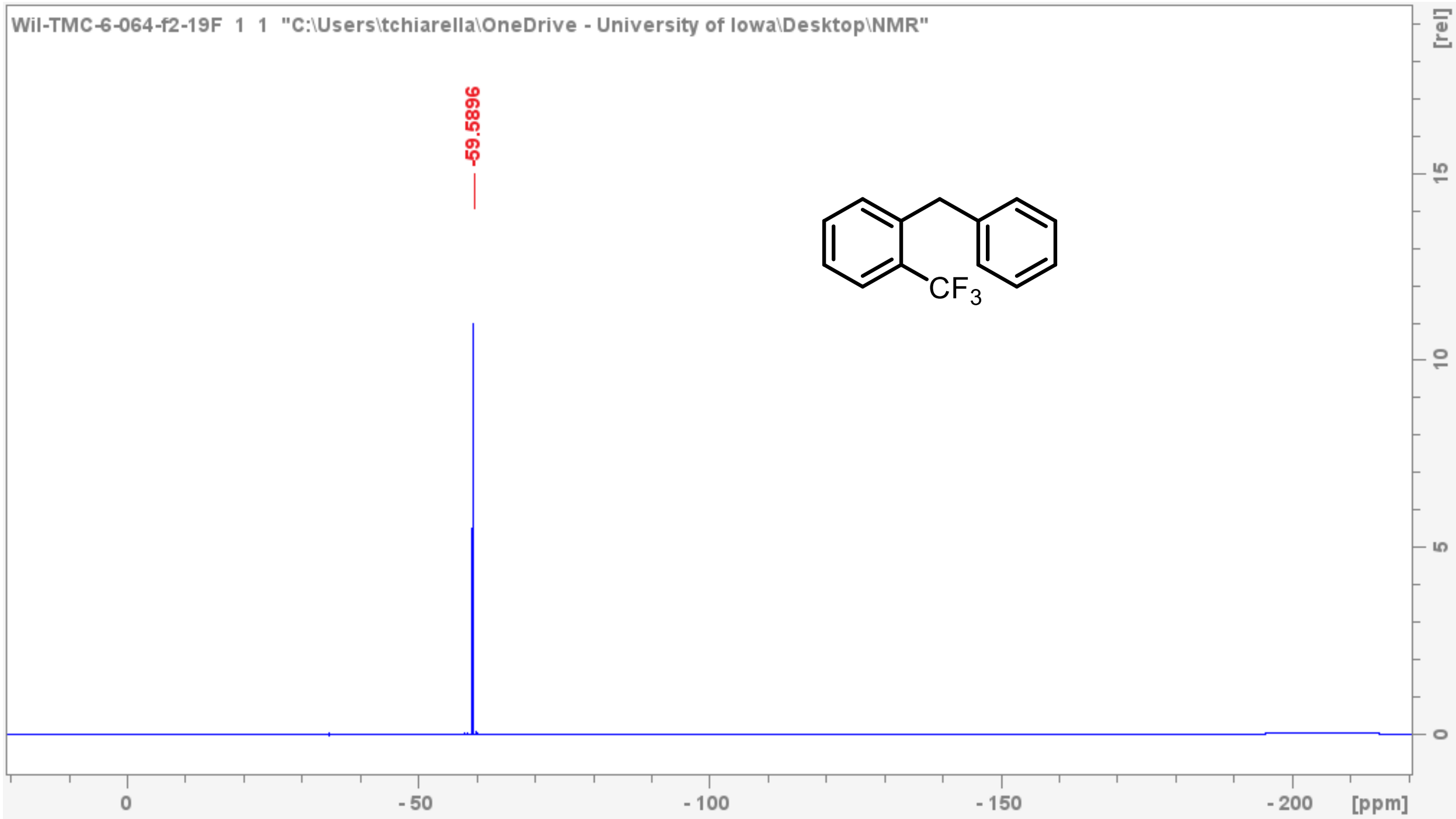

Figure S46: ^{1}H-NMR (300 MHz) of 4d in $CDCl_3$

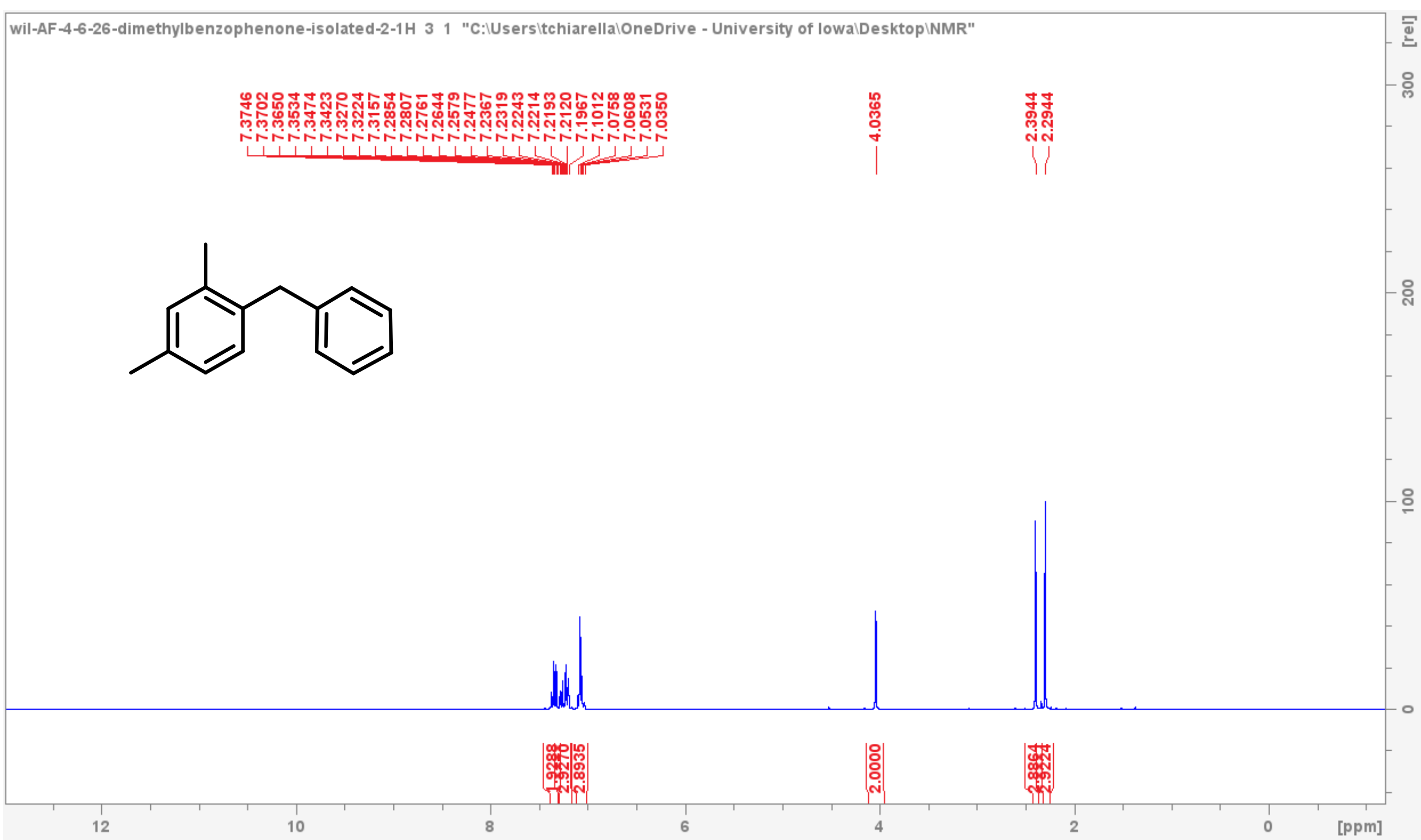

Figure S47: Zoomed in ^{1}H-NMR (300 MHz) of 4d in $CDCl_3$

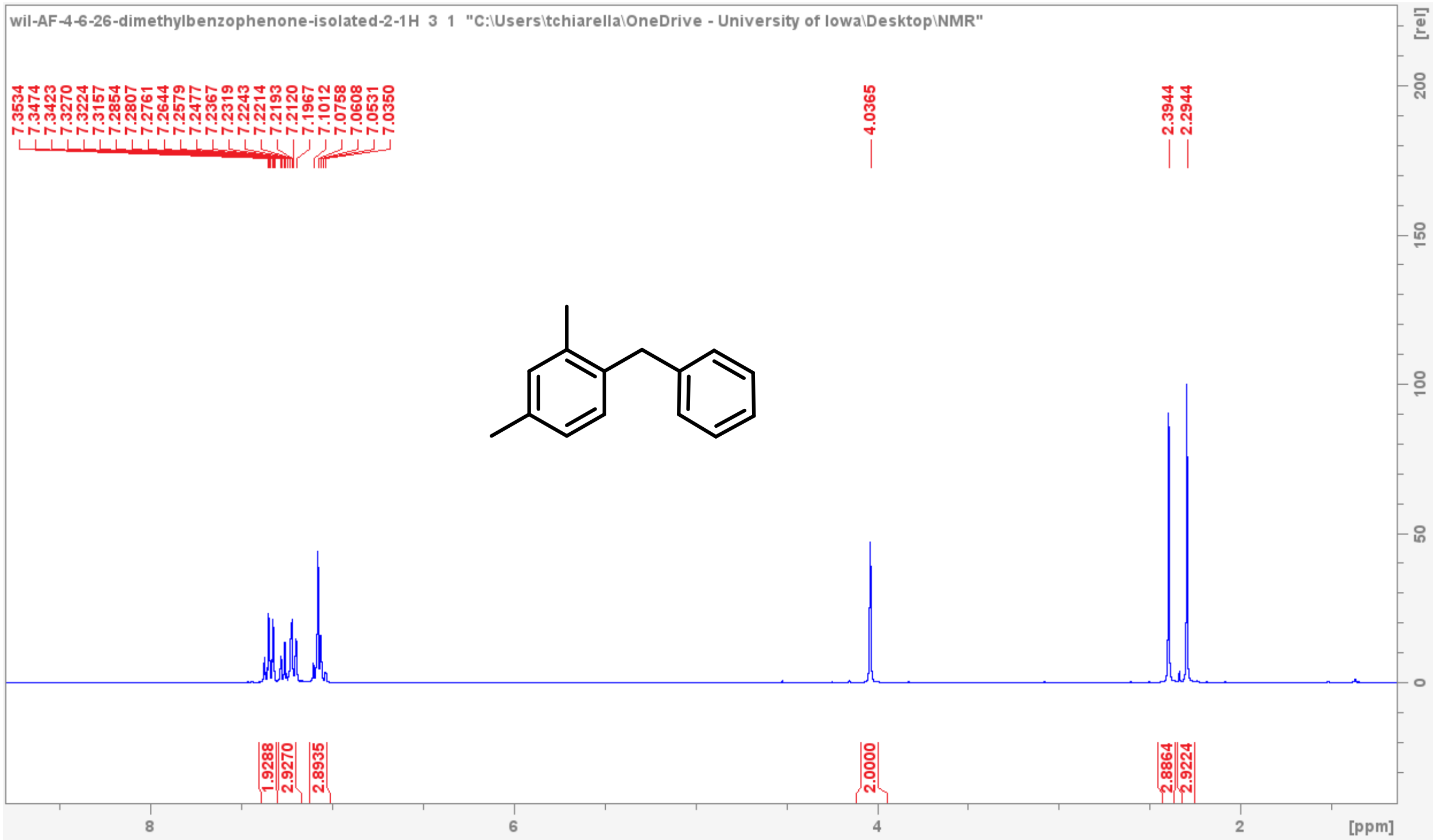

Figure S48: $^{13}C\{^{1}H\}$-NMR (75 MHz) of 4d in $CDCl_3$

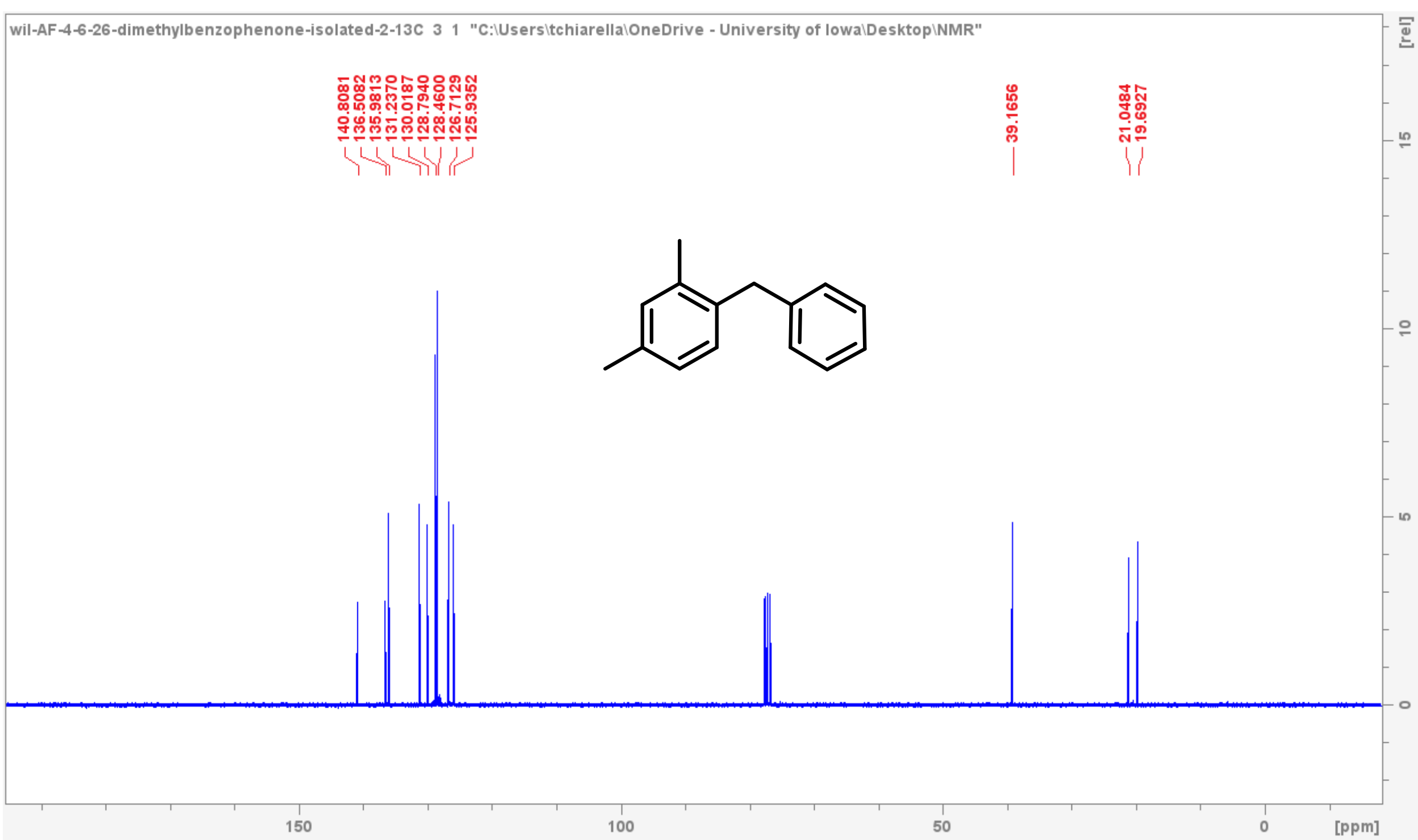

Figure S49: ^{1}H-NMR (400 MHz) of 4e in $CDCl_3$

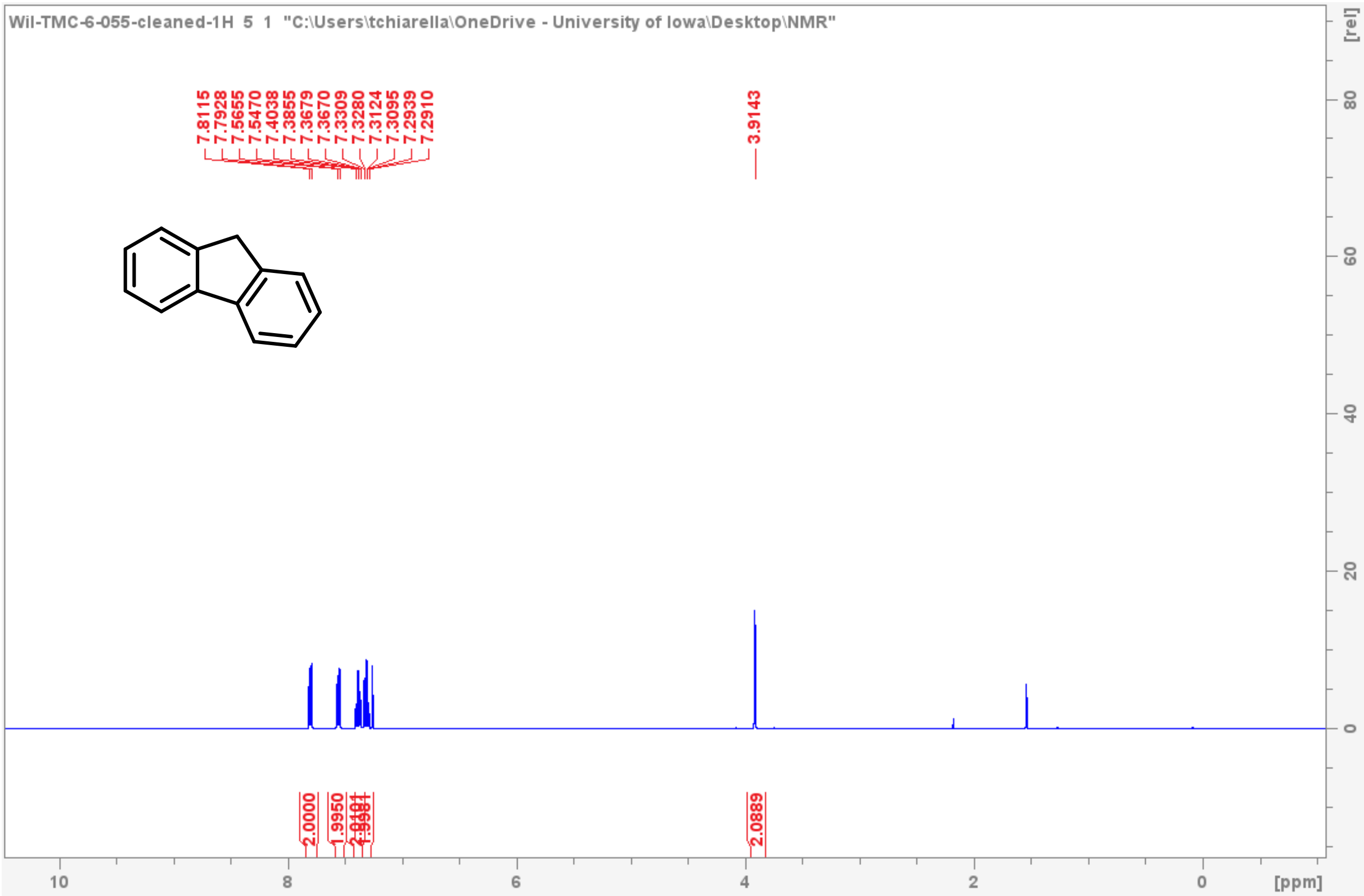

Figure S50: Zoomed in ^{1}H-NMR (400 MHz) of 4e in $CDCl_3$

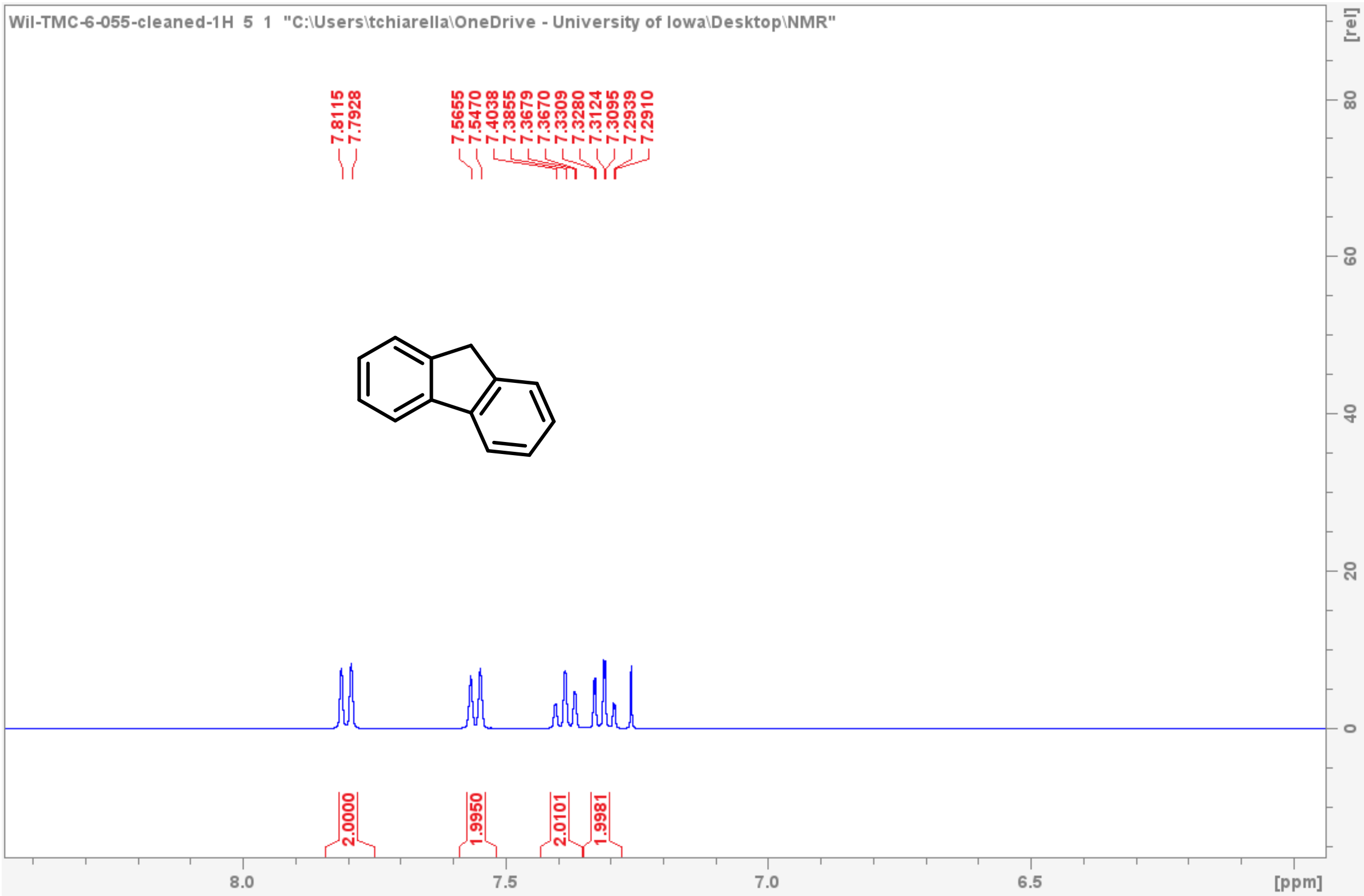

Figure S51: $^{13}C\{^{1}H\}$-NMR (100 MHz) of 4e in $CDCl_3$

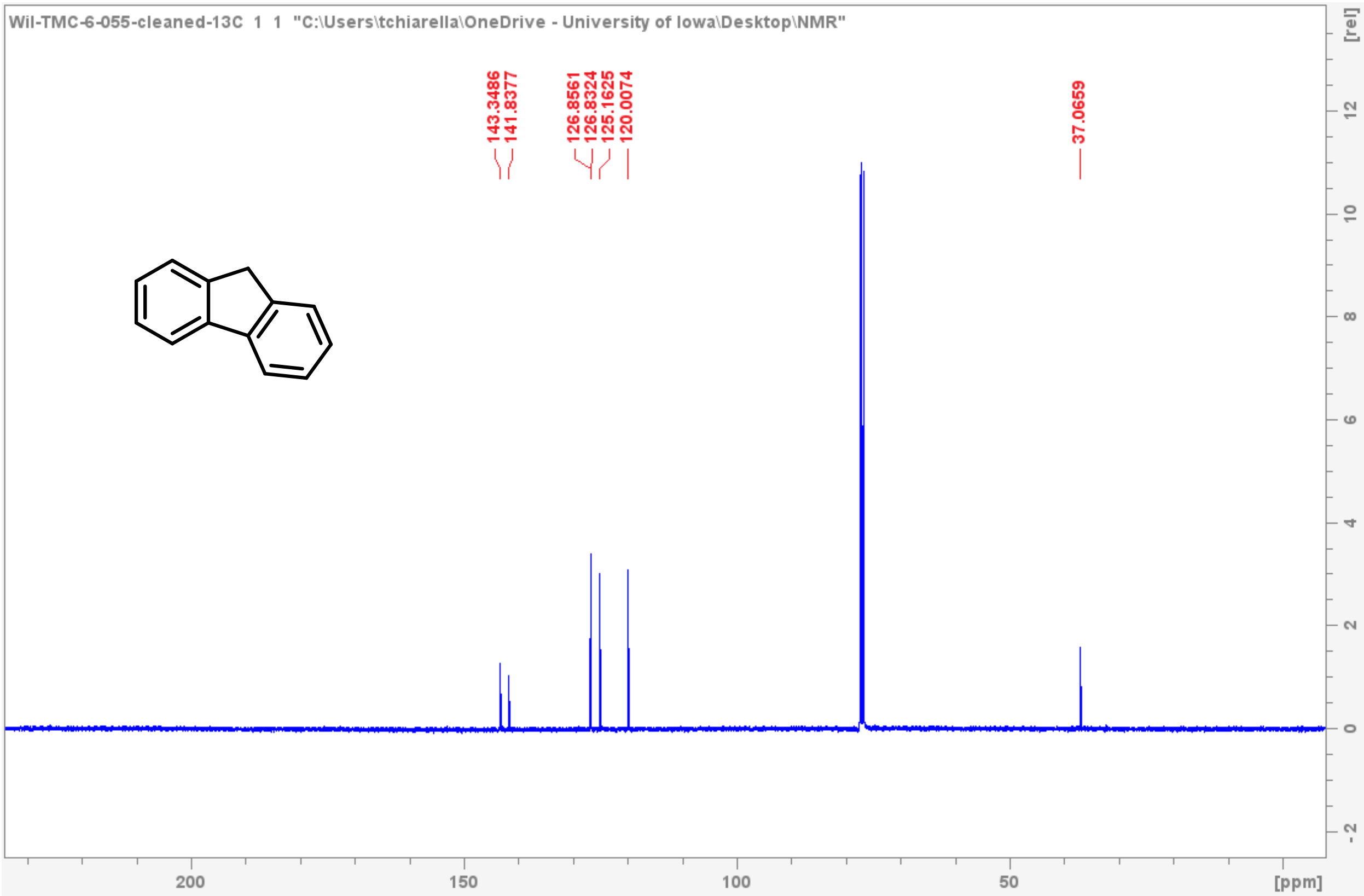

Figure S52: ^{1}H-NMR (300 MHz) of 4e' in $CDCl_3$

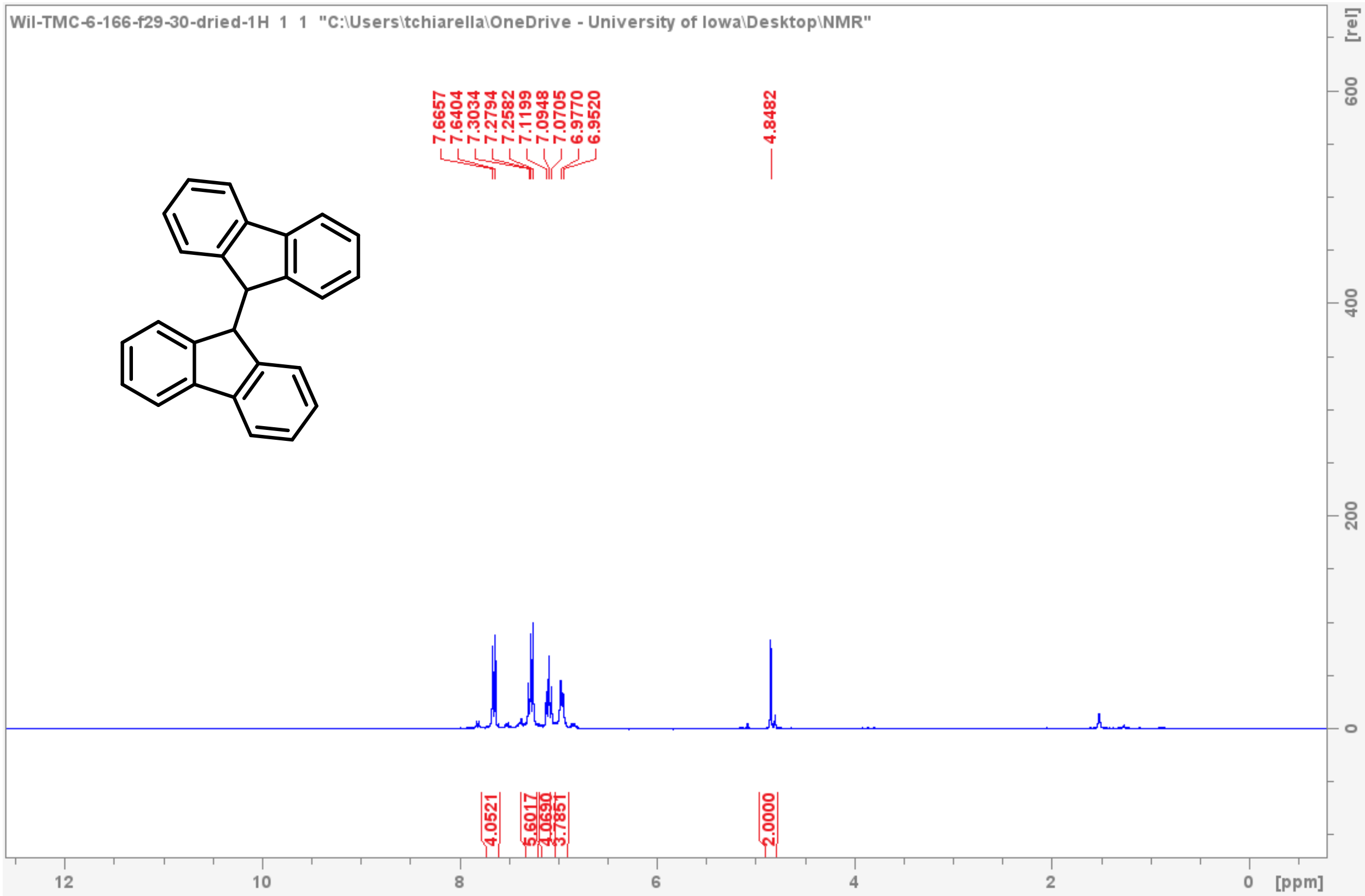

Figure S53: $^{13}C\{^{1}H\}$-NMR (75 MHz) of 4e' in $CDCl_3$

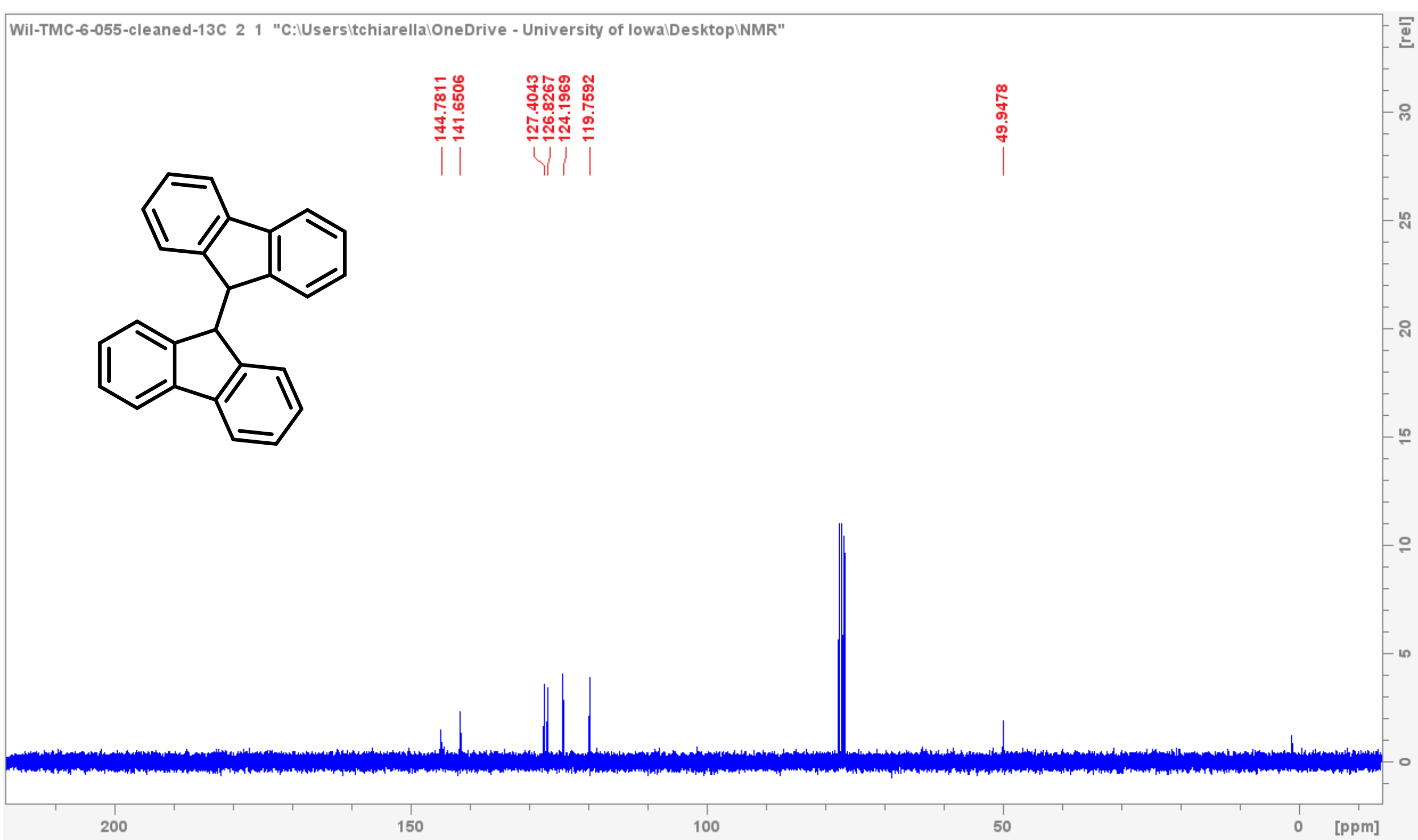

Figure S54: ^{1}H-NMR (300 MHz) of 4f in $CDCl_3$

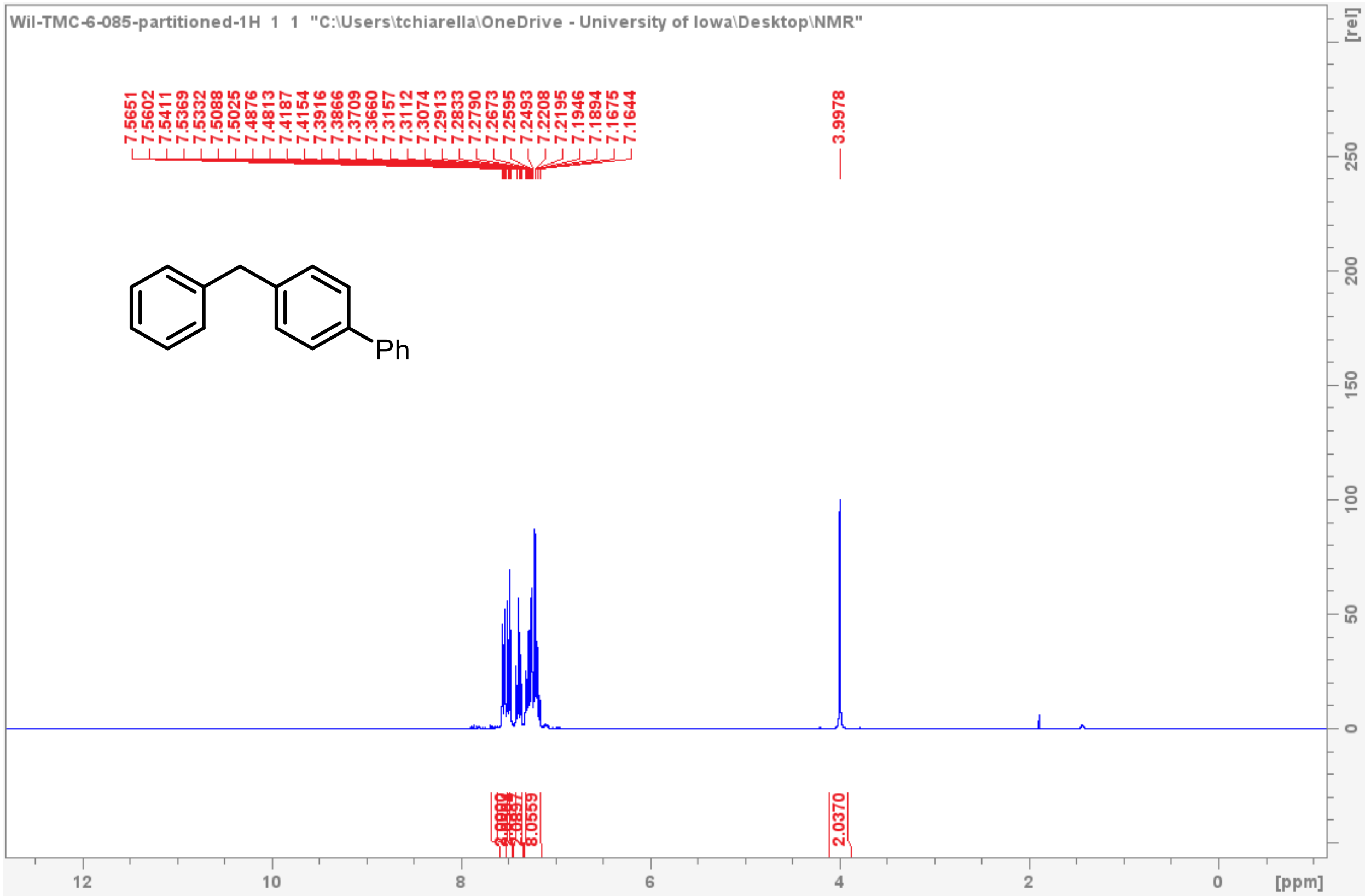

Figure S55: Zoomed in ^{1}H-NMR (300 MHz) of 4f in $CDCl_3$

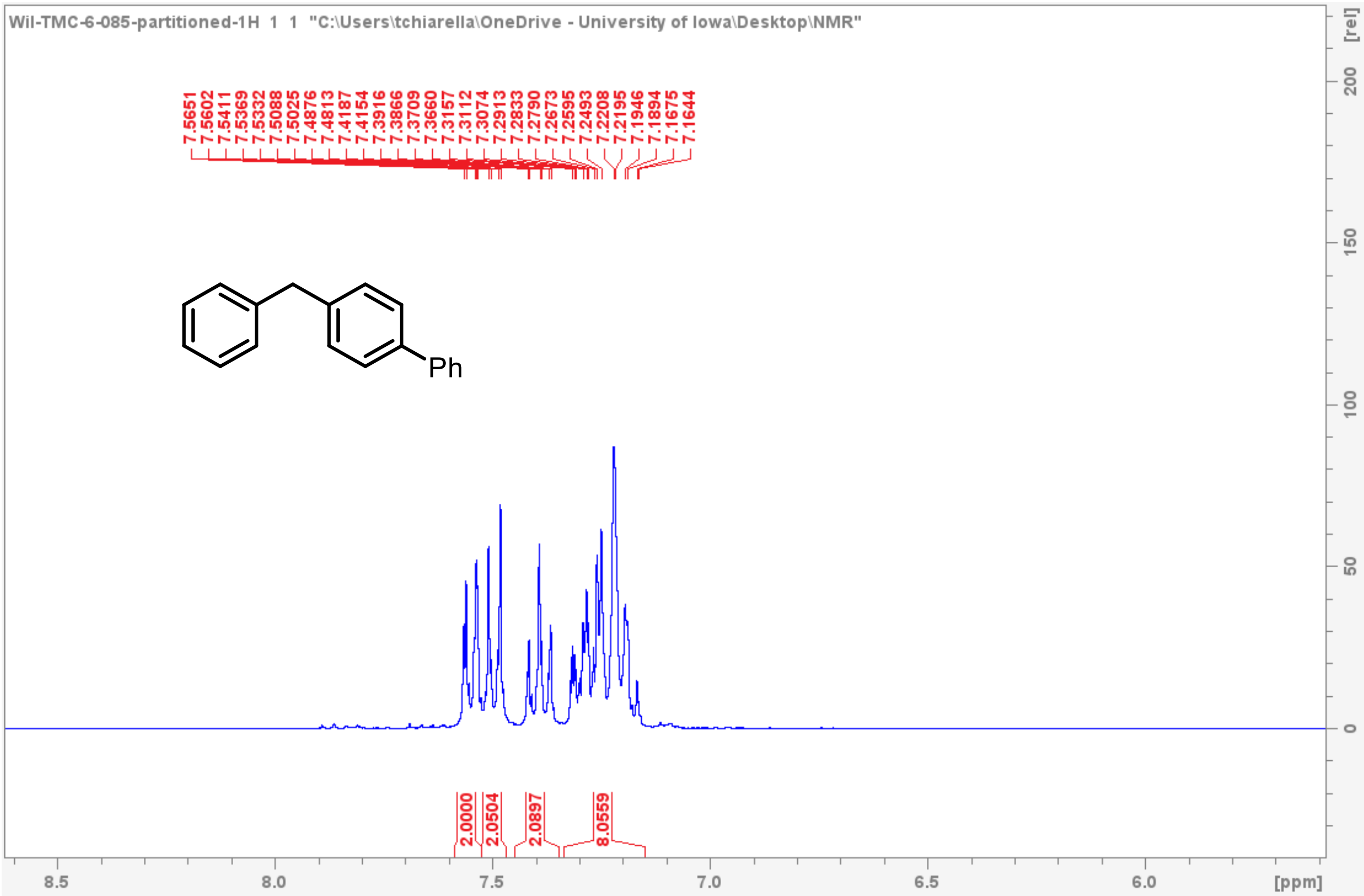

Figure S56: $^{13}C\{^{1}H\}$-NMR (75 MHz) of 4f in $CDCl_3$

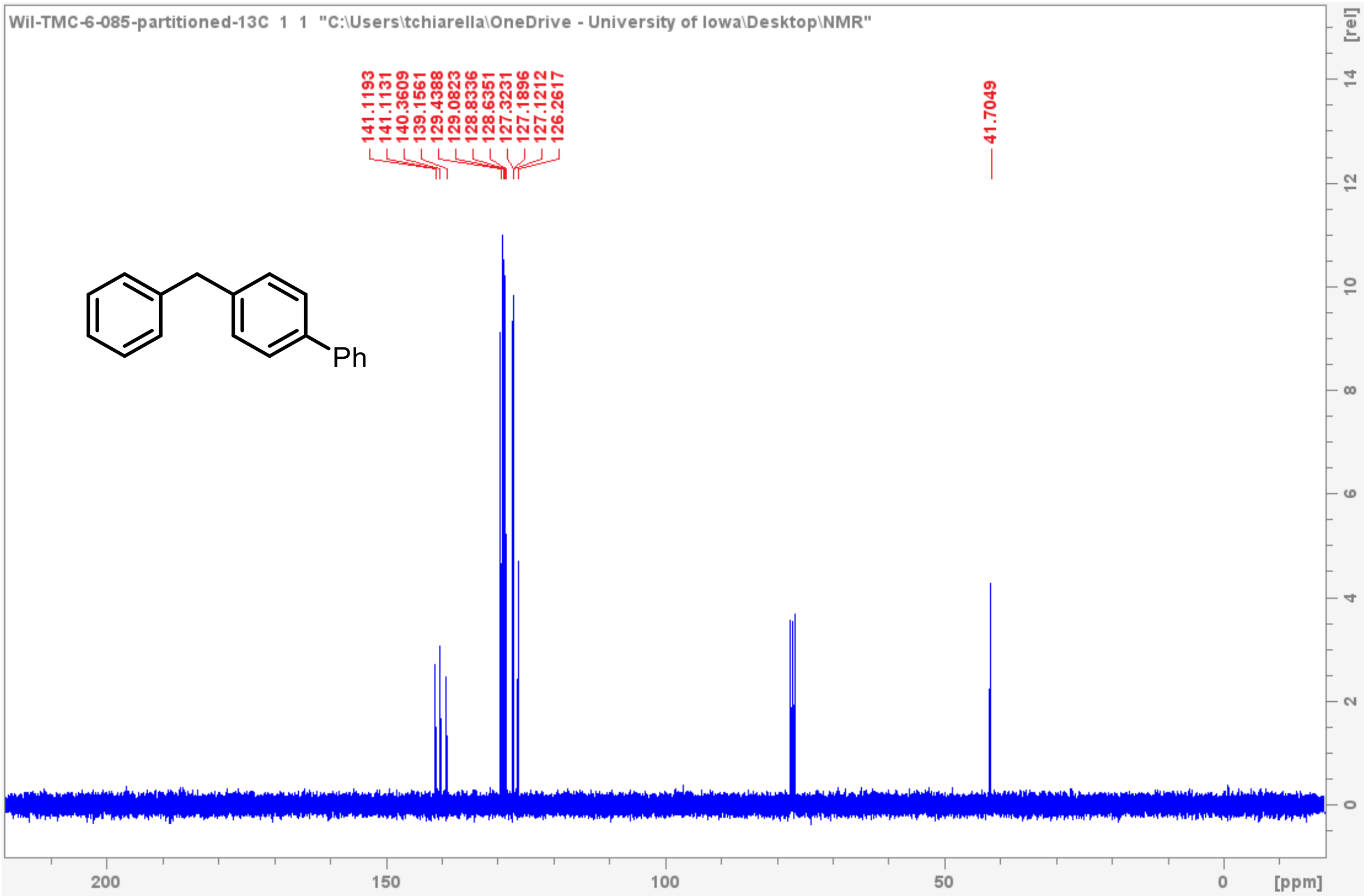

Figure S57: ^{1}H-NMR (400 MHz) of 4g in $CDCl_3$

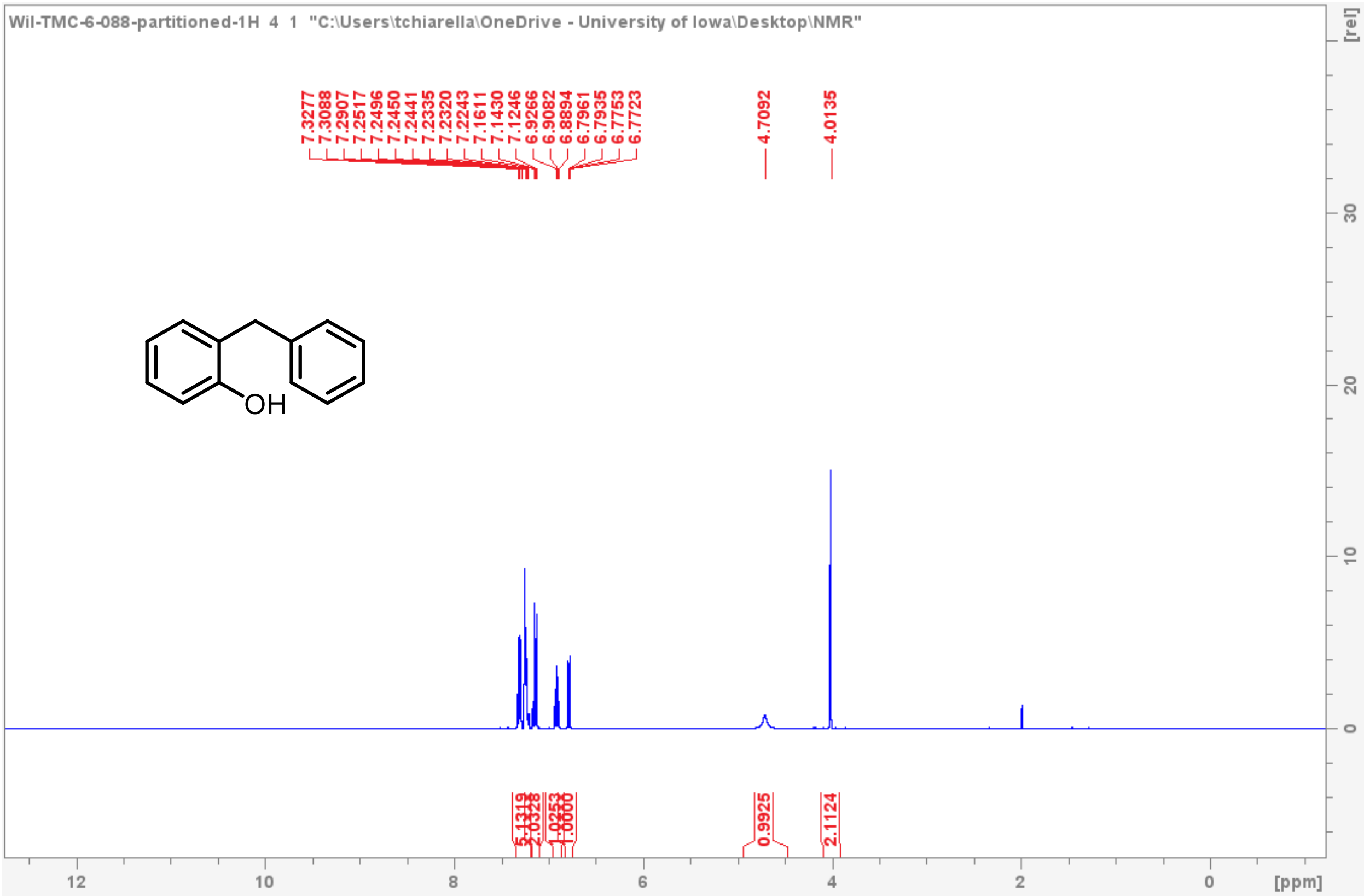

Figure S58: $^{13}C\{^{1}H\}$-NMR (100 MHz) of 4g in $CDCl_3$

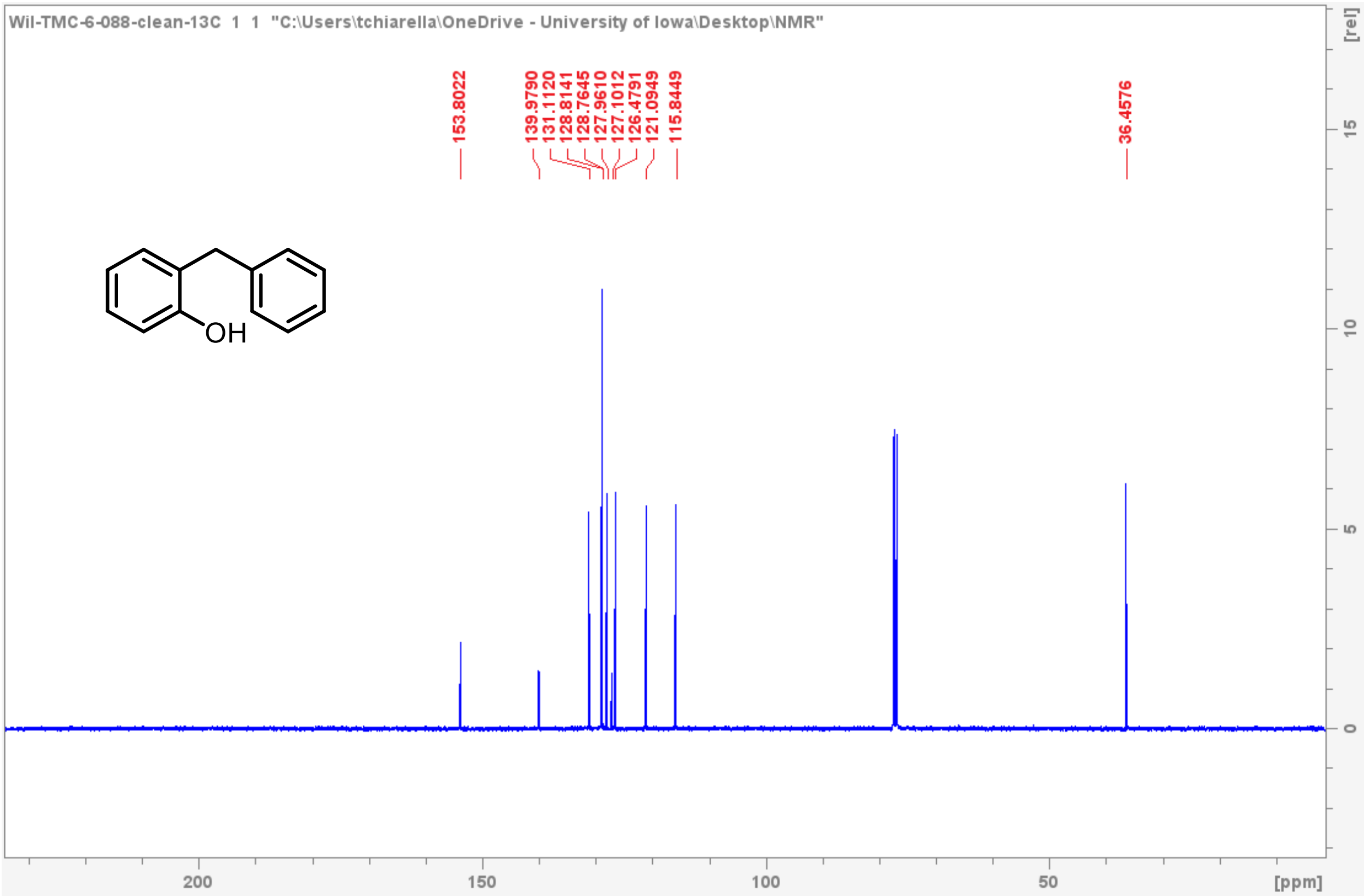

Figure S59: ^{1}H-NMR (300 MHz) of 4h in $CDCl_3$

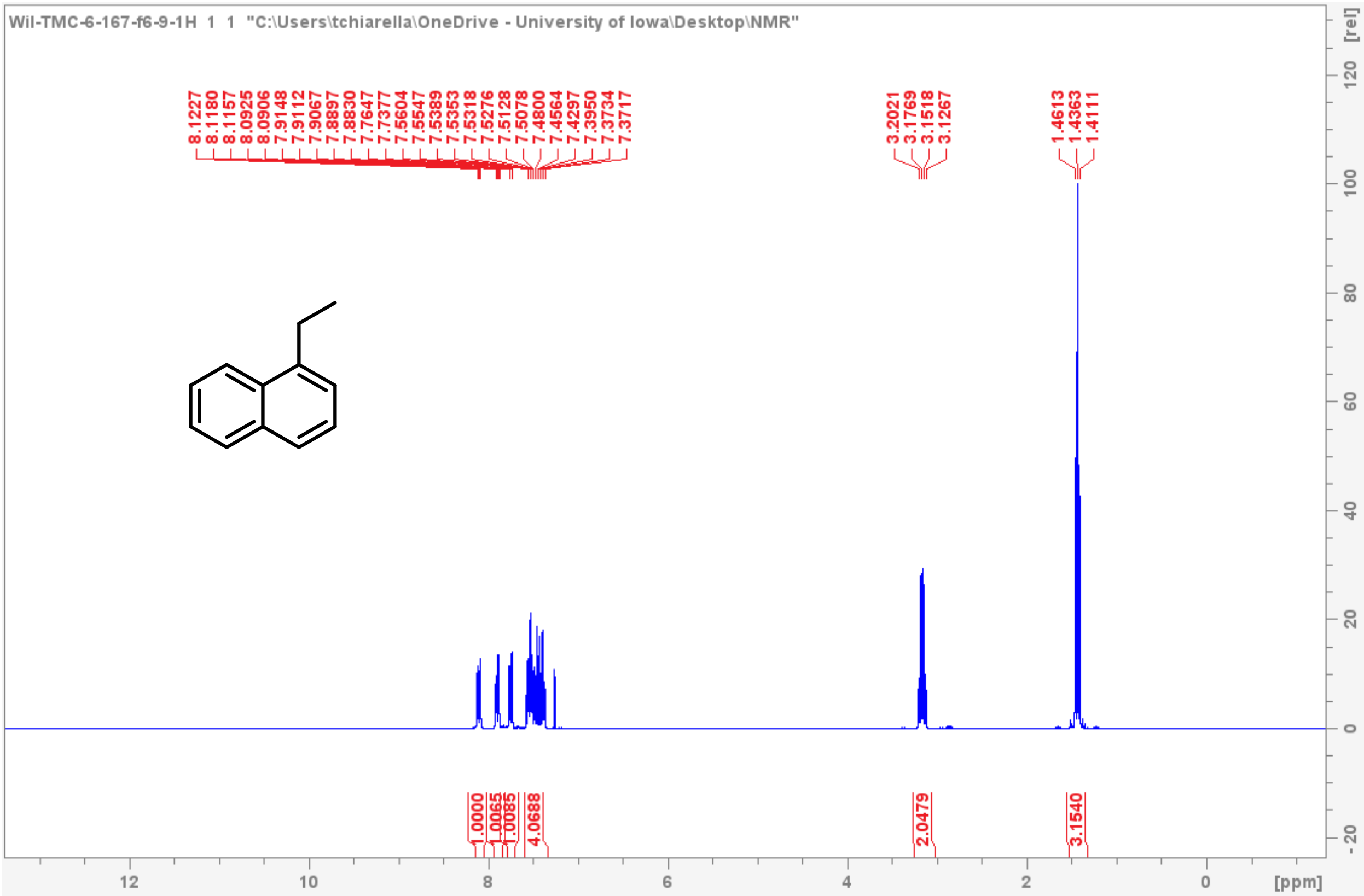

Figure S60: $^{13}C\{^{1}H\}$-NMR (100 MHz) of 4h in $CDCl_3$

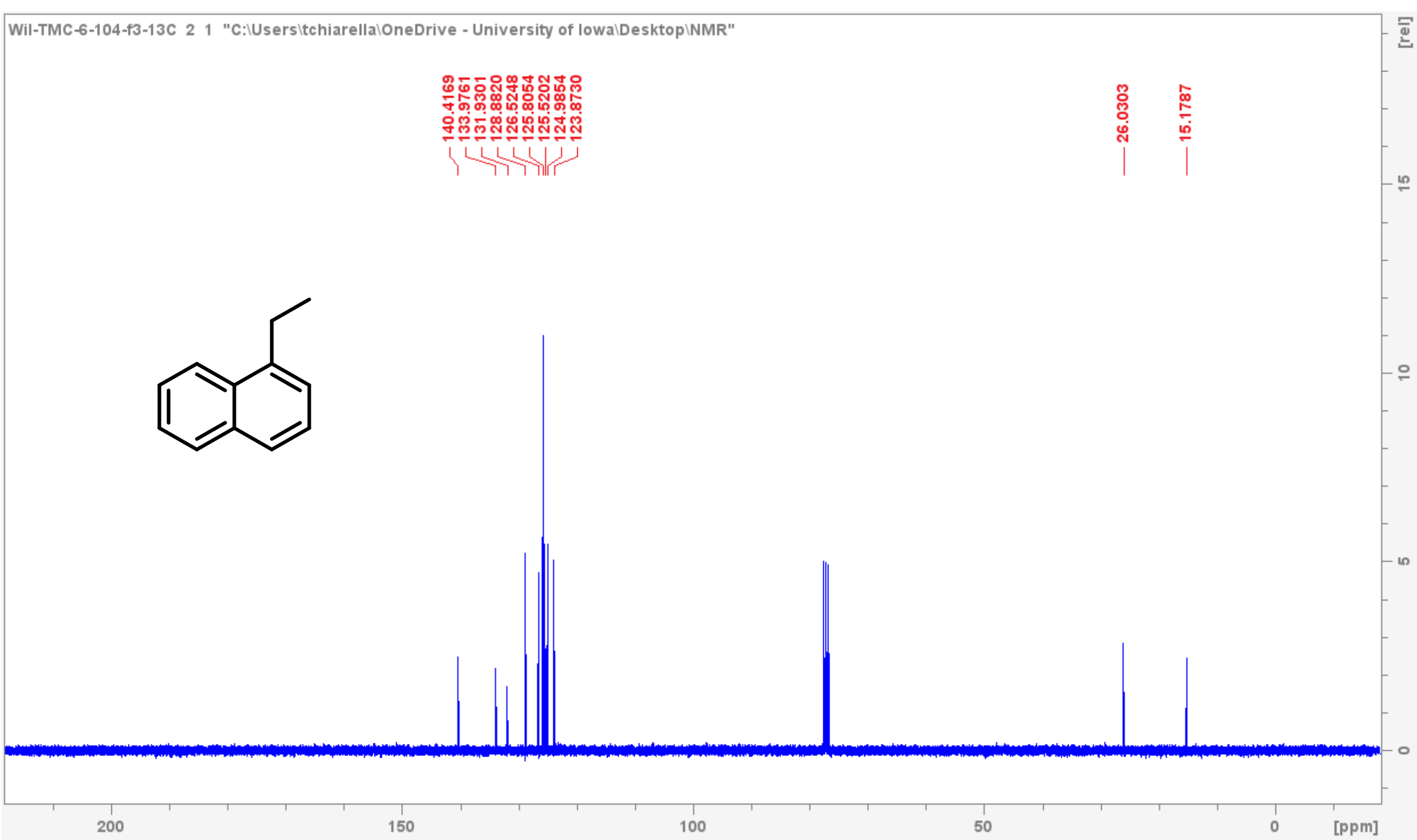

Figure S61: ^{1}H-NMR (400 MHz) of 4i in $CDCl_3$

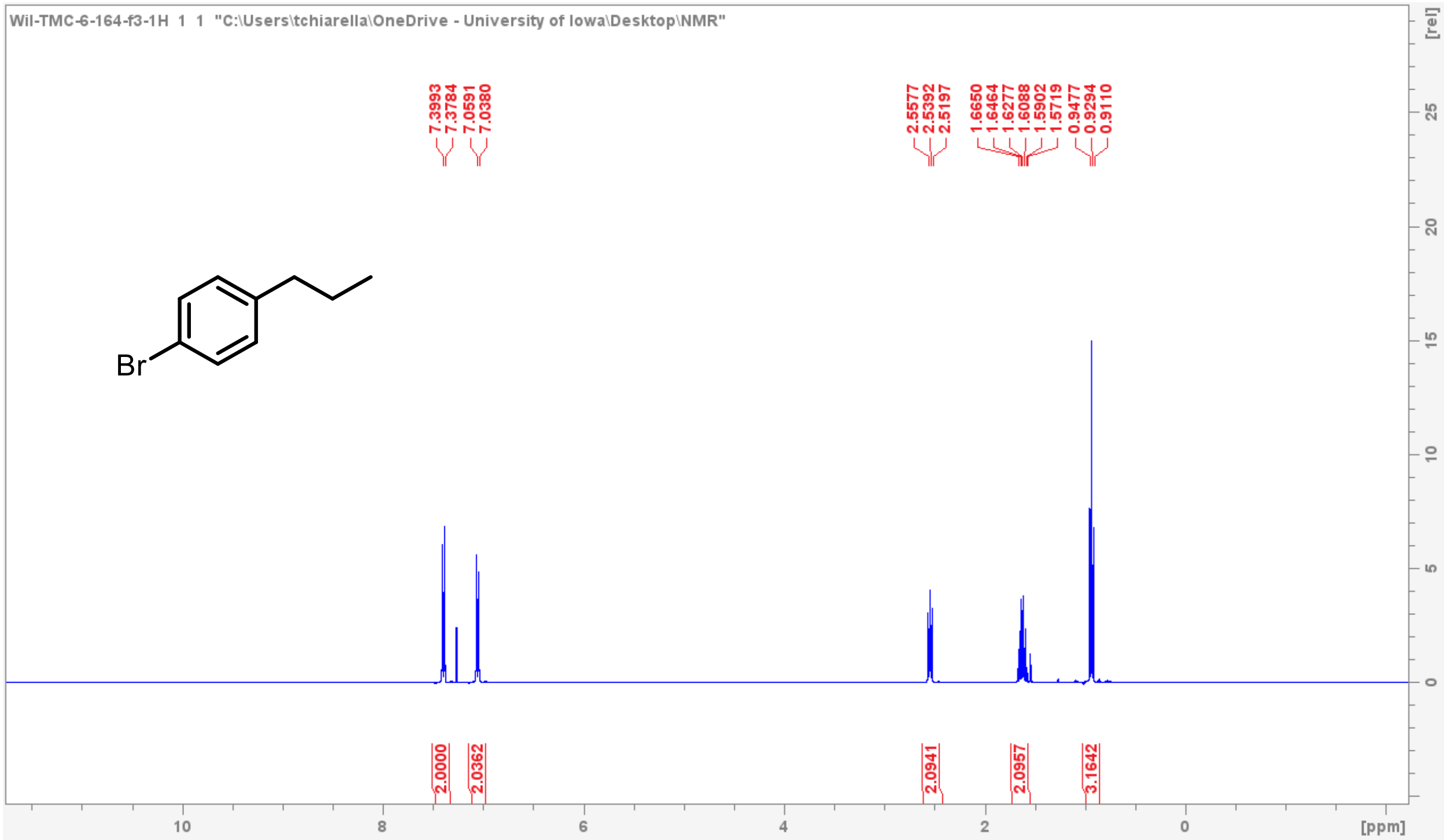

Figure S62: ^{13}C{^{1}H}-NMR (100 MHz) of 4i in $CDCl_3$

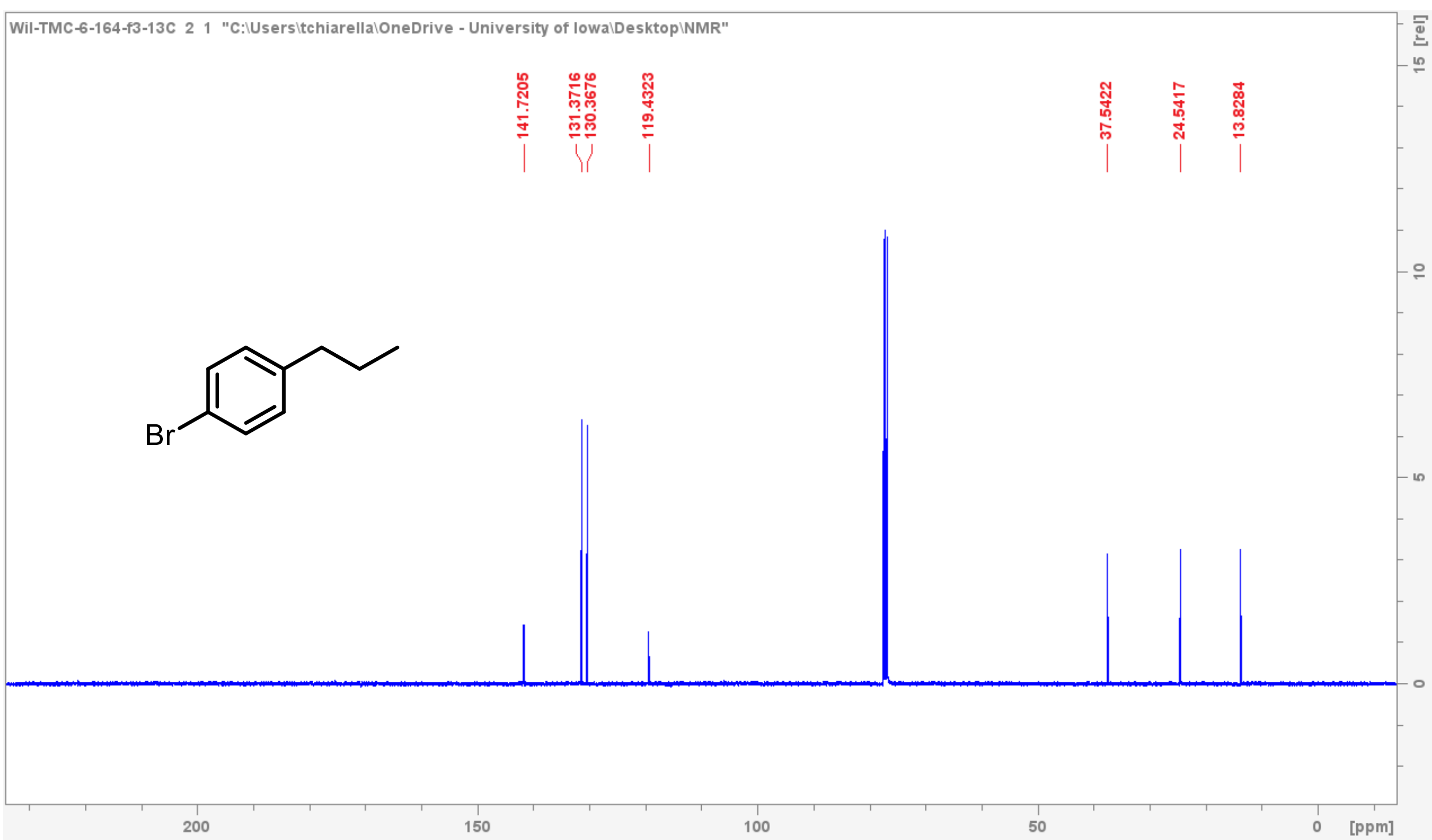

Figure S63: ^{1}H-NMR (300 MHz) of 4j in $CDCl_3$

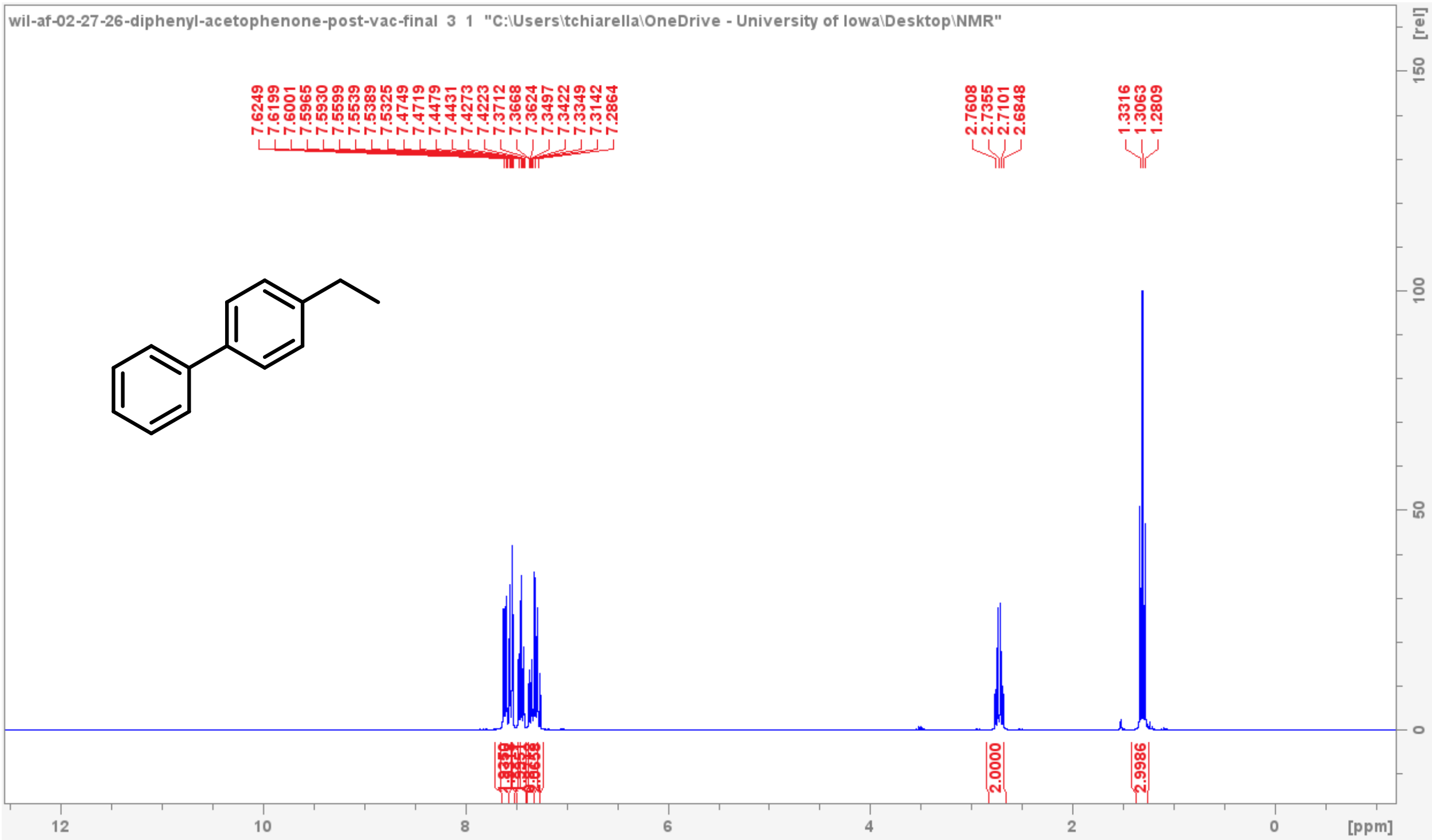

Figure S64: Zoomed in ^{1}H-NMR (300 MHz) of 4j in $CDCl_3$

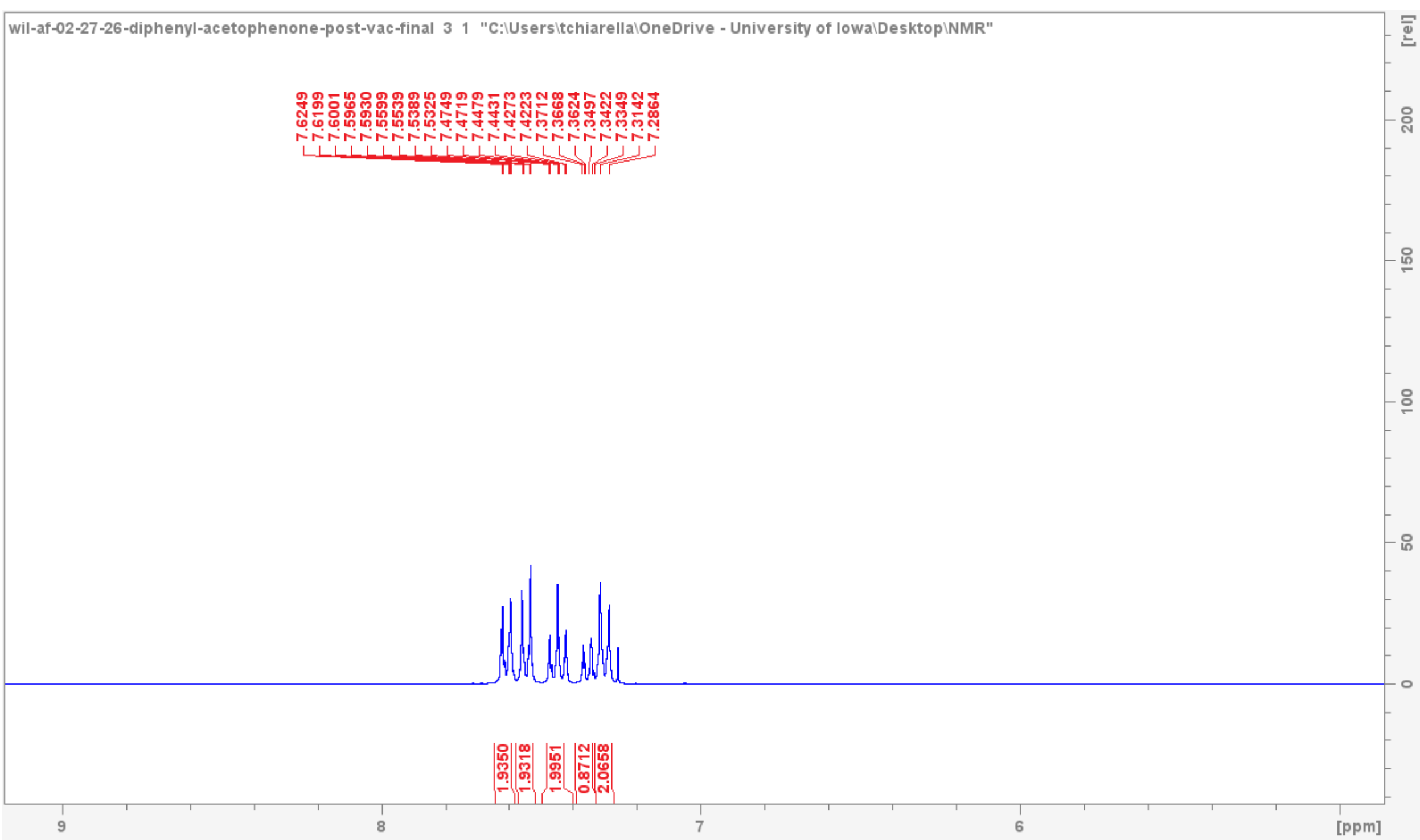

Figure S65: $^{13}C\{^{1}H\}$-NMR (75 MHz) of 4j in $CDCl_3$

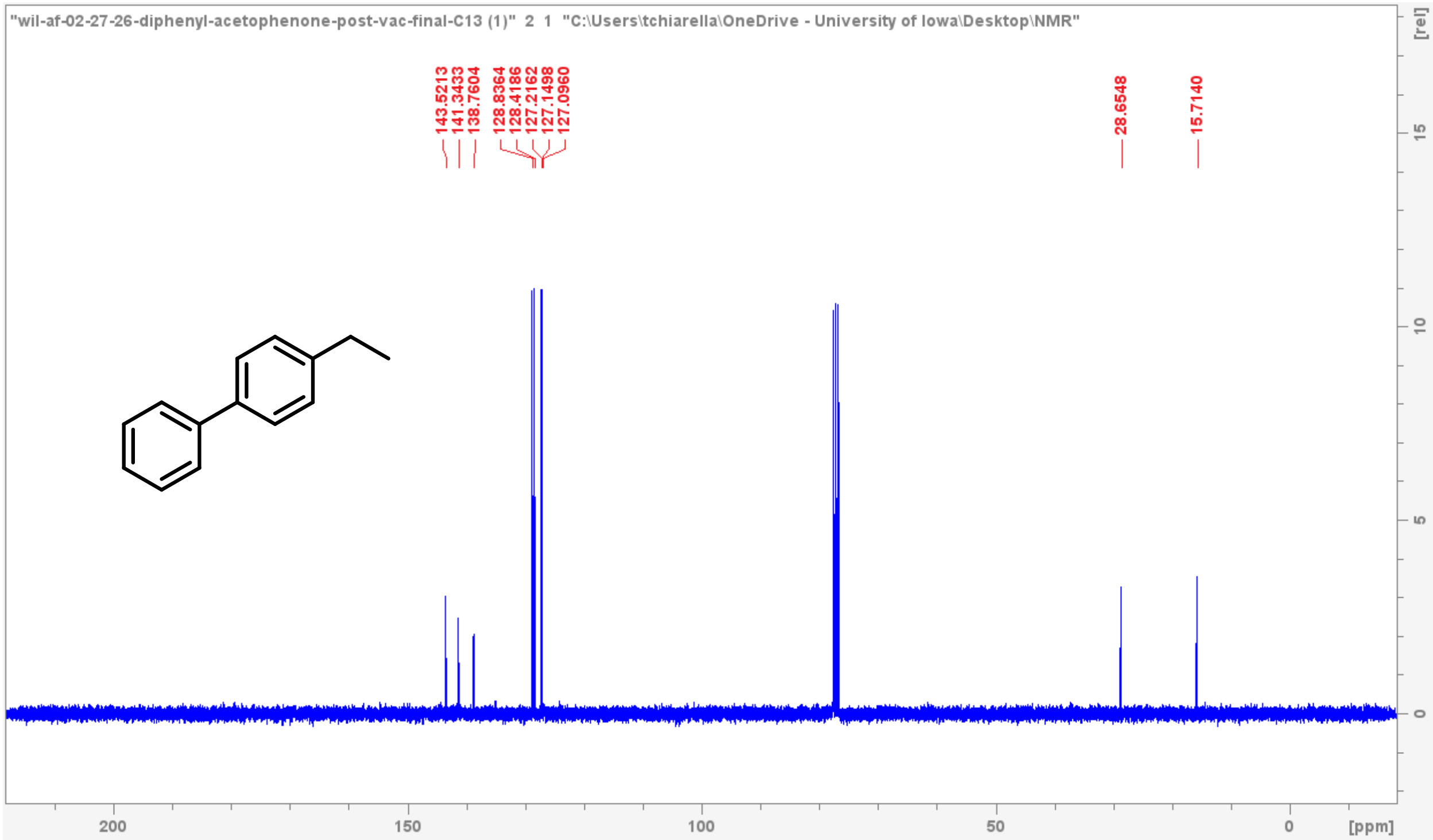

Figure S66: ^{1}H-NMR (400 MHz) of 4k in $CDCl_3$

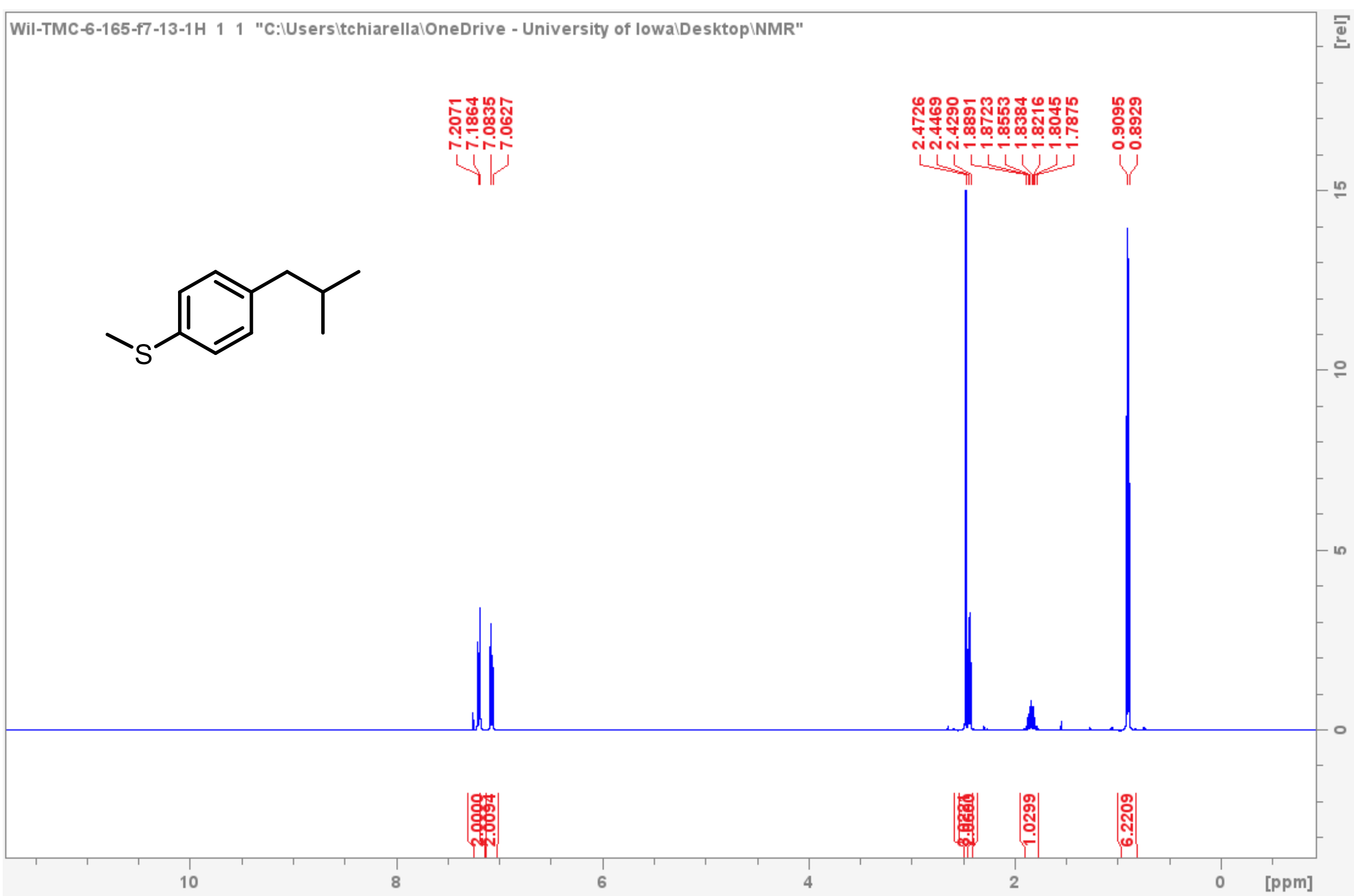

Figure S67: Zoomed in ^{1}H-NMR (400 MHz) of 4k in $CDCl_3$

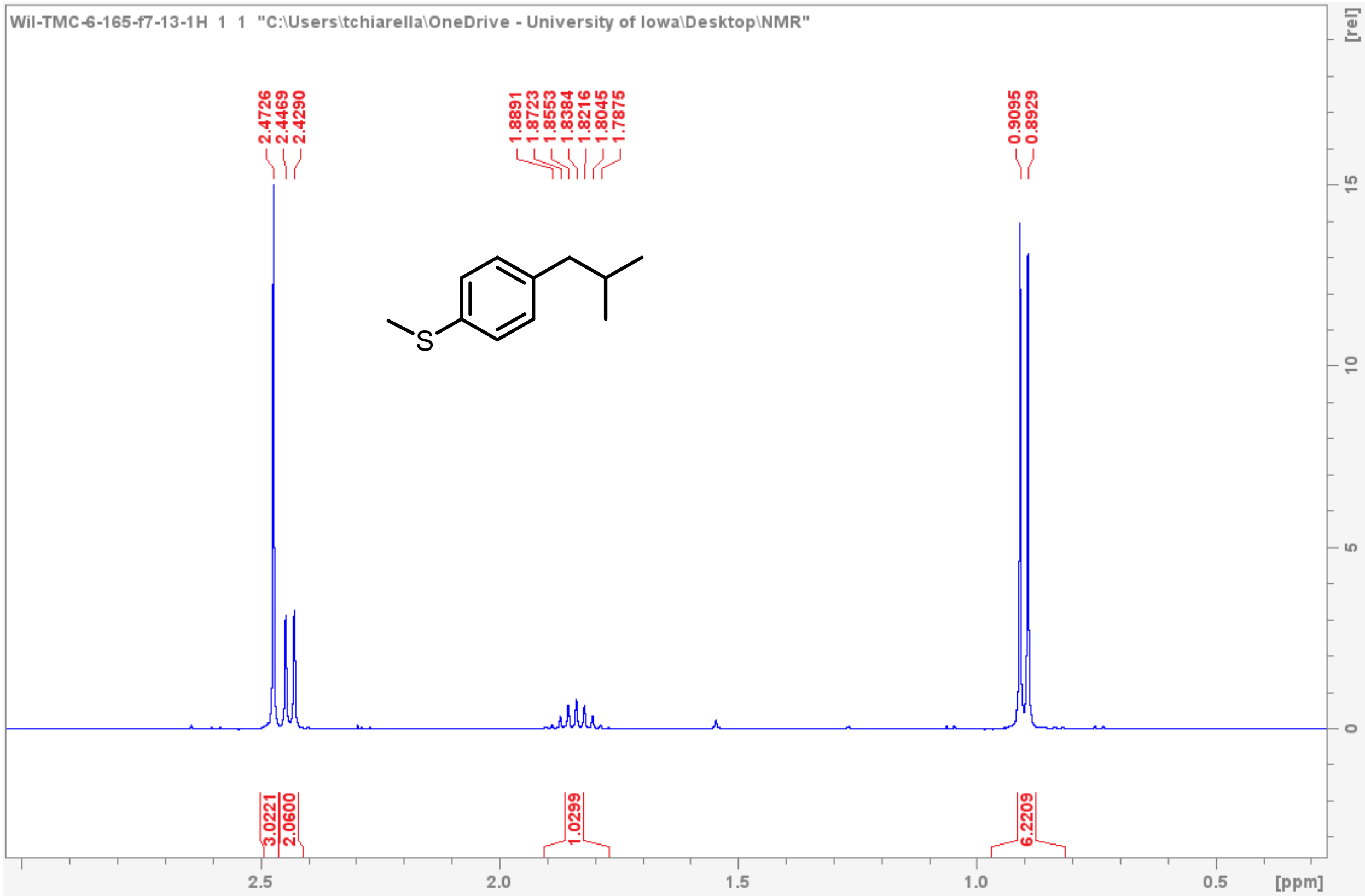

Figure S68: $^{13}C\{^{1}H\}$-NMR (100 MHz) of 4k in $CDCl_3$

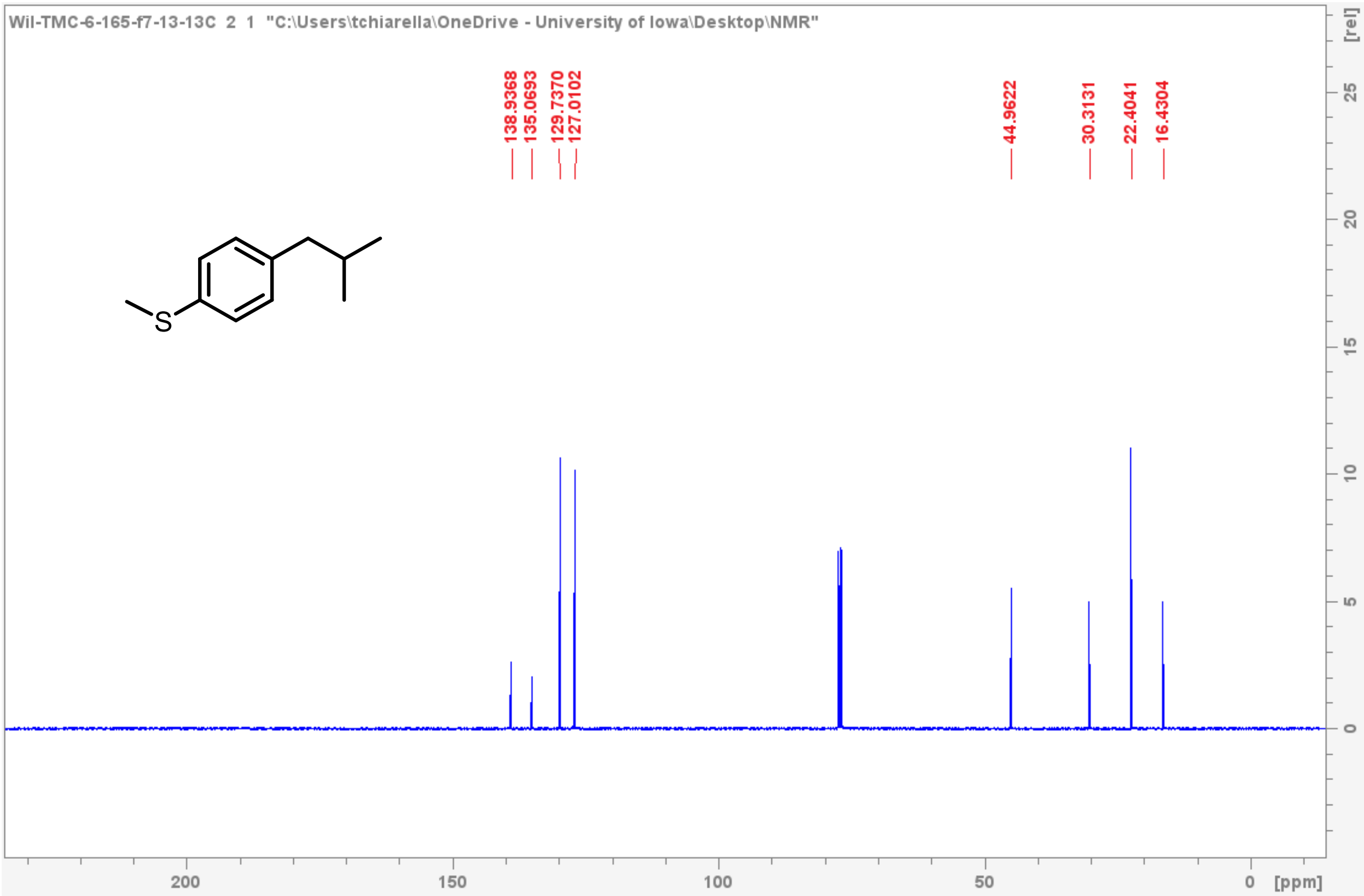

Figure S69: HSQC of 4k in $CDCl_3$

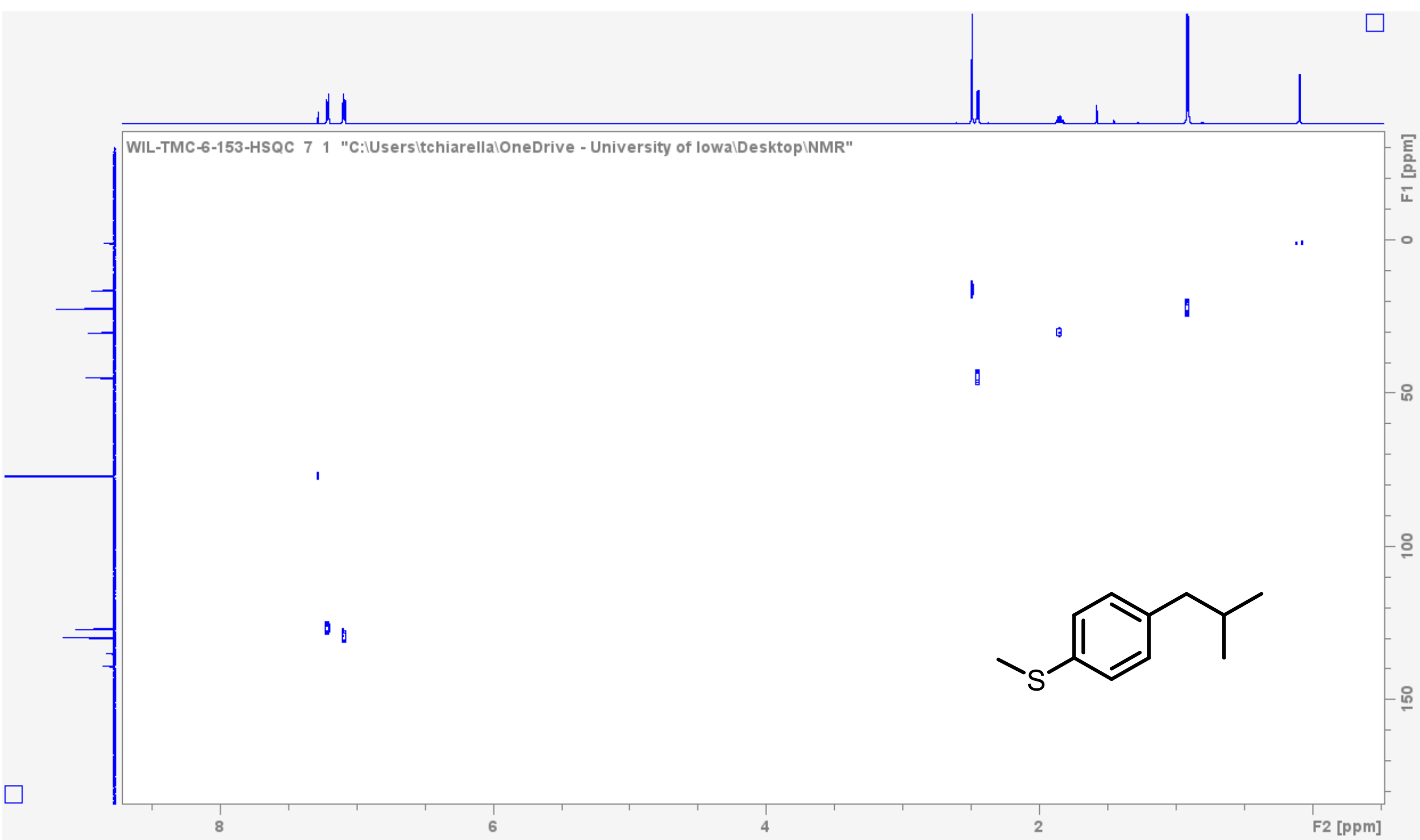

Figure S70: ^{1}H-NMR (300 MHz) of 5l in $CDCl_3$

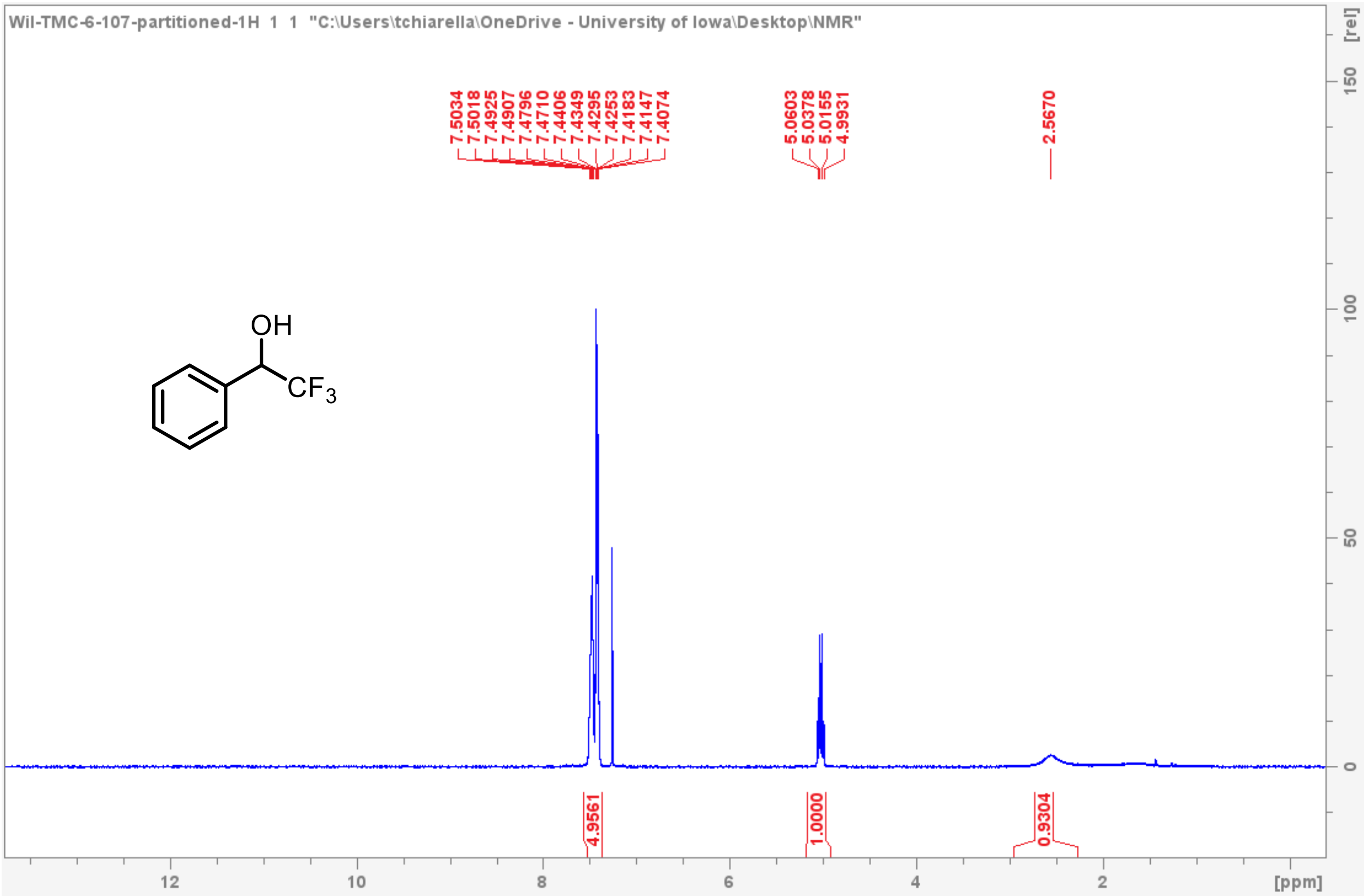

Figure S71: ^{13}C{^{1}H}-NMR (75 MHz) of 5l in $CDCl_3$

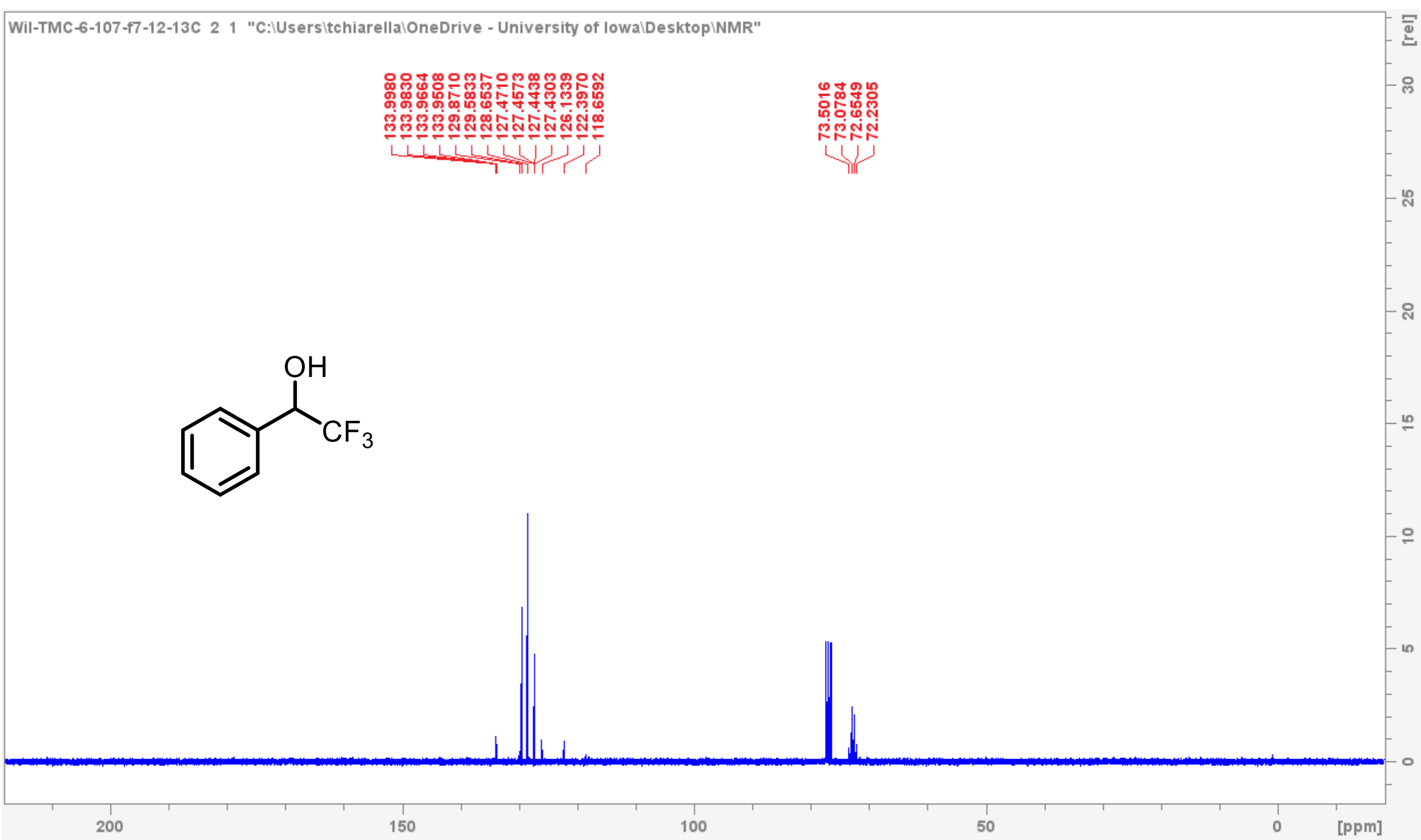

Figure S72: ^{19}F-NMR (282 MHz) of 5l in $CDCl_3$

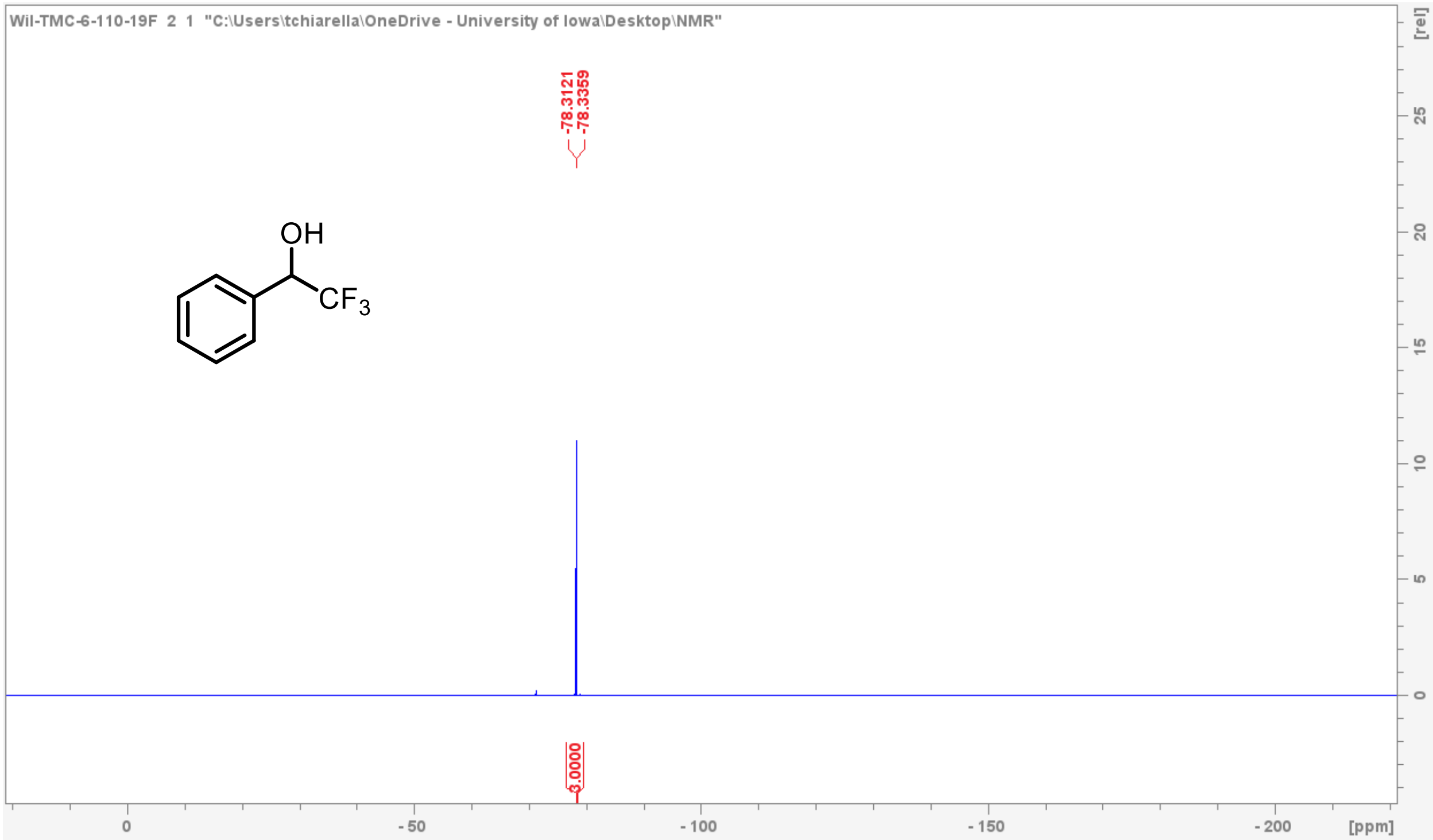

Figure S73: ^{1}H-NMR (400 MHz) of 5m in $CDCl_3$

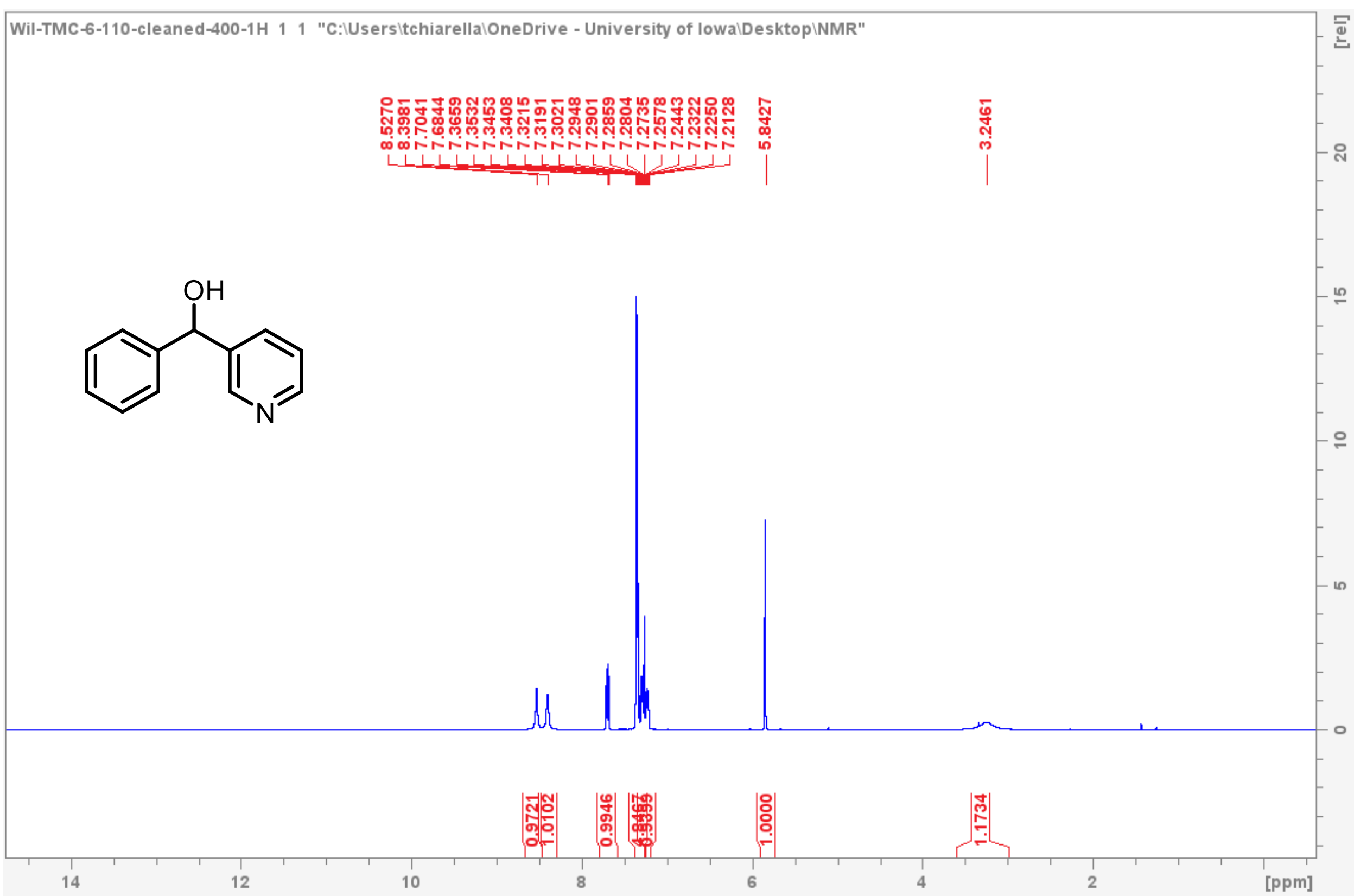

Figure S74: $^{13}C\{^{1}H\}$-NMR (100 MHz) of 5m in $CDCl_3$

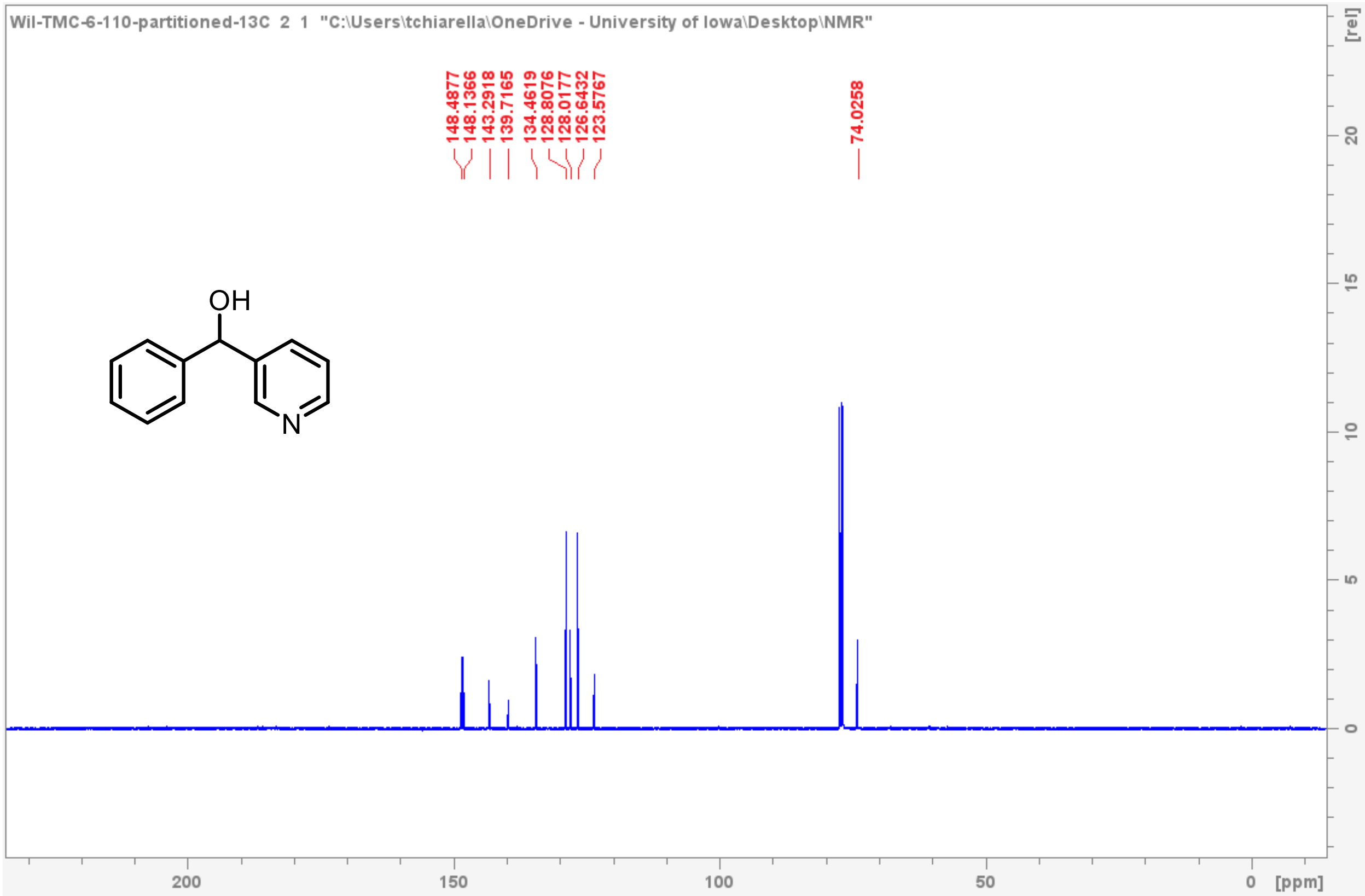

Figure S75: Stacked ^{1}H-NMR of commercial tetraphenylethylene (top) and partially isolated tetraphenylene (bottom)

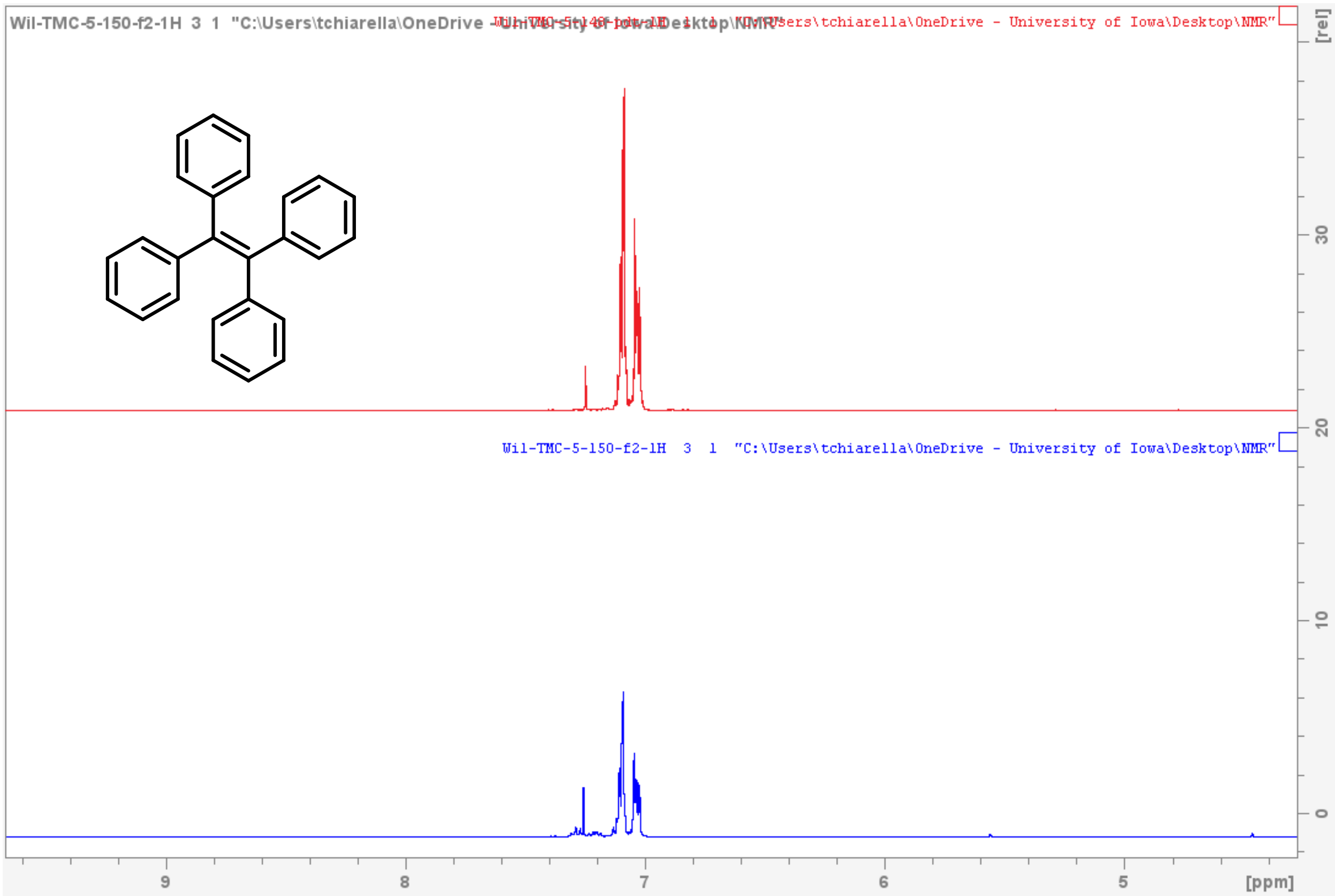

Figure S76: Stacked $^{13}C\{^1H\}$-NMR of commercial tetraphenylethylene (top) and partially isolated tetraphenylene (bottom)

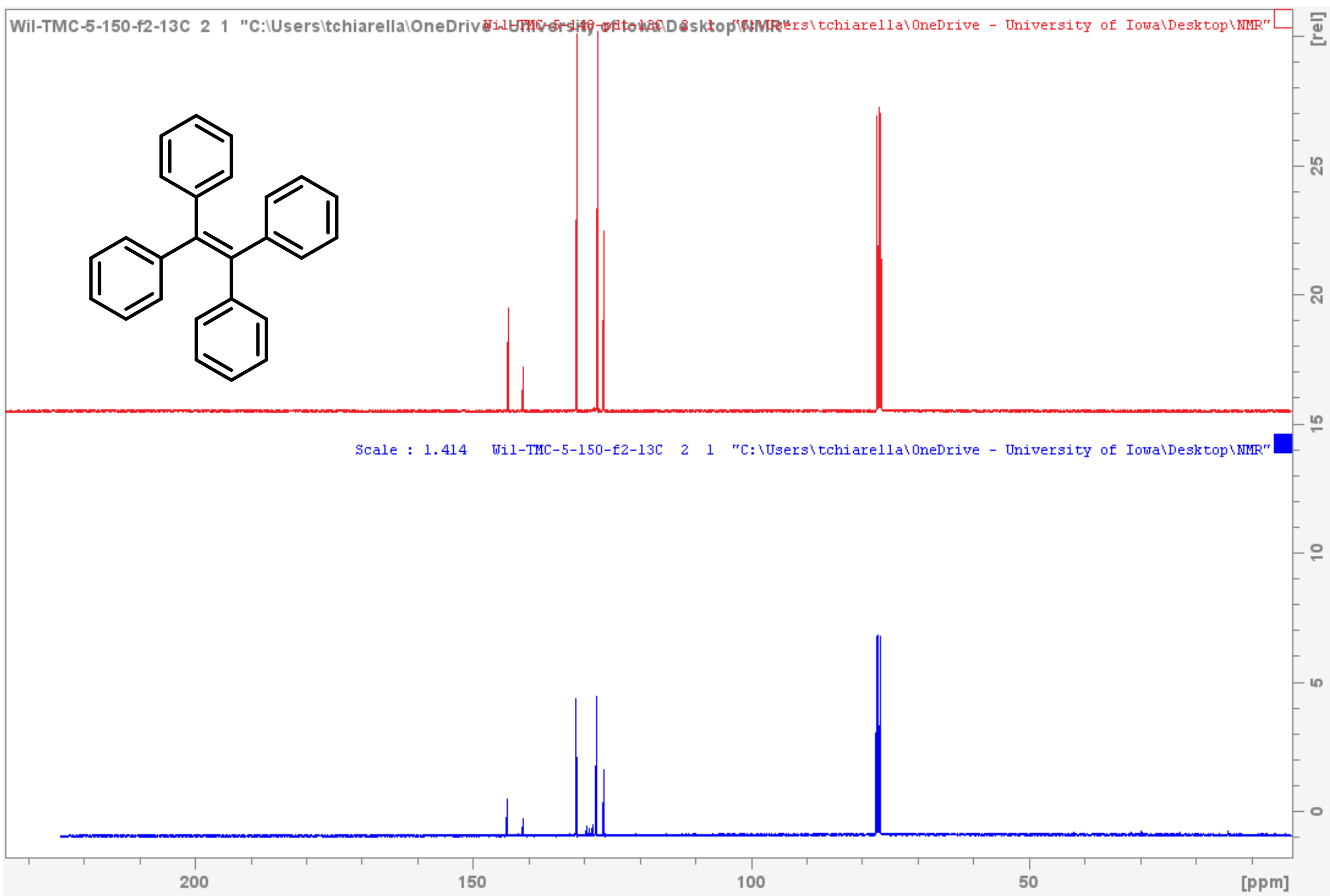

Figure S77: Annotated GC data for LFER experiment

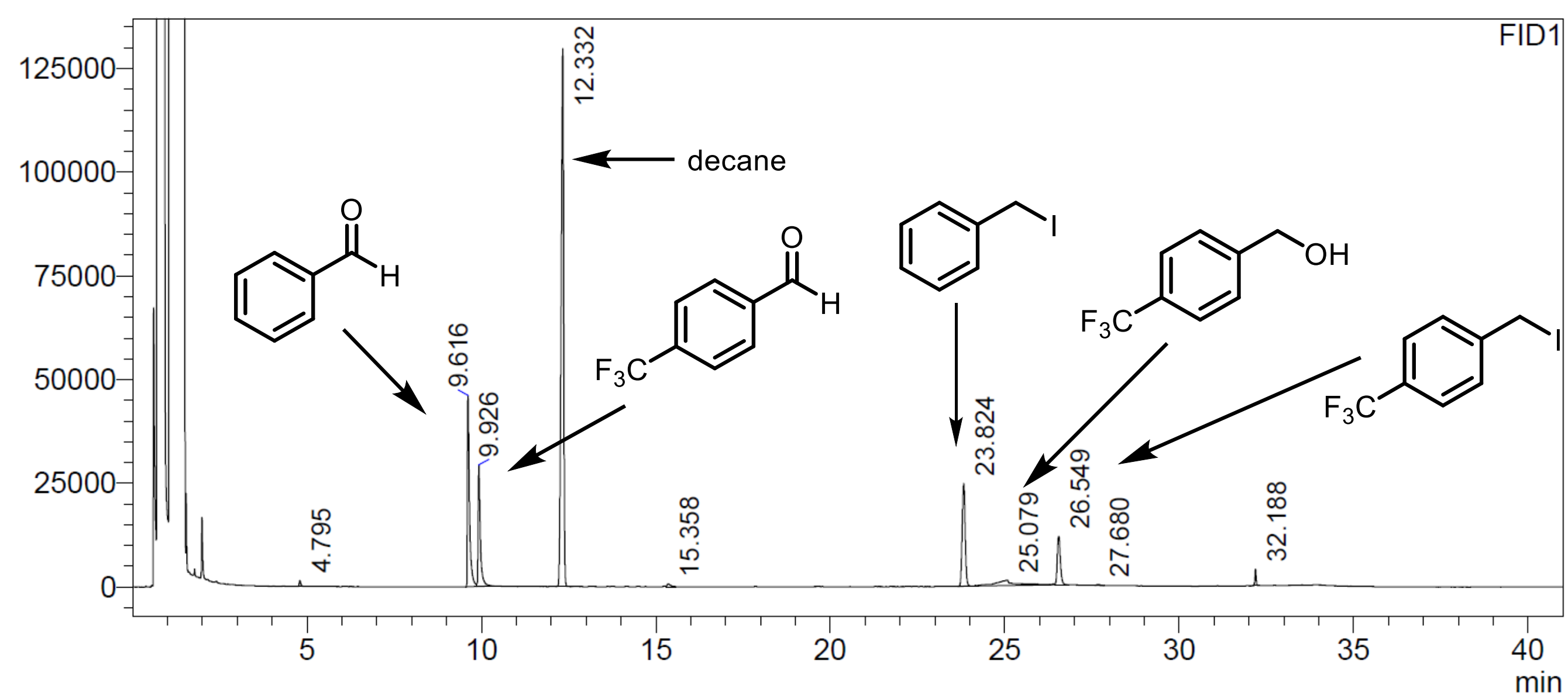

Figure S78: GC-MS spectrum of 4o

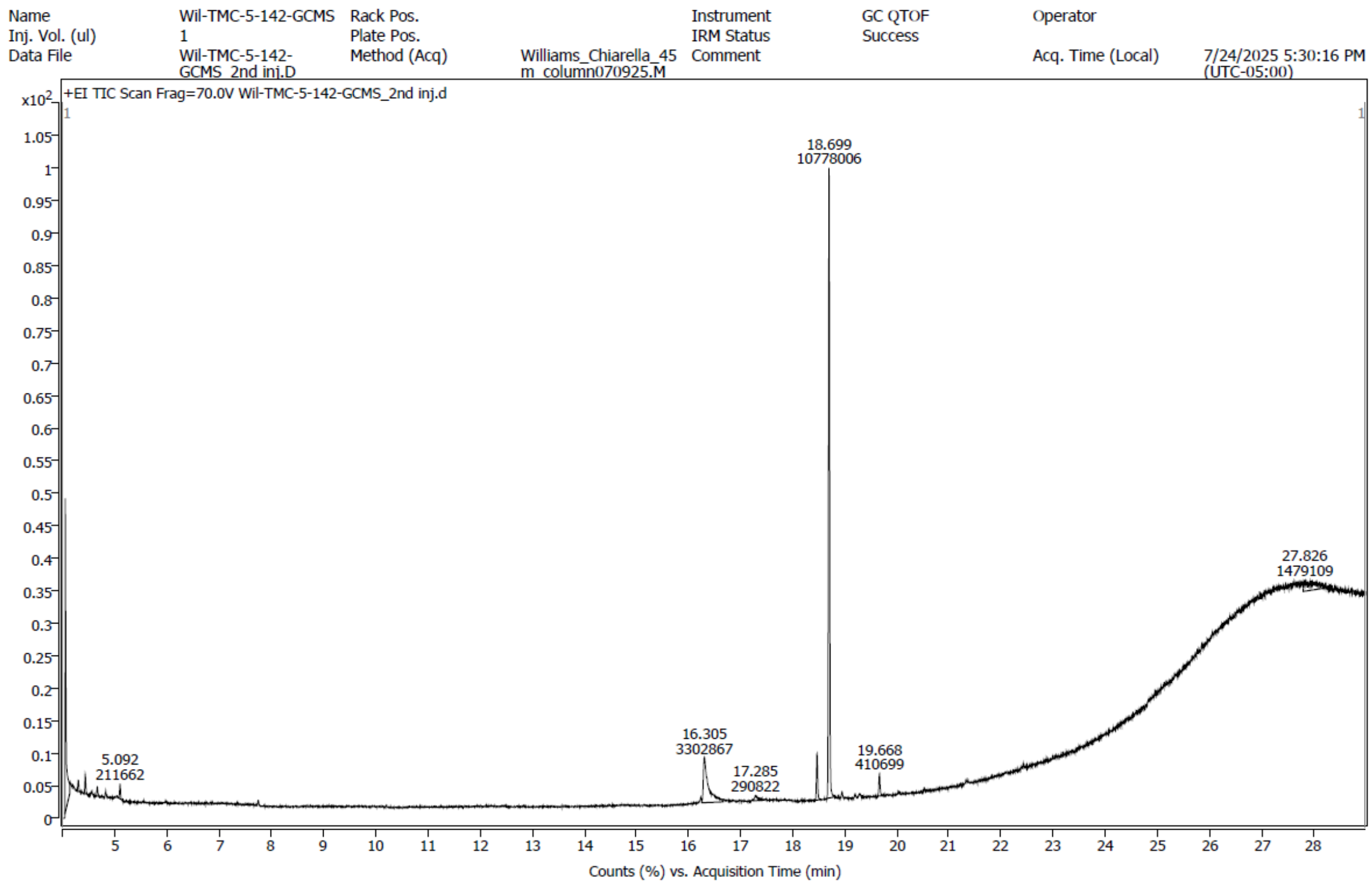

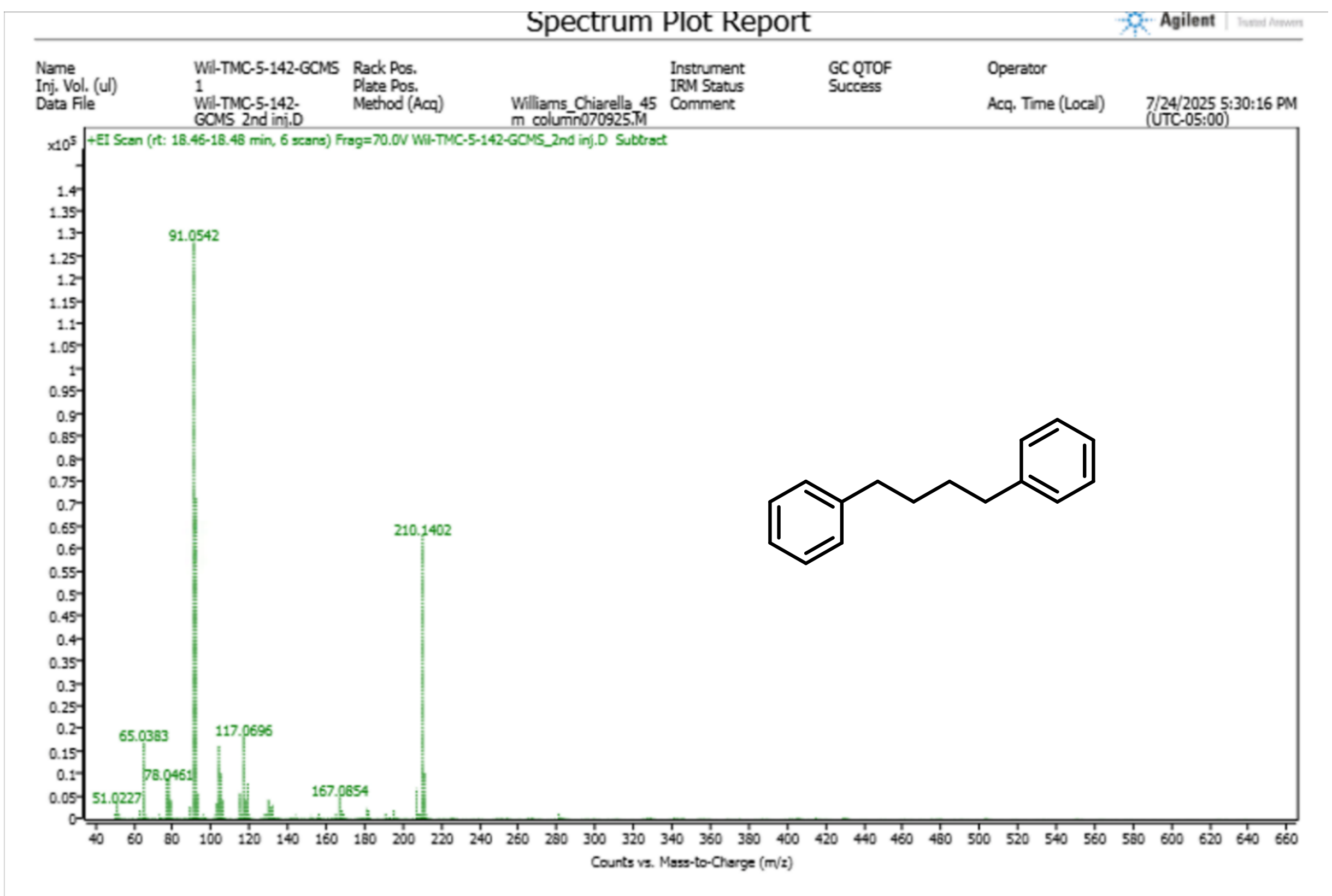

2. References:

(1) Trillo, P.; Slagbrand, T.; Adolfsson, H. Straightforward α-Amino Nitrile Synthesis Through Mo(CO)6-Catalyzed Reductive Functionalization of Carboxamides. *Angew. Chem. Int. Ed.* **2018**, *57* (38), 12347-12351. DOI: https://doi.org/10.1002/anie.201807735.
(2) Lin, Y.; Zhu, L.; Lan, Y.; Rao, Y. Development of a Rhodium(II)-Catalyzed Chemoselective C(sp3)–H Oxygenation. *Chem. Eur. J.* **2015**, *21* (42), 14937-14942. DOI: https://doi.org/10.1002/chem.201502140.
(3) Ćorković, A.; Chiarella, T.; Williams, F. J. Boron Triiodide-Mediated Reduction of Nitroarenes Using Borohydride Reagents. *Org. Lett.* **2023**, *25* (49), 8787-8791. DOI: 10.1021/acs.orglett.3c03257.
(4) Zhou, P.; Li, Y.; Xu, T. Molybdenum-Catalyzed Cross-Coupling of Benzyl Alcohols: Direct C–OH Bond Transformation via [2 + 2]-Type Addition and Elimination. *Org. Lett.* **2022**, *24* (23), 4218-4223. DOI: 10.1021/acs.orglett.2c01537.
(5) Ortiz-Trankina, L. N.; Crain, J.; Williams, C.; Mack, J. Developing benign syntheses using ion pairs via solvent-free mechanochemistry. *Green Chem.* **2020**, *22* (11), 3638-3642, 10.1039/D0GC01116A.
(6) Langer, P.; Yang, L.; Pfeiffer, C. R.; Lewis, W.; Champness, N. R. Restricting shuttling in bis(imidazolium)...pillar[5]arene rotaxanes using metal coordination. *Dalton Trans.* **2019**, *48* (1), 58-64, 10.1039/C8DT04096F.
(7) Mai, T. T.; Viswambharan, B.; Gori, D.; Guillot, R.; Naubron, J.-V.; Kouklovsky, C.; Alezra, V. Frozen Chirality of Tertiary Aromatic Amides: Access to Enantioenriched Tertiary α-Amino Acid or Amino Alcohol without Chiral Reagent. *Chem. Eur. J.* **2017**, *23* (24), 5787-5798. DOI: https://doi.org/10.1002/chem.201700110.
(8) Solladie-Cavallo, A.; Diep-Vohuule, A. A Two-Step Asymmetric Synthesis of (R)-Monoaryl Epoxides Using a Chiral Oxathiane as a Recoverable Reagent: Application to the Preparation of (R)-.beta.-Adrenergic Compounds. *J. Org. Chem.* **1995**, *60* (11), 3494-3498. DOI: 10.1021/jo00116a041.
(9) Combe, S. H.; Hosseini, A.; Song, L.; Hausmann, H.; Schreiner, P. R. Catalytic Halogen Bond Activation in the Benzylic C–H Bond Iodination with Iodohydantoins. *Org. Lett.* **2017**, *19* (22), 6156-6159. DOI: 10.1021/acs.orglett.7b03034.
(10) Salamanca-Perdigón, K.; Hurtado-Rodríguez, D.; Portilla, J.; Iriepa, I.; Rojas, H.; Becerra, D.; Castillo, J.-C. Cs2CO3-Promoted Alkylation of 3-Cyano-2(1H)-Pyridones: Anticancer Evaluation and Molecular Docking. *ChemPlusChem* **2024**, *89* (9), e202400172. DOI: https://doi.org/10.1002/cplu.202400172.
(11) Weidmann, N.; Harenberg, J. H.; Knochel, P. Continuous Flow Preparation of (Hetero)benzylic Lithiums via Iodine–Lithium Exchange Reaction under Barbier Conditions. *Org. Lett.* **2020**, *22* (15), 5895-5899. DOI: 10.1021/acs.orglett.0c01991.
(12) Epifanov, M.; Mo, J. Y.; Dubois, R.; Yu, H.; Sammis, G. M. One-Pot Deoxygenation and Substitution of Alcohols Mediated by Sulfuryl Fluoride. *J. Org. Chem.* **2021**, *86* (5), 3768-3777. DOI: 10.1021/acs.joc.0c02557.
(13) Pogránýi, B.; Mielke, T.; Díaz-Rodríguez, A.; Cartwright, J.; Unsworth, W. P.; Grogan, G. Preparative-Scale Biocatalytic Oxygenation of N-Heterocycles with a Lyophilized Peroxygenase Catalyst. *Angew. Chem. Int. Ed.* **2023**, *62* (5), e202214759. DOI: https://doi.org/10.1002/anie.202214759 (acccessed 2026/06/16).
(14) Wang, H.; Li, L.; Bai, X.-F.; Shang, J.-Y.; Yang, K.-F.; Xu, L.-W. Efficient Palladium-Catalyzed C–O Hydrogenolysis of Benzylic Alcohols and Aromatic Ketones with Polymethylhydrosiloxane. *Adv. Synth. Catal.* **2013**, *355* (2-3), 341-347. DOI: https://doi.org/10.1002/adsc.201200832.
(15) Köring, L.; Stepen, A.; Birenheide, B.; Barth, S.; Leskov, M.; Schoch, R.; Krämer, F.; Breher, F.; Paradies, J. Boron-Centered Lewis Superacid through Redox-Active Ligands: Application in C−F and S−F Bond Activation. *Angew. Chem. Int. Ed.* **2023**, *62* (13), e202216959. DOI: https://doi.org/10.1002/anie.202216959.
(16) Pichette Drapeau, M.; Tlili, A.; Zaid, Y.; Toummini, D.; Ouazzani Chahdi, F.; Sotiropoulos, J.-M.; Ollevier, T.; Taillefer, M. Transition-Metal-Free Synthesis of Biarylmethanes from Aryl Iodides and Benzylic Ketones. *Chem. Eur. J.* **2018**, *24* (66), 17449-17453. DOI: https://doi.org/10.1002/chem.201804415.
(17) Tang, R.-J.; Milcent, T.; Crousse, B. Bisulfate Salt-Catalyzed Friedel–Crafts Benzylation of Arenes with Benzylic Alcohols. *J. Org. Chem.* **2018**, *83* (22), 14001-14009. DOI: 10.1021/acs.joc.8b02361.
(18) Morimoto, K.; Itoh, M.; Hirano, K.; Satoh, T.; Shibata, Y.; Tanaka, K.; Miura, M. Synthesis of Fluorene Derivatives through Rhodium-Catalyzed Dehydrogenative Cyclization. *Angew. Chem. Int. Ed.* **2012**, *51* (22), 5359-5362. DOI: https://doi.org/10.1002/anie.201201526.

(19) Dai, W.-C.; Yang, B.; Xu, S.-H.; Wang, Z.-X. Nickel-Catalyzed Cross-Coupling of Aryl 2-Pyridyl Ethers with Organozinc Reagents: Removal of the Directing Group via Cleavage of the Carbon–Oxygen Bonds. *J. Org. Chem.* **2021**, *86* (3), 2235-2243. DOI: 10.1021/acs.joc.0c02389.
(20) Zhang, D.; Xu, Z.; Tang, T.; Le, L.; Wang, C.; Kambe, N.; Qiu, R. Pd-Catalyzed Cross-Coupling of Sb-Aryl Stibines with Halogenomethyl Arenes to Give Unsymmetirc Diarylmethanes. *Org. Lett.* **2022**, *24* (17), 3155-3160. DOI: 10.1021/acs.orglett.2c00879.
(21) Jiang, W.; Leng, X.; Yang, Y.; Lu, L.; Shen, Q. CageCarbene-Ir Complex Enables Selective Alkylation and Alkenylation of Benzyl Alcohols with Alkyl Sulfones. *Chin. J. Chem.* **2025**, *43* (24), 3582-3588. DOI: https://doi.org/10.1002/cjoc.70297.
(22) Li, Z.; Liu, G.; Huang, Z. Catalytic Direct Deoxygenative Alkylation of Alcohols with Sulfones. *J. Org. Chem.* **2025**, *90* (31), 11390-11394. DOI: 10.1021/acs.joc.5c01079.
(23) Annibaletto, J.; Jacob, C.; Theunissen, C. Ammonium Salts as Convenient Radical Precursors Using Iridium Photoredox Catalysis. *Org. Lett.* **2022**, *24* (23), 4170-4175. DOI: 10.1021/acs.orglett.2c01407.
(24) Matsubara, K.; Mitsuyama, T.; Shin, S.; Hori, M.; Ishikawa, R.; Koga, Y. Homoleptic Cobalt(II) Phenoxyimine Complexes for Hydrosilylation of Aldehydes and Ketones without Base Activation of Cobalt(II). *Organometallics* **2021**, *40* (9), 1379-1387. DOI: 10.1021/acs.organomet.1c00151.
(25) Nishigaya, Y.; Umei, K.; Watanabe, D.; Kohno, Y.; Seto, S. PBr3-mediated unexpected reductive deoxygenation of α-aryl-pyridinemethanols: synthesis of arylmethylpyridines. *Tetrahedron* **2016**, *72* (12), 1566-1572. DOI: https://doi.org/10.1016/j.tet.2016.02.005.
